# Palladium‐Catalyzed C(sp^3^)−H Arylation of Primary Amines Using a Catalytic Alkyl Acetal to Form a Transient Directing Group

**DOI:** 10.1002/chem.201804515

**Published:** 2018-11-08

**Authors:** Sahra St John‐Campbell, Alex K. Ou, James A. Bull

**Affiliations:** ^1^ Department of Chemistry Imperial College London, Molecular Sciences Research Hub, White City Campus Wood Lane London W12 0BZ UK

**Keywords:** amines, C−H functionalization, homogeneous catalysis, palladium, reaction kinetics

## Abstract

C−H Functionalization of amines is a prominent challenge due to the strong complexation of amines to transition metal catalysts, and therefore typically requires derivatization at nitrogen with a directing group. Transient directing groups (TDGs) permit C−H functionalization in a single operation, without needing these additional steps for directing group installation and removal. Here we report a palladium catalyzed γ‐C−H arylation of amines using catalytic amounts of alkyl acetals as transient activators (e.g. commercially available (2,2‐dimethoxyethoxy)benzene). This simple additive enables arylation of amines with a wide range of aryl iodides. Key structural features of the novel TDG are examined, demonstrating an important role for the masked carbonyl and ether functionalities. Detailed kinetic (RPKA) and mechanistic investigations determine the order in all reagents, and identify cyclopalladation as the turnover limiting step. Finally, the discovery of an unprecedented off‐cycle free‐amine directed *ϵ*‐cyclopalladation of the arylation product is reported.

## Introduction

**Scheme 1:**
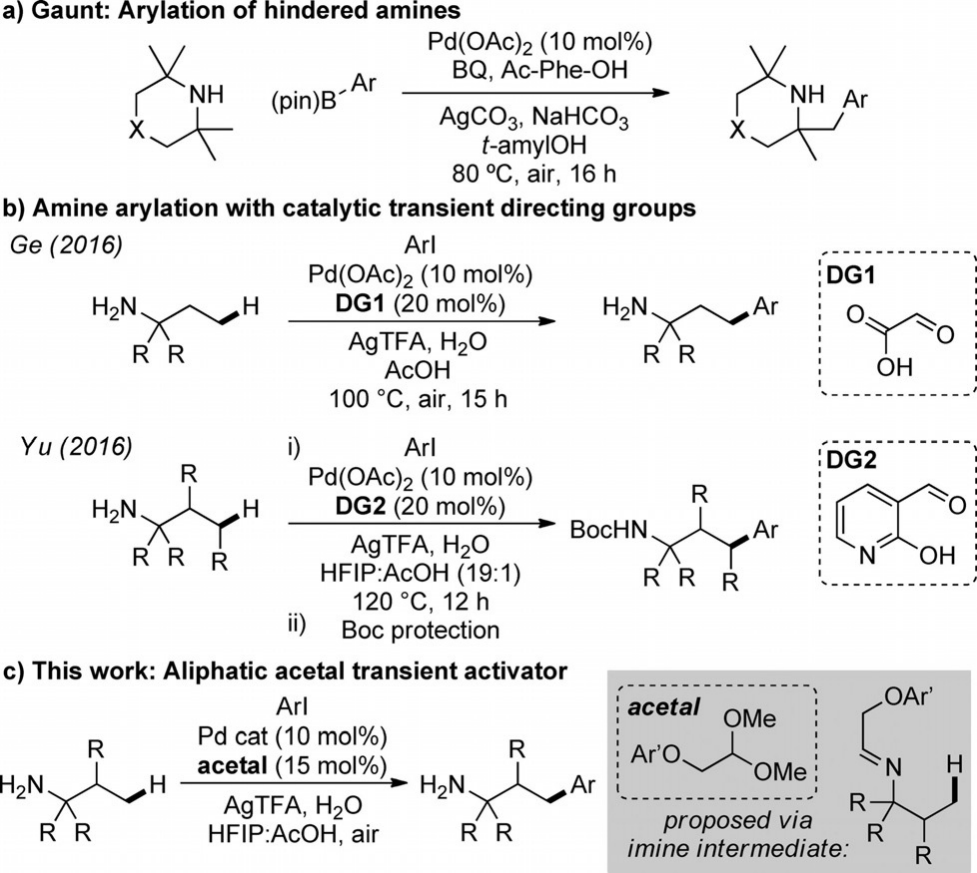
Palladium catalyzed C(sp^3^)−H arylation of unprotected amines.

Amines are crucial structural features in biological molecules, pharmaceuticals and agrochemical products.^1^ Numerous simple aliphatic amines are commercially available and react at nitrogen with well‐established transformations. In contrast, derivatization of amines by C−C bond formation at unactivated positions remains a frontier challenge in synthesis. Transition‐metal‐catalyzed C(sp^3^)−H functionalization offers huge potential for such value adding transformations of feedstock chemicals.^2^ However, free amine substrates deactivate metal catalysts by strong coordination, and readily undergo oxidation by β‐hydride elimination,^3^ typically preventing the desired transformation. Important recent developments have derivatized nitrogen with amide or sulfonamide directing groups to modify the coordinating ability of the amine and to direct C−H functionalization.^4^ This requires additional steps for directing group installation and removal. Very few C(sp^3^)−H functionalization methods have been successful on unprotected amines. Notably, Gaunt developed palladium‐catalyzed functionalization of hindered secondary amine substrates,^5^ to avoid formation of inactive bis(amine) palladium complexes, including the arylation of tetramethylpiperidine derivatives (Scheme 1 a).5d Shi reported acetoxylation of *t*‐amylamine in AcOH with diacetoxyiodobenzene as the oxidant, minimizing inactive amine‐Pd complexes by protonation.^6^

Very recently, transient directing groups (TDGs) have been applied to the functionalization of C(sp^3^)−H bonds,^7^ particularly arylation of aldehyde and ketone substrates via *endo*‐imines formed in situ with amine additives.^8^ The use of *exo*‐imine directing groups for C−H functionalization of primary amines remains rare, with examples using palladium catalysis.^9^, ^10^, ^11^, ^12^ In 2016 Dong used stoichiometric 8‐formylquinoline to form an imine which effected arylation with bisaryliodonium salts.^9^ At a similar time, Ge reported the catalytic use of glycoxylic acid to form a transient imine directing group for arylation of *t*‐amylamine derivatives (Scheme 1 b).^10^ Yu reported catalytic 2‐hydroxynicotinaldehyde as a highly effective directing group, compatible with propylamine as well as α‐ and β‐branched amines.^11^ In 2017 Murakami used a stoichiometric salicaldehyde in separate imine formation and hydrolysis steps, completed in a one‐pot sequence.^12^ While preparing this manuscript, Young reported the use of carbon dioxide to promote C−H arylation of primary and secondary aliphatic amines.^13^

Here we report new transient directing groups for C−H functionalization of amines with the first examples of alkyl aldehydes as transient directing groups (Scheme 1 c). The use of a stable acetal precursor in low catalytic loadings promotes the γ‐arylation of primary amines with aryl iodides using palladium catalysis. Furthermore, we report detailed kinetic (RPKA) and mechanistic investigations. These studies provided important insights into the reaction, including reaction orders of all components and the identification of an unusual *ϵ*‐cyclometallation pathway of the arylated products.

## Results and Discussion

We envisaged using alkyl aldehydes as TDGs for primary amine C−H arylation in order to aid reversible imine formation and open new possibilities for transient directing groups in C−H functionalization. This would enable a second coordinating site to be included at the α‐position of the aldehyde, which could be readily tuned to optimize the properties. The secondary coordinating groups were intended to provide a chelate to stabilize intermediates on the catalytic cycle,^14^ limit imine isomerization, increase aldehyde electrophilicity and prevent cyclometalation of the directing group. However, such functionalized aldehydes are typically unstable to storage, and therefore we considered the use of acetals as more accessible alternatives, being easily prepared and commercially available. This would be the first example of this type of transient directing group in any C−H functionalization reaction.

**Scheme 2:**
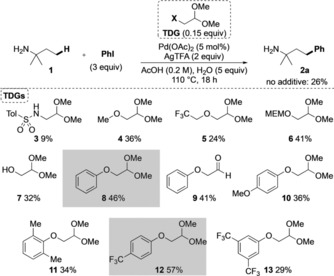
Screen of alkyl acetals as transient directing groups for amine arylation. Yields determined by ^1^H NMR using 1,3,5‐trimethoxybenzene as an internal standard.

After initial investigation, a screen of different potential TDGs was conducted using palladium acetate with silver trifluoroacetate in acetic acid and water as solvent (Scheme 2). Under these conditions, in the absence of any additional TDG using *t*‐amylamine **1**, the free amine promoted the arylation in 26 % yield, which we aimed to improve by invoking a transient imine strategy. In comparison to our previous work using mono‐*N*‐Ts ethylenediamine as a TDG for aldehyde C−H arylation,8c we first considered 2‐*N*‐tosyl acetaldehyde acetal **3**. Addition of 0.15 equivalents of the acetal gave a decreased yield compared to the background reaction, presumably due to strong coordination to the palladium catalyst. However, an improved yield was achieved by using methoxy derivative **4**. Trifluoroethyl ether **5** had no beneficial effect on the reaction. A potentially tridentate TDG containing a MEM protected alcohol **6** improved the yield to 41 %, whereas the corresponding free alcohol **7** gave 32 % yield. Pleasingly, commercially available phenoxyacetaldehyde dimethyl acetal **8** gave an improved 46 % yield. Its corresponding aldehyde **9** gave a similar result, consistent with hydrolysis under the reaction conditions. We then investigated variations to the aromatic ether. *p*‐Methoxy substituted derivative **10** was less effective than the phenyl ether, and the more hindered 2,6‐dimethoxy example **11** also gave a lower yield. Electron‐poor 4‐trifluoromethylphenyl derivative **12** gave the highest yield of 57 %. Further decreasing the electron density of the ring was detrimental (**13**, 29 %).

**Scheme 3:**
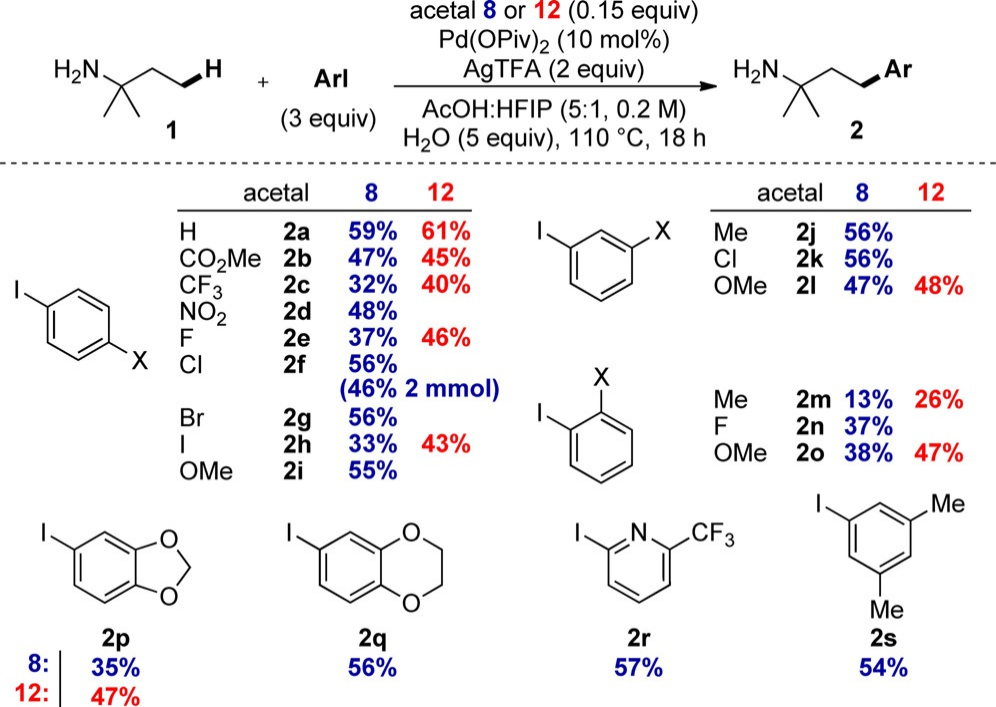
Reaction scope of aryl iodides using acetals **8** and **12** as transient directing groups.

Further optimization of the reaction conditions was conducted with commercially available **8** (0.15 equiv), which improved the yield of **2 a** to 64 % (by ^1^H NMR) and 59 % isolated yield, by using palladium pivalate (10 mol %), AgTFA, and a solvent mixture of AcOH:HFIP:H_2_O.^15^ The use of silver salts was essential. Notably, a similar maximum yield was achieved with acetal **12**. Using these conditions, the reaction was shown to be compatible with a range of aryl iodides (Scheme 3).

Both directing groups **8** and **12** were investigated: acetal **12** gave comparable and often improved yields especially for examples when **8** was less effective. In all cases, the product amines were isolated in analytically pure form by simple aqueous work‐up, without chromatography. Aryl iodides bearing both electron‐withdrawing and electron‐donating functionality at the *para*‐ and *meta*‐positions were successful to give amines **2 a**–**l**. Halogens were well tolerated, providing positions for further derivatization. Chlorophenyl derivative **2 f** was demonstrated on a 2 mmol scale with 46 % isolated yield, again involving only a simple work up to provide the pure amine compound. Pleasingly, even *ortho*‐substituted aryl iodides (Me, F and OMe), which are often incompatible with Pd‐catalyzed C−H arylation, were successful giving **2 m–o** without a significant reduction in yield, particularly using acetal **12**. More complex aryl iodides were also tolerated (**2 p–s**), including heterocyclic derivatives.

**Scheme 4:**
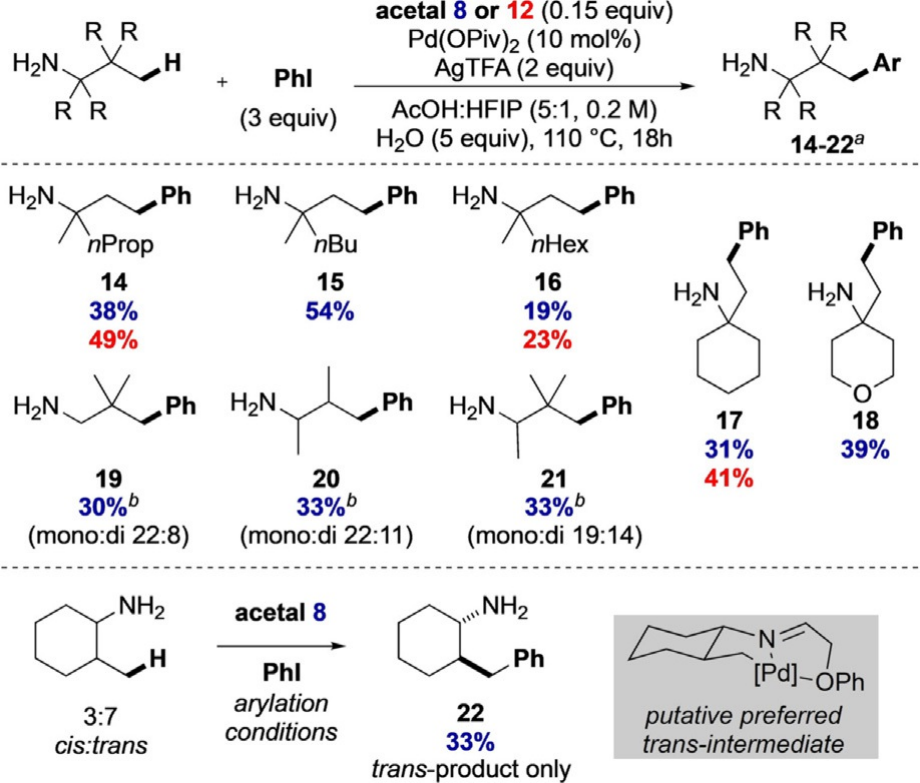
Reaction scope of aliphatic amines. [a] Yields in blue refer to acetal **8**, and red to acetal **12**. [b] Using 1.5 equiv AgTFA, 3 h.

We then investigated the amine component with iodobenzene as the coupling partner (Scheme 4). Using derivatives with longer alkyl substituents gave similar yields and displayed selective arylation at the γ‐CH_3_ position (**14** and **15**). Amine **16** with a long hexyl chain gave a lower yield. Cyclohexyl and THP containing amines were suitable substrates for the arylation, affording **17** and **18** in good yields in this operationally simple one‐pot process. Arylation of γ‐methylene positions was not observed for any example.

Interestingly, when using neopentylamine under our standard conditions arylated pivaldehyde derivatives were detected due to competing oxidation processes.^15^ Therefore, milder reaction conditions were developed for this family of amines using a reduced reaction time and silver trifluoroacetate loading (3 h, 1.5 equiv AgTFA) to reduce oxidation. These afforded an improved isolated yield for arylated neopentylamine **19** (30 %, combined mono and diarylation). α,β‐Branched amines were also arylated under these modified conditions to afford products **20** and **21**, both in 33 % yield. When using a commercial mixture of *cis*‐ and *trans*‐2‐methylcyclohexanamine the only product corresponded to *trans*‐**22** (33 % yield); no arylation occurred on the *cis*‐diastereomer indicating a high selectivity. In comparison, under the same conditions Yu's 2‐hydroxy‐nicotinaldehyde transient directing group^11^ gave a mixture of products consisting of both *trans*‐ and *cis*‐arylation as well as further arylation on other γ‐positions.

**Scheme 5:**
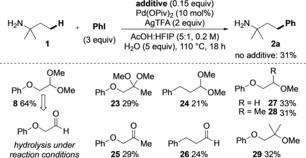
Analysis of structural features of the optimal directing group. Yields determined by ^1^H NMR using 1,3,5‐trimethoxybenzene as an internal standard.

Next, structural comparisons were undertaken to indicate the structural features of the acetal which were important in promoting the arylation (Scheme 5). Ketal **23** did not improve the yield above the background reaction, suggesting restricted imine formation. Hydrocinnamaldehyde acetal **24**, without the ether oxygen, gave a lower yield than in absence of any additive, indicating the importance of the OAr group in forming the putative bidentate TDG. In each case, the corresponding carbonyl species **25** and **26** gave similar yields, again supporting the rapid hydrolysis of the acetals under the reaction conditions. Catalytic acetal **8** was shown by ^1^H NMR to readily hydrolyse to the aldehyde under the reaction conditions. The use of ethers **27**–**29**, with similar steric and coordinating properties to acetal **8** were unable to enhance the yield, again supporting the need for imine formation.

Based on these results and prior studies we propose the reaction to be accelerated by formation of a transient imine directing group. Unfortunately, attempts to isolate Pd‐complexes with the imine were unsuccessful. Therefore, we examined the kinetics of the reaction to probe the details of the dual catalytic cycle. To date, there are no reports of kinetic analysis on transient C−H functionalization reactions. Blackmond pioneered reaction progress kinetic analysis (RPKA) to make use of the kinetic information in the full reaction profile,^16^ as well as the use of visual comparisons of concentration profiles.^17^ Burés’ recent advances have provided tools to extend the visual comparison methods to determine reaction order for catalyst^18^ and other reaction components from reaction profiles.^19^ Here, due to the heterogeneous nature of the reactions, data was generated through the quenching of individual reactions, monitoring formation of arylated amine **2 a**.

**Figure 1:**
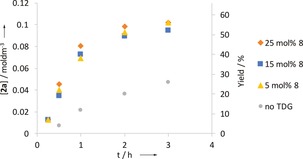
Zero order rate dependence of acetal **8**. Yields determined by ^1^H NMR using 1,3,5‐trimethoxybenzene as an internal standard on discrete reactions.

The overall reaction profile using acetal **8** indicated that the reaction was rapid, with maximum conversion occurring by 3 h and little subsequent change. Unreacted *t*‐amylamine made up the mass‐balance. Using the Burés method, the reaction was shown to be first order in Pd, consistent with a monomeric Pd catalyst. Surprisingly, variation in the loading of acetal **8** had little effect: identical rates were observed at 5, 15 and 25 mol % indicating a zero order (Figure 1).^15^, ^20^ The same rate was observed with acetal **12**, whereas the reaction was considerably slower in the absence of the acetal (with a much lower final yield) suggesting a different mechanism. Different excess experiments for amine **1**, PhI and AgTFA,^21^ also indicated zero order in these components. We also found identical reaction rates with different aryl iodide species (4‐iodoanisole, iodobenzene, and 4‐chloroiodobenzene).^15^ The degree of stirring had little effect on the reaction rate indicating mass transport is not rate limiting.

**Scheme 6:**
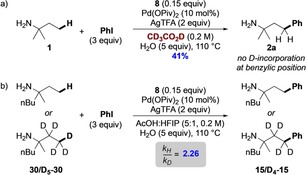
Substrate deuteration and KIE.

To probe the C−H activation step, amine **1** was subjected to the C−H arylation conditions with PhI using AcOD‐D_4_ as the solvent. No d‐incorporation was observed at the benzylic position of the arylated product, suggesting irreversible C−H activation (Scheme 6 a). Furthermore, comparison of the rate of formation of amines **15** and **D_4_**–**15** showed a faster rate for the proteo‐substrate, and a significant kinetic isotope effect (*k_H_*/*k_D_*=2.26, Scheme 6 b). These kinetic and deuteration studies all indicate cyclopalladation as the turnover limiting step in the reaction.

**Figure 2:**
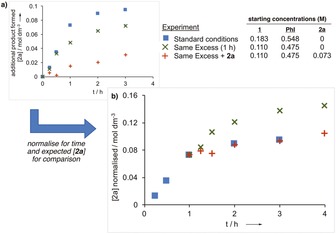
Same excess and product inhibition experiments. Showing a) concentrations of additional product formed; and b) normalized values for direct comparison (+1 h for and expected product concentration for same excess experiments).^15^

Interestingly, the addition of further acetal or Pd at later time points in the reaction did not improve conversion,^22^ posing a question about what determined the final yield obtained. The use of a ‘same excess’ experiment, developed by Blackmond,^16^ provided important insight. This experiment uses reduced concentrations of the reactants to mimic starting the reaction at a later time point (here 40 % conversion; 1 h reaction time), and can highlight catalyst deactivation or product inhibition. On time adjustment of the profiles, the traces did not overlap, indicating a reduction in rate under the standard conditions at later times due to one of these effects (Figure 2).^23^ Adding the expected product concentration from the start of the ‘same excess’ experiments gave a profile that overlaid very well with that using the standard conditions, showing the rate reduction was due to product inhibition.

**Scheme 7:**
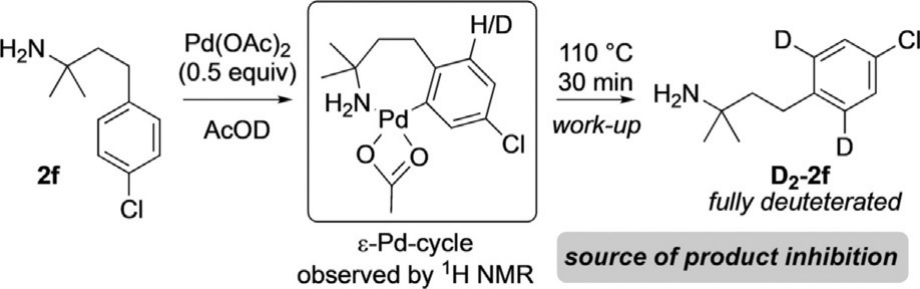
*ϵ*‐Cyclopalladation of product amine **2 f**.

While product inhibition might be expected based on equilibrium considerations, the pronounced effect suggested an additional explanation. Therefore, we examined the interaction of the product with palladium acetate in AcOD‐D_4_, expecting a preferential complexation of the product amine with Pd over the starting material. Mixing amine **2 f** with Pd(OAc)_2_ in a 2:1 ratio at rt gave three amine species, assigned as the protonated amine, a monoamino‐Pd complex, and a palladacycle corresponding to *ϵ*‐cyclometallation (Scheme 7).^15^, ^24^ On heating at the reaction temperature for 30 min, the aromatic signals changed to a 2H singlet corresponding to deuteration of the *ortho*‐positions of the aryl group. This surprisingly facile and reversible cyclopalladation process out‐competes coordination of the substrate; deuteration still readily occurred (40 % in 30 min) in the presence of a 1:1 mixture of **1** and **2 f** with 10 mol % palladium. This process represents the basis of the product inhibition in this reaction, which is also likely to occur in related studies,^10^, ^13^ by accumulating the Pd catalyst within these off‐cycle species.

**Scheme 8:**
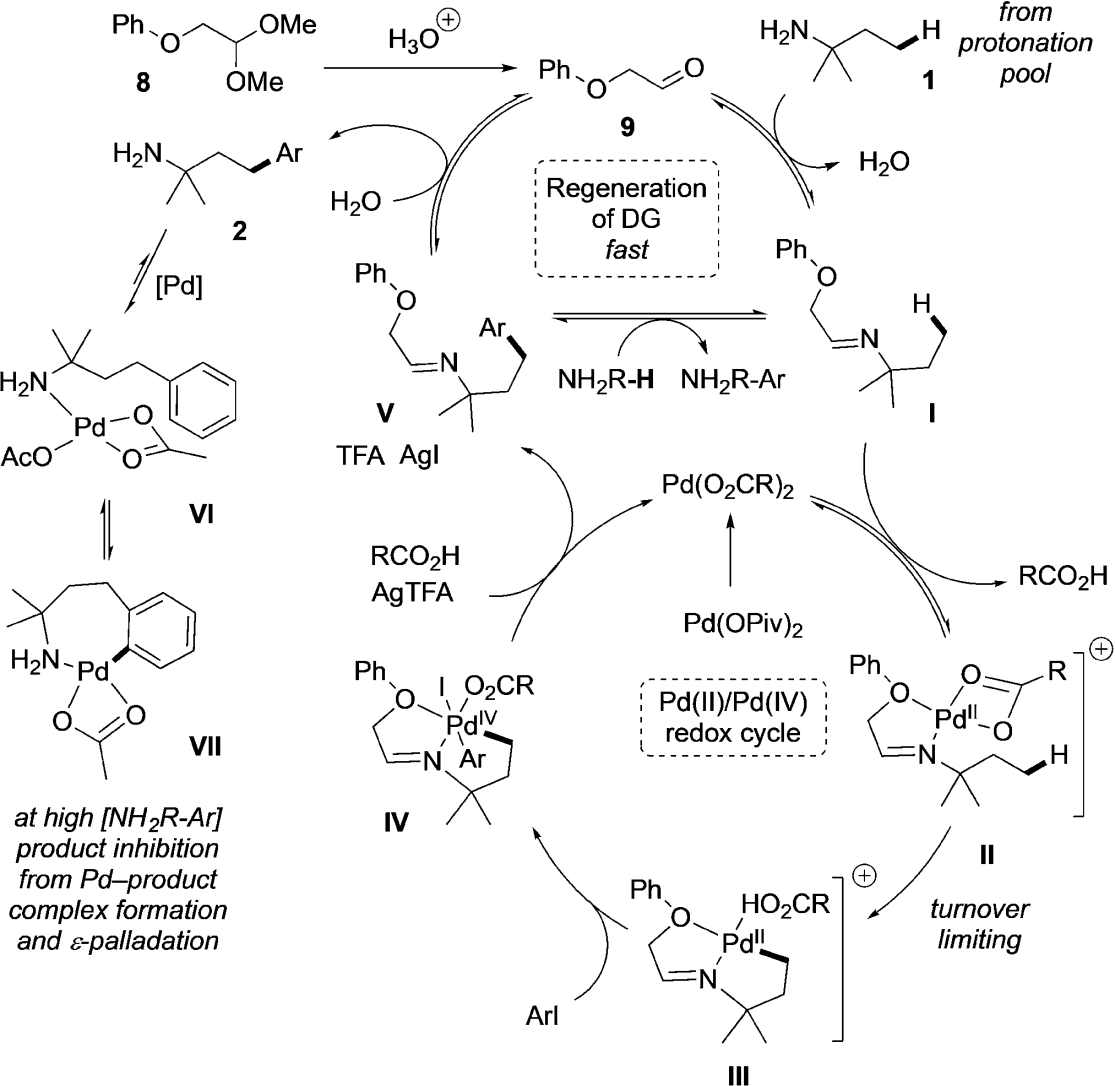
Proposed dual catalytic cycle.

Based on these studies, a proposed mechanism for the dual catalytic system is given in Scheme 8. Under the reaction conditions, acetal **8** is hydrolysed to the corresponding aldehyde **9**. The aldehyde then condenses with the free amine **1**, from a pool of the protonated amine species, to form imine **I**. Complexation to the palladium catalyst with loss of a carboxylate ligand would give a positively charged imine‐Pd species **II** which undergoes turnover‐limiting cyclopalladation to afford the cyclometalated imine intermediate **III**. It is likely that deprotonation or ligand exchange occurs to form a neutral species that then undergoes oxidative addition to Pd^IV^ intermediate **IV**. C−C bond formation by reductive elimination would afford the product imine **V** and regenerate the Pd^II^ catalyst. The transient directing group is turned over by hydrolysis with water, or direct transamination, generating the arylated product **2**. The product amine inhibits the reaction by competitive coordination to the catalyst and facile *ϵ*‐cyclometallation, removing palladium from the productive cycle. The observed reaction profile can be rationalized by the following assumptions. In the early stages of the reaction, when the product concentration is low, little to no Pd‐product amine complexation occurs, and so [cat]_t_=[cat]_0_ and the increase in product concentration is linear with zero order kinetics. However, at a critical higher product concentration (at approx. 40 % conversion), inactive and thermodynamically favourable Pd‐product complexes are formed, removing active catalyst from the reaction and causing a reduction in rate. Eventually, all of the catalyst is trapped in these complexes, causing the yield to plateau. Addition of more catalyst (10 mol %) at *t*=1 h did not lead to increased conversion; presumably also due to rapid formation of the inactive complexes at high product concentration, or due to breakdown of the transient aldehyde species.^15^

## Conclusion

We have described the development and mechanistic investigation of a new transient activator in palladium catalyzed C−H arylation of primary amines. The use of a bench stable alkyl acetal acts through formation of a transient imine directing group, with a clear influence of the (masked) aldehyde and the aryl ether as a second coordinating group. Commercially available (2,2‐dimethoxyethoxy)benzene provides a facile and rapid reaction of branched primary amines with a large range of aryl iodides. Improved yields can be obtained in some cases with (2,2‐dimethoxyethoxy)‐4‐trifluoromethylbenzene as a modified TDG. The C−H activation step was found to be turnover limiting, determined by a zero‐order rate dependence observed in all reactants as well as a pronounced H/D kinetic isotope effect. The reaction was first order in palladium catalyst. Furthermore, we have uncovered the unexpected formation of a 7‐membered palladacycle, activating an *ϵ*‐C(sp^2^)−H bond of the product, thus inhibiting the reaction. We expect these insights will also feed into future design of improved directing groups, and aid the continued rapid progress of the field.

## Experimental Section

### General procedure for the Pd‐catalyzed C−H arylation of amines

AgTFA (132 mg, 0.60 mmol), Pd(OPiv)_2_ (9.3 mg, 0.03 mmol, 10 mol %), aryl iodide (3.00 equiv), H_2_O (27 μL, 1.50 mmol), amine (0.30 mmol), (2,2‐dimethoxyethoxy)benzene **8** (7.6 μL, 0.045 mmol), AcOH (1.25 mL) and HFIP (0.25 mL) were combined in a microwave vial. The vial was sealed under air and the reaction was heated at 110 °C with stirring. After 18 h, the reaction was removed from the heat, allowed to cool to room temperature, diluted with Et_2_O and filtered through a bed of Celite, eluting with Et_2_O (10 mL). The product was extracted from the organic phase into 1 m aqueous HCl solution (3×10 mL). The combined aqueous extracts were basified with saturated aqueous sodium hydroxide solution and the free amine extracted with CH_2_Cl_2_ (3×15 mL). The combined organic extracts were dried (Na_2_SO_4_), and the solvent was removed under reduced pressure to afford the arylated product amine.

## Conflict of interest

The authors declare no conflict of interest.

## Supporting information

As a service to our authors and readers, this journal provides supporting information supplied by the authors. Such materials are peer reviewed and may be re‐organized for online delivery, but are not copy‐edited or typeset. Technical support issues arising from supporting information (other than missing files) should be addressed to the authors.

Supplementary
