# Supplementary material for: Palladium‐Catalyzed C(sp3)−H Arylation of Primary Amines Using a Catalytic Alkyl Acetal to Form a Transient Directing Group
Source: Chemistry. 2018 Nov 8;24(67):17838–43. doi: 10.1002/chem.201804515 (PMC6391947; doi:10.1002/chem.201804515)
Supplement: Supplementary file 1 — Supplementary [file CHEM-24-17838-s001.pdf]

CHEMISTRY
A European Journal

Supporting Information

Palladium-Catalyzed $C(sp^3)$–H Arylation of Primary Amines Using a Catalytic Alkyl Acetal to Form a Transient Directing Group

Sahra St John-Campbell, Alex K. Ou, and James A. Bull*[a]

chem_201804515_sm_miscellaneous_information.pdf

Table of Contents

General Experimental Considerations S03
Optimisation table for C–H arylation of *tert*-amylamine **1** with acetal **8** S04
Control reactions S05
α-Oxidation of less substituted amines S06
Optimisation of reaction conditions for neopentylamine S07
RPKA experiments S08
  General procedure and control reaction S08
  Order in Pd catalyst S09
  Order in acetal S10
  Different excess experiments: reagent orders S11
  Same excess experiments: product inhibition S12
  Effect of aryl iodide: rate and competition experiments S13
  Reaction rate using CF_3-Acetal **12** S14
Deuterium labeling experiments S15
  Reaction in AcOD-D_4 .... S15
  Kinetic isotope effect S16
Reaction of product amine **2f** with $Pd(OAc)_2$ in AcOD-D_4 .... S17
Experimental procedures and characterisation data S20
  Preparation of directing groups (**3-13**) S20
  C(sp^3)–H arylation of *tert*-amylamine with aryl iodides (**2a-s**) S23
  Preparation of amines (**S16**-**S19**, **30**) S29
  C(sp^3)–H arylation of amines (**14-22**) S34
  Preparation of additives for structural comparison of the optimal directing group (**23, 24, 27-29**) S39
References S41
^{1}H and ^{13}C NMR spectra for selected compounds S42

General Experimental Considerations

Amine C–H arylation was carried out under air, all other nonaqueous reactions were run under an inert atmosphere (argon) with flame-dried glassware using standard techniques. Anhydrous solvents were obtained by filtration through drying columns (THF, diethyl ether, CH_2Cl_2). Acetic acid, AgOAc and all palladium catalysts were purchased from Sigma Aldrich and used as provided. Hexafluoroisopropanol was purchased from Fluorochem and used as provided. The quality of the AgTFA significantly impacted yield; 98% from Sigma Aldrich (T62405) was used and stored under argon in the dark. Commercial amines were used as provided or purified by distillation. All other commercial reagents were used as supplied or purified by standard techniques where necessary.

Flash column chromatography was performed using 230-400 mesh silica with the indicated solvent system according to standard techniques. Analytical thin-layer chromatography (TLC) was performed on precoated, glass-backed silica gel plates. Visualisation of the developed chromatogram was performed by UV absorbance (254 nm), aqueous potassium permanganate, *p*-anisaldehyde, phosphomolybdic acid or vanillin stains.

Infrared spectra (ν_{max}, FTIR ATR) were recorded in reciprocal centimeters (cm^{-1}). Nuclear magnetic resonance spectra were recorded on 400 MHz spectrometers. Chemical shifts for ^1H NMR spectra are recorded in parts per million with the solvent resonance as the internal standard (chloroform δ = 7.27 ppm or acetic acid δ = 7.04 ppm). Data is reported as follows: chemical shift [multiplicity (s = singlet, d = doublet, t = triplet, q = quartet, m = multiplet and b = broad), coupling constant in Hz, integration, assignment]. ^{13}C NMR spectra were recorded with complete proton decoupling. Chemical shifts are reported in parts per million with the solvent resonance as the internal standard ($CDCl_3$: δ = 77.00 ppm). ^{19}F NMR spectra were recorded with complete proton decoupling. *J* values are reported in Hz. Assignments of $^1H/^{13}C$ spectra were made by the analysis of δ/J values, and COSY, HSQC and HMBC experiments as appropriate. Melting points are uncorrected.

Note on the use of azides: TMS-N_3 was used for the preparation of amine substrates via azide transfer. Azides are toxic and potentially explosive. No metal was allowed to contact TMS-N_3 or any intermediate. Organic azide intermediates were used immediately and not stored.

Optimisation table for C–H arylation of *tert*-amylamine 1 with acetal 8

The reaction was fully optimised using acetal **8** as the activator, by consideration of silver source, palladium catalyst, solvent, concentration, and additives. Table S1 provides a full summary:

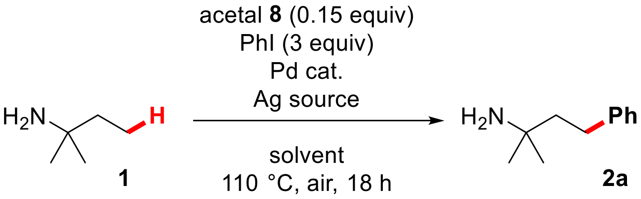

entry	Catalyst (mol%)	Ag salt	Solvent (M)	Additive (equiv)	yield (%)[a]
1	$Pd(OAc)_2$ (5)	AgTFA	AcOH (0.2)	H_2O (5)	46
2	$Pd(OAc)_2$ (5)	AgF	AcOH (0.2)	H_2O (5)	20
3	$Pd(OAc)_2$ (5)	AgOAc	AcOH (0.2)	H_2O (5)	38
4	$Pd(OAc)_2$ (5)	AgTFA	AcOH (0.2)	H_2O (5) + KF (1)	31
5	$Pd(OAc)_2$ (5)	AgTFA	AcOH (0.2)	H_2O (5) + DMSO (1)	46
6	$Pd(OAc)_2$ (5)	AgTFA	AcOH (0.1)	H_2O (5)	40
7	$Pd(OAc)_2$ (5)	AgTFA	AcOH (0.3)	H_2O (5)	45
8	$Pd(OAc)_2$ (10)	AgTFA	AcOH (0.2)	H_2O (5)	52
9	$Pd(OAc)_2$ (10)	AgTFA	AcOH (0.2)	H_2O (2.5)	50
10	$Pd(OAc)_2$ (10)	AgTFA	HFIP:AcOH (1:2, 0.2)	H_2O (5)	55
11	$Pd(OAc)_2$ (10)	AgTFA	AcOH (0.2)	H_2O (5) + MeOH (5)	48
12 (argon)	$Pd(OAc)_2$ (10)	AgTFA	AcOH (0.2)	H_2O (5)	42
13	$Pd(OAc)_2$ (10)	AgTFA	HFIP:AcOH (2:1, 0.2)	H_2O (5)	52
14	$Pd(OAc)_2$ (10)	AgTFA	HFIP:AcOH (5:1, 0.2)	H_2O (5)	49
15	$Pd(OAc)_2$ (10)	AgTFA	HFIP:AcOH (1:5, 0.2)	H_2O (5)	62
16	$Pd(OAc)_2$ (10)	AgTFA	HFIP:AcOH (1:1, 0.2)	H_2O (5)	61
17	$Pd(OAc)_2$ (10)	AgTFA	HFIP (0.2)	H_2O (5)	22
18	$Pd(OAc)_2$ (10)	AgTFA:AgOAc (1:1)	HFIP:AcOH (1:5, 0.2)	H_2O (5)	52
19	$Pd(OAc)_2$ (10)	AgTFA:AgOAc (2:1)	HFIP:AcOH (1:5, 0.2)	H_2O (5)	58
20	$Pd(OAc)_2$ (10)	AgTFA:AgOAc (1:2)	HFIP:AcOH (1:5, 0.2)	H_2O (5)	53
21	$PdCl_2$ (10)	AgTFA	HFIP:AcOH (1:5, 0.2)	H_2O (5)	56
**22**	**$Pd(OPiv)_2$ (10)**	**AgTFA**	**HFIP:AcOH (1:5, 0.2)**	**H_2O (5)**	**64 (59)**
23	$Pd(TFA)_2$ (10)	AgTFA	HFIP:AcOH (1:5, 0.2)	H_2O (5)	51
24	$Pd(OAc)_2$ (10)	AgTFA	HFIP:AcOH (1:5, 0.2)	H_2O (5) + PivOH (0.5)	55
20	$Pd(OAc)_2$ (10)	AgTFA	HFIP:AcOH (1:5, 0.2)	H_2O (5) + MesCOOH (0.5)	55
21	$Pd(OAc)_2$ (10)	AgTFA	HFIP:AcOH (1:5, 0.2)	H_2O (5) + AdCOOH (0.5)	50
22	$Pd(OAc)_2$ (10)	AgTFA	HFIP:AcOH (1:5, 0.2)	H_2O (5) + $Cu(OAc)_2$ (1)	(39)
23	$Pd(OAc)_2$ (10)	AgTFA	HFIP:AcOH (1:5, 0.2)	H_2O (5) + $CuBr_2$ (1)	(16)
24	$Pd(OAc)_2$ (10)	AgTFA	HFIP:AcOH (1:5, 0.2)	H_2O (5) + benzophenone (1)	3
25 (90 °C)	$Pd(OPiv)_2$ (10)	AgTFA	HFIP:AcOH (1:5, 0.2)	H_2O (5)	57
26 (130 °C)	$Pd(OPiv)_2$ (10)	AgTFA	HFIP:AcOH (1:5, 0.2)	H_2O (5)	55

Table S1: Optimisation of direct amine C–H arylation. Conditions: *tert*-amylamine (0.30 mmol), 2-phenoxyacetaldehyde dimethyl acetal **8** (0.15 equiv), iodobenzene (3.0 equiv), Pd catalyst, Ag source (2 equiv), additive, solvent, 110 °C, air, 18 h unless stated otherwise. [a] Yields determined by ^1H NMR using 1,3,5-trimethoxybenzene as an internal standard. Isolated yields in parentheses.

Control reactions

Control reactions under the optimised conditions demonstrated the importance of each component.

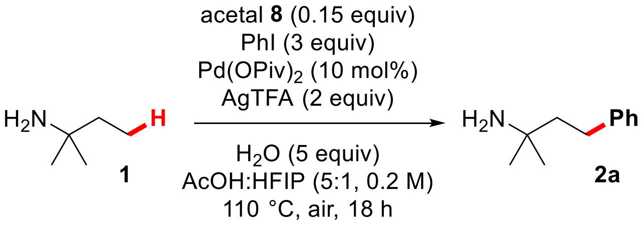

Entry	Change from conditions	Yield 2a (%)
1	none	64
2	no acetal	31
3	no H_2O	47
4	no Pd	0
5	no AgTFA	0
6	AgOAc	21
7	AgOAc + TFA (2 equiv)	21
8	CF_3-acetal **12**	60
9	0.5 equiv **8**	46
10	1.0 equiv **8**	23
11	argon	41
12	$Pd(PPh_3)_4$	32
13	$Pd(dppf)Cl_2$	20
14	no acetal, phenol (0.15 equiv)	30
15	no stirring	54

Table S2: Control reactions. [a]Yields calculated by ^1H NMR using 1,3,5-trimethoxybenzene as an internal standard.

The background reaction in the absence of acetal affords just 31% of **2a** after 18 h. Water is also important in improving the product yield (Entry 3). As expected, the reaction requires the presence of both the silver salt and palladium catalyst to progress. Unlike what we observed from the tertiary aldehyde arylation study,[1] AgTFA in itself is required for good yields and AgOAc with TFA as an additive could not be substituted. Under these conditions, CF_3-acetal **12** gave comparable yields. Higher loadings of acetal **8** were detrimental (Entries 9-10), potentially due to coordination to the catalyst. Interestingly, the reaction was much more efficient under an air atmosphere, with lower yields and much poorer recovery of the starting material observed under an argon atmosphere (Entry 11). Pd^0 catalysts/precatalysts gave lower yields suggesting a Pd^{II}/Pd^{IV} redox system. The breakdown product that can be derived from the acetal additive (phenol) cannot promote the reaction but does not hinder the yield compared to when no acetal is present (entry 14). Running the largely heterogeneous reaction with no stirring was slightly detrimental, but not significantly, and as there is a first order dependence on catalyst concentration, mass transport cannot be rate determining.

α-Oxidation of less substituted amines

α-Oxidation of primary amines to form imines can be promoted by many oxidants.[2] In our case, the process can be promoted by palladium,[3] silver (I) salts (such as recently shown by Sanford)[4] or air.

Under our optimized C–H arylation conditions, when using neopentylamine **S1** as the amine substrate, mono, di and triarylated pivaldehyde products were observed in the ^{1}H NMR spectra of the crude reaction mixture, caused by α-oxidation to the imine under the reaction conditions (Scheme S1).

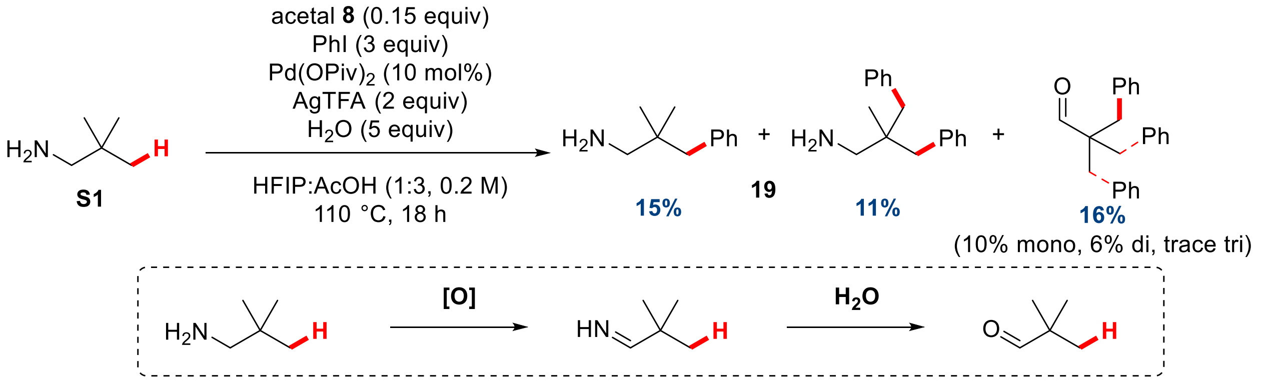

Scheme S1: Oxidation of neopentylamine leading to pivaldehyde products

Optimisation of reaction conditions for neopentylamine S1

Due to the low yield and α-oxidation on susceptible amines, further optimization was conducted using neopentylamine **S1** (table S3). Lowering the reaction temperature and shortening the reaction time to 3 h both gave an improved yield, less α-oxidation and a higher ratio of the monoarylated product

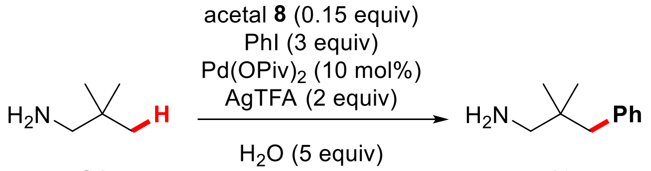

S1 **19**

Entry	Change from conditions	Yield mono (%)	Yield di (%)	Yield total (%)	Pivaldehyde products (%)
1	none	15	11	26	16
2	90 °C	20	8	28	trace
3	CF_3-acetal **12**	15	13	28	16
4	3 h	21	9	30	trace
5	5 equiv PhI	12	17	29	10
6	no acetal **8**	14	4	18	23
**7**	**3 h, 1.5 equiv Ag**	**25**	**10**	**35(30)**	**trace**
8	3 h, 1.5 equiv Ag, Ar	13	6	29	trace
9	3 h, 0.1 M	21	12	33	trace
10	3 h, 1.5 equiv Ag, 0.1 M	21	11	33	trace
11	3 h, 1.2 equiv Ag, 0.1 M	22	10	33	trace

Table S3: Optimisation table for neopentylamine **S1**. [a]Yields calculated by ^{1}H NMR using 1,3,5-trimethoxybenzene as an internal standard. Isolated yield in parentheses.

CF_3-Acetal **12** did not give any significant improvement in yield (Entry 3). Increased equivalents of PhI led to a greater proportion of diarylation with no significant improvement in total yield. The reaction in absence of acetal **8** affords the products in lower yields with increased amounts of α-oxidation (Entry 6) again indicating the importance of this additive. Combining the shorter reaction time and use of a lower loading of AgTFA gave an improved yield of the products (Entry 7, 35% NMR yield). As seen with *tert*-amylamine **1**, running the reaction under argon was detrimental. A lower concentration with various loadings of AgTFA gave similar results (Entries 9–11).

To avoid the α-oxidation, amines bearing a reactive α-hydrogen were arylated under these modified conditions (3 h and 1.5 equiv of AgTFA). However, under either set of conditions, amines with less substitution gave only trace quantities of product, or in the case of propylamine, none of the desired product (Figure S1). This can be attributed to a combination of inherently reduced reactivity (Thorpe Inglod effect), enhanced unproductive bis-amine complex formation and more facile α-oxidation.

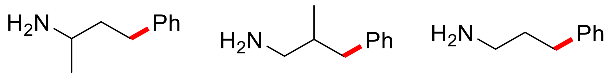

trace **trace** **0%**

Figure S1: Failed amine substrates

RPKA experiments

To investigate the mechanism of the reaction, we used reaction progress kinetic analysis (RPKA). This technique, using the whole reaction profile to analyse the kinetics of a reaction, was developed by Blackmond in 2005.[5] In 2012 RPKA was applied directly to concentration or conversion data.[6] In this study, "same excess" and "different excess" experiments were used to attain qualitative data to aid in understanding the reaction mechanism. More recently Burés applied the use of RPKA in the determination of numerical orders of catalyst[7] and reagents[8] using variable time normalization. These techniques were used in combination here as appropriate to determine reaction order of the reagents, and examine catalyst deactivation.

General procedure for RPKA experiments

Each data point shown represents a discrete, worked up reaction following the procedure outlined below.

AgTFA (66 mg, 0.30 mmol), $Pd(OPiv)_2$ (4.7 mg, 0.015 mmol, 10 mol%), iodobenzene (50.5 µL, 0.45 mmol), H_2O (13.5 µL, 0.75 mmol), *tert*-amylamine **1** (17.5 µL, 0.15 mmol), (2,2-dimethoxyethoxy)benzene **8** (3.8 µL, 0.0225 mmol), AcOH (0.625 mL) and HFIP (0.125 mL) were combined in a microwave vial. The vial was sealed, and the reaction was stirred at 110 °C for 18 h. The reaction was allowed to cool to room temperature, was diluted with CH_2Cl_2 and filtered through a bed of Celite which was then washed with CH_2Cl_2. The solvent was removed under reduced pressure and 10–20 mg of accurately weighed 1,3,5-trimethoxybenzene was added, the residue was fully dissolved in $CDCl_3$ and the yield of the arylated product (**2a**) was calculated by ^{1}H NMR by comparison to the internal standard.

Concentrations were calculated given the total volume of liquids in parent reaction: 0.8218 mL, changes in this value between experiments were deemed negligible.

Control reaction

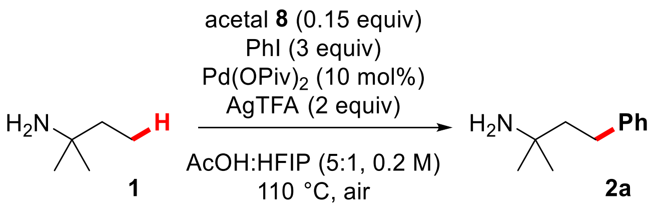

P = arylated product **2a** (blue); RSM = recovered starting material, *tert*-amylamine **1** (orange)

time/h	[RSM]/M	[P]/M
**0.25**	0.146	0.013
**0.5**	0.128	0.035
**1**	0.078	0.073
**2**	0.062	0.089
**3**	0.040	0.095
**6**	0.029	0.104
**12**	0.031	0.091
**18**	0.033	0.100
**24**	0.042	0.093

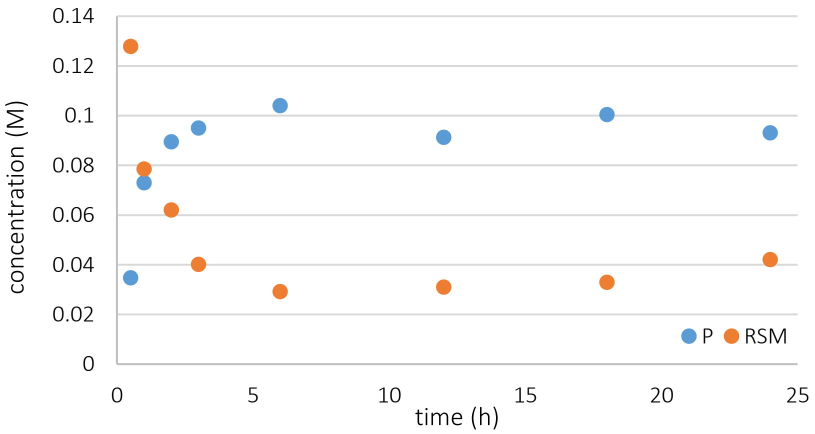

Table S4: Raw data for control reaction. **Figure S2:** Reaction profile for C–H arylation of *tert*-amylamine **1** with acetal **8** (0.15 equiv)

The reaction proceeds to maximum conversion and maximum SM consumption after 3 h, plateauing at later time points. Further RPKA experiments were conducted over 3 h.

Order in Pd catalyst

The graphical method developed by Burés[7] was used to determine the order in the catalyst. A reaction profile was obtained using lower (5, 7.5 mol%) and higher (15 mol%) $Pd(OPiv)_2$ loadings.

	[P] for catalyst loading/M		
**time/h**	**5 mol%**	**7.5 mol%**	**15 mol%**
**0.25**	0.005476	0.010952	0.021903
**0.50**	0.020078	0.021903	0.062059
**1**	0.040156	0.051107	0.091263
**2**	0.071185	0.07301	0.10404
**3**	0.080312	0.083962	0.096739

Table S5: Raw data for different catalyst concentrations.

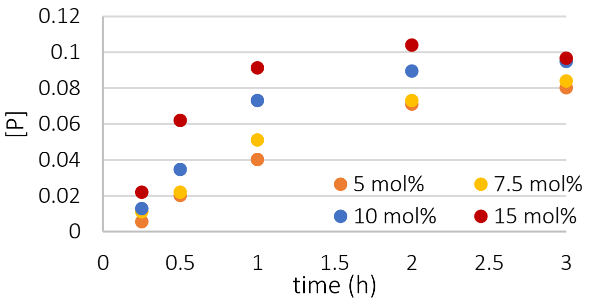

Figure S3: Plot of product formation at different $Pd(OPiv)_2$ loadings

Product formation was slowed at lower catalyst loadings and sped up at the higher loading, ultimately reaching similar conversion. This data could then be used to calculate the order in catalyst by multiplying the time axis by $[cat]^X$ (where X = catalyst order) and observing possible overlay (figure S4).

Normalised time data to determine catalyst order[7]

	$t[cat]^x$											
	for catalyst order = 0.5 ($t[cat]^{0.5}$)				for catalyst order = 1.0 ($t[cat]^1$)				for catalyst order = 2.0 ($t[cat]^2$)			
**time/h**	**5 mol%**	**7.5 mol%**	**10 mol%**	**15 mol%**	**5 mol%**	**7.5 mol%**	**10 mol%**	**15 mol%**	**5 mol%**	**7.5 mol%**	**10 mol%**	**15 mol%**
**0.25**	0.0239	0.0293	0.0338	0.0414	2.28E-03	6.85E-03	4.56E-03	3.42E-03	2.08E-05	4.68E-05	8.33E-05	1.87E-04
**0.50**	0.0478	0.0585	0.0676	0.0827	4.56E-03	1.37E-02	9.13E-03	6.85E-03	4.16E-05	9.37E-05	1.67E-04	3.75E-04
**1**	0.0955	0.1170	0.1351	0.1655	9.13E-03	2.74E-02	1.83E-02	1.37E-02	8.33E-05	1.87E-04	3.33E-04	7.50E-04
**2**	0.1911	0.2340	0.2702	0.3309	1.83E-02	5.48E-02	3.65E-02	2.74E-02	1.67E-04	3.75E-04	6.66E-04	1.50E-03
**3**	0.2866	0.3510	0.4053	0.4964	2.74E-02	8.21E-02	5.48E-02	4.11E-02	2.50E-04	5.62E-04	1.00E-03	2.25E-03

Table S6: Normalised time data different catalyst concentrations at orders of 0.5, 1.0 and 2.

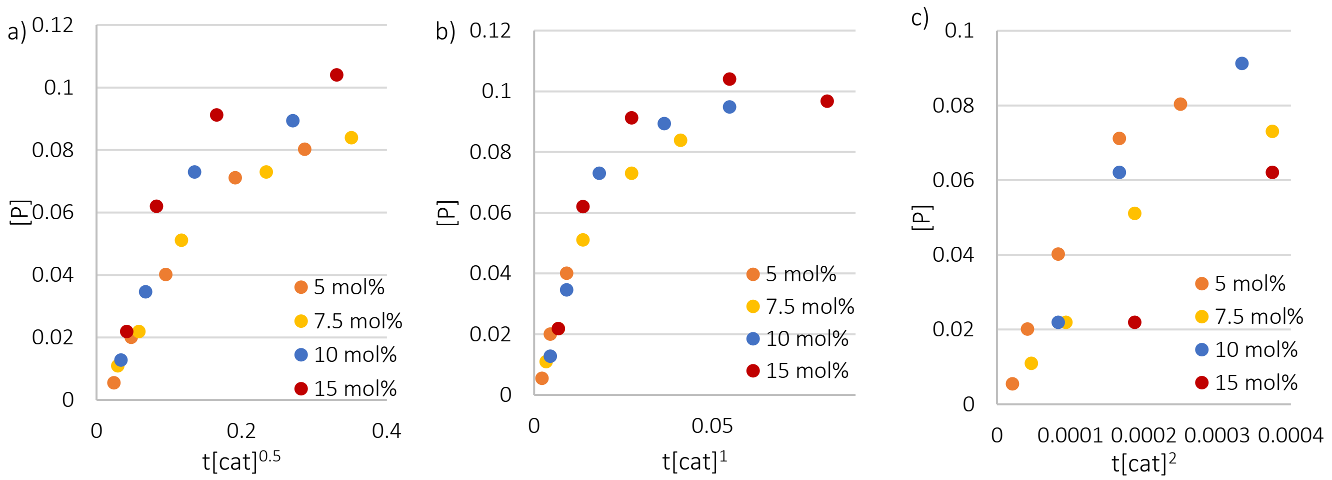

Figure S4: Time normalised plots for catalyst orders of a) 0.5, b) 1 and c) 2, showing the reaction to be first order in catalyst due to overlay

Using this visual method, the reaction is first order in Pd. This value is consistent with accepted catalytic cycles involving a monomeric palladium species.[9]

Order in acetal

Reaction profiles were obtained when altering the loading of acetal **8** (no acetal, 5 mol% and 20 mol%) which were compared to the standard conditions (15 mol%).

	[P] for acetal loading/M		
**time/h**	**none**	**5 mol%**	**25 mol%**
**0.25**		0.013	0.013
**0.50**	0.007	0.040	0.046
**1**	0.022	0.069	0.080
**2**	0.037	0.093	0.099
**3**	0.047	0.102	0.102
**6**	0.060	0.095	0.102

Table S7: Raw data for different acetal loadings.

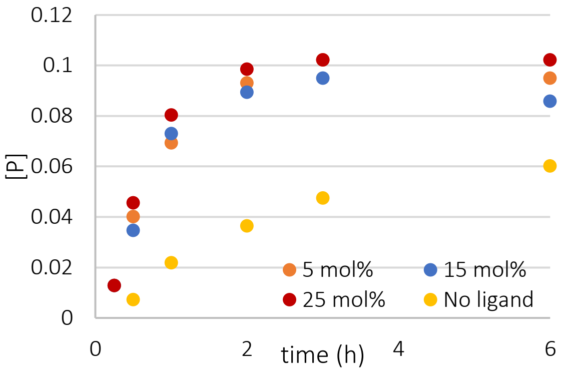

Figure S5: Plot of product formation at different acetal loadings

The reaction rate was independent of acetal concentration, being the same in all cases, indicating a zero order with respect to acetal **8**. This suggests that the rate determining step is not related to turnover of the TDG, and we have reached saturation kinetics of the acetal at loadings as low as 5 mol%. However, when no acetal is present, the rate is distinctly lower; highlighting a likely change in mechanism when there is no potential for imine formation.

Different excess experiments: reagent orders

Different excess experiments were conducted to determine reaction order of the reagents,[6] reducing the amine, aryl iodide and silver trifluoroacetate concentrations (table S8). A third experiment in each series was also conducted using even lower concentrations, to investigate any change in reaction orders with more extreme changes in concentration.

Experiment	Concentration amine 1/M	Concentration PhI/M	Concentration AgTFA/M
**A**	0.183 (1 equiv)	0.548 (3 equiv)	0.365 (2 equiv)
**B**	**0.140** (0.77 equiv)	0.548 (3 equiv)	0.365 (2 equiv)
**B2**	**0.097** (0.53 equiv)	0.548 (3 equiv)	0.365 (2 equiv)
**C**	0.183 (1 equiv)	**0.365** (2 equiv)	0.365 (2 equiv)
**C2**	0.183 (1 equiv)	**0.274** (1.5 equiv)	0.365 (2 equiv)
**D**	0.183 (1 equiv)	0.548 (3 equiv)	**0.274** (1.5 equiv)
**D2**	0.183 (1 equiv)	0.548 (3 equiv)	**0.183** (1 equiv)

Table S8: Starting concentrations (M) for different excess experiments

	[P] for different excess experiments/M					
**time/h**	**B**	**B2**	**C**	**C2**	**D**	**D2**
**0.25**	0.022	0.012	0.018	0.011	0.015	0.011
**0.50**	0.031	0.034	0.031	0.029	0.038	0.033
**0.75**	0.048	0.045	0.055	0.038	0.046	0.049
**1**	0.062	0.054	0.062	0.053	0.058	0.053
**2**	0.083	0.059	0.082	0.077	0.075	0.051
**3**	0.081	0.057	0.091	0.080	0.077	0.053

Table S9: Raw data for different excess experiments

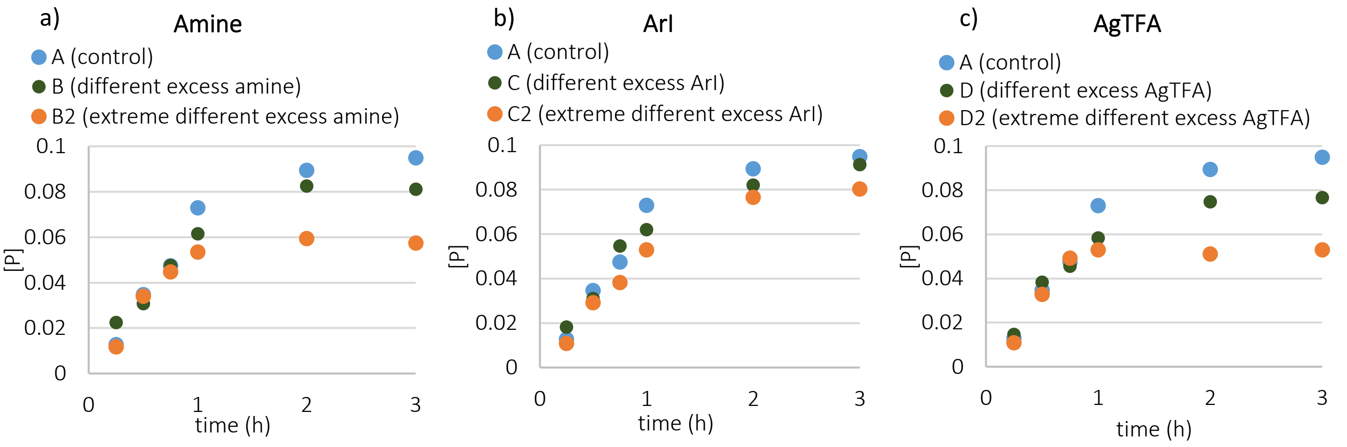

Figure S6: Plot of product formation raw data for same excess experiments a) amine b) PhI c) AgTFA

<u>Amine different excess (Figure S6a):</u> The rate of product formation for experiments A, B and B2, are the same up to the point of where the conversion to product stops at the different amine concentrations, even with a large change in concentration. The rate is independent of the amine SM concentration, hence zero order. We can conclude that formation of possible bis(amine) palladium complexes with the starting material is not hindering the reaction.

<u>Aryl iodide different excess (Figure S6b):</u> the same rate was observed at 2 or 3 equivalents of PhI. At much lower concentrations (C2, 1.5 equiv PhI) there was only a slight drop in rate. The reaction is zero order in PhI under our conditions.

<u>AgTFA different excess (Figure S6c):</u> The increase in product yield between the control and both different excess experiments, occurs at the same rate up to the point of the maximum yield, suggesting a zero order dependence in AgTFA. The final yield is noticeably dependent on the amount of AgTFA; with just 1 equiv AgTFA in experiment D2, the yield dropped significantly.

Same excess experiments

Same excess experiments can determine possible product inhibition or catalyst deactivation.[6] In this study, a time point was selected where the reaction had progressed to 40% product yield, in this case 1 h. The reaction was set up according to the general procedure but using only 60% of the usual amine concentration (10.5 µL, 0.09 mmol, 0.110 M), and less ArI (2.6 equivalents, 44 µL, 0.39 mmol, 0.475 M; assuming only the 0.4 equiv reacted to product). The loadings of $Pd(OPiv)_2$, acetal **8**, water, and AgTFA were kept constant.

To consider product inhibition, a same excess experiment was also conducted with the addition of the product **2a** for each discrete experiment at the concentration expected at the 1 h time point (40%; 9.8 mg, 0.06 mmol, 0.073 M).

	Experiment	Concentration SM/M	Concentration ArI/M	Concentration P added/M
A	Control	0.183	0.548	0
B	Same Excess t = 1 h	**0.110**	**0.475**	0
C	Same Excess t = 1 h + product **xx**	**0.110**	**0.475**	**0.073**

Table S10: Starting concentrations (M) for same excess experiments.

time/h	adjusted time/h	[P]/M Same excess t = 1 h	→	[P]/M Same excess t = 1 h [P] adjusted	[P]/M same excess t = 1 h + product 2a
**0.25**	**1.25**	0.011		0.084	0.078
**0.5**	**1.5**	0.033		0.106	0.075
**1**	**2**	0.048		0.121	0.088
**2**	**3**	0.065		0.138	0.093
**3**	**4**	0.072		0.145	0.104

Table S11: Raw data for same excess experiments, for [P] adjustment, a value of 0.073 M of product **2a** was added to each observed value. Where product was added, 0.073 M of product **2a** was added to each reaction with all other reagents (for each point), which is included in this value.

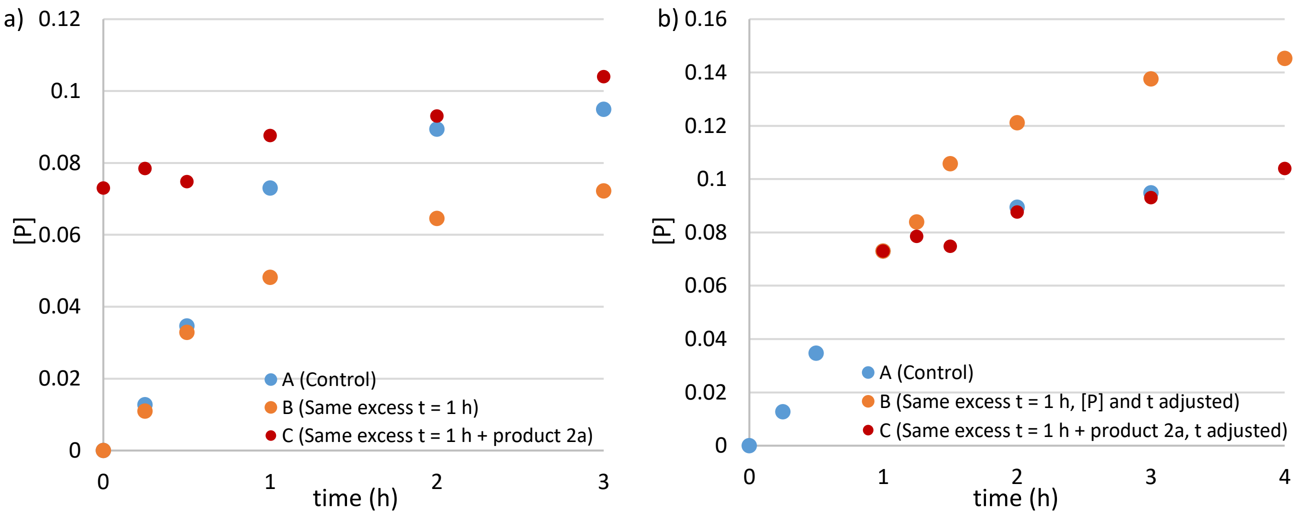

Figure S7: a) plot of observed/raw product concentration for same excess experiments b) Plot using adjusted time and [P]

Figure S7a shows the raw data collected for the same excess experiments and the comparison to the control.

To normalise the data, both same excess experiments were time shifted by 1 h (Figure S7b). As using product concentration, same excess experiment (B) was also adjusted to account for the expected product concentration under standard conditions (+ 0.073M added to each point). The same excess experiment (B, orange curve) shows that the product is formed at a faster rate to the standard control reaction (A, blue) indicating inhibition. When adding product at the start of the reaction (red), the points follow the control curve suggesting product inhibition is a significant factor in the arrested rate, preventing full conversion.

Effect of aryl iodide: Rate and competition experiments

To investigate the relevance of the aryl iodide in the catalytic cycle to support the different excess experiments, profiles of the arylation with different aryl iodides, as well as competition experiments were conducted.
Examples of aryl iodides with different electronics were selected, that gave similar overall yields. Changing the electronics of the aryl iodide had essentially no effect on the rate of the reaction (Table S12, Fig S8). This data also demonstrates zero order kinetics at the start of the reaction.

Time/ h	[P] 4-OMe	[P] 4-Cl
**0.25**	0.011	0.009
**0.5**	0.035	0.031
**0.75**	0.053	0.047
**1**	0.069	0.062
**2**	0.077	0.082

Table S12: Raw data for rate amine arylation with different aryl iodides.

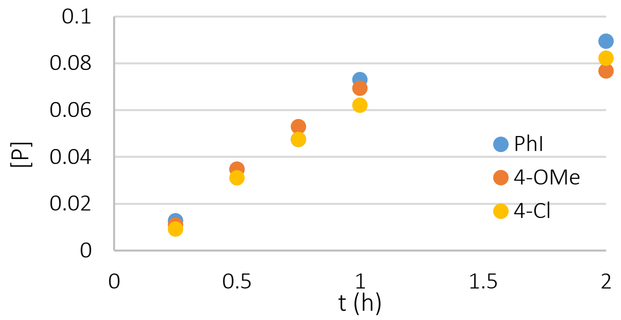

Figure S8: Plot of product formation with different ArI's

General procedure for competition experiments with different aryl iodides

AgTFA (132 mg, 0.60 mmol), $Pd(OPiv)_2$ (9.3 mg, 0.03 mmol, 10 mol%), aryl iodide 1 (0.60 mmol), aryl iodide 2 (0.60 mmol), H_2O (27 µL, 1.50 mmol), *tert*-amylamine **1** (35 µL, 0.30 mmol), (2,2-dimethoxyethoxy)benzene **8** (7.6 µL, 0.045 mmol), AcOH (1.25 mL) and HFIP (0.25 mL) were combined in a microwave vial. The vial was sealed, and the reaction was stirred at 110 °C for 18 h. The reaction was allowed to cool to room temperature, was diluted with CH_2Cl_2 and filtered through a bed of Celite which was then washed with CH_2Cl_2. The solvent was removed under reduced pressure and 10–20 mg of accurately weighed 1,3,5-trimethoxybenzene was added, the residue was fully dissolved in $CDCl_3$ and the yield of the arylated products (**2**) were calculated by ^{1}H NMR in comparison to the internal standard.

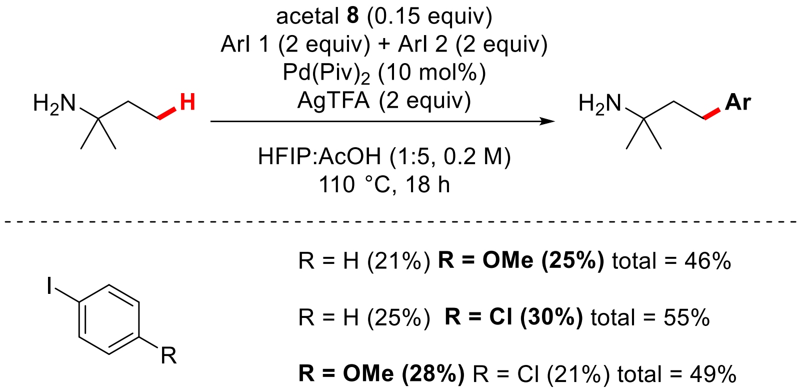

Scheme S2: Competition experiments with various aryl iodides

In competition experiments, only slight preferences were observed for electron rich 4-iodoanisole. These results are consistent with the oxidative addition not being rate determining. The slight preference for the 4-iodoanisole may be due to increased pre-coordination with the more electron rich aromatic and the Pd catalyst.

*Reaction rate using CF_3-acetal **12***

The reaction rates of the two most active acetal directing groups (**8** and **12**) were compared for the arylation of *tert*-amylamine **1**. In both cases the reaction progress was monitored by the formation of the product **2a**. The distribution of acetal or aldehyde DG as well as possible intermediates or breakdown products were also investigated.

time (h)	[P]/M
**0.25**	0.011
**0.5**	0.031
**0.75**	0.053
**1**	0.078
**2**	0.089
**3**	0.111

Table S13: Raw data for rate of CF_3 acetal **12** promoted arylation.

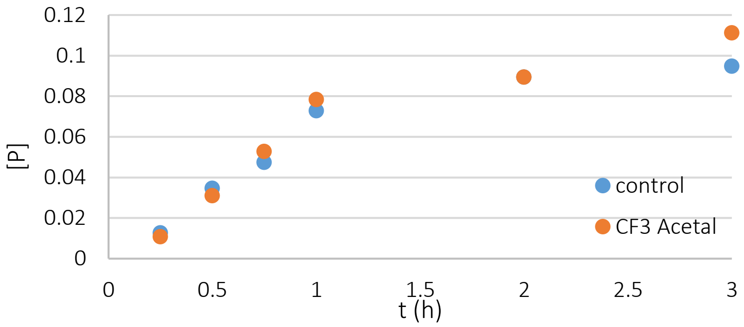

Figure S9: Plot of product formation with different acetals

The rate of the reaction for both acetals was the same at the early stages of the reaction, suggesting a similar effect on the rate determining step. In this reaction, with *tert*-amylamine **1** and iodobenzene, the overall yields were similar. The trifluoromethylated acetal DG often gave improved yields however, suggesting the effect may not be uniform across different substrates.

Fate of the acetal component

Various forms of the acetal were identified at different time points. Possible identities as seen by ^{1}H NMR of crude reaction mixtures are given in figure S10, and quantified in Table S14.

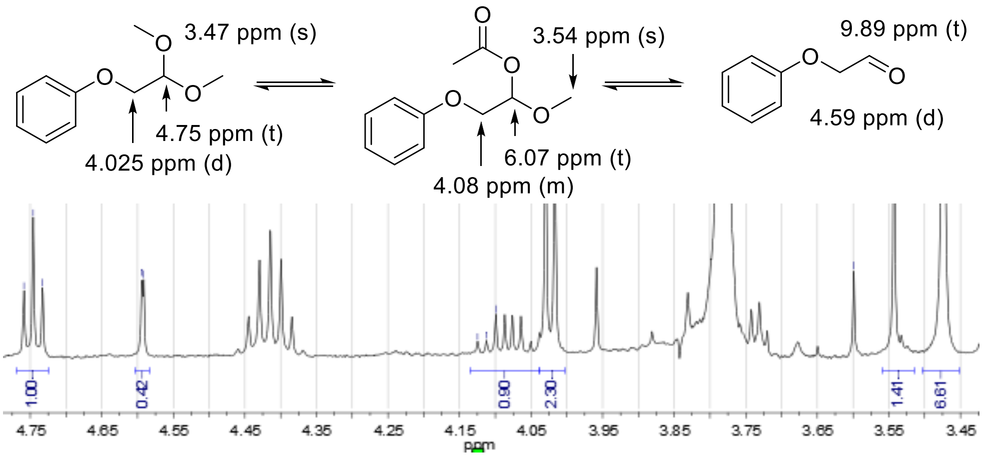

Figure S10: Crude ^{1}H NMR spectra of related acetal signals

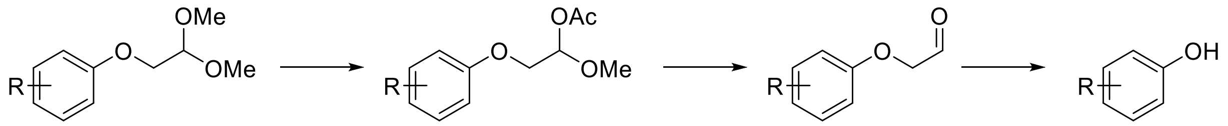

	Distribution of phenoxy acetal 8 (%)					Distribution of CF_3 acetal 12 (%)				
**t (h)**	**acetal**	**aldehyde**	**intermediate**	**phenol**	**total**	**acetal**	**aldehyde**	**intermediate**	**phenol**	**total**
0.25	46	11	18	34	109	57	4	9	3	73
0.5	20	8	2	70	100	48	5	16	17	86
0.75	9	3	3	99	114	25	6	12	45	88
1	0	0	0	90	90	17	9	22	53	101

Table S14: Proportion of acetal breakdown products during the course of the amine arylation reaction.

The acetal can break down to give the corresponding phenol over the course of the reaction (shown to be inactive in promoting C–H arylation). However, the addition of further acetal before or during the reaction course did not improve final yields for *tert*-amylamine, presumably due to the overriding product inhibition. The CF_3-acetal **12** has some improved stability and perhaps substrates that are inherently slower to react benefit from this.

Deuterium labelling experiments

Reaction in AcOD-D₄

Running the reaction in deuterated acetic acid only (CD_3CO_2D, without the usual HFIP co-solvent) resulted in no deuterium incorporation into the benzylic position of the product, suggesting that the C–H activation step is irreversible. This was determined by the relative integrations of the benzylic CH_2 position with the other product signals in the ^1H NMR of the crude reaction mixture, which are the same as for the reaction in the proteo-acetic acid (Figure S11).

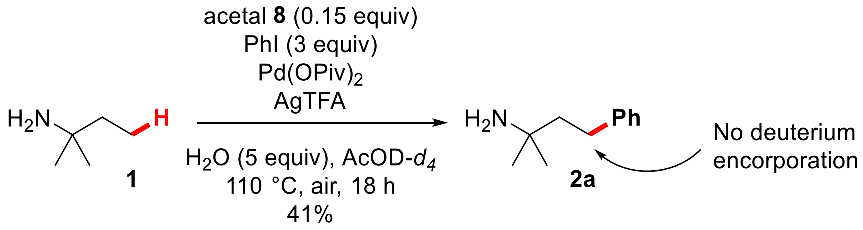

Scheme S3: C–H Arylation in AcOD-D_4

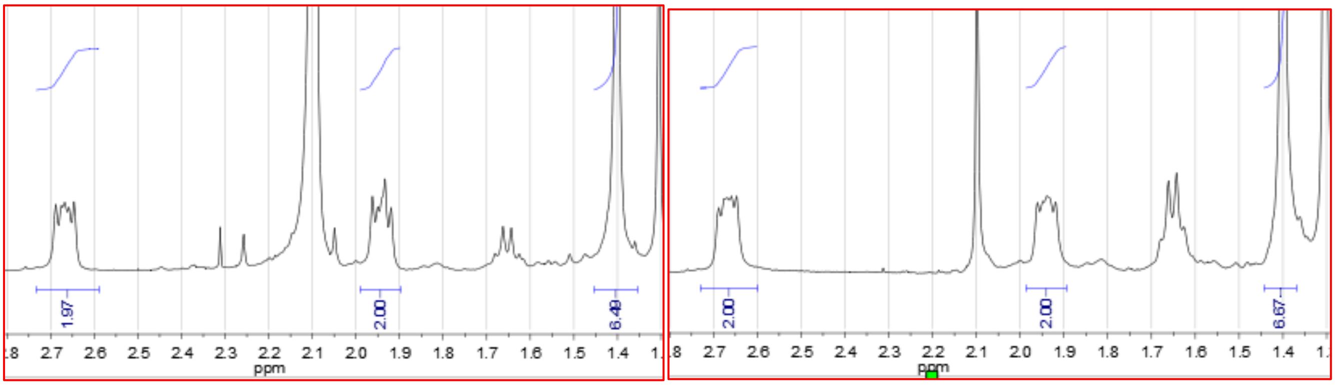

Figure S11: Crude ^1H NMR spectra showing product signals for arylation reaction in CH_3CO_2H (left) and CD_3CO_2D (right)

Kinetic isotope effect

The rate of arylation of deuterated amine **D_5-30** was compared the reaction profile with the parent proteo-amine **30** in different experiments (Scheme S4, figure S12).

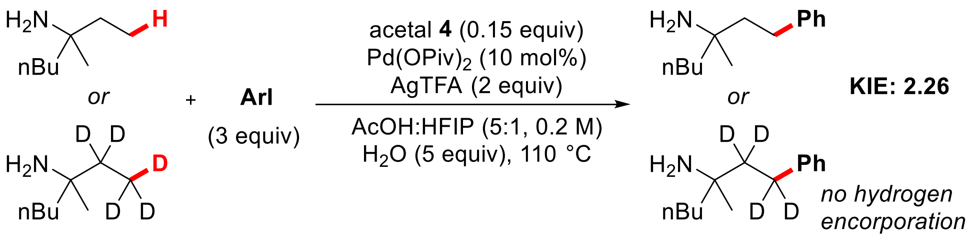

30/D_5-30 **15/D_4-15**

Scheme S4: KIE experiment

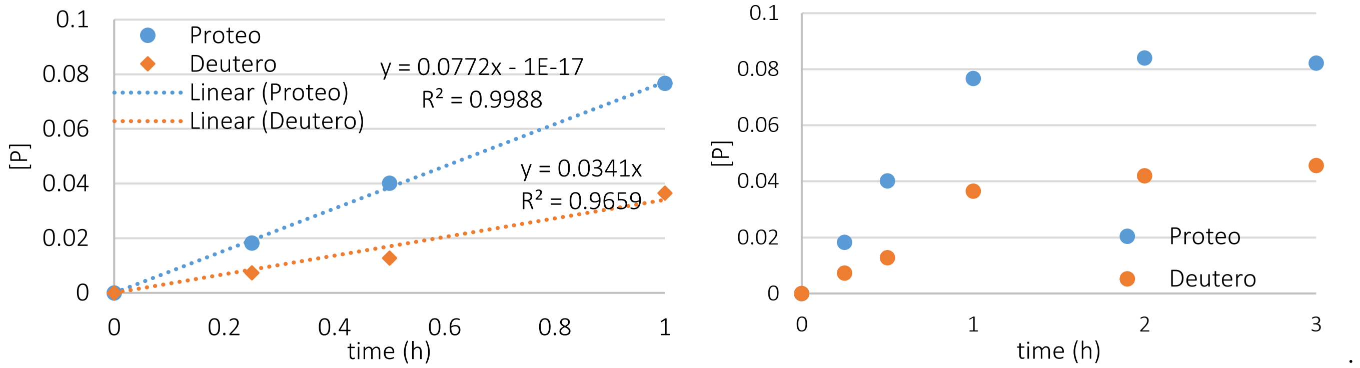

Figure S12: Plots of product formation with proteo and deutero amine substrates

At times points < 1 h the reaction displayed zero order kinetics for both the proteo and deutero substrates. Using a linear trendline gave a rate of 0.0772 M·h^{-1} for the proteo species and 0.0341 M·h^{-1} for the deutero; giving a KIE of 2.26. This suggests a C–H activation is the TOLs. After this time, the reaction rate reduces as the catalyst concentration decreases due to complexation with the product.

Reaction of product amine 2f with Pd(OAc)$_2$ in AcOD-D$_4$

To probe potential complexation between the product amine and palladium, the amine and palladium catalyst were combined in a 2:1 ratio in AcOD-D$_4$ expecting a potential bis(amine)palladium complex to be favoured (Scheme S5). The *p*-chloro phenyl containing product amine **2f** was used to observe any potential change in aromatic signals. Unexpectedly after heating for 30 min, and working up the reaction, the **D2-2f** was isolated, fully deuterated at the *ortho*-position, ε-to the amine.

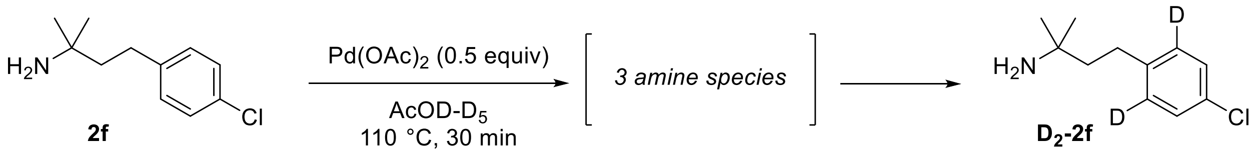

Scheme S5: Interactions of **2f** with Pd(OAc)$_2$

Procedure: Amine **2f** (9.9 mg, 0.05 mmol) was dissolved in AcOD-D$_4$ (0.5 mL) and the solution added to a Young's NMR tube. The tube was sealed under air and an ^{1}H NMR taken at 25 °C. Pd(OAc)$_2$ (5.6 mg, 0.025 mmol) was added and solubilised by warming. The tube was re-sealed and a ^{1}H NMR attained at 25 °C. The tube was then heated in a pre-heated oil bath at 110 °C for 30 minutes. The reaction was allowed to cool to rt and an ^{1}H NMR was taken at 25 °C. The contents of the tube were transferred into a separating funnel using Et$_2$O. The product was extracted using 1 M aqueous HCl, and the combined aqueous extracts basified with saturated aqueous sodium hydroxide. The free amine was then extracted with CH$_2$Cl$_2$. The combined organic extracts were dried (Na$_2$SO$_4$), filtered and solvent removed under reduced pressure to afford deuterated amine **D$_2$-2f** as a yellow solid (3 mg, 30%).

4-(4-Chlorophenyl-2,6-d2)-2-methylbutan-2-amine (D$_2$-2f)

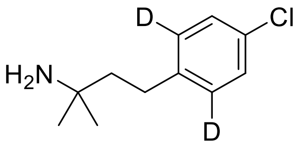

IR (film)/cm^{-1} 2958, 2926, 2861, 1579, 1445, 1366, 1260, 1102, 1018. ^{1}H NMR (400 MHz, CDCl$_3$) δ 7.25 (s, 2 H, Ar-H), 2.66–2.61 (m, 2 H, CH$_2$), 1.67–1.63 (m, 2 H, CH$_2$), 1.50 (bs, 2 H, NH$_2$), 1.17 (s, 6 H, C(CH$_3$)$_2$). ^{13}C NMR (101 MHz, CDCl$_3$) δ 128.4 (2 × Ar-CH), 49.5 (C_q(CH$_3$)$_2$), 46.9 (CH$_2$), 30.5 (C(CH$_3$)$_2$), 30.4 (CH$_2$). Disappearance of the ε-C in the ^{13}C NMR spectra of the deutero compared to the proteo amine (after full characterisation by HSQC and HMBC) enabled conclusive regioselectivity assignment. HRMS (ES) m/z Calcd. for $C_{11}H_{15}N^{35}ClD_2$ [M+H]$^+$: 200.1184; Found: 200.1175.

Figure S13 shows the ^{1}H NMR spectra arising from this study.

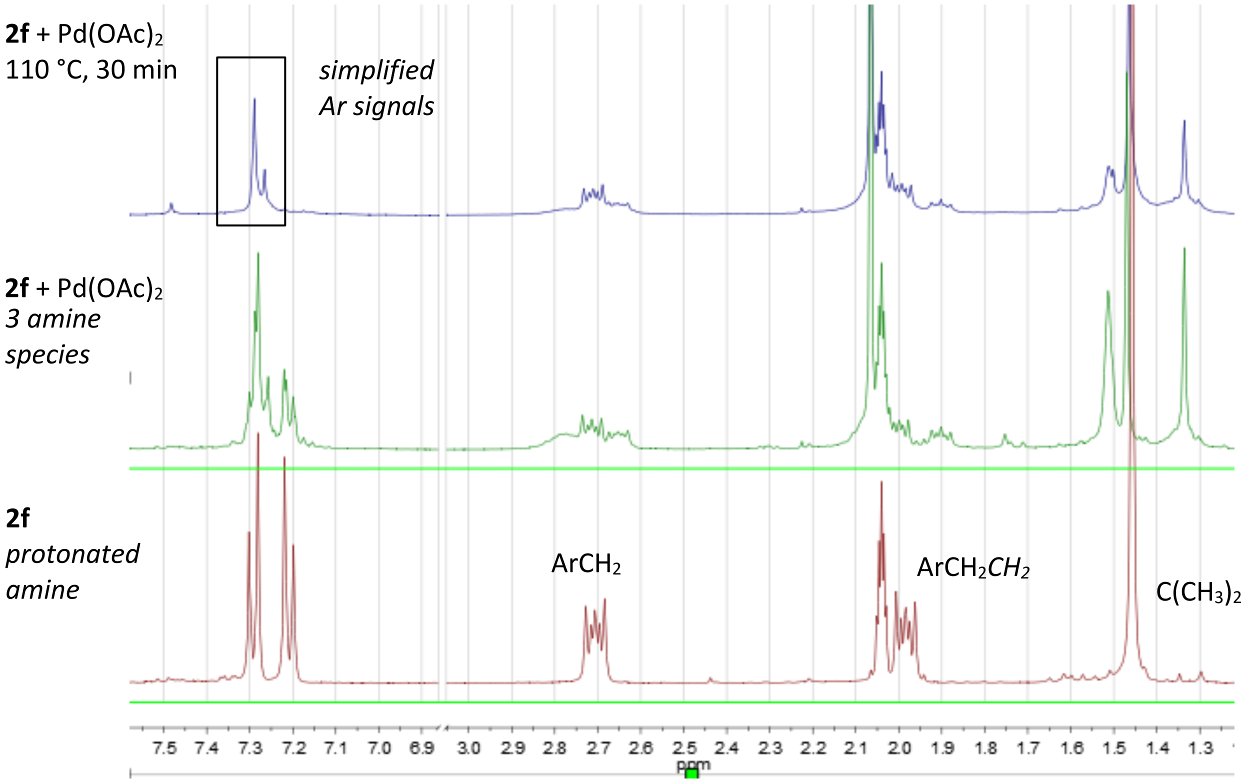

Figure S13: ^{1}H NMR experiments demonstrating product-palladium complex formation. Bottom: Amine **2f** in AcOD-D_4, middle: after addition and solubilization of 0.5 equivalents of $Pd(OAc)_2$, top) after heating to 110 °C for 30 min. Region between 3-7 ppm cut for clarity, no signals were observed in this range.

The spectra of the amine **2f** alone in AcOD-D_4 represents the protonated amine species.

When adding $Pd(OAc)_2$, the benzylic CH_2 signal (2.7 ppm) shows the formation of 2 additional environments and there are corresponding signals for the other CH_2 and *gem*-dimethyl (in a 1:0.8 ratio), suggesting 2 new species.

In the aromatic region, typical *p*-substitution pattern becomes more complex and there is a growing singlet at 7.28 ppm amongst the other aromatic signals. This corresponds to deuteration of the Ar signals.

Heating this mixture gave a similar spectrum though the aromatic signals further simplified.

These observations are due to deuteration of the relevant aromatic C–H bonds by reversible C–H activation through a palladacycle. After an acid/base workup the deuterated product could be cleanly isolated. A control reaction, where the amine product **2f** was heated in AcOD-d_4 in the absence of palladium showed no deuteration occurring. When the reaction was repeated with both amines **1** and **2f** in a 1:1 ratio, deuteration of the product still readily occurred.

From this data, we propose a mixture of protonated amine **2f-H$^+$**, and amine complex **(S2)** and cyclometalated complex **(S3)** (Scheme S6 and Figure S14). The putative assignment of potential species is given in Scheme S6, though more complex structures with bridging acetates cannot be ruled out.

The facile amine directed ε-cyclopalladation is likely to be the main cause of product inhibition.

It is likely that this facile cyclometallation is competitive to our turnover limiting irreversible C(sp^3)–H step at high concentrations of product; with the catalyst resting as these product complexes. In the C–H arylation, there is no evidence at functionalisation at this position of the product, suggesting that despite facile C(sp^2)–H activation, high energy barriers for oxidative addition or reductive elimination are apparent.

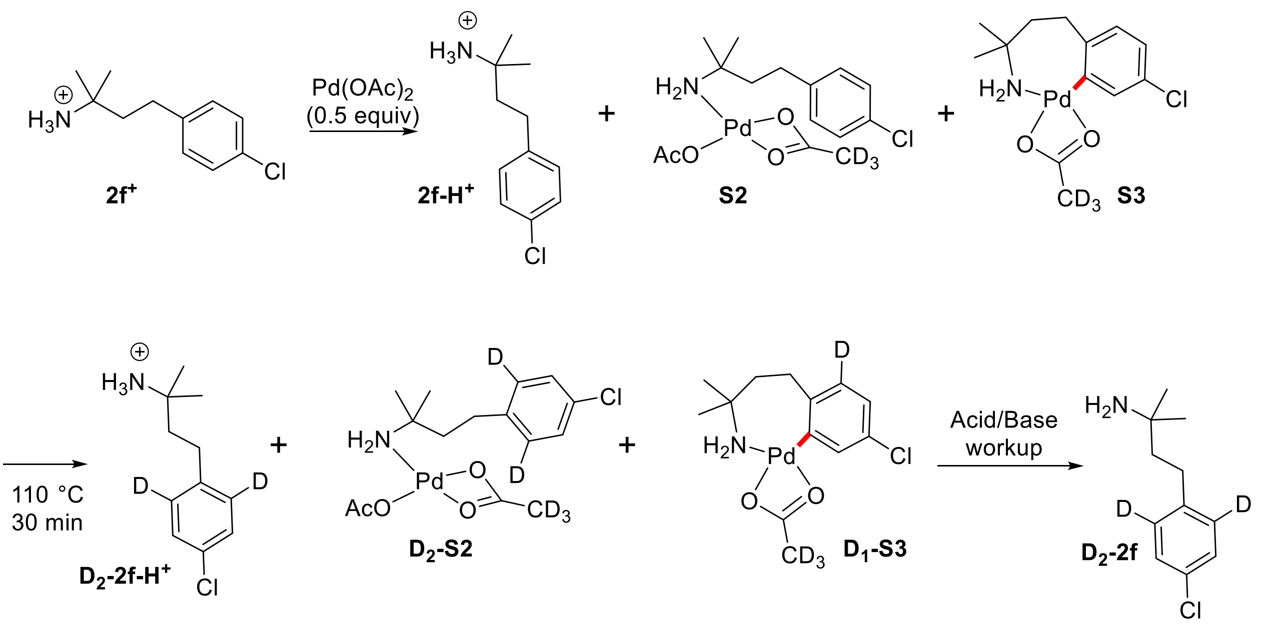

Scheme S6: Product amine-Pd complex formation and deuteration

See figure S14 for fully assigned spectra of the deuterated mixture.

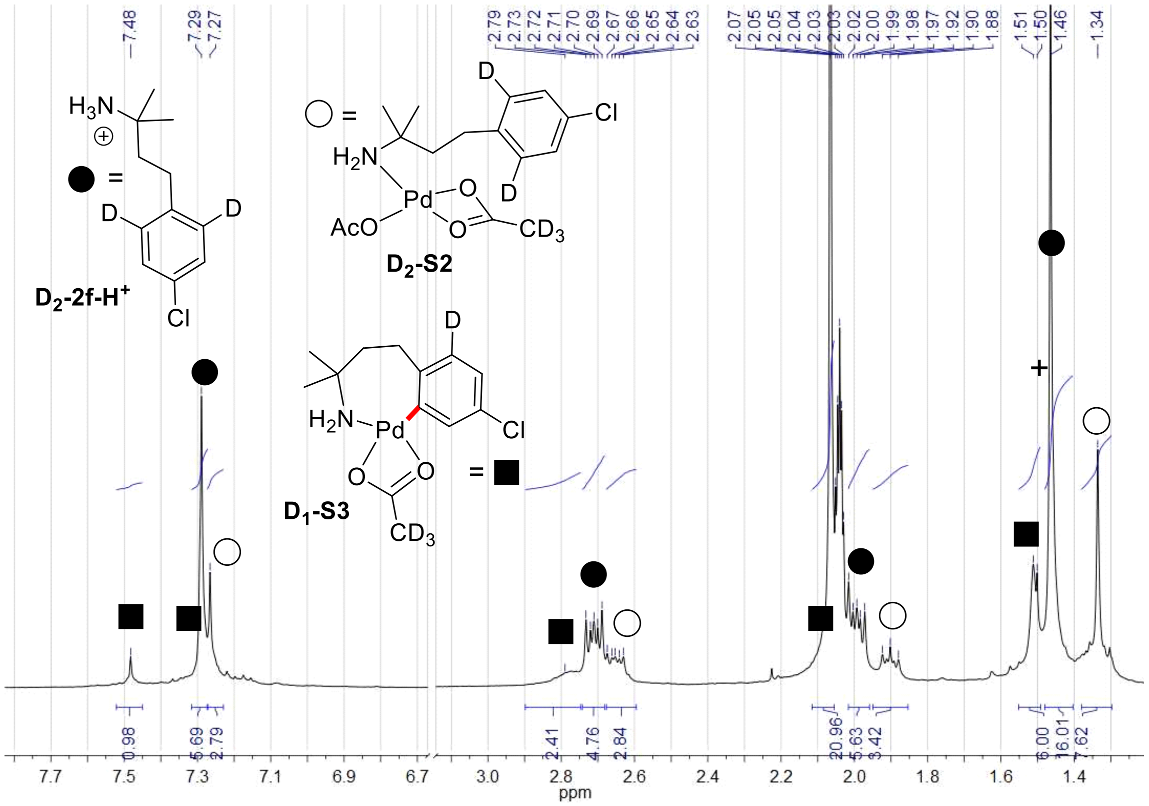

Figure S14: ^{1}H NMR of free and complexed amine species after heating at 110 °C for 30 min

Tentative support for these species was also obtained by mass spectrometry studies on the reaction mixture, from a 1:1 **2f**/$Pd(OAc)_2$ mixture in AcOD, with the following observed masses displaying appropriate Pd isotope patterns:
Protonated amine **2f**$^{+}$: HRMS (TOF-ES^{+}) m/z Calcd. for $C_{11}H_{17}{}^{35}ClN$ [M]$^{+}$: 198.1050; Found: 198.1049.
Complex **D$_2$-S2**: HRMS (TOF-ES^{+}) m/z Calcd. for $C_{13}H_{14}D_5{}^{35}ClNO_2{}^{106}Pd$ [M]$^{+}$: 367.0453; Found: 367.0314.
Complex **D$_1$-S3**: HRMS (TOF-ES^{+}) m/z Calcd. for $C_{13}H_{14}D_4{}^{35}ClNO_2{}^{106}PdNa$ [M+Na]$^{+}$: 388.0204; Found: 388.0563.

Experimental procedures and characterisation data

*Preparation of directing groups (**3, 5, 9-13**)*

Examples **4**, **6**, **7** and **8**, were commercially available.

N-(2,2-dimethoxyethyl)-4-methylbenzenesulfonamide (3)

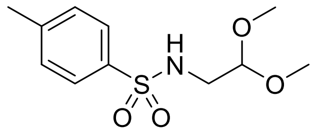

Tosyl chloride (955 mg, 5.00 mmol) was added to a stirred solution of 2,2-dimethoxyethan-1-amine (544 µL, 5.00 mmol) and triethylamine (2.10 mL, 15.0 mmol) in CH_2Cl_2 (1.07 mL). The reaction mixture was diluted with CH_2Cl_2, and the organic phase was washed with water and brine, dried (Na_2SO_4) filtered and solvent removed under reduced pressure to afford acetal **3** as a colourless oil (1.25 g, 100%). R_f 0.33 (40% Et_2O/pentane). IR (film)/cm^{-1} 3287, 3187, 2936, 2838, 1598, 1454, 1437, 1376, 1318, 1306, 1158, 1088, 1080, 1054, 1040. ^{1}H NMR (400 MHz, $CDCl_3$) δ 7.76–7.74 (m, 2 H, Ar-H), 7.33–7.31 (m, 2 H, Ar-H), 4.59 (t, *J* = 5.9 Hz, 1 H, NH), 4.34 (t, J = 5.6 Hz, 1 H, CH), 3.34 (s, 6 H, CH(OC*H*$_3$)$_2$), 3.04 (t, *J* = 5.9 Hz, 2 H, CH_2), 2.44 (s, 3 H, Ar-CH_3). ^{13}C NMR (101 MHz, $CDCl_3$) δ 13C NMR (101 MHz, CDCl3) δ 143.6 (Ar-C_q), 136.7 (Ar-C_q) 129.8 (2 × Ar-C), 127.1 (2 × Ar-C), 102.6 (*C*H(OCH$_3$)$_2$), 54.7 (CH(O*C*H$_3$)$_2$), 44.5 (CH_2), 21.5 (Ar-CH_3). Spectroscopic data for this compound (NMR, IR) is consistent with the literature.[10]

2-(2,2-Dimethoxyethoxy)-1,1,1-trifluoroethane (5)

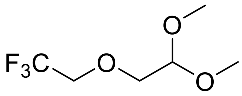

Sodium hydride (48 mg, 1.20 mmol as 60% dispersion in mineral oil) was added to a stirred solution of 2,2,2-trifluoroethan-1-ol (87.0 µL, 1.20 mmol) in DMF (1.0 mL) at 0 °C and the reaction was allowed to warm to rt and stirred for 5 minutes. 2-Bromo-1,1-dimethoxyethane (118 µL, 1.0 mmol) was added and the reaction was stirred at 70 °C overnight. Water was added and the product extracted with Et_2O. The combined organic extracts were washed with brine, dried ($MgSO_4$), filtered and the solvent removed under reduced pressure. Purification by flash column chromatography (SiO_2, 10% Et_2O/Pentane) afforded acetal **5** as a colourless oil (36 mg, 19%). R_f 0.11 (10% Et_2O/pentane). IR (film)/cm^{-1} 2942, 1738, 1277, 1154, 1122, 1068, 964. ^{1}H NMR (400 MHz, $CDCl_3$) δ 4.51 (t, *J* = 5.1 Hz, 1 H, C*H*(OCH$_3$)$_2$), 3.92 (q, *J* = 8.7 Hz, 2 H, CH_2), 3.66 (d, *J* = 5.1 Hz, 2 H, CH_2), 3.42 (s, 6 H, CH(OC*H*$_3$)$_2$). ^{13}C NMR (101 MHz, $CDCl_3$) δ 123.9 (q, $^1J_{C\text{-}F}$ = 280 Hz, CF_3), 102.8 (*C*H(OCH$_3$)$_2$), 68.5 (q, $^2J_{C\text{-}F}$ = 34.1 Hz, *C*H$_2$CF$_3$). 54.3 (CH_2). ^{19}F NMR (377 MHz, $CDCl_3$) δ -74.38. HRMS (APCI) m/z Calcd. for $C_6H_{12}F_3O_3$ $[M+H]^+$: 189.0733; Found: 189.0730.

2-Phenoxyacetaldehyde (9)

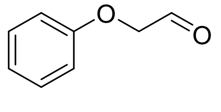

(2,2-Dimethoxyethoxy)benzene **8** was added to a stirred solution of 1 M aqueous HCl (625 µL, 0.63 mmol) and AcOH (1.25 mL, 21.8 mmol) in EtOH (5.0 mL) and the reaction was refluxed for 3 h. The solvent was removed under reduced pressure, saturated aqueous sodium bicarbonate was added and the product extracted with CH_2Cl_2, dried ($MgSO_4$), filtered and solvent removed under reduced pressure. Purification by flash column chromatography (SiO_2, 20% Et_2O/Pentane) afforded aldehyde **9** as a colourless oil (101 mg, 74%). R_f: 0.16 (20% Et_2O/pentane). IR (film)/cm^{-1} 1737 (C=O), 1598, 1494, 1291, 1242, 1048. ^{1}H NMR (400 MHz, $CDCl_3$) δ 9.89 (t, J = 1.0 Hz, 1 H, CHO), 7.36–7.31 (m, 2 H, Ph-H), 7.05–7.02 (m, 1 H, Ph-H), 6.93–6.91 (m, 2 H, Ph-H), 4.59 (d, J = 1.0 Hz, 2 H, CH_2). ^{13}C NMR (101 MHz, $CDCl_3$) δ 199.5 (CHO), 157.6 (Ph-C_q), 129.8 (2 × Ph-C), 122.0 (Ph-C), 114.5 (2 × Ph-C), 72.6 (CH_2). Spectroscopic data for this compound (^{1}H NMR),[11] (^{13}C NMR, IR)[12] is consistent with the literature.

1-(2,2-Dimethoxyethoxy)-4-methoxybenzene (10)

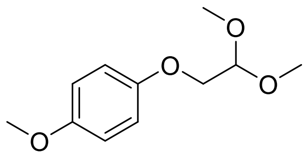

Sodium hydride (48 mg, 1.20 mmol as 60% dispersion in mineral oil) was added to a stirred solution of 4-methoxyphenol (124 mg, 1.00 mmol) in DMF (3.3 mL) at 0 °C and the reaction was allowed to warm to rt and stirred for 5 minutes. 2-Bromo-1,1-dimethoxyethane (142 µL, 1.20 mmol) was added and the reaction was stirred at 120 °C overnight. Water was added and the product extracted with Et_2O. The combined organic extracts were washed with brine, dried ($MgSO_4$), filtered and the solvent removed under reduced pressure. Purification by flash column chromatography (SiO_2, 10% Et_2O/Pentane) afforded acetal **10** as a colourless oil (154 mg, 73%). R_f 0.30 (20% Et_2O/pentane). IR (film)/cm^{-1} 2935, 2834, 1506, 1460, 1443, 1229, 1133, 1034, 1073. ^1H NMR (400 MHz, $CDCl_3$) δ 6.89–6.82 (m, 4 H, Ar-H), 4.71 (t, J = 5.2 Hz, 1 H, CH), 3.97 (d, J = 5.2 Hz, 2 H, CH_2), 3.77 (s, 3 H, OCH_3), 3.46 (s, 6 H, $CH(OC\mathit{H}_3)_2$). ^{13}C NMR (101 MHz, $CDCl_3$) δ 154.1 (Ar-C_q), 152.7 (Ar-C_q), 115.7 (2 × Ar-C), 114.6 (2 × Ar-C), 102.2 ($\mathit{C}H(OCH_3)_2$), 68.3 (OCH_2), 55.7 (Ar-OCH_3), 54.0 (2 × OCH_3). HRMS (APCI) m/z Calcd. for $C_{11}H_{17}O_4$ $[M+H]^+$: 213.1121; Found: 213.1119.

2-(2,2-Dimethoxyethoxy)-1,3-dimethylbenzene (11)

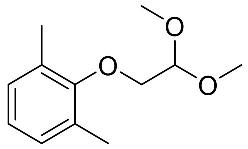

Sodium hydride (48 mg, 1.20 mmol as 60% dispersion in mineral oil) was added to a stirred solution of 2,6-dimethylphenol (122 mg, 1.00 mmol) in DMF (3.3 mL) at 0 °C and the reaction was allowed to warm to rt and stirred for 5 minutes. 2-Bromo-1,1-dimethoxyethane (142 µL, 1.2 mmol) was added and the reaction was stirred at 120 °C overnight. Water was added and the product extracted with Et_2O. The combined organic extracts were washed with brine, dried ($MgSO_4$), filtered and the solvent removed under reduced pressure. Purification by flash column chromatography (SiO_2, 5% Et_2O/Pentane) afforded acetal **11** as a colourless oil (140 mg, 62%). R_f 0.26 (10% Et_2O/pentane). IR (film)/cm^{-1} 2932, 2832, 1476, 1263, 1204, 1133, 1093, 1073, 1032. ^1H NMR (400 MHz, $CDCl_3$) δ 7.02–7.00 (m, 2 H, Ar-H), 6.95–6.91 (m, 1 H, Ar-H), 4.76 (t, J = 5.2 Hz, 1 H, CH), 3.85 (d, J = 5.2 Hz, 2 H, CH_2), 3.48 (s, 6 H, $CH(OC\mathit{H}_3)_2$), 2.31 (s, 6 H, 2 × Ar-CH_3). ^{13}C NMR (101 MHz, $CDCl_3$) δ 155.6 (Ar-C_q), 130.7 (2 × Ar-C_q), 128.9 (2 ⍰ Ar-C), 123.9 (Ar-C), 102.8 ($\mathit{C}H(OCH_3)_2$), 71.5 (OCH_2), 54.0 (2 × OCH_3), 16.2 (2 × Ar-CH_3). HRMS (EI^+) m/z Calcd. for $C_{12}H_{18}O_3$ $[M]^+$: 210.1256; Found: 210.1267.

1-(2,2-Dimethoxyethoxy)-4-(trifluoromethyl)benzene (12)

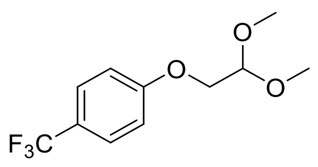

$CsCO_3$ (2.40g, 7.5 mmol), NaI (7.5 mg, 0.05 mmol), 4-trifluoromethylphenol (810 mg, 5.00 mmol), 2-bromo-1,1-dimethoxyethane (1.20 mL, 10.0 mmol) and DMF (1.70 mL) were combined in a flame dried microwave vial, purged with argon and sealed. The reaction was heated to 65 °C overnight, allowed to cool to rt and filtered through a pad of Celite (washed with EtOAc). Water was added and the product extracted with EtOAc. The combined organic extracts were washed with water, brine, dried (Na_2SO_4), filtered and solvent removed under reduced pressure. Purification by flash column chromatography (SiO_2, 10% Et_2O/Pentane) afforded acetal **12** as a colourless oil (451 mg, 36%). R_f 0.20 (10% Et_2O/pentane). IR (film)/cm^{-1} 2942, 1615, 1591, 1519, 1324, 1310, 1259, 1159, 1108, 1067, 1045. ^1H NMR (400 MHz, $CDCl_3$) δ 7.55 (d, J = 8.5 Hz, 2 H, Ar-H), 7.00 (d, J = 8.5 Hz, 2 H, Ar-H), 4.74 (t, J = 5.1 Hz, 1 H, CH), 4.05 (d, J = 5.1 Hz, 2 H, CH_2), 3.48 (s, 6 H, CH(OC*H*$_3$)$_2$). ^{13}C NMR (101 MHz, $CDCl_3$) δ 160.9 (Ar-C_q), 126.9 (q, $^3J_{C\text{-}F}$ = 3.6 Hz, 2 × Ar-C), 124.35 (q, $^1J_{C\text{-}F}$ = 271.1 Hz, CF_3), 123.32 (q, $^2J_{C\text{-}F}$ = 32.8 Hz, Ar-C_q), 114.6 (2 × Ar-C), 102.0 (*C*H(OCH_3)$_2$), 67.8 (OCH_2), 54.3 (2 × OCH_3). ^{19}F NMR (377 MHz, $CDCl_3$) δ -61.58. HRMS (EI^+) m/z Calcd. for $C_{11}H_{13}O_3F_3$ $[M]^+$: 250.0817; Found: 250.0827.

1-(2,2-Dimethoxyethoxy)-3,5-bis(trifluoromethyl)benzene (13)

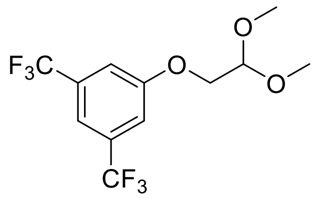

$CsCO_3$ (2.40g, 7.5 mmol), NaI (7.5 mg, 0.05 mmol), 3,5-bis(trifluoromethyl)phenol (761 µL, 5.00 mmol), 2-bromo-1,1-dimethoxyethane (1.20 mL, 10.0 mmol) and DMF (1.70 mL) were combined in a flame dried microwave vial, purged with argon and sealed. The reaction was heated to 65 °C overnight, allowed to cool to room temperature and filtered through a pad of Celite (washed with EtOAc). Water was added and the product extracted with EtOAc. The combined organic extracts were washed with water, brine, dried (Na_2SO_4), filtered and solvent removed under reduced pressure. Purification by flash column chromatography (SiO_2, 5% Et_2O/Pentane) afforded acetal **13** as a colourless oil (322 mg, 20%). R_f 0.32 (10% Et_2O/pentane). IR (film)/cm^{-1} 2942, 2838, 1614, 1467, 1881, 1257, 1171, 1124, 1076, 1045. ^1H NMR (400 MHz, $CDCl_3$) δ 7.49 (s, 1 H, Ar-H), 7.35 (s, 2 H, Ar-H), 4.74 (t, J = 5.1 Hz, 1 H, CH), 4.09 (d, J = 5.1 Hz, 2 H, CH_2), 3.49 (s, 6 H, CH(OC*H*$_3$)$_2$). ^{13}C NMR (101 MHz, $CDCl_3$) δ 159.1 (Ar-C_q), 132.9 (dd, $^2J_{C\text{-}F}$ = 33.3 Hz, 2 × Ar-C_q), 123.10 (q, $^1J_{C\text{-}F}$ = 272.6 Hz, 2 × CF_3), 115.0 (q, $^3J_{C\text{-}F}$ = 3.7 Hz 2 × Ar-C), 114.8 (q, $^3J_{C\text{-}F}$ = 3.7 Hz, Ar-C), 101.9 (*C*H(OCH_3)$_2$), 68.3 (OCH_2), 54.4 (CH(O*C*H$_3$)$_2$). ^{19}F NMR (377 MHz, $CDCl_3$) δ -63.07. HRMS (APCI) m/z Calcd. for $C_{10}H_8F_6O$ $[M-2OCH_3+H]^+$: 259.0552; Found: 259.0563.

C(sp^3)–H Arylation of tert-*amylamine* ***1*** *with aryl iodides* (**2a**-**s**)

General procedure A) AgTFA (132 mg, 0.60 mmol), Pd(OPiv)$_2$ (9.3 mg, 0.03 mmol, 10 mol%), aryl iodide (3.00 equiv), H_2O (27 µL, 1.50 mmol), amine (0.30 mmol), (2,2-dimethoxyethoxy)benzene **8** (7.6 µL, 0.045 mmol), AcOH (1.25 mL) and HFIP (0.25 mL) were combined in a microwave vial. The vial was sealed under air and the reaction was stirred at 110 °C for 18 h. The reaction was allowed to cool to room temperature and filtered through a bed of Celite with Et_2O. The product was extracted from the organic phase into 1 M aqueous HCl solution (3 × 10 mL). The combined aqueous extracts were basified with saturated aqueous sodium hydroxide solution and the free amine extracted with CH_2Cl_2 (3 × 15 mL), dried (Na_2SO_4), and the solvent was removed under reduced pressure to afford the title amines.

2-Methyl-4-phenylbutan-2-amine (2a)

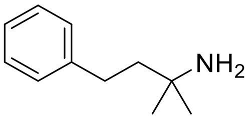

General procedure A was followed using *tert*-amylamine **1** (35 µL, 0.30 mmol) and iodobenzene (101 µL, 0.90 mmol) to afford amine **2a** as a yellow oil (29 mg, 59%). IR (film)/cm^{-1} 3026, 2958, 1502, 1494, 1454, 1392, 1365, 1222, 1177, 1068, 1030. ^{1}H NMR (400 MHz, $CDCl_3$) δ 7.31–7.27 (m, 2 H, Ph-H), 7.21–7.17 (m, 3 H, Ph-H), 2.68–2.64 (m, 2 H, CH_2), 1.71–1.66 (m, 2 H, CH_2), 1.18 (s, 6 H, $C(CH_3)_2$). ^{13}C NMR (101 MHz, $CDCl_3$) δ 142.7 (Ar-C_q), 128.3 (2 × Ar-C), 128.3 (2 × Ar-C), 125.6 (Ar-C), 49.5 (C($CH_3)_2$), 47.0 (CH_2), 31.1 (CH_2), 30.3 (C(CH_3)$_2$). Spectroscopic data for this compound (^{1}H NMR)[13] (^{13}C NMR, IR)[14] is consistent with the literature.

Using CF_3-acetal **12** (12 mg, 0.045 mmol), amine **2a** was obtained as a yellow oil (30 mg, 61%), data obtained was identical.

Methyl 4-(3-amino-3-methylbutyl)benzoate (2b)

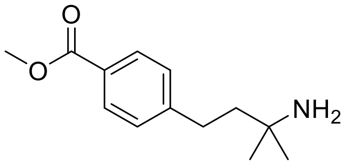

General procedure A was followed using *tert*-amylamine **1** (35 µL, 0.30 mmol) and methyl 4-iodobenzoate (236 mg, 0.90 mmol) to afford amine **2b** as a brown oil (33 mg, 47%). IR (film)/cm^{-1} 2957, 1721 (s, C=O), 1608, 1433, 1271, 1175, 1108, 1015. ^{1}H NMR (400 MHz, CDCl3) δ 7.96 (d, *J* = 8.3 Hz, 2 H, Ar-H), 7.27 (d, *J* = 8.3 Hz, 2 H, Ar-H), 3.91 (s, 3 H, OCH_3), 2.74–2.70 (m, 2 H, CH_2), 1.71–1.66 (m, 2 H, CH_2), 1.51 (bs, 2 H, NH_2), 1.19 (s, 6 H, $C(CH_3)_2$). ^{13}C NMR (101 MHz, $CDCl_3$) δ 169.4 (C=O), 148.4 (Ar-C_q), 129.7 (2 × Ar-C), 128.3 (2 × Ar-C), 127.7 (Ar-C_q), 52.0 (OCH_3), 49.5 (C($CH_3)_2$), 46.6 (CH_2), 31.2 (CH_2), 30.4 (C(CH_3)$_2$). Spectroscopic data for this compound (NMR, IR) is consistent with the literature.[15]

Using CF_3-acetal **12** (12 mg, 0.045 mmol), amine **2b** was obtained as a brown oil (30 mg, 45%), data obtained was identical.

2-Methyl-4-(4-(trifluoromethyl)phenyl)butan-2-amine (2c)

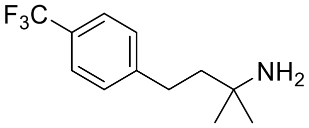

General procedure A was followed using *tert*-amylamine **1** (35 µL, 0.30 mmol) and 1-iodo-4-(trifluoromethyl)benzene (132 µL, 0.90 mmol) to afford amine **2c** as a yellow oil (22 mg, 32%). ^{1}H NMR (400 MHz, $CDCl_3$) δ 7.54 (d, J = 8.0 Hz, 2 H, Ar-H), 7.31 (d, J = 8.0 Hz, 2 H, Ar-H), 2.75–2.70 (m, 2 H, CH_2), 1.70–1.66 (m, 2 H, CH_2), 1.42 (bs, 2 H, NH_2), 1.19 (s, 6 H, $C(CH_3)_2$). ^{13}C NMR (101 MHz, $CDCl_3$) δ 146.9 (Ar-C_q), 128.6 (2 × Ar-C), 128.1 (q, $^2J_{C-F}$ = 32.3 Hz, Ar-C_q), 125.3 (*q*, $^3J_{C-F}$ = 3.4 Hz, 2 × Ar-C), 124.3 (q, $^1J_{C-F}$ = 271.6 Hz, CF_3) 49.5 (C($CH_3)_2$), 46.6 (CH_2), 31.0 (CH_2), 30.4 (C(CH_3)$_2$). ^{19}F NMR (377 MHz, $CDCl_3$) δ -62.29. Spectroscopic data for this compound (NMR, IR) is consistent with the literature.[15]

Using CF_3-acetal **12** (12 mg, 0.045 mmol), amine **2c** was obtained as a yellow oil (28 mg, 40%), data obtained was identical.

2-Methyl-4-(4-nitrophenyl)butan-2-amine (2d)

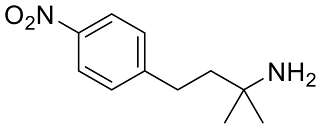

General procedure A was followed using *tert*-amylamine **1** (35 µL, 0.30 mmol) and 1-iodo-4-nitrobenzene (224 mg, 0.90 mmol) to afford amine **2d** as a brown solid (30 mg, 48%). IR (film)/cm^{-1} 2949, 1738, 1596, 1508, 1342, 1217, 1109. ^{1}H NMR (400 MHz, $CDCl_3$) δ 8.15 (d, *J* = 8.7 Hz, 2 H, Ar-H), 7.36 (d, *J* = 8.7 Hz, 2 H, Ar-H), 2.80–2.76 (m, 2 H, CH_2), 1.71–1.67 (m, 2 H, CH_2), 1.48 (bs, 2 H, NH_2), 1.20 (s, 6 H, $C(CH_3)_2$). ^{13}C NMR (101 MHz, $CDCl_3$) δ 150.8 (Ar-C_q), 146.2 (Ar-C_q), 129.1 (2 × Ar-C), 123.7 (2 × Ar-C), 49.5 (*C*$(CH_3)_2$), 46.3 (CH_2), 31.1 (CH_2), 30.5 (C(*C*$H_3)_2$). Spectroscopic data for this compound (NMR, IR) is consistent with the literature.[15]

4-(4-Fluorophenyl)-2-methylbutan-2-amine (2e)

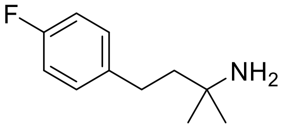

General procedure A was followed using *tert*-amylamine **1** (35 µL, 0.30 mmol) and 1-fluoro-4-iodobenzene (104 µL, 0.90 mmol) to afford amine **2e** as a brown oil (20 mg, 37%). IR (film)/cm^{-1} 2959, 1600, 1508, 1365, 1219, 1156. ^{1}H NMR (400 MHz, $CDCl_3$) δ 7.17–7.13 (m, 2 H, Ar-C), 7.00–6.94 (m, 2 H, Ar-C), 2.66–2.61 (m, 2 H, CH_2), 1.67–1.63 (m, 2 H, CH_2), 1.42 (bs, 2 H, NH_2), 1.17 (s, 6 H, $C(CH_3)_2$). ^{13}C NMR (101 MHz, $CDCl_3$) δ 161.1 (d, $^{1}J_{C\text{-}F}$ = 243.2 Hz, Ar-C_q), 138.3 (d, $^{4}J_{C\text{-}F}$ = 3.1 Hz, Ar-C_q), 129.5 (d, $^{3}J_{C\text{-}F}$ = 7.8 Hz, 2 × Ar-C), 115.0 (d, $^{2}J_{C\text{-}F}$ = 21.1 Hz, 2 × Ar-C), 49.5 (*C*$(CH_3)_2$), 47.1 (CH_2), 30.3 (C(*C*$H_3)_2$), 30.2 (CH_2). ^{19}F NMR (377 MHz, $CDCl_3$) δ -118.0. Spectroscopic data for this compound (NMR, IR) is consistent with the literature.[15]

Using CF_3-acetal **12** (12 mg, 0.045 mmol), amine **2e** was obtained as a brown oil (25 mg, 46%), data obtained was identical.

4-(4-Chlorophenyl)-2-methylbutan-2-amine (2f)

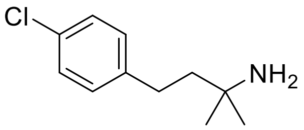

General procedure A was followed using *tert*-amylamine **1** (35 µL, 0.30 mmol) and 1-chloro-4-iodobenzene (214 mg, 0.90 mmol) to afford amine **2f** as a yellow oil (33 mg, 56%). IR (film)/cm^{-1} 2957, 1738, 1490, 1365, 1228, 1217, 1092, 1014. ^{1}H NMR (400 MHz, $CDCl_3$) δ 7.25–7.23 (m, 2 H, Ar-H), 7.13 (d, *J* = 8.4 Hz, 2 H, Ar-H), 2.65–2.61 (m, 2 H, CH_2), 1.67–1.62 (m, 2 H, CH_2), 1.46 (bs, 2 H, NH_2), 1.17 (s, 6 H, $C(CH_3)_2$). ^{13}C NMR (101 MHz, $CDCl_3$) δ 141.2 (Ar-C_q), 131.3 (Ar-C_q), 129.6 (2 × Ar-C), 128.4 (2 × Ar-C), 49.5 (*C*$(CH_3)_2$), 46.9 (CH_2), 30.44 (CH_2), 30.42 (C(*C*$H_3)_2$). Spectroscopic data for this compound (NMR, IR) is consistent with the literature.[15]

On a 2.0 mmol scale, amine **2f** was afforded as a yellow oil (182 mg, 46%).

For **D_2-2f** see page S17.

4-(4-Bromophenyl)-2-methylbutan-2-amine (2g)

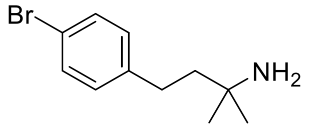

General procedure A was followed using *tert*-amylamine **1** (35 µL, 0.30 mmol) and 1-bromo-4-iodobenzene (256 mg, 0.90 mmol) to afford amine **2g** as a brown oil (41 mg, 56%). IR (film)/cm^{-1} 2957, 1487, 1364, 1070, 1010. ^{1}H NMR (400 MHz, $CDCl_3$) δ 7.41–7.38 (m, 2 H, Ar-H), 7.09–7.07 (m, 2 H, Ar-H), 2.62–2.59 (m, 2 H, CH_2), 1.66–1.62 (m, 2 H, CH_2), 1.43 (bs, 2 H, NH_2), 1.17 (s, 6 H, $C(CH_3)_2$). ^{13}C NMR (101 MHz, $CDCl_3$) δ 141.7 (Ar-C_q), 131.4 (2 × Ar-C), 130.1 (2 × Ar-C), 119.3 (Ar-C_q), 49.5 ($\mathit{C}(CH_3)_2$), 46.8 (CH_2), 30.5 (CH_2), 30.4 ($C(\mathit{C}H_3)_2$). Spectroscopic data for this compound (NMR, IR) is consistent with the literature.[15]

4-(4-Iodophenyl)-2-methylbutan-2-amine (2h)

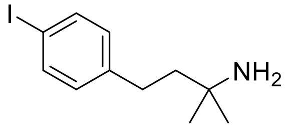

General procedure A was followed using *tert*-amylamine **1** (35 µL, 0.30 mmol) and 1,4-diiodobenzene (297 mg, 0.90 mmol) to afford amine **2h** as a brown oil (32 mg, 37%) inclusive of 4% dimer product. IR (film)/cm^{-1} 2940, 1708, 1486, 1398, 1381, 1284, 1244, 1204, 1181, 1151, 1007. ^{1}H NMR (400 MHz, $CDCl_3$) δ 7.61–7.58 (m, 2 H, Ar-H), 6.97–6.95 (m, 2 H, Ar-H), 2.63–2.58 (m, 2 H, CH_2), 1.66–1.62 (m, 2 H, CH_2), 1.51 (bs, 2 H, NH_2), 1.17 (s, 6 H, $C(CH_3)_2$). ^{13}C NMR (101 MHz, $CDCl_3$) δ 142.4 (Ar-C_q), 137.4 (Ar-C), 130.4 (Ar-C), 128.2 (Ar-C_q), 49.5 ($\mathit{C}(CH_3)_2$), 46.7 (CH_2), 30.6 (CH_2), 30.3 ($C(\mathit{C}H_3)_2$). HRMS (TOF-ES$^+$) m/z Calcd. for $C_{11}H_{17}NI$ $[M+H]^+$: 290.0406; Found: 290.0410. Spectroscopic data for this compound (NMR, IR) is consistent with the literature.[16]

Using CF_3-acetal **12** (12 mg, 0.045 mmol), amine **2h** was obtained as a brown oil (37 mg, 43%), data obtained was identical, again containing <5% dimer.

4-(4-Methoxyphenyl)-2-methylbutan-2-amine (2i)

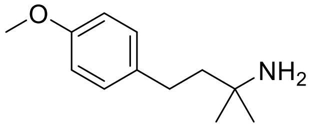

General procedure A was followed using *tert*-amylamine **1** (35 µL, 0.30 mmol) and 4-iodoanisole (211 mg, 0.90 mmol) to afford amine **2i** as a brown oil (32 mg, 55%). IR (film)/cm^{-1} 2955, 1738, 1610, 1510, 1365, 1242, 1176, 1034. ^{1}H NMR (400 MHz, $CDCl_3$) δ 7.12 (d, *J* = 8.6 Hz, 2 H, Ar-H), 6.84 (d, *J* = 8.6 Hz, 2 H, Ar-H), 3.79 (s, 3 H, OCH_3), 2.62–2.58 (m, 2 H, CH_2), 1.67–1.63 (m, 2 H, CH_2), 1.35 (bs, 2 H, NH_2), 1.17 (s, 6 H, $C(CH_3)_2$). ^{13}C NMR (101 MHz, $CDCl_3$) δ 157.6 (Ar-C_q), 134.8 (Ar-C_q), 129.1 (2 × Ar-C), 113.8 (2 × Ar-C), 55.2 (OCH_3), 49.5 ($\mathit{C}(CH_3)_2$), 47.3 (CH_2), 30.4 ($C(\mathit{C}H_3)_2$), 30.1 (CH_2). Spectroscopic data for this compound (NMR, IR) is consistent with the literature.[15]

2-Methyl-4-(m-tolyl)butan-2-amine (2j)

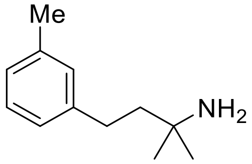

General procedure A was followed using *tert*-amylamine **1** (35 µL, 0.30 mmol) and 1-iodo-3-methylbenzene (156 µL, 0.90 mmol) to afford amine **2j** as a brown oil (30 mg, 56%). IR (film)/cm^{-1} 2957, 2864, 1670, 1608, 1588, 1456, 1380, 1364, 1218. ^{1}H NMR (400 MHz, $CDCl_3$) δ 7.18 (t, J = 7.5 Hz, 1 H, Ar-H), 7.03–7.00 (m, 3 H, Ar-H), 2.65–2.60 (m, 2 H, CH_2), 2.34 (s, 3 H, Ar-CH_3), 1.70–1.65 (m, 2 H, CH_2), 1.41 (bs, 2 H, NH_2), 1.18 (s, 6 H, $C(CH_3)_2$). ^{13}C NMR (101 MHz, $CDCl_3$) δ 142.7 (Ar-C_q), 137.9 (Ar-C_q), 129.1 (Ar-C), 128.3 (Ar-C), 126.4 (Ar-C), 125.3 (Ar-C), 49.5 ($\mathit{C}(CH_3)_2$), 47.2 (CH_2), 31.0 (CH_2), 30.4 ($C(\mathit{C}H_3)_2$), 21.4 (Ar-CH_3). Spectroscopic data for this compound (NMR, IR) is consistent with the literature.[15]

4-(3-Chlorophenyl)-2-methylbutan-2-amine (2k)

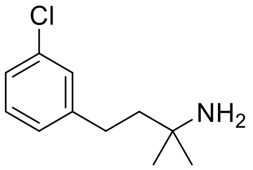

General procedure A was followed using *tert*-amylamine **1** (35 µL, 0.30 mmol) and 1-chloro-3-iodobenzene (111 µL, 0.90 mmol) to afford amine **2k** as a brown oil (34 mg, 56%). IR (film)/cm^{-1} 2958, 2865, 1596, 1573, 1474, 1427, 1365, 1206, 1077. ^{1}H NMR (400 MHz, $CDCl_3$) δ 7.23–7.15 (m, 3 H, Ar-H), 7.09–7.07 (m, 1 H, Ar-H), 2.66–2.62 (m, 2 H, CH_2), 1.68–1.64 (m, 2 H, CH_2), 1.40 (bs, 2 H, NH_2), 1.17 (s, 6 H, $C(CH_3)_2$). ^{13}C NMR (101 MHz, $CDCl_3$) δ 144.9 (Ar-C_q), 134.1 (Ar-C_q), 129.6 (Ar-C), 128.4 (Ar-C), 126.5 (Ar-C), 125.9 (Ar-C), 49.5 ($\mathit{C}(CH_3)_2$), 46.7 (CH_2), 30.8 (CH_2), 30.4 ($C(\mathit{C}H_3)_2$). Spectroscopic data for this compound (NMR, IR) is consistent with the literature.[15]

4-(3-Methoxyphenyl)-2-methylbutan-2-amine (2l)

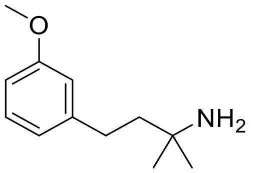

General procedure A was followed using *tert*-amylamine **1** (35 µL, 0.30 mmol) and 3-iodoanisole (107 µL, 0.90 mmol) to afford amine **2l** as a brown oil (27 mg, 47%). IR (film)/cm^{-1} 2958, 1600, 1583, 1488, 1465, 1454, 1434, 1365, 1257, 1151, 1042. ^{1}H NMR (400 MHz, $CDCl_3$) δ 7.21 (t, J = 7.8 Hz, 1 H, Ar-H), 6.81–6.72 (m, 3 H, Ar-H), 3.81 (s, 3 H, OCH_3), 2.66–2.62 (m, 2 H, CH_2), 1.71–1.66 (m, 2 H, CH_2), 1.40 (bs, 2 H, NH_2), 1.18 (s, 6 H, $C(CH_3)_2$). ^{13}C NMR (101 MHz, $CDCl_3$) δ 159.7 (Ar-C_q), 144.5 (Ar-C_q), 129.3 (Ar-C), 120.7 (Ar-C), 114.1 (Ar-C), 111.0 (Ar-C), 55.1 (OCH_3), 49.5 ($\mathit{C}(CH_3)_2$), 46.9 (CH_2), 31.2 (CH_2), 30.4 ($C(\mathit{C}H_3)_2$). HRMS (TOF-ES$^+$) m/z Calcd. for $C_{12}H_{20}NO$ $[M+H]^+$: 194.1545; Found: 194.1543.

Using CF_3-acetal **12** (12 mg, 0.045 mmol), amine **2l** was obtained as a yellow oil (28 mg, 48%), data obtained was identical.

2-Methyl-4-(o-tolyl)butan-2-amine (2m)

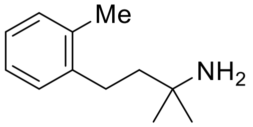

General procedure A was followed using *tert*-amylamine **1** (35 µL, 0.30 mmol) and 1-iodo-2-methylbenzene (115 µL, 0.90 mmol) to afford amine **2m** as a brown oil (7 mg, 13%). IR (film)/cm^{-1} 2961, 2925, 1666, 1605, 1458, 1366, 1259, 1101, 1031. ^{1}H NMR (400 MHz, $CDCl_3$) δ 7.15–7.11 (m, 4 H, Ar-H), 2.66–2.62 (m, 2 H, CH_2), 2.33 (s, 3 H, Ar-CH_3), 1.63–1.59 (m, 4 H, CH_2 + NH_2), 1.20 (s, 6 H, $C(CH_3)_2$). ^{13}C NMR (101 MHz, $CDCl_3$) δ 140.8 (Ar-C_q), 135.7 (Ar-C_q), 130.2 (Ar-C), 128.7 (Ar-C), 126.0 (Ar-C), 125.9 (Ar-C), 49.6 ($\mathit{C}(CH_3)_2$), 45.8 (CH_2), 30.3 ($C(\mathit{C}H_3)_2$), 28.4 (CH_2), 19.2 (Ar-CH_3). HRMS (TOF-ES$^+$) m/z Calcd. for $C_{12}H_{20}N$ $[M+H]^+$: 178.1596; Found: 178.1590.

Using CF_3-acetal **12** (12 mg, 0.045 mmol), amine **2m** was obtained as a brown oil (14 mg, 26%), data obtained was identical.

4-(2-Fluorophenyl)-2-methylbutan-2-amine (2n)

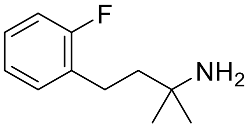

General procedure A was followed using *tert*-amylamine **1** (35 µL, 0.30 mmol) and 1-fluoro-2-iodobenzene (115 µL, 0.90 mmol) to afford amine **2n** as a brown oil (20 mg, 37%). IR (film)/cm^{-1} 2959, 1584, 1491, 1455, 1365, 1227, 1181. ^{1}H NMR (400 MHz, $CDCl_3$) δ 7.22–7.14 (m, 2 H, Ar-H), 7.08–6.98 (m, 2 H, Ar-H), 2.71–2.67 (m, 2 H, CH_2), 1.69–1.64 (m, 2 H, CH_2), 1.47 (bs, 2 H, NH_2), 1.19 (s, 6 H, $C(CH_3)_2$). ^{13}C NMR (101 MHz, $CDCl_3$) δ 161.1 (d, $^{1}J_{C\text{-}F}$ = 244.7 Hz, Ar-C_q), 130.4 (d, $^{3}J_{C\text{-}F}$ = 5.2 Hz, Ar-C), 129.6 (d, $^{2}J_{C\text{-}F}$ = 15.9 Hz, Ar-C_q), 127.4 (d, $^{3}J_{C\text{-}F}$ = 8.2 Hz, Ar-C), 124.0 (d, $^{4}J_{C\text{-}F}$ = 3.4 Hz, Ar-C), 115.2 (d, $^{2}J_{C\text{-}F}$ = 22.3 Hz, Ar-C), 49.6 ($C(CH_3)_2$), 45.5 (CH_2), 30.2 ($C(CH_3)_2$), 24.3 (CH_2). ^{19}F NMR (377 MHz, $CDCl_3$) δ -119.3. HRMS (TOF-ES$^+$) m/z Calcd. for $C_{11}H_{17}NF$ $[M+H]^+$: 182.1345; Found: 182.1352. Spectroscopic data for this compound (NMR, IR) is consistent with the literature.[16]

4-(2-Methoxyphenyl)-2-methylbutan-2-amine (2o)

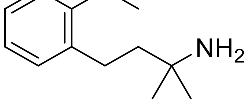

General procedure A was followed using *tert*-amylamine **1** (35 µL, 0.30 mmol) and 2-iodoanisole (105 µL, 0.90 mmol) to afford amine **2o** as a brown oil (22 mg, 38%). IR (film)/cm^{-1} 2957, 1599, 1493, 1463, 1241, 1029. ^{1}H NMR (400 MHz, $CDCl_3$) δ 7.20–7.14 (m, 2 H, Ar-H), 6.91–6.84 (m, 2 H, Ar–H), 3.83 (s, 3 H, OCH_3), 2.67–2.63 (m, 2 H, CH_2), 1.66–1.61 (m, 2 H, CH_2), 1.51 (bs, 2 H, NH_2), 1.18 (s, 6 H, $C(CH_3)_2$). ^{13}C NMR (101 MHz, $CDCl_3$) δ 157.3 (Ar-C_q), 131.1 (Ar-C_q), 129.5 (Ar-C), 126.9 (Ar-C), 120.4 (Ar-C), 110.2 (Ar-C), 55.2 (OCH_3), 49.6 (CH_2), 45.2 ($C(CH_3)_2$), 30.2 ($C(CH_3)_2$), 25.3 (CH_2). HRMS (TOF-ES$^+$) m/z Calcd. for $C_{12}H_{20}NO$ $[M+H]^+$: 194.1545; Found: 194.1552.

Using CF_3-acetal **12** (12 mg, 0.045 mmol), amine **2o** was afforded as a yellow oil (23 mg, 47%), data obtained was identical.

4-(Benzo[d][1,3]dioxol-5-yl)-2-methylbutan-2-amine (2p)

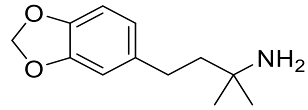

General procedure A was followed using *tert*-amylamine **1** (35 µL, 0.30 mmol) and 5-iodobenzo[d][1,3]dioxole (110 µL, 0.90 mmol) to afford amine **2p** as a brown oil (22 mg, 35%). IR (film)/cm^{-1} 2958, 1502, 1488, 1440, 1364, 1245, 1187, 1037. ^{1}H NMR (400 MHz, $CDCl_3$) δ 6.76 – 6.63 (m, 3 H, Ar-H), 5.92 (s, 2 H, OCH_2), 2.60–2.55 (m, 2 H, CH_2), 1.65–1.61 (m, 2 H, CH_2), 1.43 (bs, 2 H, NH_2), 1.16 (s, 6 H, $C(CH_3)_2$). ^{13}C NMR (101 MHz, $CDCl_3$) δ 147.5 (Ar-C_q), 145.4 (Ar-C_q), 136.6 (Ar-C_q), 120.8 (Ar-C), 108.8 (Ar-C), 108.1 (Ar-C), 100.7 (OCH_2), 49.4 ($C(CH_3)_2$), 47.3 (CH_2), 30.8 (CH_2), 30.4 ($C(CH_3)_2$). HRMS (TOF-ES$^+$) m/z Calcd. for $C_{12}H_{18}NO_2$ $[M+H]^+$: 208.1338; Found: 208.1333.

Using CF_3-acetal **12** (12 mg, 0.045 mmol), amine **2p** was afforded as a brown oil (29 mg, 47%), data obtained was identical.

4-(2,3-Dihydrobenzo[b][1,4]dioxin-6-yl)-2-methylbutan-2-amine (2q)

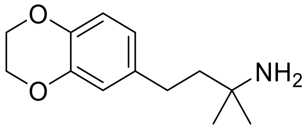

General procedure A was followed using *tert*-amylamine **1** (35 µL, 0.30 mmol) and 6-iodo-2,3-dihydrobenzo[b][1,4]dioxine (336 mg, 0.90 mmol) to afford amine **2q** as a yellow oil (37 mg, 56%). IR (film)/cm^{-1} 2960, 1589, 1507, 1458, 1283, 1257, 1067. ^{1}H NMR (400 MHz, $CDCl_3$) δ 6.78 (d, J = 8.2 Hz, 1 H, Ar-H), 6.71 (d, J = 2.0 Hz, 1 H, Ar-H), 6.67 (dd, J = 8.2, 2.0 Hz, 1 H, Ar-H), 4.24 (s, 4 H, 2 × OCH_2), 2.57–2.53 (m, 2 H, CH_2), 1.66–1.62 (m, 2 H, CH_2), 1.54 (bs, 2 H, NH_2), 1.16 (s, 6 H, $C(CH_3)_2$). ^{13}C NMR (101 MHz, $CDCl_3$) δ 143.3 (Ar-C_q), 141.5 (Ar-C_q), 136.1 (Ar-

C_q), 121.1 (Ar-C), 117.0 (Ar-C), 116.8 (Ar-C), 64.4 (OCH_2), 64.3 (OCH_2), 49.5 ($C(CH_3)_2$), 47.1 (CH_2), 30.32 ($C(CH_3)_2$), 30.27 (CH_2). HRMS (TOF-ES$^+$) m/z Calcd. for $C_{13}H_{20}NO_2$ $[M+H]^+$: 222.1494; Found: 222.1496.

2-Methyl-4-(6-(trifluoromethyl)pyridin-2-yl)butan-2-amine (2r)

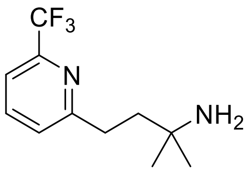

General procedure A was followed using *tert*-amylamine **1** (35 µL, 0.30 mmol) and 2-iodo-6-(trifluoromethyl)pyridine (246 mg, 0.90 mmol) to afford amine **2r** as a brown oil (40 mg, 57%). IR (film)/cm^{-1} 2963, 1601, 1463, 1340, 1182, 1133, 1111, 1090. ^{1}H NMR (400 MHz, $CDCl_3$) δ 7.76 (t, J = 7.8 Hz, 1 H, Ar-H), 7.50 (d, J = 7.6 Hz, 1 H, Ar-H), 7.37 (d, J = 7.9 Hz, 1 H, Ar-H), 2.96–2.91 (m, 2 H, CH_2), 1.84–1.79 (m, 2 H, CH_2), 1.43 (bs, 2 H, NH_2), 1.19 (s, 6 H, $C(CH_3)_2$). ^{13}C NMR (101 MHz, $CDCl_3$) δ 163.5 (Ar-C_q), 147.61 (q, $^2J_{C-F}$ = 34.2 Hz, Ar-C_q), 137.5 (Ar-C), 125.4 (Ar-C), 121.6 (q, $^1J_{C-F}$ = 274.2 Hz, CF_3), 117.6 (q, $^3J_{C-F}$ = 2.7 Hz, Ar-C), 49.5 ($C(CH_3)_2$), 44.4 (CH_2), 33.4 (CH_2), 30.4 ($C(CH_3)_2$). ^{19}F NMR (377 MHz, $CDCl_3$) δ -68.04. Spectroscopic data for this compound (NMR, IR) is consistent with the literature.[15]

4-(3,5-dimethylphenyl)-2-methylbutan-2-amine (2s)

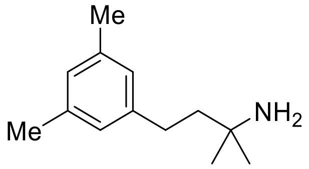

General procedure A was followed using *tert*-amylamine **1** (35 µL, 0.30 mmol) and 1-iodo-3,5-dimethylbenzene (130 µL, 0.90 mmol) to afford amine **2s** as a yellow oil (31 mg, 54%). IR (film)/cm^{-1} 3013, 2956, 2918, 2864, 1605, 1466, 1380, 1364, 1036. ^{1}H NMR (400 MHz, $CDCl_3$) δ 6.83 (s, 3 H, Ar-H), 2.61–2.56 (m, 2 H, CH_2), 2.30 (s, 3 H, Ar-CH_3), 2.30 (s, 3 H, Ar-CH_3), 1.69–1.64 (m, 2 H, CH_2), 1.37 (bs, 2 H, NH_2), 1.17 (s, 6 H, $C(CH_3)_2$). ^{13}C NMR (101 MHz, $CDCl_3$) δ 142.7 (Ar-C_q), 137.8 (2 × Ar-C_q), 127.3 (Ar-C), 126.1 (2 × Ar-C), 49.6 ($C(CH_3)_2$), 47.3 (CH_2), 30.9 (CH_2), 30.4 ($C(CH_3)_2$), 21.2 (2 × Ar-CH_3). HRMS (TOF-ES$^+$) m/z Calcd. for $C_{15}H_{20}N$ $[M+H]^+$: 192.1754; Found: 192.1752.

*Preparation of amines (**S16-S19, 30**)*

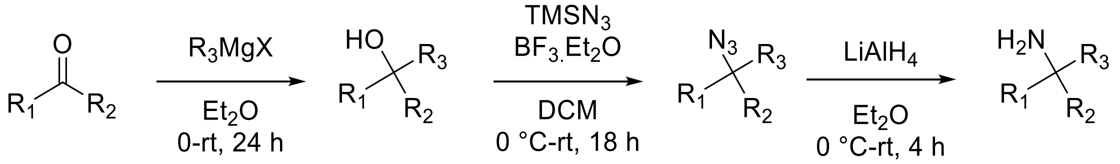

Procedure for the synthesis of amines via azide transfer based on preparation by Shi.[17] All other amines were commercially available.

General procedure B) Grignard reagent (15.0 mmol, 1.5 equiv) was added dropwise to a stirred solution of ketone (10 mmol, 1 equiv) in Et_2O (13.3 mL, 0.75 M) at 0 °C, the reaction was allowed to warm to rt and stirred for 18 h. The reaction was quenched by slow addition of saturated aqueous ammonium chloride solution and stirred for 10 minutes. The reaction was diluted with Et_2O and the organic phase was washed with water and brine, dried (Na_2SO_4), filtered and the solvent removed under reduced pressure to afford the crude alcohol which was used in the next step without further purification.

General procedure C) $TMSN_3$ (1.2 equiv in 2 mL CH_2Cl_2) was added to a stirred solution of alcohol (1 equiv) in CH_2Cl_2 (1.12 M). $BF_3.Et_2O$ (1.2 equiv) was then added dropwise at 0 °C, the reaction was allowed to warm to rt and stirred for 18 h. The reaction was quenched by slow addition of saturated aqueous ammonium bicarbonate solution and stirred for 10 minutes. The reaction was diluted with CH_2Cl_2 and the organic phase was washed with water and brine, dried (Na_2SO_4), filtered and the solvent removed under reduced pressure to afford the crude azide which was used in the next step without further purification.

General procedure D) Lithium aluminium hydride (1 equiv) was slowly added to a stirred solution of azide (1 equiv) in Et_2O (1.25 M) at 0 °C. The reaction was allowed to warm to room temperature and stirred for 4 h. The reaction was quenched by slow addition of $Na_2SO_4{\cdot}10H_2O$ at 0 °C until there was no further effervescence. The reaction mixture was filtered through Celite and the Celite was washed thoroughly with Et_2O (Caution: any unquenched $LiAlH_4$ on the Celite was quenched with water before disposal). The solvent removed under reduced pressure to afford the crude amine which was purified by vacuum distillation.

3-Methylhexan-3-ol (S4)

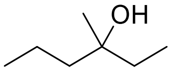

General procedure B was followed using methylmagnesium chloride (5.36 mL, 15 mmol, 2.8 M solution in Et_2O) and 3-hexanone (1.23 mL, 10.0 mmol) to afford tertiary alcohol **S4** as a colourless oil (1.11 g, 95%). R_f = 0.40 (40% Et_2O/Pentane). IR (film)/cm^{-1} 3375 (br, OH), 2960, 2933, 2874, 1460, 1377, 1154. ^{1}H NMR (400 MHz, $CDCl_3$) δ 1.49 (q, *J* = 7.5 Hz, 2 H, CH_2), 1.46–1.39 (m, 2 H, CH_2), 1.36–1.30 (m, 2 H, CH_2), 1.16 (bs, 1 H, OH), 1.15 (s, 3 H, CH_3), 0.95–0.88 (m, 6 H, 2 × CH_3). ^{13}C NMR (101 MHz, $CDCl_3$) δ 72.9 (C_qOH), 43.8 (CH_2), 34.3 (CH_2), 26.4 (CH_3), 17.1 (CH_2), 14.7 (CH_3), 8.2 (CH_3). Spectroscopic data for this compound (^{1}H NMR, IR) is consistent with the literature.[18]

3-Methylheptan-3-ol (S5)

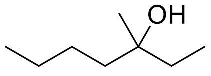

General procedure B was followed using methylmagnesium chloride (5.36 mL, 15 mmol, 2.8 M solution in Et_2O) and 3-heptanone (1.41 mL, 10.0 mmol) to afford tertiary alcohol **S5** as a colourless oil (1.33 g, 100%). R_f = 0.40 (40% Et_2O/Pentane). IR (film)/cm^{-1} 3371 (br, OH), 2961, 2932, 2882, 1738, 1460, 1376, 1217, 1151. ^{1}H NMR (400 MHz, $CDCl_3$) δ 1.49 (q, *J* = 7.5 Hz, 2 H, CH_2), 1.47–1.42 (m, 2 H, CH_2), 1.35–1.29 (m, 4 H, 2 × CH_2), 1.16 (bs, 1 H, OH), 1.15 (s, 3 H, CH_3), 0.94–0.88 (m, 6 H, 2 × CH_3). ^{13}C NMR (101 MHz, $CDCl_3$) δ 72.9 (C_qOH), 41.0 (CH_2), 34.2 (CH_2), 26.4 (CH_3), 26.1 (CH_2), 23.3 (CH_2), 14.1 (CH_3), 8.2 (CH_3). Spectroscopic data for this compound (NMR, IR) is consistent with the literature.[16]

3-Methylnonan-3-ol (S6)

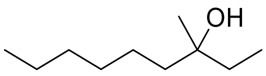

General procedure B was followed using methylmagnesium chloride (5.36 mL, 15 mmol, 2.8 M solution in Et_2O) and 3-nonanone (1.73 mL, 10.0 mmol) to afford tertiary alcohol **S6** as a colourless oil (1.31 g, 83%). R_f = 0.40 (40% Et_2O/Pentane). IR (film)/cm^{-1} 3368 (br, OH), 2961, 2929, 2858, 1738, 1460, 1375, 1148. ^{1}H NMR (400 MHz, $CDCl_3$) δ 1.48 (q, *J* = 7.5 Hz, 2 H, CH_2), 1.46–1.42 (m, 2 H, CH_2), 1.36–1.26 (m, 8 H, 4 × CH_2), 1.17 (bs, 1 H, OH), 1.14 (s, 3 H, CH_3), 0.91–0.88 (m, 6 H, 2 × CH_3). ^{13}C NMR (101 MHz, $CDCl_3$) δ 72.9 (C_qOH), 41.4 (CH_2), 34.2 (CH_2), 31.9 (CH_2), 29.9 (CH_2), 26.4 (CH_3), 23.8 (CH_2), 22.6 (CH_2), 14.1 (CH_3), 8.2 (CH_3). Spectroscopic data for this compound (NMR, IR) is consistent with the literature.[16]

1-Ethylcyclohexan-1-ol (S7)

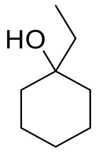

General procedure B was followed using ethylmagnesium bromide (6.00 mL, 15 mmol, 2.5 M solution in Et_2O) and cyclohexanone (1.04 mL, 10 mmol) to afford tertiary alcohol **S7** as a colourless oil (1.15 g, 90%). R_f = 0.40 (40% Et_2O/Pentane). IR (film)/cm^{-1} 3377 (br, OH), 2926, 2856, 1447, 1168, 1123. ^{1}H NMR (400 MHz, $CDCl_3$) δ 1.63–1.38 (m, 12 H, 6 × CH_2), 1.15 (bs, 1 H, OH), 0.91 (t, *J* = 7.5 Hz, 3 H, CH_3). ^{13}C NMR (101 MHz, $CDCl_3$) δ 71.4 (C_qOH), 36.9 (2 × CH_2), 34.7 (CH_2), 25.9 (CH_2), 22.2 (2 × CH_2), 7.2 (CH_3). Spectroscopic data for this compound (NMR, IR) is consistent with the literature.[16]

4-Ethyltetrahydro-2H-pyran-4-ol (S8)

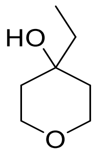

General procedure B was followed using ethylmagnesium bromide (6.00 mL, 15 mmol, 2.5 M solution in Et_2O) and tetrahydro-4H-pyran-4-one (0.92 mL, 10 mmol) to afford tertiary alcohol **S8** as a colourless oil (673 mg, 52%). R_f = 0.16 (60% Et_2O/Pentane). IR (film)/cm^{-1} 3396 (br, OH), 2944, 2868, 1464, 1388, 1238, 1159, 1096, 1021. ^{1}H NMR (400 MHz, $CDCl_3$) δ 3.81–3.73 (m, 4 H, 2 × OCH_2), 1.72–1.64 (m, 2 H, 2 × C(*H*)H), 1.56–1.44 (m, 4 H, 2 × C(H)*H* + CH_2), 1.17 (bs, 1 H, OH), 0.93 (t, J = 7.5 Hz, 3 H, CH_3). ^{13}C NMR (101 MHz, $CDCl_3$) δ 69.0 (C_qOH), 63.9 (2 × OCH_2), 37.1 (2 × CH_2), 35.7 (CH_2), 6.7 (CH_3). HRMS (APCI) m/z Calcd. for $C_7H_{15}O_2$ $[M+H]^+$: 131.1067; Found: 131.1069.

3-Methylheptan-1,1,1,2,2-d$_5$-3-ol (S9)

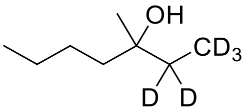

General procedure B was followed using (ethyl-d$_5$)magnesium bromide (6.25 mL, 12.5 mmol, 2.0 M solution in Et_2O, prepared from 1-bromoethane-1,1,2,2,2-d$_5$ (933 µL, 12.5 mmol), magnesium turnings (304 mg, 12.5 mL) in Et_2O (6.25 mL) using standard techniques) and hexan-2-one (1.23 mL, 10.0 mmol). Product was impure after workup (contained aldol by-products) and so the product was further purified by flash column chromatography (SiO_2, 10% Et_2O/pentane) followed by vacuum distillation to afford tertiary alcohol **S9** as a colourless oil (512 mg, 38%). R_f = 0.26 (20% Et_2O/Pentane). IR (film)/cm^{-1} 3441 (Br, OH), 2957, 2933, 1701, 1465, 1469, 1375, 1144. ^{1}H NMR (400 MHz, $CDCl_3$) δ 1.46–1.42 (m, 2 H, CH_2), 1.34–1.29 (m, 4 H, 2 × CH_2), 1.15 (s, 3 H, CH_2), 0.92 (t, *J* = 6.9 Hz, 3 H, CH_3). ^{13}C NMR (101 MHz, $CDCl_3$) δ 72.8 (C_qOH), 41.0 (CH_2), 26.4 (CH_3), 26.1 (CH_2), 23.3 (CH_2), 14.1 (CH_3).

3-Azido-3-methylhexane (S10)

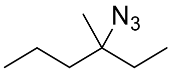

General procedure C was followed using 3-methylhexan-3-ol **S4** (1.00 g, 8.60 mmol) to afford tertiary azide **S10** as a colourless oil (1.21 g, 100%). R_f = 0.10 (40% Et_2O/Pentane). IR (film)/cm^{-1} 2964, 2936, 2875, 2084 (N_3), 1462, 1381, 1251, 1145. ^{1}H NMR (400 MHz, $CDCl_3$) δ 1.57–1.52 (m, 2 H, CH_2), 1.50–1.45 (m, 2 H, CH_2), 1.41–1.33 (m, 2 H, CH_2), 1.21 (s, 3 H, CH_3), 0.93 (q, *J* = 7.4 Hz, 6 H, 2 × CH_3). ^{13}C NMR (101 MHz, $CDCl_3$) δ 64.6 (C_qN_3), 41.2 (CH_2), 32.0 (CH_2), 22.8 (CH_3), 17.2 (CH_2), 14.4 (CH_3), 8.3 (CH_3).

3-Azido-3-methylheptane (S11)

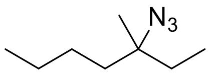

General procedure C was followed using 3-methylheptan-3-ol **S5** (1.27 g, 9.75 mmol) to afford tertiary azide **S11** as a colourless oil (1.33 g, 88%). R_f = 0.10 (40% Et_2O/Pentane). IR (film)/cm^{-1} 2963, 2935, 2864, 2085 (N_3), 1462, 1380, 1251. ^{1}H NMR (400 MHz, $CDCl_3$) δ 1.55 (qd, *J* = 7.7, 0.9 Hz, 2 H, CH_2), 1.52–1.47 (m, 2 H, CH_2), 1.22 (s, 3 H, CH_3), 1.34–1.30 (m, 4 H, 2 × CH_2), 0.95–0.90 (m, 6 H, 2 × CH_3). ^{13}C NMR (101 MHz, $CDCl_3$) δ 64.5 (C_qN_3), 38.6 (CH_2), 32.0 (CH_2), 26.1 (CH_2), 23.1 (CH_2), 22.8 (CH_3), 14.0 (CH_3), 8.3 (CH_3). Spectroscopic data for this compound (NMR, IR) is consistent with the literature.[16]

3-Azido-3-methylnonane (S12)

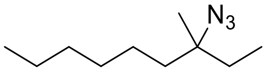

General procedure C was followed using 3-methylnonan-3-ol **S6** (1.27 g, 8.02 mmol) to afford tertiary azide **S12** as a colourless oil (1.39 g, 95%). R_f = 0.10 (40% Et_2O/Pentane). IR (film)/cm^{-1} 2959, 2931, 2859, 2086 (N_3), 1738, 1461, 1378, 1251. ^{1}H NMR (400 MHz, $CDCl_3$) δ 1.55 (q, *J* = 7.6 Hz, 2 H, CH_2), 1.51–1.45 (m, 2 H, CH_2), 1.38–1.24 (m, 8 H, 4 × CH_2), 1.21 (s, 3 H, CH_3), 0.94–0.88 (m, 6 H, 2 × CH_3). ^{13}C NMR (101 MHz, CDCl3) δ 64.6 (C_qN_3), 38.9 (CH_2), 32.0 (CH_2), 31.7 (CH_2), 29.6 (CH_2), 23.8 (CH_2), 22.8 (CH_3), 22.6 (CH_2), 14.0 (CH_3), 8.3 (CH_3). Spectroscopic data for this compound (NMR, IR) is consistent with the literature.[16]

1-Azido-1-ethylcyclohexane (S13)

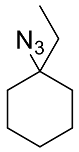

General procedure C was followed using 1-ethylcyclohexan-1-ol **S7** (1.10 g, 8.60 mmol) to afford tertiary azide **S13** as a colourless oil (1.27 g, 97%). R_f = 0.10 (40% Et_2O/Pentane). IR (film)/cm^{-1} 2931, 2971, 2094 (N_3), 1447, 1257, 1157, 1149. ^{1}H NMR (400 MHz, $CDCl_3$) δ 1.70–1.65 (m, 2 H, 2 × C(*H*)H), 1.62–1.51 (m, 7 H, 3 × CH_2 + C(H)*H*), 1.39–1.32 (m, 2 H, CH_2), 1.30–1.21 (m, 1 H, C(H)*H*), 0.95 (t, J = 7.5 Hz, 3 H, CH_3). ^{13}C NMR (101 MHz, $CDCl_3$) δ 64.4 (C_q-N_3), 34.2 (2 × CH_2), 32.8 (CH_2), 25.5 (CH_2), 22.2 (2 × CH_2), 7.7 (CH_3). Spectroscopic data for this compound (NMR, IR) is consistent with the literature.[17]

4-Azido-4-ethyltetrahydro-2H-pyran (S14)

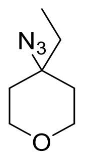

General procedure C was followed using 4-ethyltetrahydro-2H-pyran-4-ol **S8** (630 mg, 4.84 mmol) to afford tertiary azide **S14** as a pale yellow oil (750 mg, 100%). R_f = 0.16 (10% Et_2O/Pentane). IR (film)/cm^{-1} 2958, 2858, 2098 (N_3), 1382, 1257, 1159, 1104, 1020. ^{1}H NMR (400 MHz, $CDCl_3$) δ 3.81–3.77 (m, 2 H, 2 × OC(*H*)H), 3.70–3.63 (m, 2 H, 2 × OC(H)*H*), 1.69–1.59 (m, 6 H, 3 × CH_2), 0.99 (t, *J* = 7.5 Hz, 3 H, CH_3). ^{13}C NMR (101 MHz, $CDCl_3$) δ 69.6 (C_qN_3), 63.9 (2 × OCH_2), 34.3 (2 × CH_2), 33.1 (CH_2), 7.3 (CH_3). HRMS (APCI) m/z Calcd. for $C_7H_{12}N_3O$ [M-H]$^-$: 154.0975; Found: 154.0973.

3-Azido-3-methylheptane-1,1,1,2,2-d$_5$ (S15)

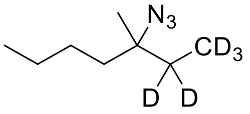

General procedure C was followed using 3-methylheptan-1,1,1,2,2-d$_5$-3-ol **S9** (512 mg, 3.79 mmol) to afford tertiary azide **S15** as a colourless oil (607 mg, 100%). R_f = 0.10 (40% Et_2O/Pentane). IR (film)/cm^{-1} 2960, 2935, 2864, 2093 (N_3), 1254, 1131. ^{1}H NMR (400 MHz, $CDCl_3$) δ 1.51–1.47 (m, 2 H, CH_2), 1.34–1.30 (m, 4 H, 2 × CH_2), 1.21 (s, 3 H, CH_3), 0.93 (t, *J* = 7.0 Hz, 3 H, CH_3). ^{13}C NMR (101 MHz, $CDCl_3$) δ 64.4 (C_qN_3), 38.6 (CH_2), 26.1 (CH_2), 23.1 (CH_2), 22.8 (CH_3), 14.0 (CH_3).

3-Methylhexan-3-amine (S16)

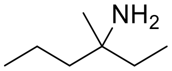

General procedure D was followed using 3-azido-3-methylhexane **S10** (1.21 g, 8.60 mmol) to afford amine **S16** as a colourless oil (305 mg, 28%). IR (film)/cm^{-1} 2958, 2931, 2873, 1583, 1461, 1376, 1179. ^{1}H NMR (400 MHz, $CDCl_3$) δ 1.35 (q, *J* = 7.6 Hz, 2 H, CH_2), 1.31–1.22 (m, 4 H, 2 × CH_2), 1.00 (s, 3 H, CH_3), 0.93–0.88 (m, 3 H, CH_3), 0.85 (t, *J* = 7.6 Hz, 3 H, CH_3). ^{13}C NMR (101 MHz, $CDCl_3$) δ 51.4 (C_qNH_2), 44.8 (CH_2), 35.1 (CH_2), 27.5 (CH_3), 17.1 (CH_2), 14.8 (CH_3), 8.2 (CH_3). Spectroscopic data for this compound (NMR) is consistent with the literature.[15]

3-Methylheptan-3-amine (30)

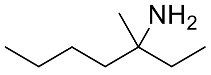

General procedure D was followed using 3-azido-3-methylheptane **S11** (1.30 g, 8.37 mmol) to afford amine **30** as a colourless oil (305 mg, 28%). IR (film)/cm^{-1} 2958, 2929, 2860, 1593, 1460, 1376, 1175. ^{1}H NMR (400 MHz, $CDCl_3$) δ 1.36 (q, *J* = 7.5 Hz, 2 H, CH_2), 1.32–1.20 (m, 8 H, 3 × CH_2 + NH_2), 1.00 (s, 3 H, CH_3), 0.91 (t, *J* = 6.9 Hz, 3 H, CH_3), 0.85 (t, *J* = 7.5 Hz, 3 H, CH_3). ^{13}C NMR (101 MHz, $CDCl_3$) δ 51.3 (C_qNH_2), 41.9 (CH_2), 35.0 (CH_2), 27.5 (CH_3), 26.2 (CH_2), 23.4 (CH_2), 14.1 (CH_3), 8.2 (CH_3). Spectroscopic data for this compound (NMR, IR) is consistent with the literature. Spectroscopic data for this compound (NMR, IR) is consistent with the literature.[15]

3-Methylnonan-3-amine (S17)

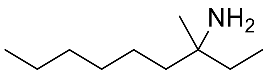

General procedure D was followed using 3-azido-3-methylnonane **S12** (1.34 g, 7.31 mmol) to afford amine **S17** as a colourless oil (600 mg, 52%). IR (film)/cm^{-1} 2958, 2926, 2855, 1610, 1460, 1375, 1173. ^{1}H NMR (400 MHz, $CDCl_3$) δ 1.36 (q, *J* = 7.5 Hz, 2 H, CH_2), 1.32–1.24 (m, 10 H, 5 × CH_2), 1.18 (bs, 2 H, NH_2), 1.01 (s, 3 H, CH_3), 0.90–0.84 (m, 6 H, 2 × CH_3). ^{13}C NMR (101 MHz, $CDCl_3$) δ 51.4 (C_qNH_2), 42.3 (CH_2), 35.1 (CH_2), 31.9 (CH_2), 30.1 (CH_2), 27.6 (CH_3), 23.9 (CH_2), 22.6 (CH_2), 14.1 (CH_3), 8.2 (CH_3). HRMS (TOF-ES$^+$) m/z Calcd. for $C_{10}H_{24}N$ $[M+H]^+$: 158.1909; Found: 158.1902. Spectroscopic data for this compound (NMR, IR) is consistent with the literature.[16]

1-Ethylcyclohexan-1-amine (S18)

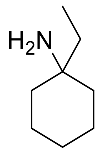

General procedure D was followed using 1-azido-1-ethylcyclohexane **S13** (1.23 g, 8.03 mmol) to afford amine **S18** as a colourless oil (685 mg, 67%). IR (film)/cm^{-1} 2964, 2921, 2849, 1596, 1449. ^{1}H NMR (400 MHz, $CDCl_3$) δ 1.47–1.26 (m, 12 H, 6 × CH_2), 1.19 (bs, 2 H, NH_2), 0.86 (q, *J* = 7.0 Hz, 3 H, CH_3). ^{13}C NMR (101 MHz, $CDCl_3$) δ 50.4 (C_qNH_2), 38.2 (2 × CH_2), 34.9 (CH_2), 26.1 (CH_2), 22.3 (2 × CH_2), 7.2 (CH_3). Spectroscopic data for this compound (NMR) is consistent with the literature.[15]

4-Ethyltetrahydro-2H-pyran-4-amine (S19)

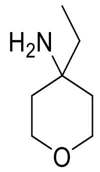

General procedure D was followed using 4-azido-4-ethyltetrahydro-2H-pyran **S14** (750 mg, 4.84 mmol) to afford amine **S19** as a pale yellow oil (53 mg, 8%). Due to low yield, amine was purified by flash column chromatography (20% MeOH/CH_2Cl_2) not distillation. R_f = 0.18 (20% MeOH/CH_2Cl_2). IR (film)/cm^{-1} 3370 (br, NH_2), 2960, 2938, 2863, 1601, 1462, 1237, 1180, 1107, 1079, 1016. ^{1}H NMR (400 MHz, $CDCl_3$) δ 3.80–3.68 (m, 4 H, 2 × CH_2), 1.67–1.60 (m, 4 H, CH_2 + NH_2), 1.47 (q, *J* = 7.5 Hz, 2 H, CH_2), 1.40–1.35 (m, 2 H, 2 × C(H)*H*), 0.91 (t, *J* = 7.5 Hz, 3 H, CH_3). ^{13}C NMR (101 MHz, $CDCl_3$) δ 63.9 (2 × OCH_2), 49.0 (C_qNH_2), 37.8 (2 × CH_2), 35.7 (CH_2), 6.8 (CH_3). HRMS (ES) m/z Calcd. for $C_7H_{16}NO$ $[M+H]^+$: 130.1232; Found: 130.1234.

3-Methylheptan-1,1,1,2,2-d$_5$-3-amine (D$_5$-30)

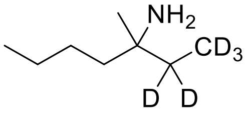

General procedure D was followed using 3-azido-3-methylheptane-1,1,1,2,2-d$_5$ **S15** (607 mg, 3.79 mmol) to afford amine **D$_5$-30** as a colourless oil (213 mg, 40%). IR (film)/cm^{-1} 2957, 2929, 2860, 2221, 1395, 1466, 1374 1057. ^{1}H NMR (400 MHz, $CDCl_3$) δ 1.34–1.18 (m, 8 H, 3 × CH_2 + NH_2), 1.01 (s, 3 H, CH_3), 0.91 (t, *J* = 6.9 Hz, 3 H, CH_3). ^{13}C NMR (101 MHz, $CDCl_3$) δ 51.2 (C_qNH_2), 42.0 (CH_2), 27.5 (CH_3), 26.2 (CH_2), 23.4 (CH_2), 14.1 (CH_3). HRMS (ES) m/z Calcd. for $C_8H_{16}ND_5$ $[M+H]^+$: 135.1910; Found: 135.1906.

*C(sp^3)–H arylation of amines (**14**-**22**)*

3-Methyl-1-phenylhexan-3-amine (14)

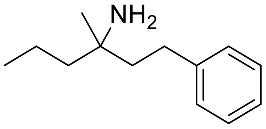

General procedure A was followed using 3-methylhexane-3-amine **S16** (35 mg, 0.30 mmol) and iodobenzene (101 µL, 0.90 mmol) to afford arylated amine **14** as a brown oil (22 mg, 38%). IR (film)/cm^{-1} 2956, 2929, 2870, 1495, 1454, 103. ^{1}H NMR (400 MHz, $CDCl_3$) δ 7.31–7.27 (m, 2 H, Ph-H), 7.21–7.17 (m, 3 H, Ph-H), 2.65–2.61 (m, 2 H, CH_2), 1.68–1.63 (m, 2 H, CH_2), 1.44–1.32 (m, 6 H, 2 × CH_2 + NH_2), 1.12 (s, 3 H, CH_3), 0.95 (t, *J* = 6.8 Hz, 3 H, CH_3). ^{13}C NMR (101 MHz, $CDCl_3$) δ 142.9 (Ph-C_q), 128.4 (2 × Ph-C), 128.3 (2 × Ph-C), 125.6 (Ph-C), 51.5 (C_qNH_2), 45.4 (CH_2), 44.9 (CH_2), 30.6 (CH_2), 28.1 (CH_3), 17.2 (CH_2), 14.8 (CH_3). Spectroscopic data for this compound (NMR, IR) is consistent with the literature.[15]

Using CF_3-acetal **12** (12 mg, 0.045 mmol), amine **14** was afforded as a brown oil (28 mg, 49%), data obtained was identical.

3-Methyl-1-phenylheptan-3-amine (15)

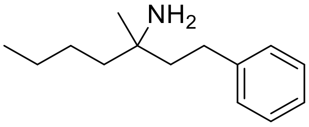

General procedure A was followed using 3-methylheptane-3-amine **30** (39 mg, 0.30 mmol) and iodobenzene (101 µL, 0.90 mmol) to afford arylated amine **15** as a yellow oil (33 mg, 54%). IR (film)/cm^{-1} 2955, 2928, 2859, 1495, 1454, 1376. ^{1}H NMR (400 MHz, $CDCl_3$) δ 7.31–7.27 (m, 2 H, Ph-H), 7.22–7.16 (m, 3 H, Ph-H), 2.66–2.61 (m, 2 H, CH_2), 1.68–1.64 (m, 2 H, CH_2), 1.44–1.25 (m, 8 H, 3 × CH_2 + NH_2), 1.12 (s, 3 H, CH_3), 0.94 (t, J = 6.9 Hz, 3 H, CH_3). ^{13}C NMR (101 MHz, $CDCl_3$) δ 142.9 (Ph-C_q), 128.4 (2 × Ph-C), 128.3 (2 × Ph-C), 125.7 (Ph-C), 51.4 (C_qNH_2), 44.8 (CH_2), 42.7 (CH_2), 30.6 (CH_2), 28.1 (CH_3), 26.2 (CH_2), 23.4 (CH_2), 14.1 (CH_3). HRMS (TOF-ES$^+$) m/z Calcd. for $C_{14}H_{24}N$ $[M+H]^+$: 206.1909; Found: 206.1919. Spectroscopic data for this compound (NMR, IR) is consistent with the literature.[15]

3-Methyl-1-phenylnonan-3-amine (16)

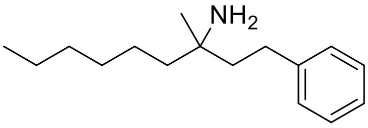

General procedure A was followed using 3-methylnonane-3-amine **S17** (47 mg, 0.30 mmol) and iodobenzene (101 µL, 0.90 mmol) to afford arylated amine **16** as a yellow oil (13 mg, 19%). IR (film)/cm^{-1} 2955, 2927, 2856, 1670, 1454, 1375. ^{1}H NMR (400 MHz, $CDCl_3$) δ 7.31–7.27 (m, 2 H, Ph-H), 7.21–7.17 (m, 3 H, Ph-H), 2.65–2.61 (m, 2 H, CH_2), 1.68–1.63 (m, 2 H, CH_2), 1.44–1.37 (m, 2 H, NH_2), 1.36–1.25 (m, 10 H, 5 × CH_2), 1.12 (s, 3 H, CH_3), 0.92–0.86 (m, 3 H, CH_3). ^{13}C NMR (101 MHz, $CDCl_3$) δ 142.9 (Ph-C_q), 128.4 (2 × Ph-C), 128.3 (2 × Ph-C), 125.7 (Ph-C), 51.5 (C_qNH_2), 44.8 (CH_2), 42.9 (CH_2), 31.9, (CH_2) 30.6 (CH_2), 30.0 (CH_2), 28.1 (CH_3), 24.0 (CH_2), 22.6 (CH_2), 14.1 (CH_3). Spectroscopic data for this compound (NMR) is consistent with the literature.[16]

Using CF_3-acetal **12** (12 mg, 0.045 mmol), amine **16** was afforded as a yellow oil (16 mg, 23%), data obtained was identical.

1-Phenethylcyclohexan-1-amine (17)

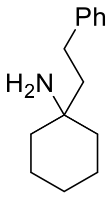

General procedure A was followed using 1-ethylcyclohexan-1-amine **S18** (38 mg, 0.30 mmol) to afford arylated amine **17** as a brown oil (19 g, 31%). IR (film)/cm^{-1} 2923, 2854, 1496, 1452, 1058, 1032. ^{1}H NMR (400 MHz, $CDCl_3$) δ 7.31–7.27 (m, 2 H, Ph-H), 7.23–7.17 (m, 3 H, Ph-H), 2.68–2.64 (m, 2 H, CH_2), 1.70–1.66 (m, 2 H, CH_2), 1.58–1.36 (m, 12 H, 5 × CH_2 + NH_2). ^{13}C NMR (101 MHz, $CDCl_3$) δ 143.1 (Ph-C_q), 128.4 (4 × Ph-C), 125.6 (Ph-C), 50.6 (C_q-NH_2), 38.8 (2 × CH_2), 29.5 (CH_2), 26.0 (CH_2), 22.3 (3 × CH_2). Spectroscopic data for this compound (NMR, IR) is consistent with the literature.[15]

Using CF_3-acetal **12** (12 mg, 0.045 mmol), amine **17** was afforded as a brown oil (25 mg, 41%), data obtained was identical.

4-Phenethyltetrahydro-2H-pyran-4-amine (18)

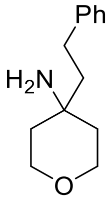

General procedure A was followed using 4-ethyltetrahydro-2H-pyran-4-amine **S19** (39 mg, 0.30 mmol) to afford arylated amine **18** as a brown oil (24 mg, 39%). IR (film)/cm^{-1} 2935, 2861, 1602, 1454, 1236, 1104, 1019, 1032. ^{1}H NMR (400 MHz, $CDCl_3$) δ 7.32–7.27 (m, 2 H, Ph-H), 7.22–7.18 (m, 3 H, Ph-H), 3.82–3.71 (m, 4 H, 2 × OCH_2), 2.71–2.66 (m, 2 H, CH_2), 1.76–1.68 (m, 4 H, CH_2 + 2 × C(*H*)H), 1.46–1.41 (m, 4 H, 2 × C(H)*H* + NH_2). ^{13}C NMR (101 MHz, $CDCl_3$) δ 142.4 (Ph-C_q), 128.5 (2 × Ph-C), 128.3 (2 × Ph-C), 125.8 (Ph-C), 64.0 (2 × OCH_2), 48.6 (C_q-NH_2), 46.0 (CH_2), 38.7 (2 × CH_2), 29.1 (CH_2). HRMS (TOF-ES$^+$) m/z Calcd. for $C_{13}H_{20}NO$ $[M+H]^+$: 206.1545; Found: 206.1547.

General procedure E (to avoid alpha oxidation)

AgTFA (99 mg, 0.45 mmol), $Pd(OPiv)_2$ (9.3 mg, 0.03 mmol, 10 mol%), iodobenzene (3.00 equiv), H_2O (27 µL, 1.50 mmol), amine (0.30 mmol), (2,2-dimethoxyethoxy)benzene **8** (7.6 µL, 0.045 mmol), AcOH (1.25 mL) and HFIP (0.25 mL) were combined in a microwave vial. The vial was sealed and the reaction was stirred at 110 °C for 3 h. The reaction was allowed to cool to rt and filtered through a bed of Celite which was washed with Et_2O. The product was extracted from the organic phase into 1 M aqueous HCl solution. The combined aqueous extracts were basified with saturated aqueous sodium hydroxide solution, and the free amine extracted with CH_2Cl_2, dried (Na_2SO_4) and the solvent was removed under reduced pressure to afford the title amines.

2,2-Dimethyl-3-phenylpropan-1-amine (19-mono) and 2-benzyl-2-methyl-3-phenylpropan-1-amine (19-di)

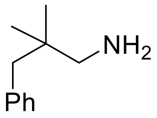

19-mono

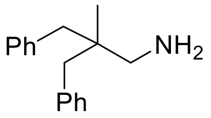

19-di

General procedure E was followed using 2,2-dimethylpropan-1-amine (35 µL, 0.30 mmol) and iodobenzene (101 µL, 0.90 mmol) to afford a (3.55:1) mixture of monoarylated amine **19-mono** (11 mg, 22%) and diarylated amine **19-di** (6 mg, 8%) as a brown oil. IR (film)/cm^{-1} 3027, 2955, 1484, 1452, 1369. ^{13}C NMR (101 MHz, $CDCl_3$) δ 139.0, 138.7, 130.7, 130.5, 127.8, 127.7, 125.9, 125.8, 52.6, 48.5, 45.7, 44.3, 39.6, 35.9, 27.2, 24.5, 21.9.

2,2-Dimethyl-3-phenylpropan-1-amine (19-mono)

^{1}H NMR (400 MHz, $CDCl_3$) δ 7.30–7.27 (m, 2 H, Ph-H), 7.27–7.19 (m, 1 H, Ph-H), 7.18–7.11 (m, 2 H, Ph-H), 2.54 (s, 2 H, CH_2), 2.49 (s, 2 H, CH_2), 0.86 (s, 6 H, $C(CH_3)_2$). Spectroscopic data for this compound (NMR, IR) is consistent with the literature.[19]

2-Benzyl-2-methyl-3-phenylpropan-1-amine (19-di)

^{1}H NMR (400 MHz, $CDCl_3$) δ 7.30–7.27 (m, 4 H, Ph-H), 7.27–7.19 (m, 2 H, Ph-H), 7.18–7.11 (m, 4 H, Ph-H), 2.71 (d, J = 13.1 Hz, 2 H, 2 × C*H*(H)), 2.60 (d, J = 13.2 Hz, 2 H, 2 × CH(*H*)), 2.45 (s, 2 H, CH_2), 0.79 (s, 3 H, CH_3). HRMS (TOF-ES$^+$) m/z Calcd. for $C_{17}H_{22}N$ $[M+H]^+$: 240.1752; Found: 240.1745.

3-Methyl-4-phenylbutan-2-amine (20-mono) and 3-benzyl-4-phenylbutan-2-amine (20-di)

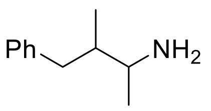

20-mono

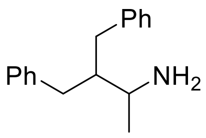

20-di

General procedure E was followed using 3-methylbutan-2-amine (35 µL, 0.30 mmol) and iodobenzene (101 µL, 0.90 mmol) to afford a (2:1) mixture of monoarylated amine **20-mono** (11 mg, 22%) and diarylated amine **20-di** (8 mg, 11%) as a brown oil. IR (film)/cm^{-1} 3025, 2962, 2926, 1669, 1600, 1494, 1452, 1374, 1029. ^{13}C NMR (101 MHz, $CDCl_3$) δ 141.3, 129.1, 129.0, 128.3, 128.2, 125.8, 125.7, 50.5, 46.7, 42.4, 39.7, 35.7, 20.1, 19.5, 14.4.

3-Methyl-4-phenylbutan-2-amine (20-mono)

^{1}H NMR (400 MHz, $CDCl_3$) δ 7.35–7.25 (m, 2 H, Ph-H), 7.21–7.12 (m, 3 H, Ph-H), 2.88 (dt, *J* = 12.7, 6.4 Hz, 1 H, C*H*NH_2), 2.80 (dd, *J* = 13.3, 5.1 Hz, 1 H, PhC(*H*)H), 2.33 (dd, *J* = 13.3, 9.5 Hz, 1 H, PhC(H)*H*), 1.76–1.68 (m, 1 H, C*H*CH_3), 1.09 (d, *J* = 6.5 Hz, 3 H, CH_3), 0.83 (d, *J* = 6.8 Hz, 3 H, CH_3). HRMS (TOF-ES$^+$) m/z Calcd. for $C_{11}H_{18}N$ $[M+H]^+$: 164.1439; Found: 164.1438.

3-Benzyl-4-phenylbutan-2-amine (20-di)

δ 7.35–7.25 (m, 4 H, Ph-H), 7.21–7.12 (m, 6 H, Ph-H), 2.99–2.92 (m, 1 H, C*H*NH_2), 2.77–2.72 (m, 1 H, PhC(*H*)H), 2.63 (dd, *J* = 13.8, 7.0 Hz, 1 H, PhC(*H*)H), 2.55 (dd, *J* = 13.8, 7.5 Hz, 1 H, PhC(H)*H*), 2.48 (dd, *J* = 13.7, 7.3 Hz, 1 H, PhC(H)*H*), 2.03–1.96 (m, 1 H, CH), 1.11 (d, *J* = 6.4 Hz, 3 H, CH_3). HRMS (TOF-ES$^+$) m/z Calcd. for $C_{17}H_{22}N$ $[M+H]^+$: 240.1752; Found: 240.1750.

3,3-Dimethyl-4-phenylbutan-2-amine (21-mono) and 3-benzyl-3-methyl-4-phenylbutan-2-amine (21-di)

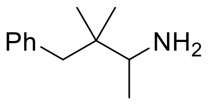

21-mono

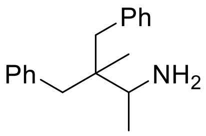

21-di

General procedure E was followed using 3,3-dimethylbutan-2-amine (40 µL, 0.30 mmol) and iodobenzene (101 µL, 0.90 mmol) to afford a (1.25:1) mixture of monoarylated amine **21-mono** (10 mg, 19%) and diarylated amine **21-di** (11 mg, 14%) as a yellow oil. IR (film)/cm^{-1} 3026, 2962, 2872, 1601, 1494, 1452, 1375, 1031. ^{13}C NMR (101 MHz, $CDCl_3$) δ 139.2, 138.9, 130.9, 130.8, 130.7, 127.8, 127.7, 125.9, 125.8, 54.4, 50.9, 44.6, 41.6, 41.5, 41.3, 38.1, 23.1, 22.5, 22.2, 18.5.

3,3-Dimethyl-4-phenylbutan-2-amine (21-mono)

^{1}H NMR (400 MHz, $CDCl_3$) δ 7.31–7.28 (m, 2 H, Ph-H), 7.26–7.19 (m, 1 H, Ph-H), 7.19–7.14 (m, 2 H, Ph-H), 2.79 (q, *J* = 6.6 Hz, 1 H, CH), 2.70 (s, 2 H, CH_2), 1.09 (d, *J* = 6.6 Hz, 3 H, CH_3), 0.85 (s, 3 H, CH_3). 0.84 (s, 3 H, CH_3). HRMS (TOF-ES$^+$) m/z Calcd. for $C_{12}H_{20}N$ $[M+H]^+$: 178.1596; Found: 178.1588.

3-Benzyl-3-methyl-4-phenylbutan-2-amine (21-di)

^{1}H NMR (400 MHz, $CDCl_3$) δ 7.31–7.28 (m, 4 H, Ph-H), 7.26–7.19 (m, 2 H, Ph-H), 7.19–7.14 (m, 4 H, Ph-H), 2.90 (d, *J* = 13.2 Hz, 1 H, C*H*(H)), 2.72 (q, *J* = 6.6 Hz, 1 H, CH), 2.63 (d, *J* = 12.9 Hz, 1 H, C*H*(H)), 2.53 (d, *J* = 12.9 Hz, 1 H, CH(*H*)), 2.44 (d, *J* = 13.1 Hz, 1 H CH(*H*)), 1.15 (d, *J* = 6.6 Hz, 3 H, CH_3), 0.82 (s, 3 H, CH_3). HRMS (TOF-ES$^+$) m/z Calcd. for $C_{18}H_{24}N$ $[M+H]^+$: 254.1909; Found: 254.1914.

trans-2-Benzylcyclohexan-1-amine (22)

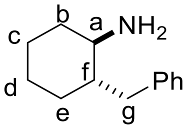

General procedure A was followed using *cis/trans*-2-methylcyclohexan-1-amine (40 µL, 0.30 mmol, approx 3:7 *cis*:*trans*) and iodobenzene (101 µL, 0.90 mmol) to afford arylated *trans*-amine **22** as a yellow oil (19 mg, 33%). IR (film)/cm^{-1} 3324, 2920, 2852, 1606, 1582, 1551, 1493, 1445, 1284. ^{1}H NMR (400 MHz, $CDCl_3$) δ 7.30–7.26 (m, 2 H, Ph-H), 7.21–7.16 (m, 3 H, Ph-H), 3.17 (dd, *J* = 13.2, 3.8 Hz, 1 H, PhCH^g(H)), 2.41 (td, J = 10.2, 4.0 Hz, 1 H, C$H^a$$NH_2$), 2.24 (dd, *J* = 13.2, 9.8 Hz, 1 H, PhCH(H^g)), 1.89–1.85 (m, 1 H, CH^c(H)), 1.72–1.66 (m, 1 H, CH^b(H)), 1.63–1.54 (m, 2 H, CH^e(H) + CH^d(H)), 1.45–1.03 (m, 6 H, CH^f + CH(H^b) + CH(H^c) + CH(H^d) + NH_2), 0.94–0.83 (m, 1 H, CH(H^e)). ^{13}C NMR (101 MHz, $CDCl_3$) δ 141.0 (Ar-C_q), 129.3 (2 × Ar-C), 128.1 (2 × Ar-C), 125.6 (Ar-C), 54.9 (C^aHNH_2), 47.6 (C^fH), 39.5 (PhC^gH_2), 36.8 (C^bH_2), 30.4 (C^eH_2), 25.8 (CH_2), 25.5 (CH_2). HRMS (TOF-ES$^+$) m/z Calcd. for $C_{13}H_{20}N$ $[M+H]^+$: 190.1596; Found: 190.1599.

H^a exhibits 2 large *trans* diaxial H–H splitting (10 Hz, b_{ax} and f_{ax}) and one small *cis* axial-equatorial splitting (4.0 Hz, b_{eq}) consistent with proposed trans stereochemistry with a di-equatorial conformation for the substituents.

Isomeric ratio of the starting material:

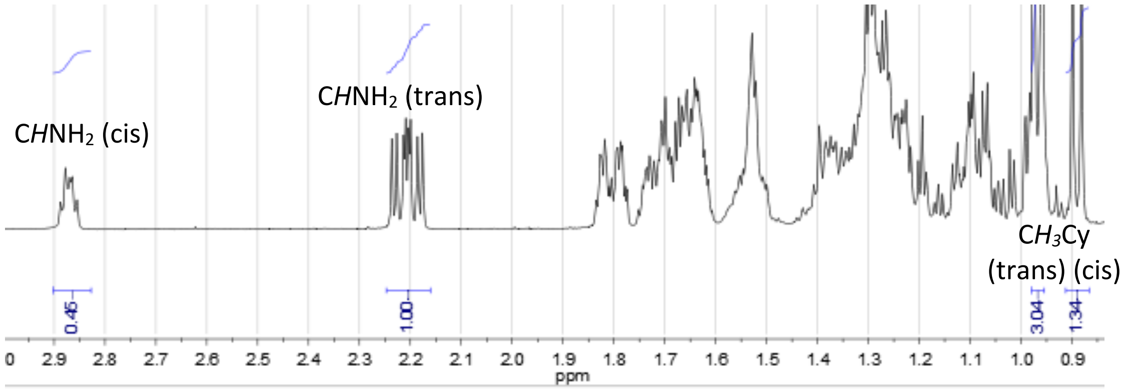

cis/trans Assigned by chemical shift, *J* values and literature NMR for a *trans*-enantiomer.[20]

Calculated ratio: *cis*:*trans* 3:7

*Preparation of additives for structural comparison of the optimal directing group (**23, 24, 27-29**)*

Additives **25** and **26** were commercially available.

(2,2-Dimethoxypropoxy)benzene (23)

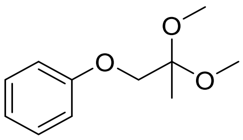

Trimethoxymethane (164 µL, 1.50 mmol) was added to a stirred solution of 1-phenoxypropan-2-one (150 µL, 1.00 mmol) and 4-methylbenzenesulfonic acid monohydrate (1.9 mg, 0.01 mmol) in MeOH (10 mL) and the reaction was heated to 60 °C overnight. The reaction was allowed to cool to rt, $MgSO_4$ was added, the mixture was filtered and solvent removed under reduced pressure. Purification by flash column chromatography (SiO_2, 10% Et_2O/Pentane) afforded acetal **23** as a colourless oil (196 mg, 100%) R_f 0.29 (10% Et_2O/pentane). IR (film)/cm^{-1} 2944, 2831, 1600, 1588, 1496, 1475, 1374, 1235, 1119, 1083, 1060. ^{1}H NMR (400 MHz, $CDCl_3$) δ 7.33–7.28 (m, 2 H, Ph-H), 7.00–6.95 (m, 3 H, Ph-H), 3.92 (s, 2 H, CH_2), 3.29 (s, 6 H, $CH(OC\mathit{H}_3)_2$), 1.49 (s, 3 H CH_3). ^{13}C NMR (101 MHz, $CDCl_3$) δ 158.7 (Ph-C_q), 129.4 (2 × Ph-C), 121.1 (Ph-C), 114.7 (2 × Ph-C), 99.9 ($\mathit{C}H(OCH_3)_2$), 68.7 (CH_2), 48.4 (2 × OCH_3), 20.3 (CH_3). HRMS (EI$^+$) m/z Calcd. for $C_{11}H_{16}O_3$ $[M]^+$: 196.1099; Found: 196.1108.

(3,3-Dimethoxypropyl)benzene (24)

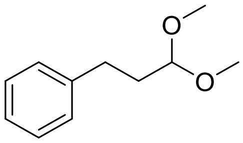

4-Methylbenzenesulfonic acid monohydrate (380 mg, 0.20 mmol) in MeOH (2.0 mL) was added to a stirred solution of 3-phenylpropanal (2.60 mL, 20.0 mmol) and trimethoxymethane (3.30 mL, 30.0 mmol) in MeOH (4.0 mL) containing a small amount of 3 Å molecular sieves and the reaction was stirred at 25 °C for 2 h. The reaction was diluted with Et_2O, saturated aqueous sodium bicarbonate was added and the product extracted with Et_2O, dried (Na_2SO_4), filtered and solvent removed under reduced pressure. Vacuum distillation afforded acetal **24** as a colourless oil (468 mg, 13%) R_f 0.32 (10% Et_2O/pentane). IR (film)/cm^{-1} 2951, 2829, 1496, 1454, 1191, 1171, 1123, 1051. ^{1}H NMR (400 MHz, $CDCl_3$) δ 7.32–7.28 (m, 2 H, Ph-H), 7.22–7.18 (m, 3 H, Ph-H), 4.38 (t, J = 5.7 Hz, 1 H, CH), 3.35 (s, 6 H, $CH(OC\mathit{H}_3)_2$), 2.71–2.67 (m, 2 H, CH_2), 1.97–1.91 (m, 2 H, CH_2). ^{13}C NMR (101 MHz, $CDCl_3$) δ 141.6 (Ph-C_q), 128.4 (4 × Ph-C), 125.9 (Ph-H), 103.7 ($\mathit{C}H(OCH_3)_2$), 52.7 ($CH(O\mathit{C}H_3)_2$), 34.1 (CH_2), 30.9 (CH_2). Spectroscopic data for this compound (NMR, IR) is consistent with the literature.[21]

(2-Methoxyethoxy)benzene (27)

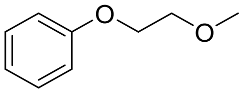

Sodium hydride (48 mg, 1.20 mmol as 60% dispersion in mineral oil) was added to a stirred solution of 2-phenoxyethan-1-ol (251 µL, 2.00 mmol) in THF (6.7 mL) at 0 °C and the reaction was stirred for 15 minutes. Iodomethane (131 µL, 2.10 mmol) was added and the reaction was stirred at rt overnight. Water was added and the product extracted with Et_2O, dried ($MgSO_4$), filtered and the solvent removed under reduced pressure. Purification by flash column chromatography (SiO_2, 10% Et_2O/Pentane) afforded the methyl ether **27** as a colourless oil (158 mg, 32%). R_f 0.21 (10% Et_2O/pentane). IR (film)/cm^{-1} 2926, 2878, 2819, 1598, 1587, 1495, 1453, 1243, 1126, 1060, 1033. ^{1}H NMR (400 MHz, $CDCl_3$) δ 7.31–7.26 (m, 2 H, Ph-H), 6.98–6.92 (m, 3 H, Ph-H), 4.14–4.12 (m, 2 H, CH_2), 3.77–3.75 (m, 2 H, CH_2), 3.46 (s, 3 H, OCH_3). ^{13}C NMR (101 MHz, $CDCl_3$) δ 158.7 (Ph-C_q), 129.4 (2 × Ph-C), 120.9 (Ph-C), 114.6 (2 × Ph-C), 71.1 (OCH_2), 67.1 (OCH_2), 59.2 (OCH_3). Spectroscopic data for this compound (^{1}H NMR, IR) is consistent with the literature.[22]

(2-Methoxypropoxy)benzene (28)

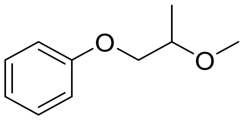

Sodium hydride (40 mg, 1.00 mmol as 60% dispersion in mineral oil) was added to a stirred solution of 2-methyl-1-phenoxypropan-2-ol (83 mg, 0.50 mmol) in THF (1.0 mL) at 0 °C and the reaction was allowed to warm to rt and stirred for 1 h. Iodomethane (93 µL, 1.5 mmol) was added and the reaction was stirred at rt overnight. Water was added and the product extracted with Et_2O, dried ($MgSO_4$), filtered and the solvent removed under reduced pressure. Purification by flash column chromatography (SiO_2, 10% Et_2O/Pentane) afforded ether **28** as a colourless oil (66 mg, 79%). R_f 0.32 (20% Et_2O/pentane). IR (film)/cm^{-1} 2976, 2930, 2877, 2824, 1599, 1587, 1495, 1455, 1290, 1242, 1079, 1037. ^{1}H NMR (400 MHz, $CDCl_3$) δ 7.32–7.27 (m, 2 H, Ph-H), 6.98–6.91 (m, 3 H, Ph-H), 4.00 (dd, J = 9.7, 5.8 Hz, 1 H, C(*H*)H), 3.90 (dd, J = 9.7, 4.5 Hz, 1 H, C(H)*H*), 3.77–3.70 (m, 1 H, CH), 3.47 (s, 3 H, OCH_3), 1.29 (d, J = 6.3 Hz, 3 H, CH_3). ^{13}C NMR (101 MHz, $CDCl_3$) δ 158.9 (Ph-C_q), 129.4 (2 × Ph-C), 120.8 (Ph-C), 114.6 (2 × Ph-C), 75.3 (CH), 71.3 (CH_2), 57.0 (OCH_3), 16.7 (CH_3). HRMS (ESI) m/z Calcd. for $C_{10}H_{15}O_2$ $[M+H]^+$: 167.1072; Found: 167.1064.

2-Methyl-1-phenoxypropan-2-ol (S20)

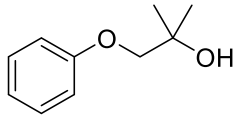

2,2-Dimethyloxirane (266 µL, 3.00 mmol), phenol (94 mg, 1.00 mmol), K_2CO_3 (276 mg, 2.00 mmol) and DMF (1 mL) were combined in a microwave vial, sealed and heated to 150 °C in a microwave reactor for 30 minutes. Water was added and the product extracted with EtOAc, dried ($MgSO_4$), filtered and solvent removed under reduced pressure. Purification by flash column chromatography (20% Et_2O/pentane) afforded alcohol **S20** as a colourless oil (174 mg, 87%). R_f 0.26 (20% Et_2O/pentane). IR (film)/cm^{-1} 3400, 2974, 2930, 2871, 1599, 1587, 1495, 1456, 1230, 1170, 1044. ^{1}H NMR (400 MHz, $CDCl_3$) δ 7.33–7.28 (m, 2 H, Ph-H), 7.00–6.93 (m, 3 H, Ph-H), 3.81 (s, 2 H, CH_2), 2.22 (bs, 1 H, OH), 1.36 (s, 6 H, $C(CH_3)_2$). ^{13}C NMR (101 MHz, $CDCl_3$) δ 158.7 (Ar-C_q), 129.5 (2 × Ar-C), 121.1 (Ar-C), 114.6 (2 × Ar-C), 75.9 (CH_2), 70.1 (*C*(CH_3)$_2$), 26.1 (C(*C*H_3)$_2$). Spectroscopic data for this compound (NMR, IR) is consistent with the literature.[23]

(2-Methoxy-2-methylpropoxy)benzene (29)

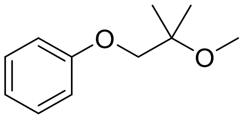

Sodium hydride (40 mg, 1.00 mmol as 60% dispersion in mineral oil) was added to a stirred solution of 2-methyl-1-phenoxypropan-2-ol **S20** (83 mg, 0.50 mmol) in THF (1.0 mL) at 0 °C and the reaction was allowed to warm to rt and stirred for 1 h. Iodomethane (93 µL, 1.5 mmol) was added and the reaction was stirred at rt overnight. Water was added and the product extracted with Et_2O, dried ($MgSO_4$), filtered and the solvent removed under reduced pressure. Purification by flash column chromatography (SiO_2, 10% Et_2O/Pentane) afforded ether **29** as a colourless oil (76 mg, 84%). R_f 0.32 (20% Et_2O/pentane). IR (film)/cm^{-1} 2975, 2936, 2829, 1599, 1587, 1496, 1471, 1364, 1238, 1184, 1170, 1080, 1048. ^{1}H NMR (400 MHz, $CDCl_3$) δ 7.32–7.27 (m, 2 H, Ph-H), 6.98–6.93 (m, 3 H, Ph-H), 3.84 (s, 2 H, CH_2), 3.32 (s, 3 H, OCH_3), 1.32 (s, 6 H. $C(CH_3)_2$). ^{13}C NMR (101 MHz, $CDCl_3$) δ 159.0 (Ar-C_q), 129.4 (2 × Ar-C), 120.8 (Ar-C), 114.6 (2 × Ar-C), 74.3 (*C_q*(CH_3)$_2$), 73.6 (OCH_2), 49.9 (OCH_3), 22.4 (C(*C*H_3)$_2$). HRMS (EI^+) m/z Calcd. for $C_{11}H_{16}O_2$ $[M]^+$: 180.1150; Found: 180.1157.

References

[1] S. St John-Campbell, A. J. P. White, J. A. Bull, *Chem. Sci.* **2017**, *8*, 4840–4847.
[2] M. Largeron, *European J. Org. Chem.* **2013**, *2013*, 5225–5235.
[3] J. Sheng, Y. Guo, J. Wu, *Tetrahedron* **2013**, *69*, 6495–6499.
[4] J. J. Topczewski, P. J. Cabrera, N. I. Saper, M. S. Sanford, *Nature* **2016**, *531*, 220–224.
[5] D. G. Blackmond, *Angew. Chemie Int. Ed.* **2005**, *44*, 4302–4320.
[6] R. D. Baxter, D. Sale, K. M. Engle, J.-Q. Yu, D. G. Blackmond, *J. Am. Chem. Soc.* **2012**, *134*, 4600–4606.
[7] J. Burés, *Angew. Chemie Int. Ed.* **2016**, *55*, 2028–2031.
[8] J. Burés, *Angew. Chemie Int. Ed.* **2016**, *55*, 16084–16087.
[9] X. Hu, J. Liu, L. Wang, F. Huang, C.-Z. Sun, D.-Z. Chen, *Org. Chem. Front.* **2018**, *5*, 1670–1678.
[10] D. L. Boger, C. E. Brotherton, M. D. Kelley, *Tetrahedron* **1981**, *37*, 3977–3980.
[11] H. C. Arndt, S. A. Carroll, *Synthesis.* **1979**, *1979*, 202–204.
[12] Y. S. Hon, Y. C. Wong, C. P. Chang, C. H. Hsieh, *Tetrahedron* **2007**, *63*, 11325–11340.
[13] U. Wannagat, V. Damrath, U. Harder, *Monatshefte fur Chemie* **1994**, *1169*, 1159–1169.
[14] M. Rueping, C. Vila, U. Uria, *Org. Lett.* **2012**, *14*, 768–771.
[15] Y. Liu, H. Ge, *Nat. Chem.* **2016**, *9*, 26–32.
[16] M. Kapoor, D. Liu, M. C. Young, *J. Am. Chem. Soc.* **2018**, *140*, 6818–6822.
[17] K. Chen, D. Wang, Z.-W. Li, Z. Liu, F. Pan, Y.-F. Zhang, Z.-J. Shi, *Org. Chem. Front.* **2017**, *4*, 2097–2101.
[18] A. J. M. Janssen, A. J. H. Klunder, B. Zwanenburg, *Tetrahedron* **1991**, *47*, 7645–7662.
[19] X. Sun, X. Wen, Y. Chen, C. Shi, C. Gao, Y. Wu, L. Wang, X. Yang, H. Sun, *Eur. J. Med. Chem.* **2015**, *103*, 269–288.
[20] S. Marino, D. Stachurska-Buczek, D. Huggins, B. Krywult, C. Sheehan, T. Nguyen, N. Choi, J. Parsons, P. Griffiths, I. James, et al., *Molecules* **2004**, *9*, 405–426.
[21] Y.-S. Hon, C.-F. Lee, R.-J. Chen, Y.-F. Huang, *Synth. Commun.* **2003**, *33*, 2829–2842.
[22] A. Wada, S. Kanatomo, S. Nagai, *Chem. Pharm. Bull.* **1985**, *33*, 1016–1022.
[23] P. S. Dragovich, T. J. Prins, R. Zhou, *J. Org. Chem.* **1995**, *60*, 4922–4924.

^{1}H and ^{13}C NMR spectra of selected compounds

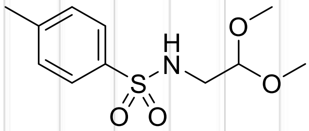

3

^{1}H NMR (400 MHz, $CDCl_3$)

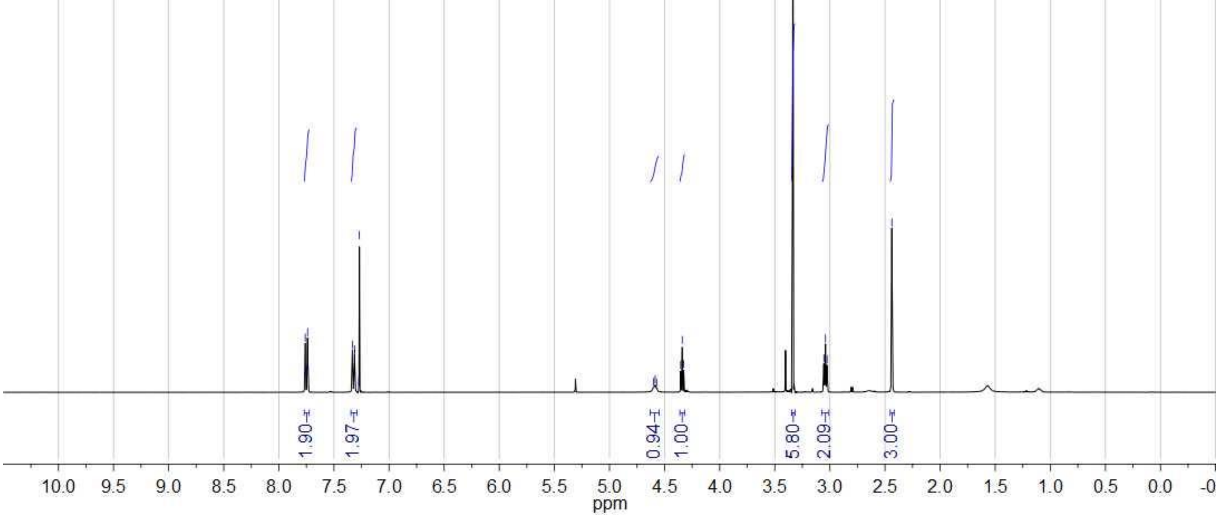

3

^{13}C NMR (101 MHz, $CDCl_3$)

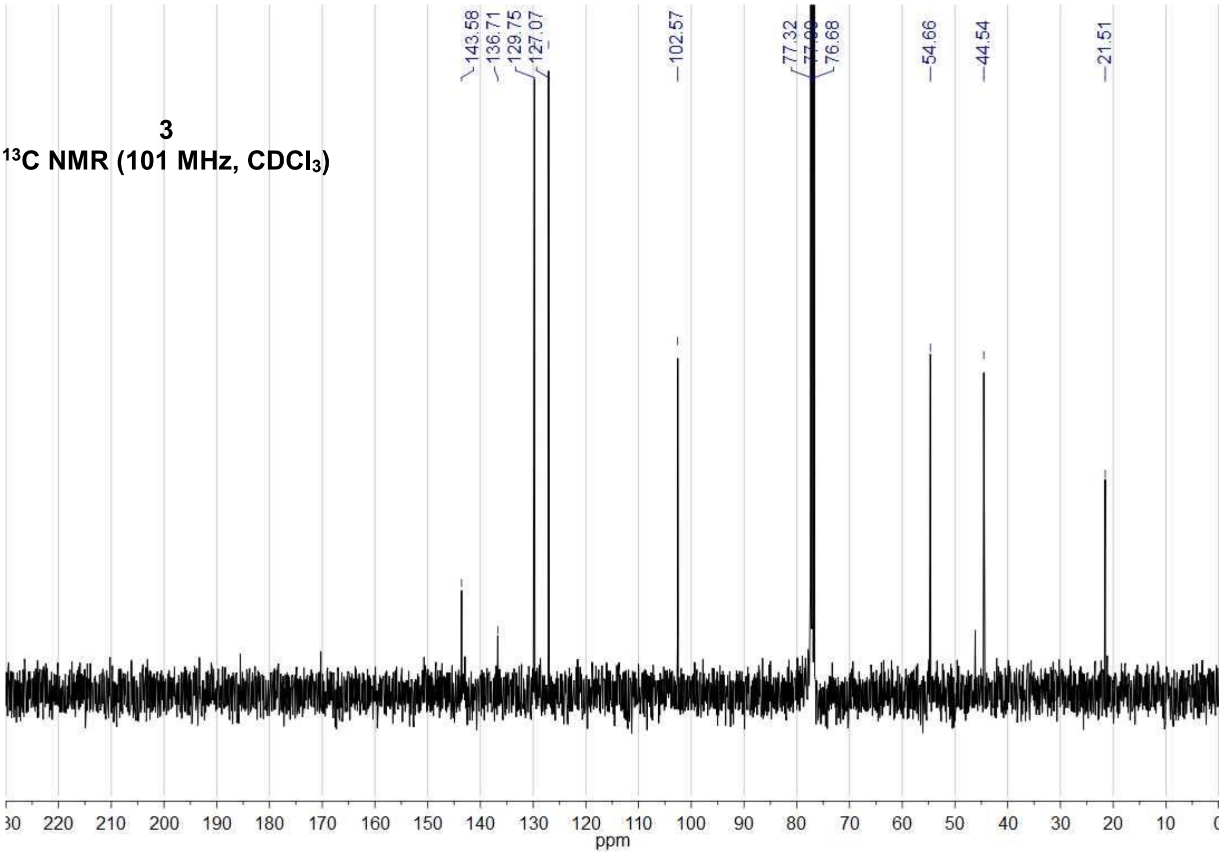

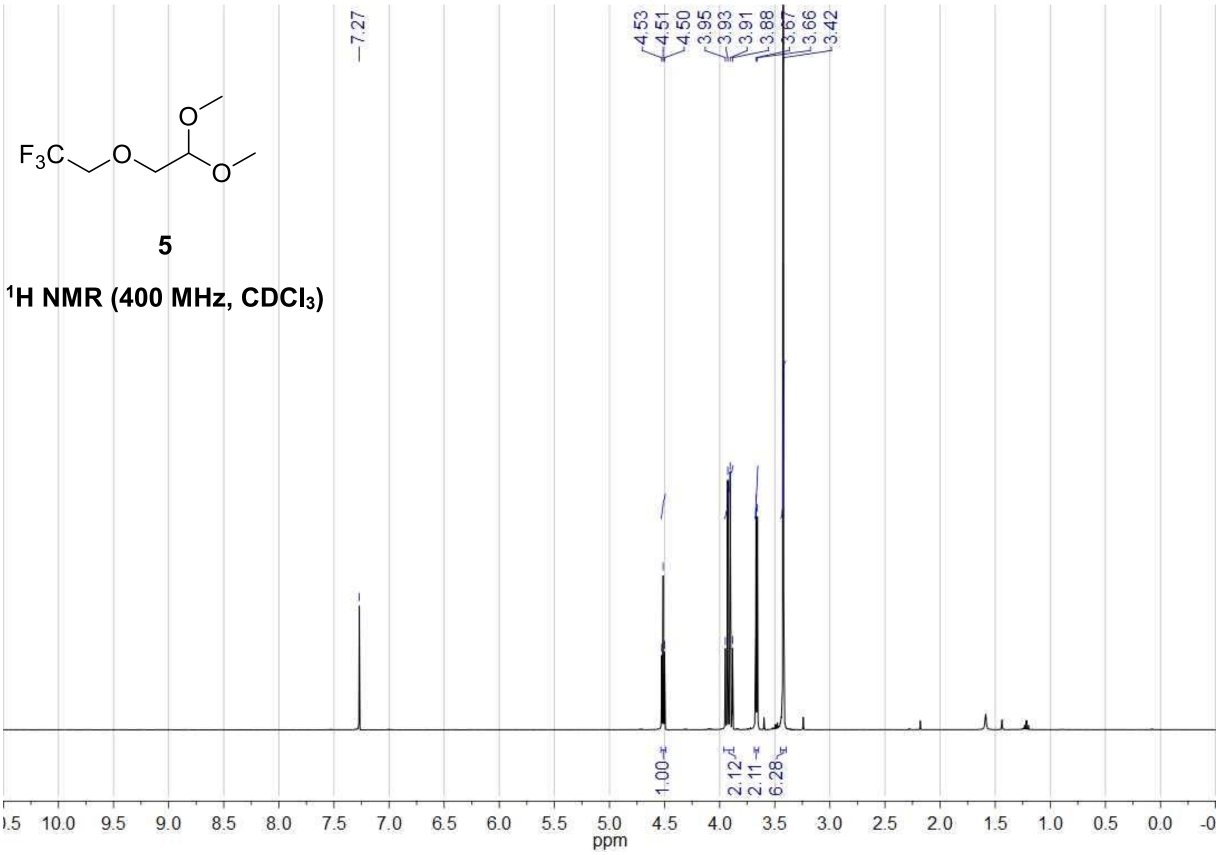

5

^{1}H NMR (400 MHz, $CDCl_3$)

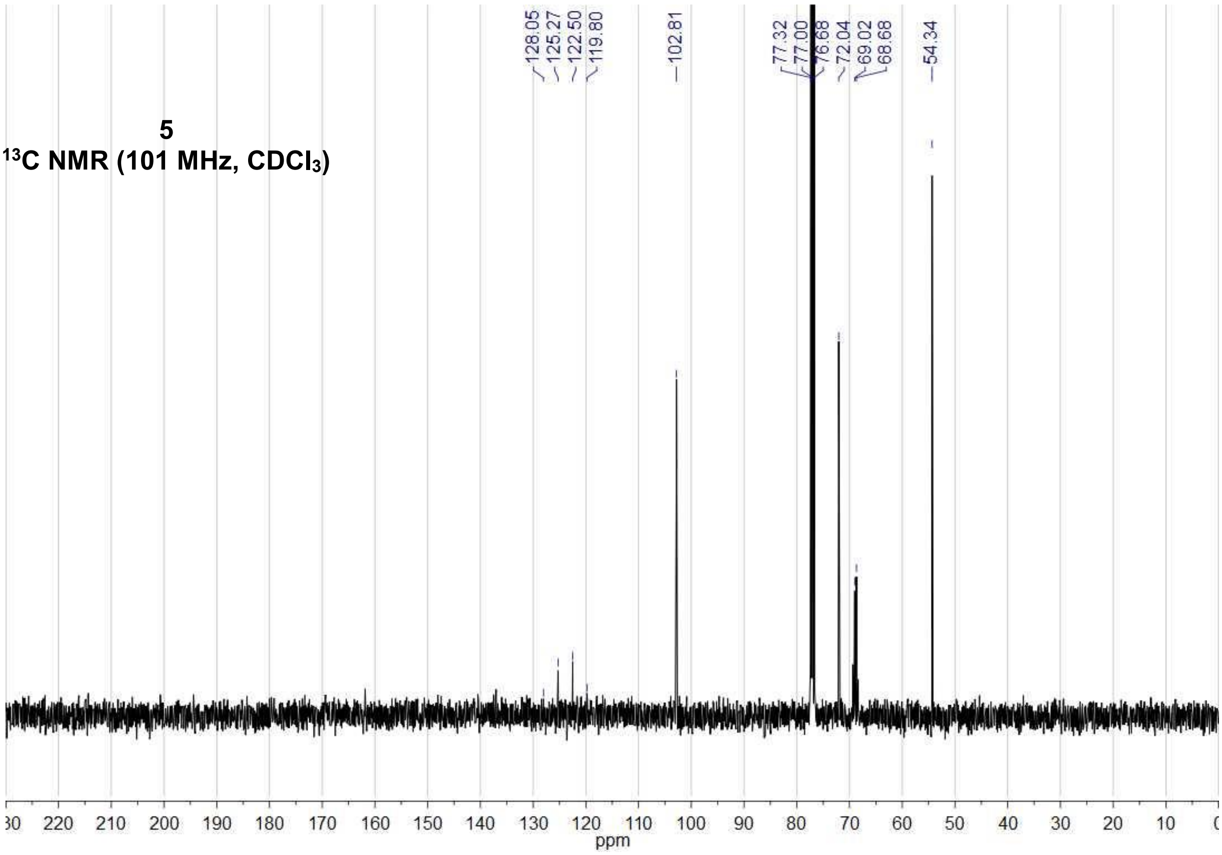

5

^{13}C NMR (101 MHz, $CDCl_3$)

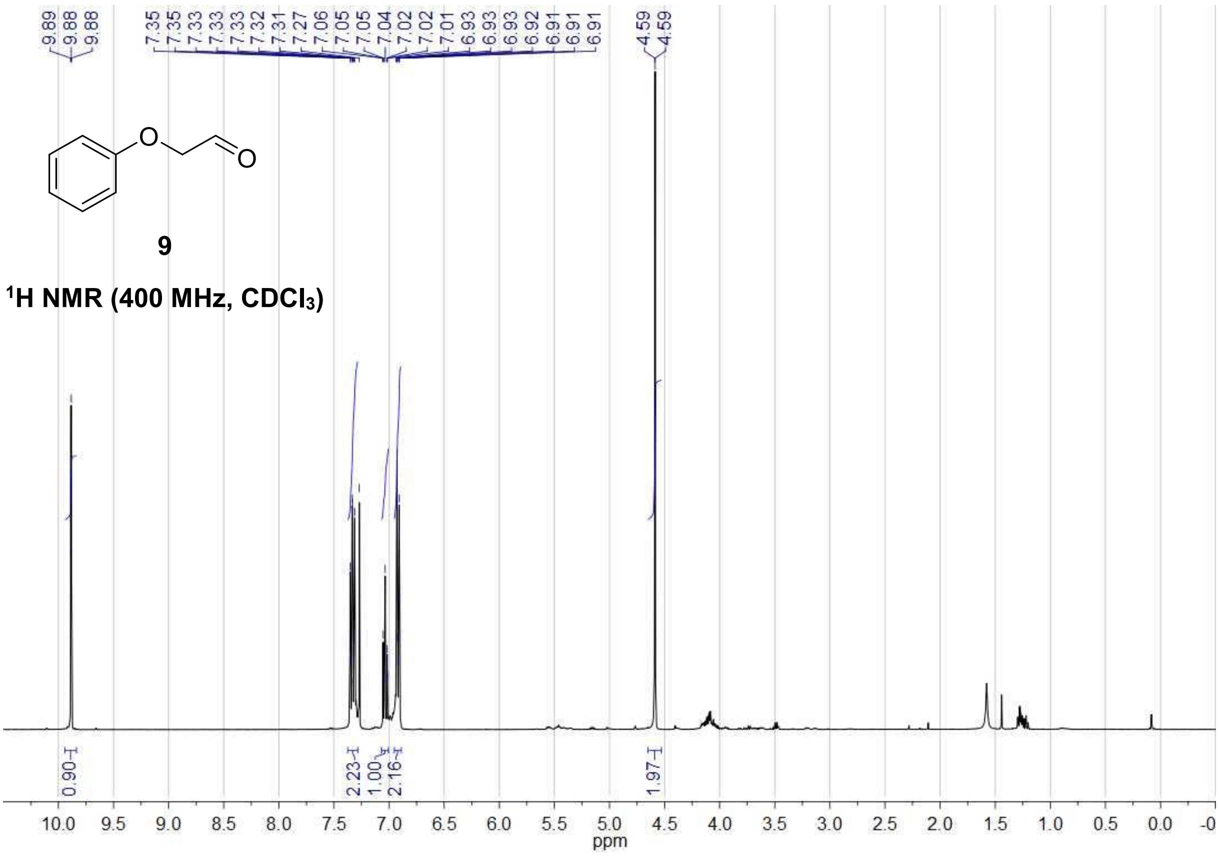
9
¹H NMR (400 MHz, CDCl₃)
9.89 9.88 9.88
7.35 7.35 7.33 7.33 7.32 7.31 7.27 7.06 7.05 7.05 7.04 7.02 7.01 6.93 6.93 6.93 6.92 6.91 6.91 6.91
4.59 4.59
0.90 2.23 1.00 2.16 1.97
10.0 9.5 9.0 8.5 8.0 7.5 7.0 6.5 6.0 5.5 5.0 4.5 4.0 3.5 3.0 2.5 2.0 1.5 1.0 0.5 0.0
ppm

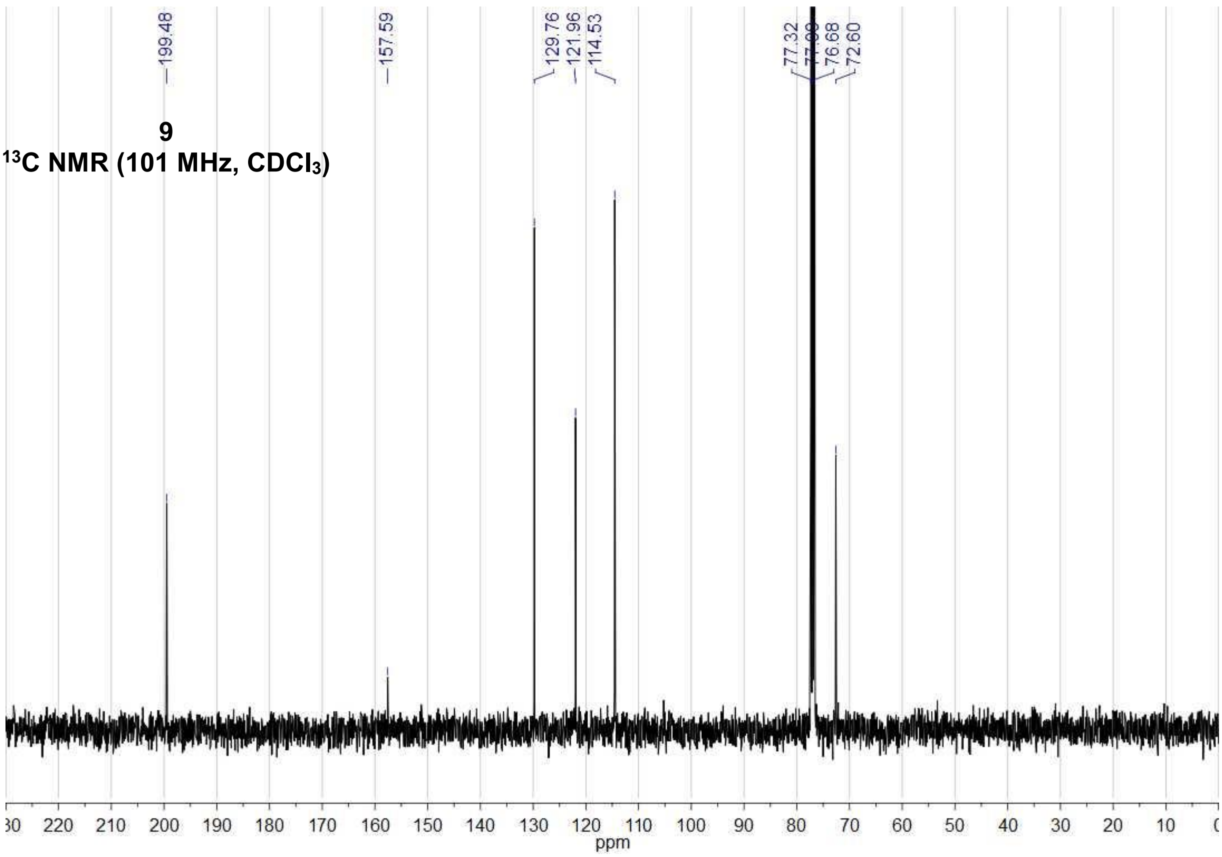
9
¹³C NMR (101 MHz, CDCl₃)
199.48
157.59
129.76 121.96 114.53
77.32 76.68 72.60
220 210 200 190 180 170 160 150 140 130 120 110 100 90 80 70 60 50 40 30 20 10
ppm

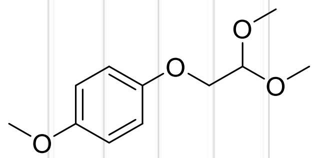

10

^{1}H NMR (400 MHz, $CDCl_3$)

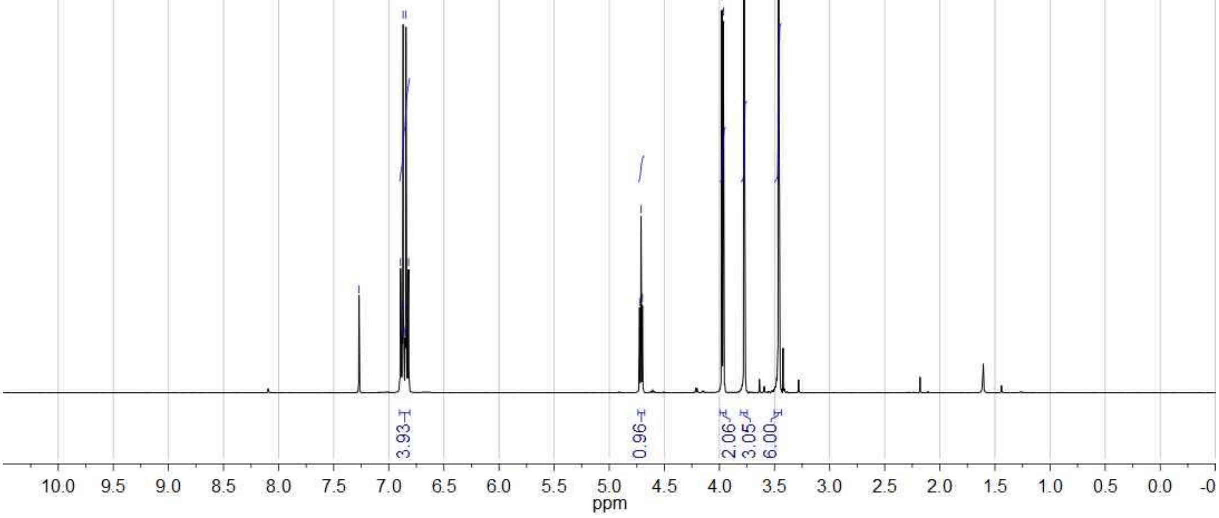

10

^{13}C NMR (101 MHz, $CDCl_3$)

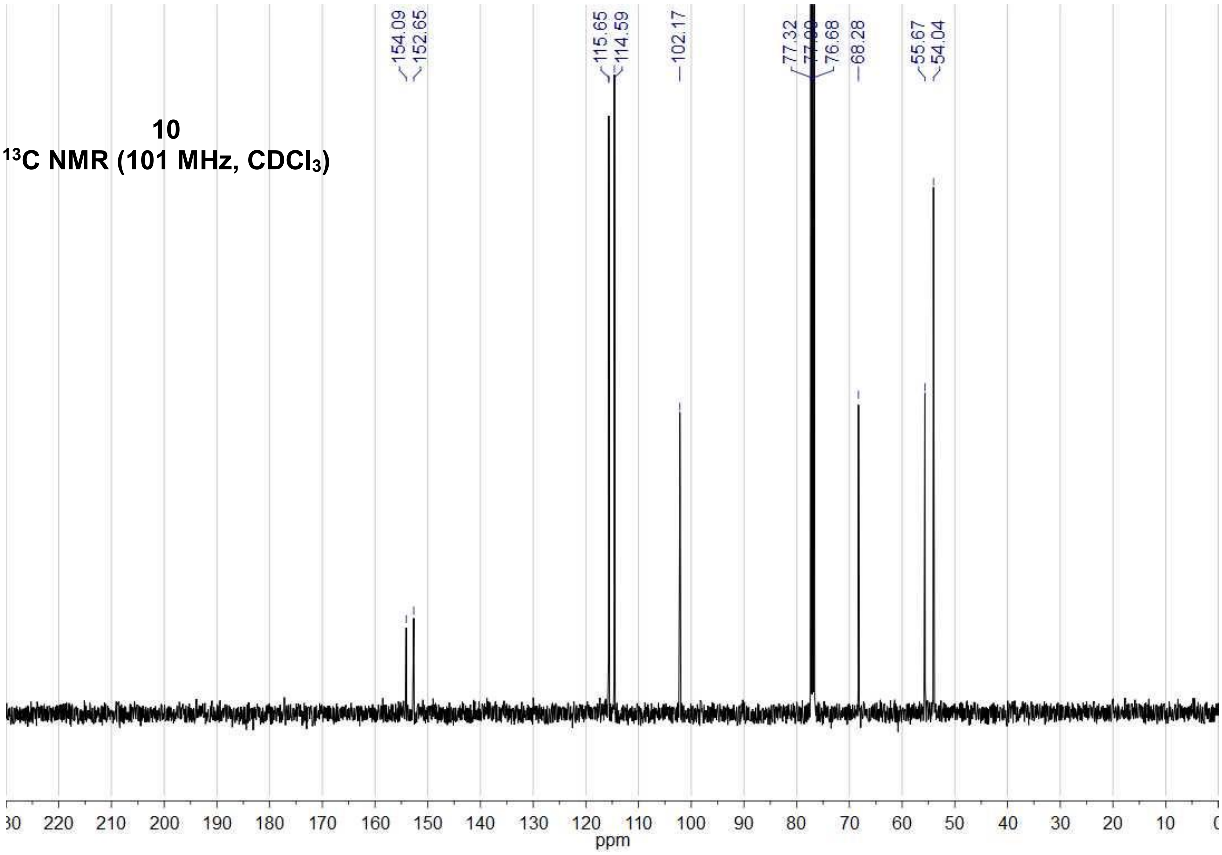

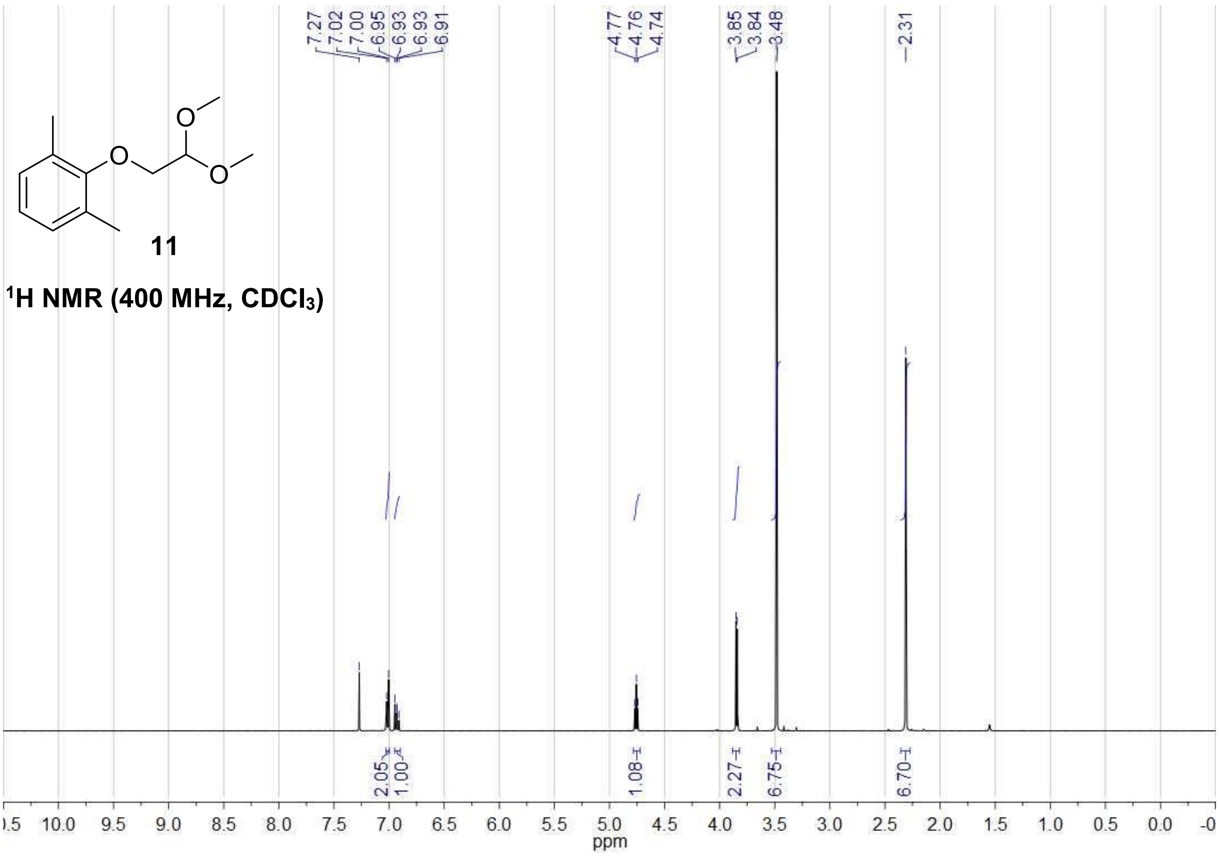

11

^{1}H NMR (400 MHz, $CDCl_3$)

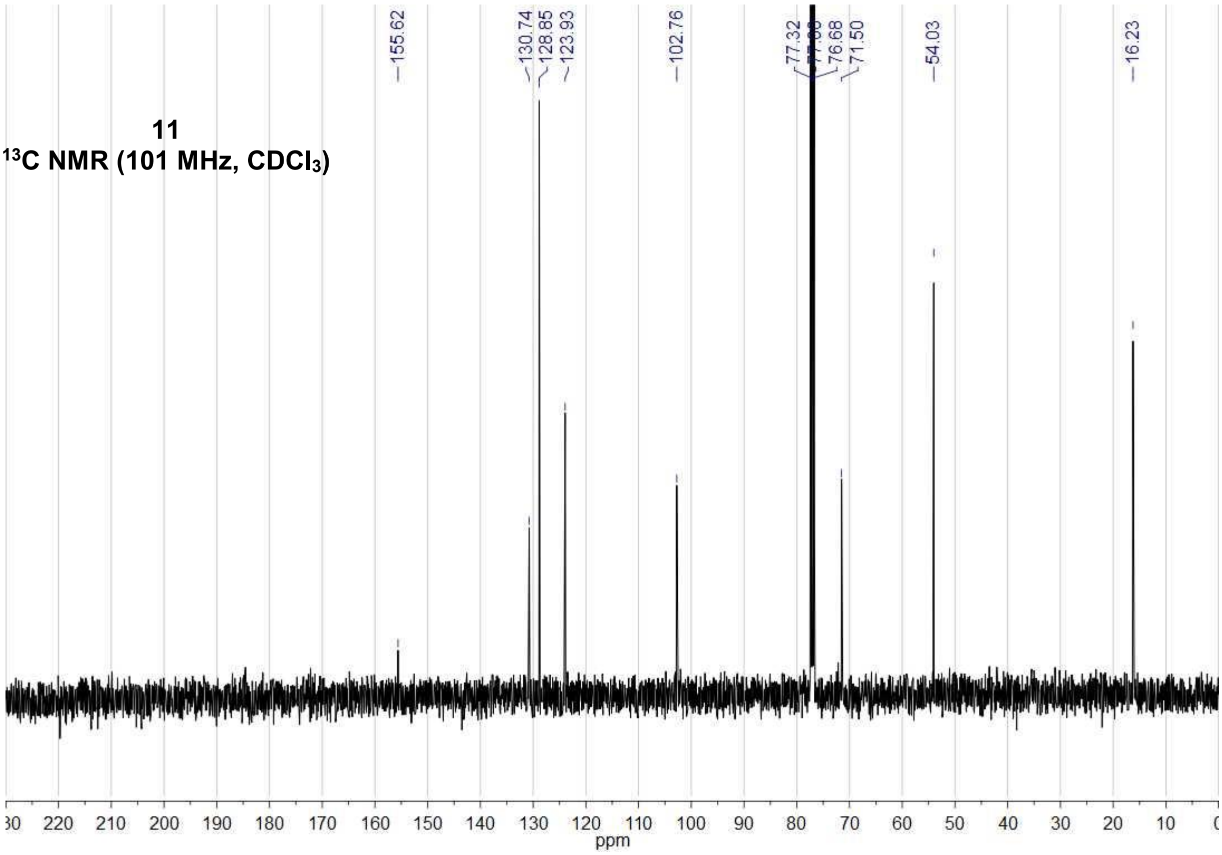

11

^{13}C NMR (101 MHz, $CDCl_3$)

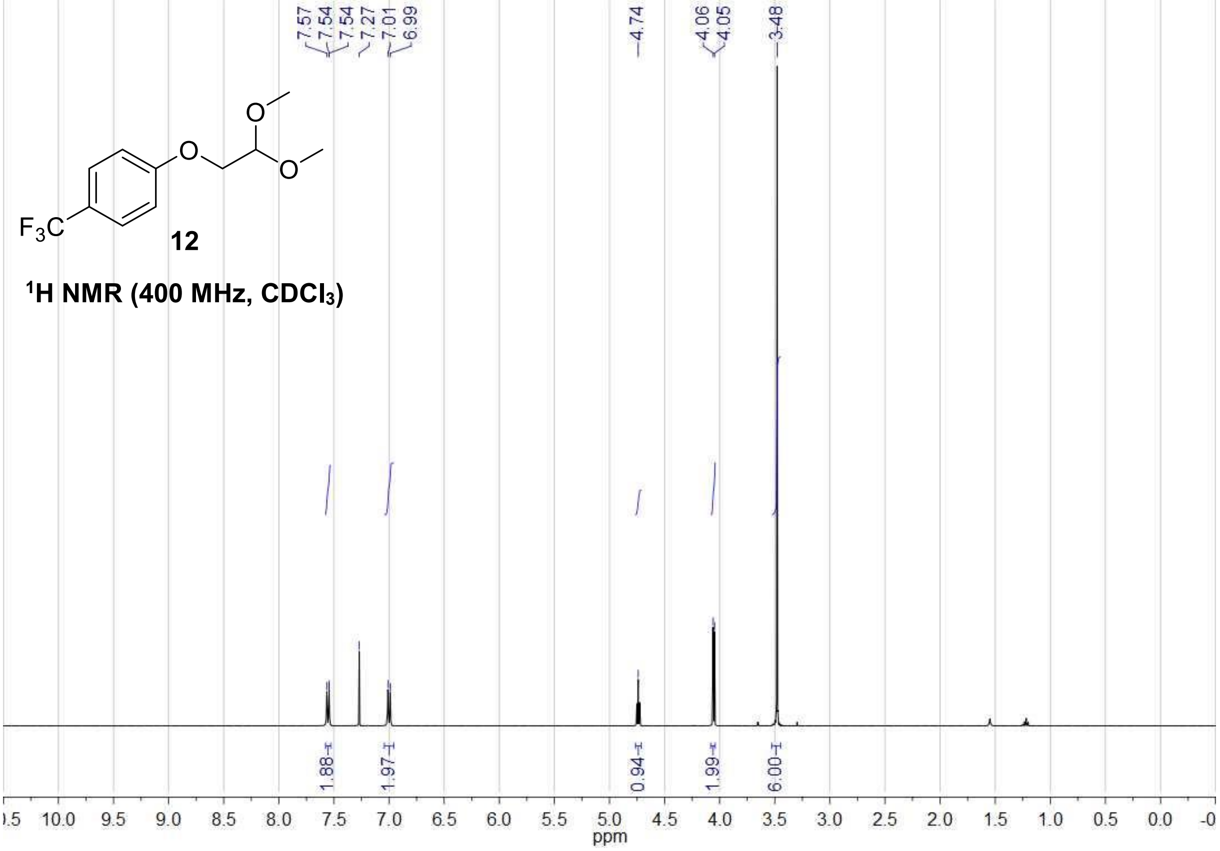
12
1H NMR (400 MHz, CDCl3)
7.57
7.54
7.54
7.27
7.01
6.99
4.74
4.06
4.05
3.48
1.88
1.97
0.94
1.99
6.00
ppm

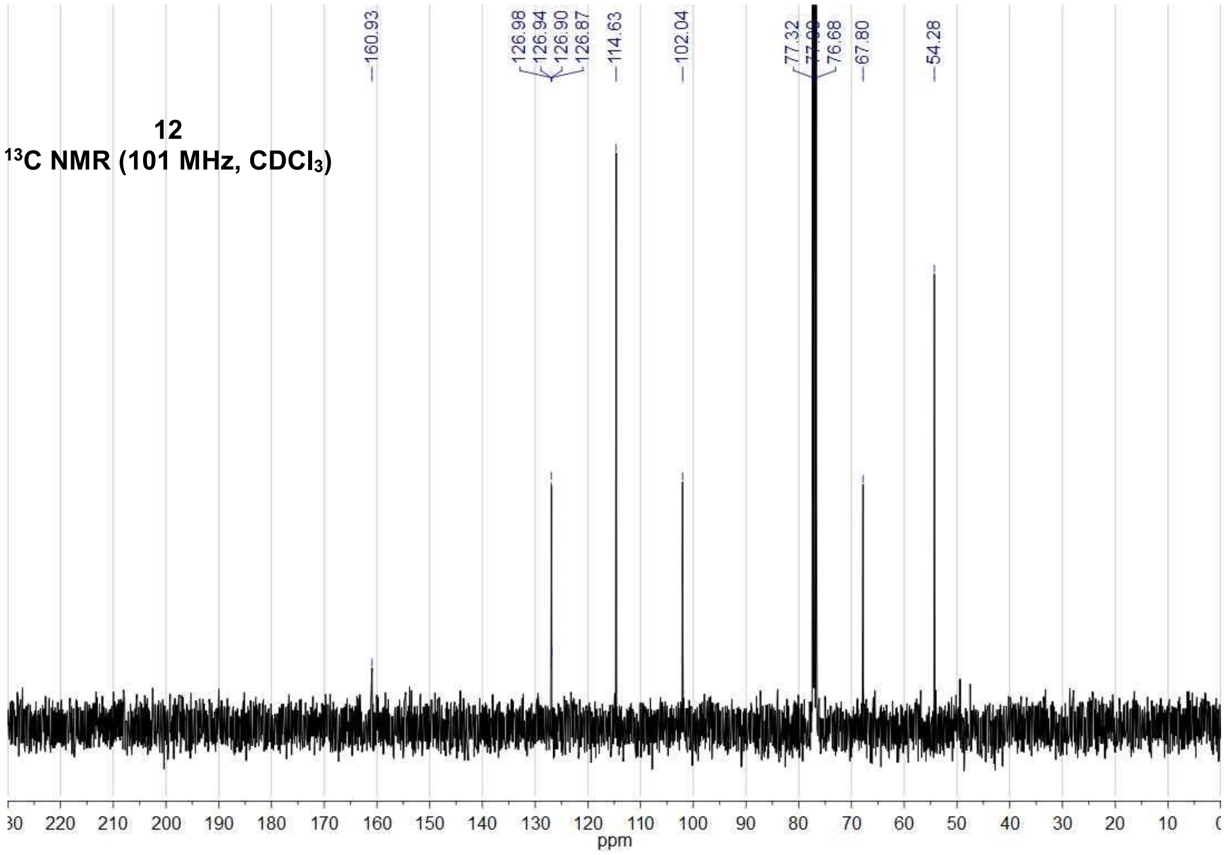
12
13C NMR (101 MHz, CDCl3)
160.93
126.98
126.94
126.90
126.87
114.63
102.04
77.32
76.68
67.80
54.28
ppm

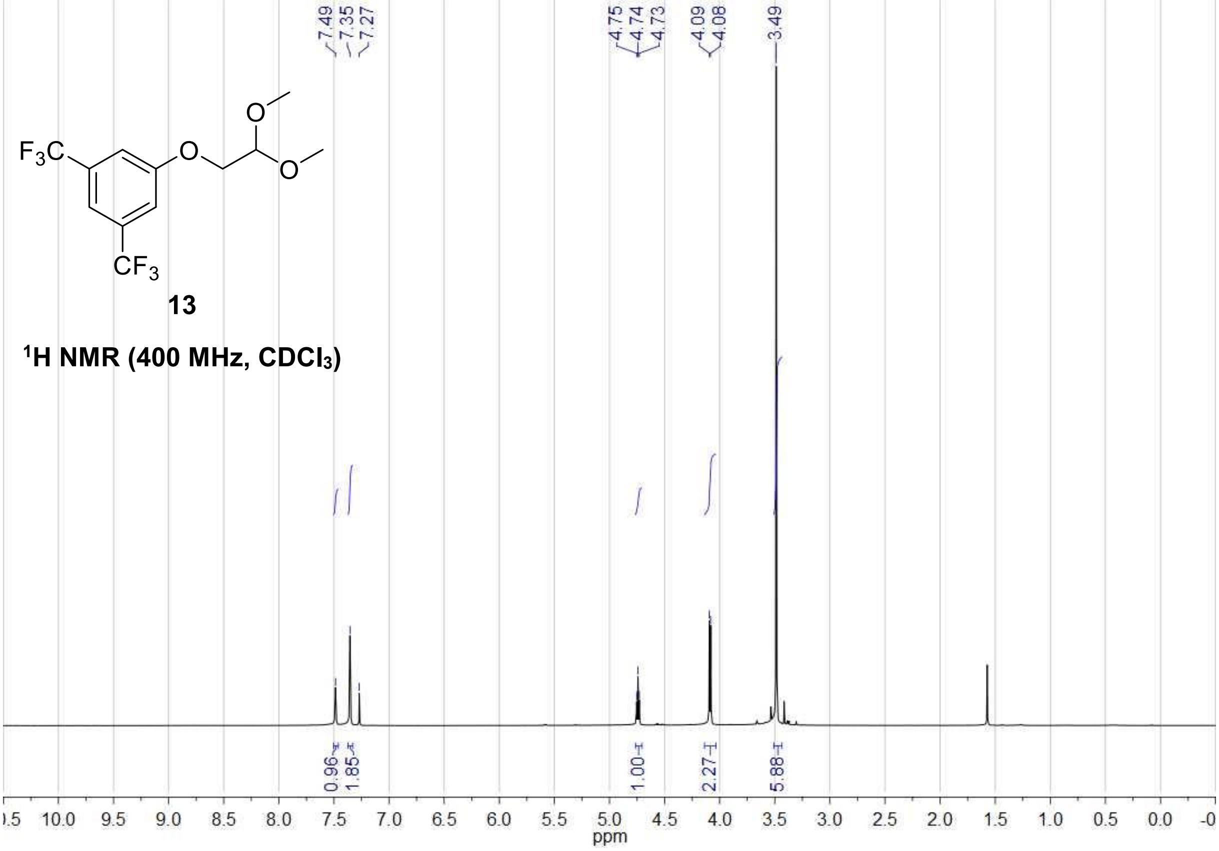
F3C
CF3
O
13
1H NMR (400 MHz, CDCl3)
7.49
7.35
7.27
4.75
4.74
4.73
4.09
4.08
3.49
0.96
1.85
1.00
2.27
5.88
ppm

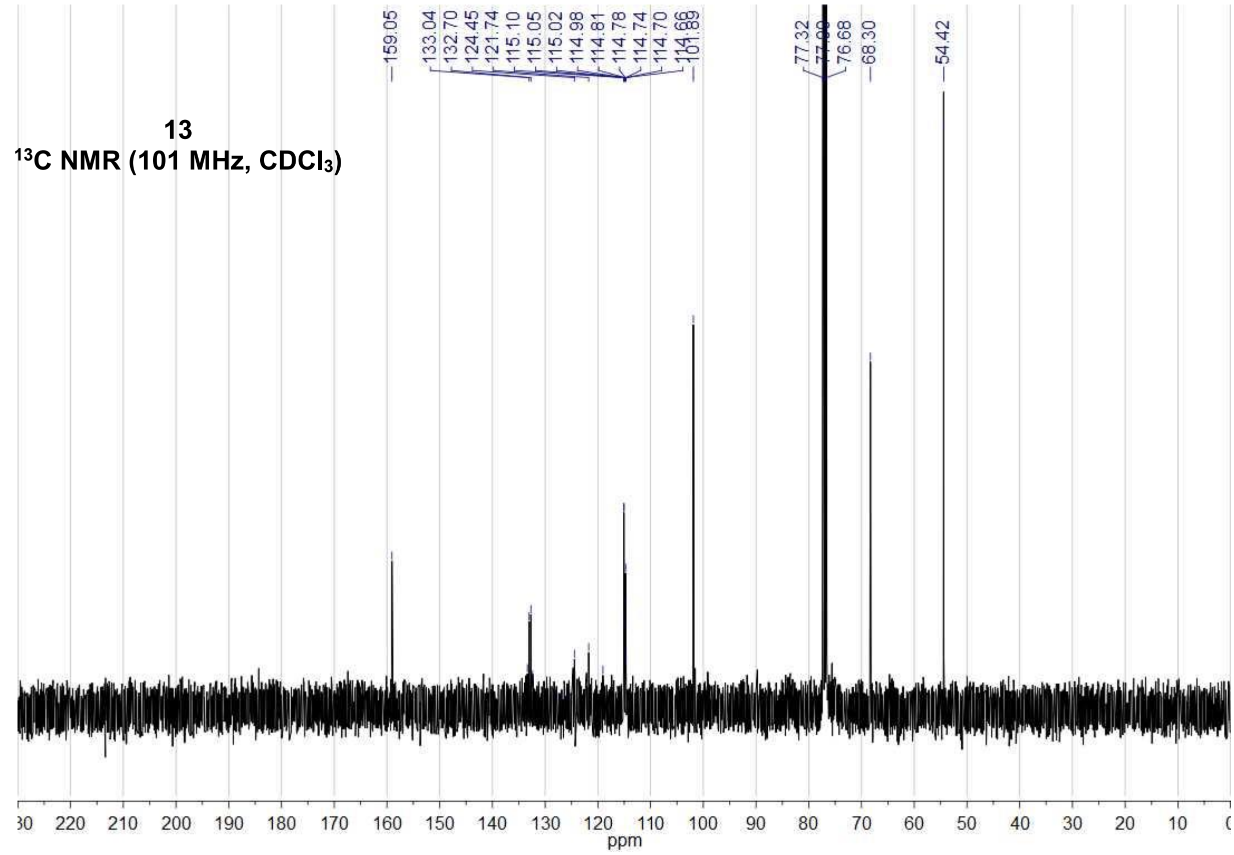
13
13C NMR (101 MHz, CDCl3)
159.05
133.04
132.70
124.45
121.74
115.10
115.05
115.02
114.98
114.81
114.78
114.74
114.70
114.66
101.89
77.32
76.68
68.30
54.42
ppm

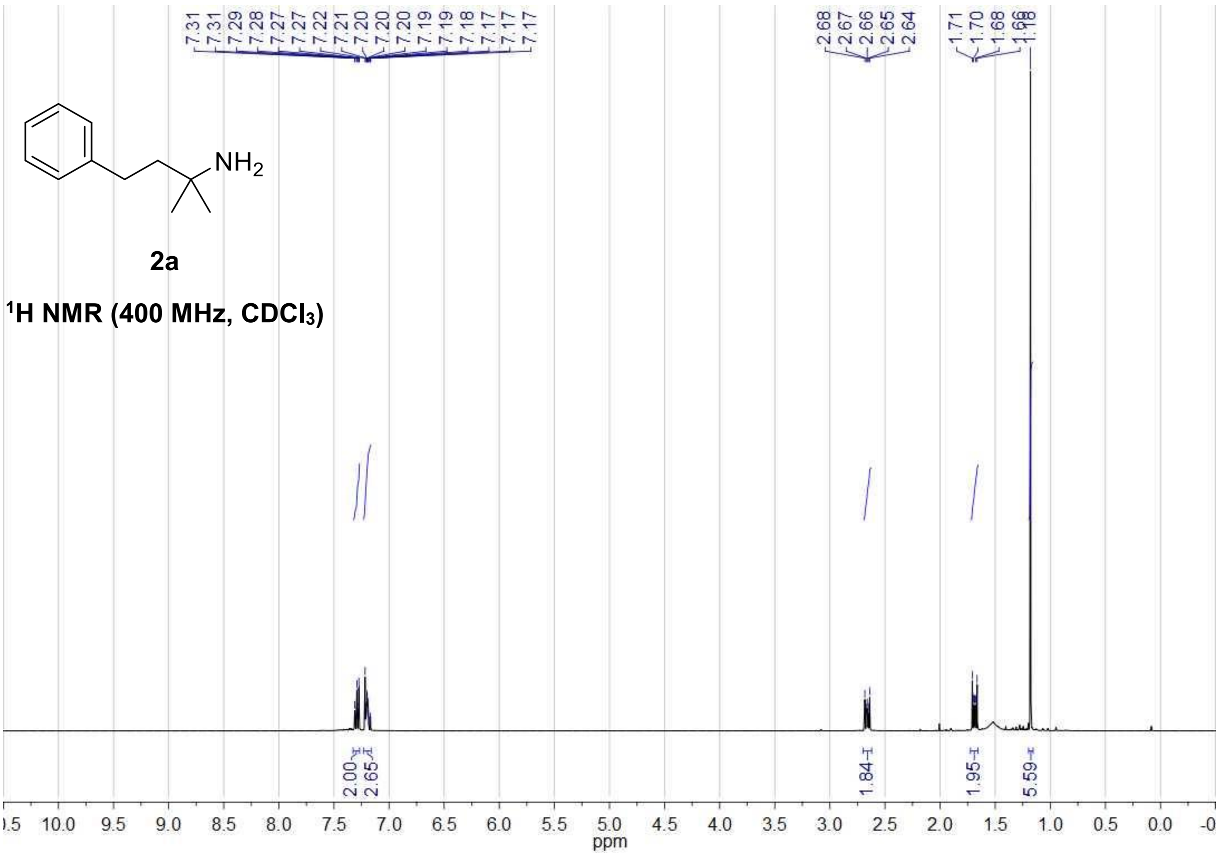

2a

^{1}H NMR (400 MHz, $CDCl_3$)

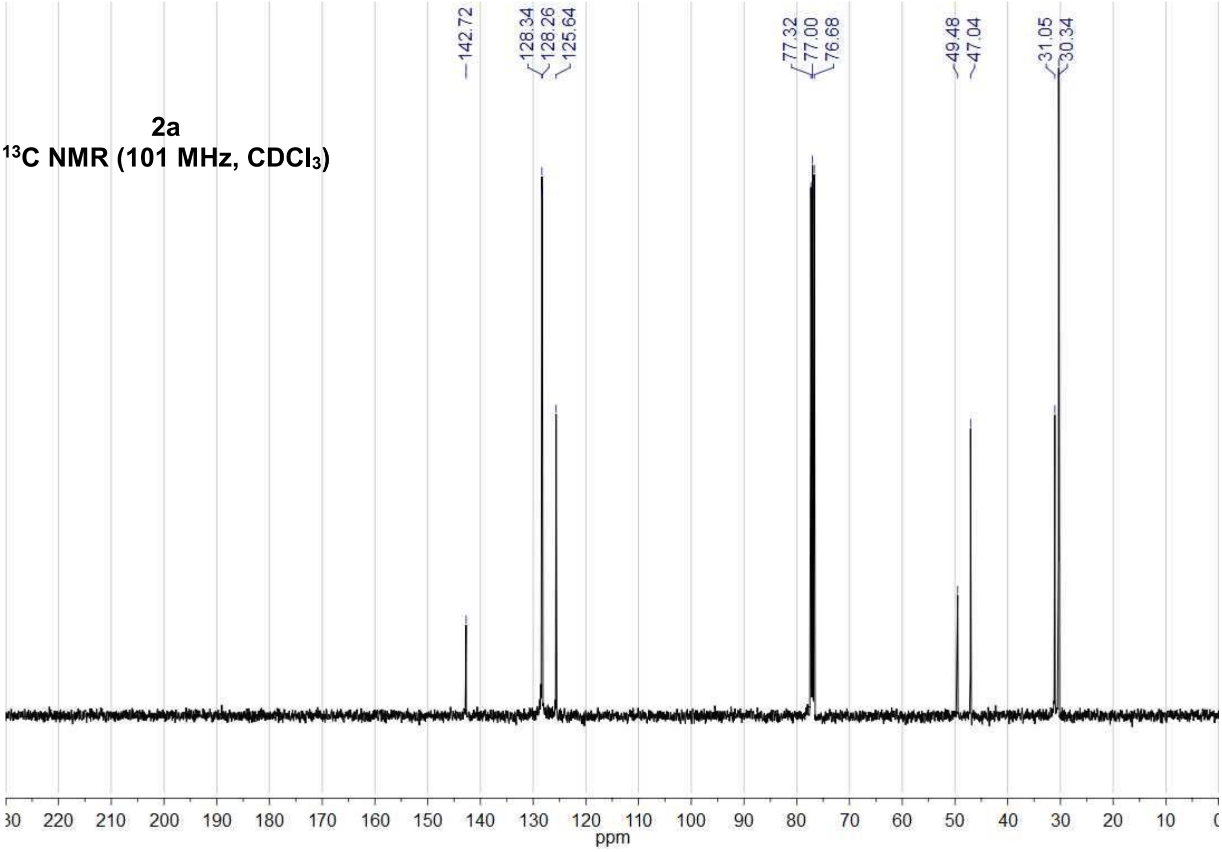

2a

^{13}C NMR (101 MHz, $CDCl_3$)

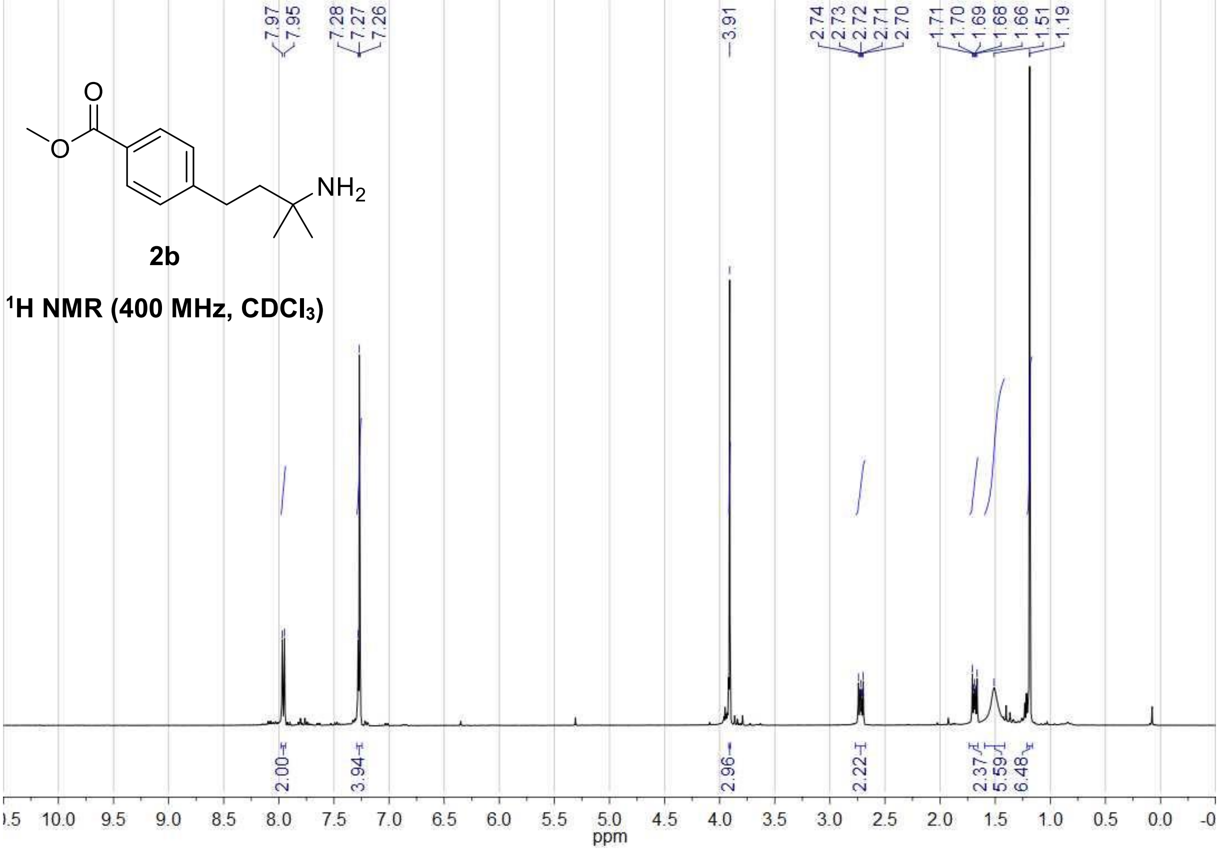

2b

^{1}H NMR (400 MHz, $CDCl_3$)

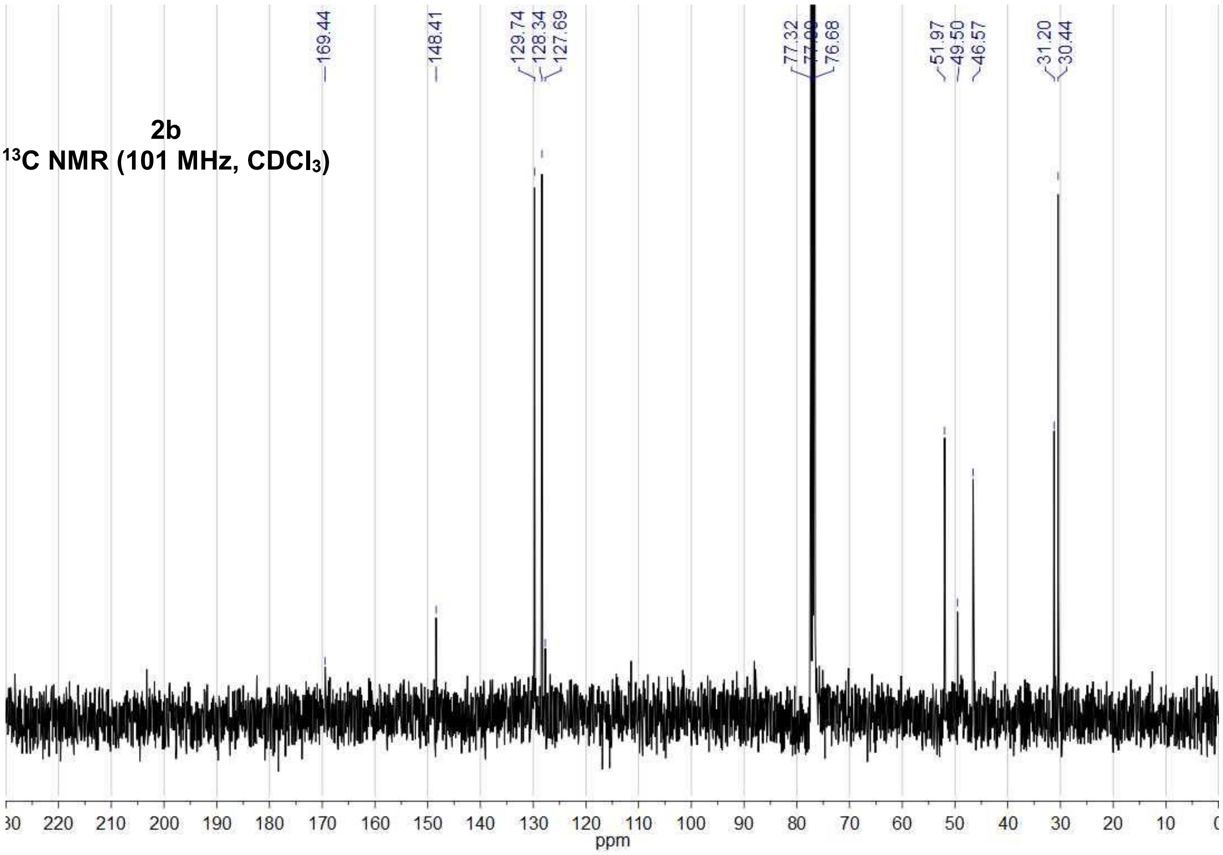

2b

^{13}C NMR (101 MHz, $CDCl_3$)

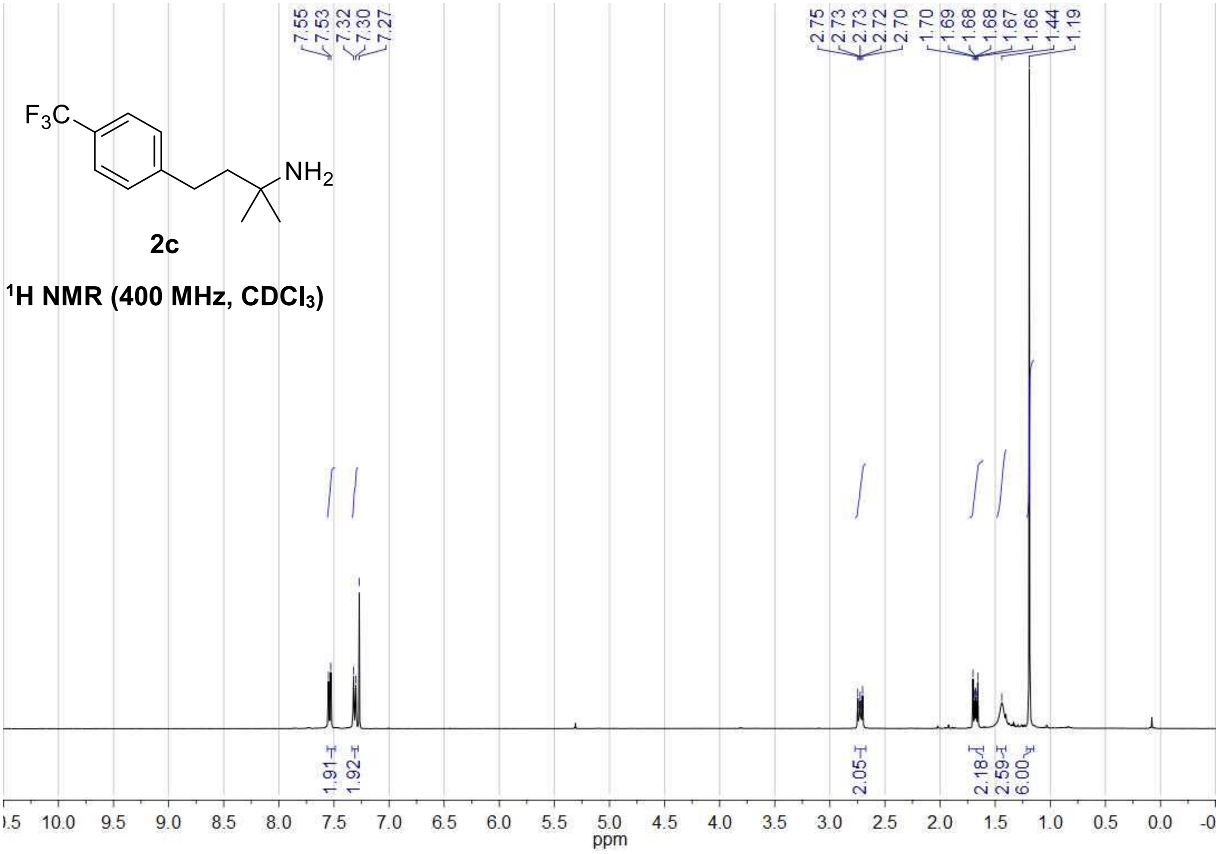
7.55 7.53 7.32 7.30 7.27
2.75 2.73 2.73 2.72 2.70 1.70 1.69 1.68 1.68 1.67 1.66 1.44 1.19
F3C
NH2
2c
1H NMR (400 MHz, CDCl3)
1.91 1.92 2.05 2.18 2.59 6.00
ppm

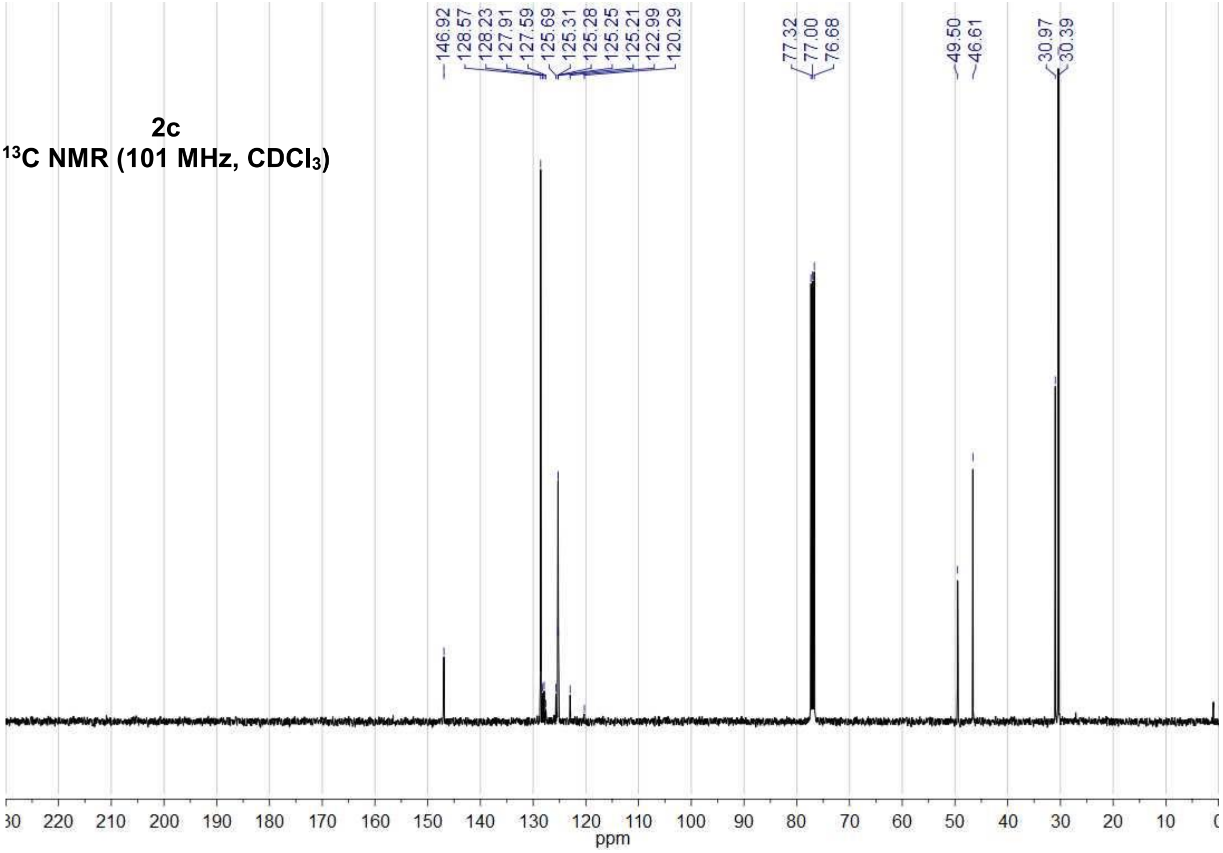
146.92 128.57 128.23 127.91 127.59 125.69 125.31 125.28 125.25 125.21 122.99 120.29
77.32 77.00 76.68
49.50 46.61
30.97 30.39
2c
13C NMR (101 MHz, CDCl3)
ppm

2d

^{1}H NMR (400 MHz, CDCl$_3$)

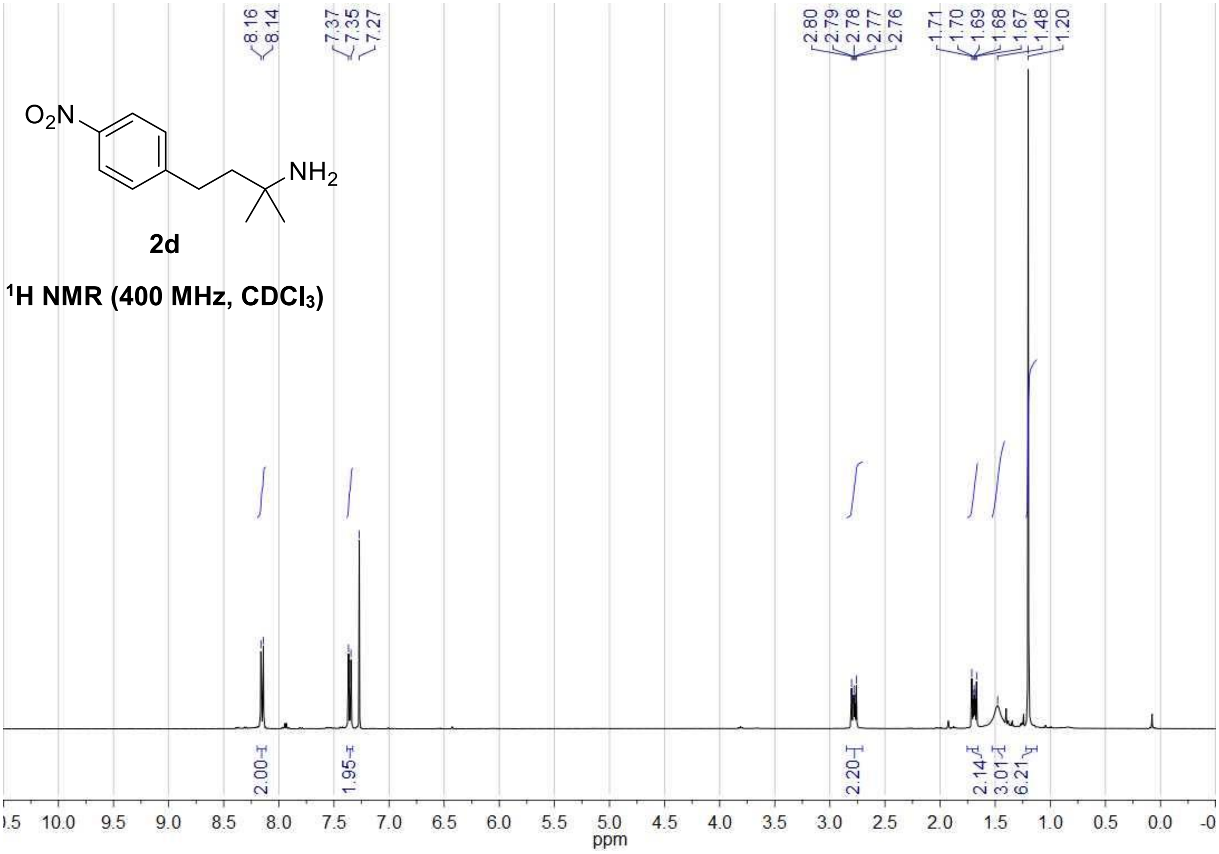

2d

^{13}C NMR (101 MHz, CDCl$_3$)

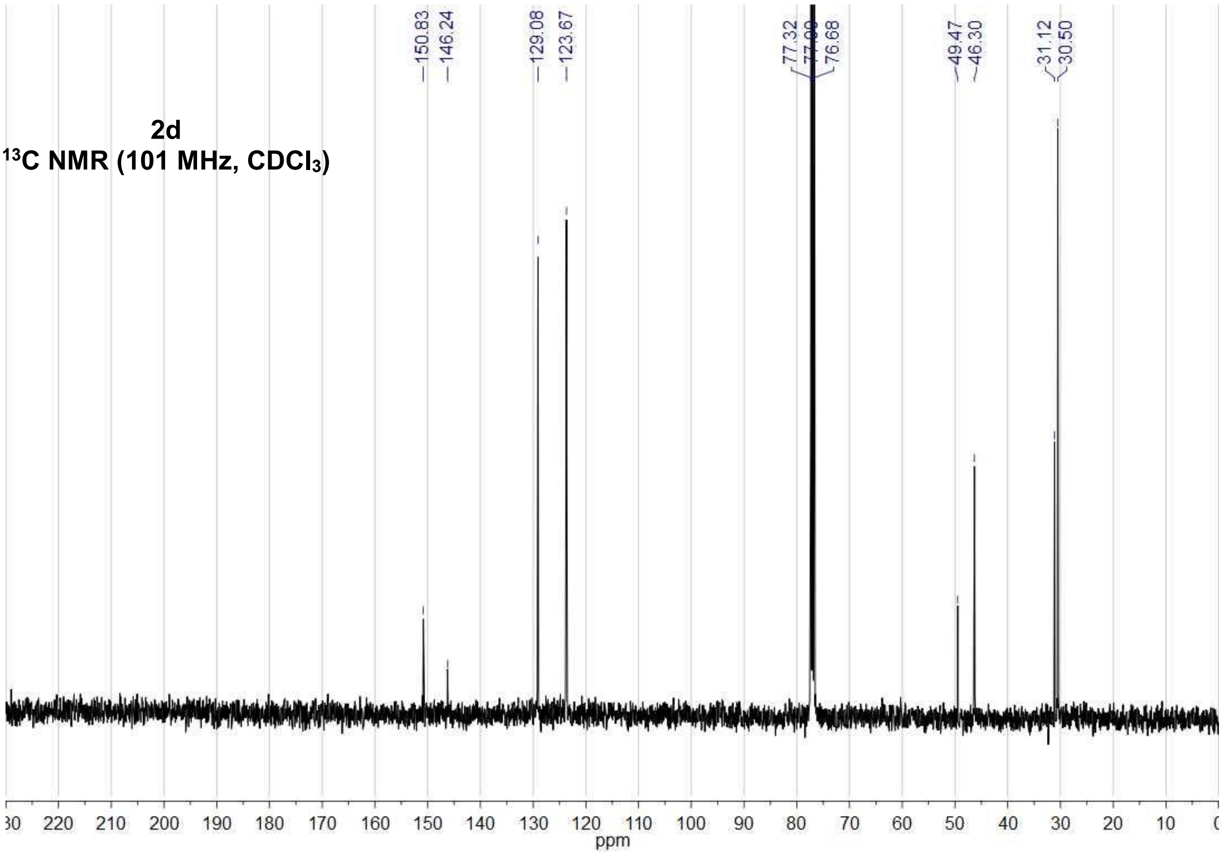

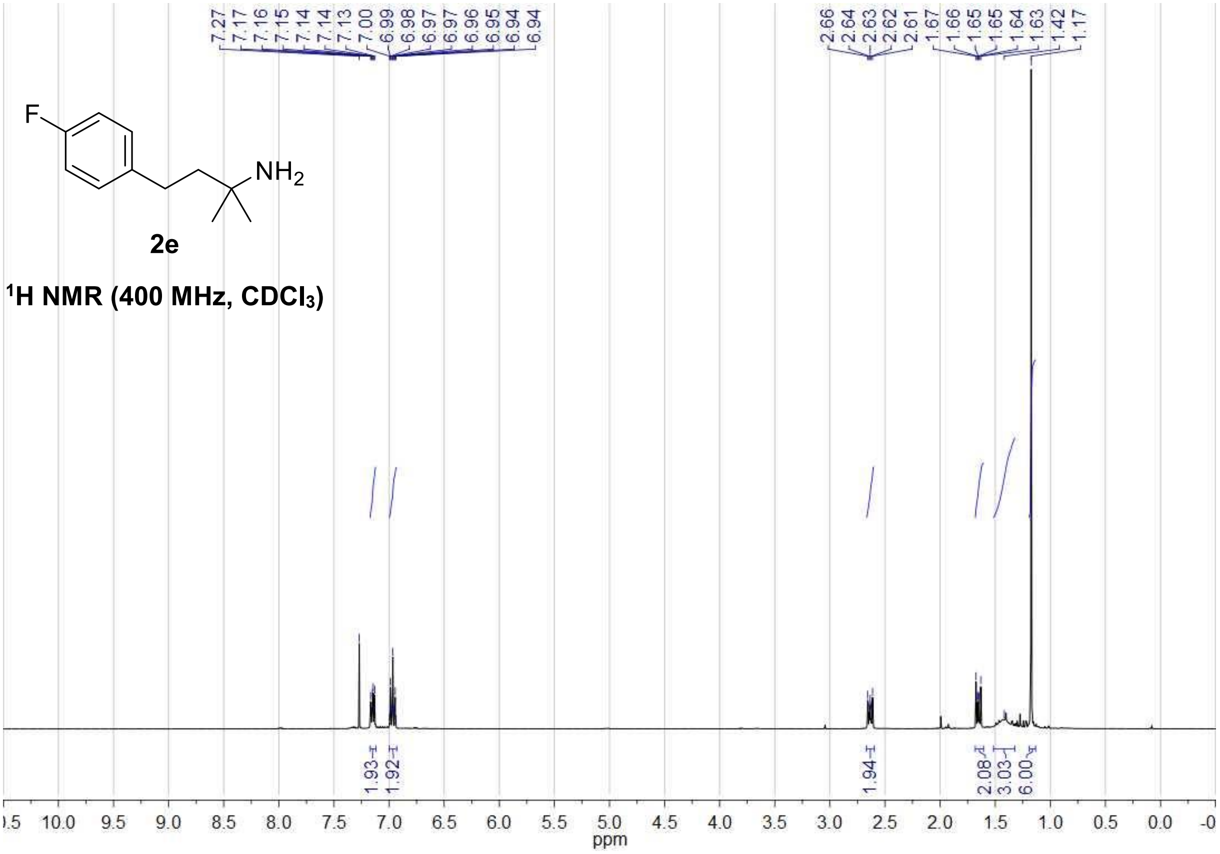
2e
^{1}H NMR (400 MHz, $CDCl_3$)
7.27 7.17 7.16 7.15 7.14 7.14 7.13 7.00 6.99 6.98 6.97 6.97 6.96 6.95 6.94 6.94
2.66 2.64 2.63 2.62 2.61 1.67 1.66 1.65 1.65 1.64 1.63 1.42 1.17
1.93 1.92 1.94 2.08 3.03 6.00
ppm

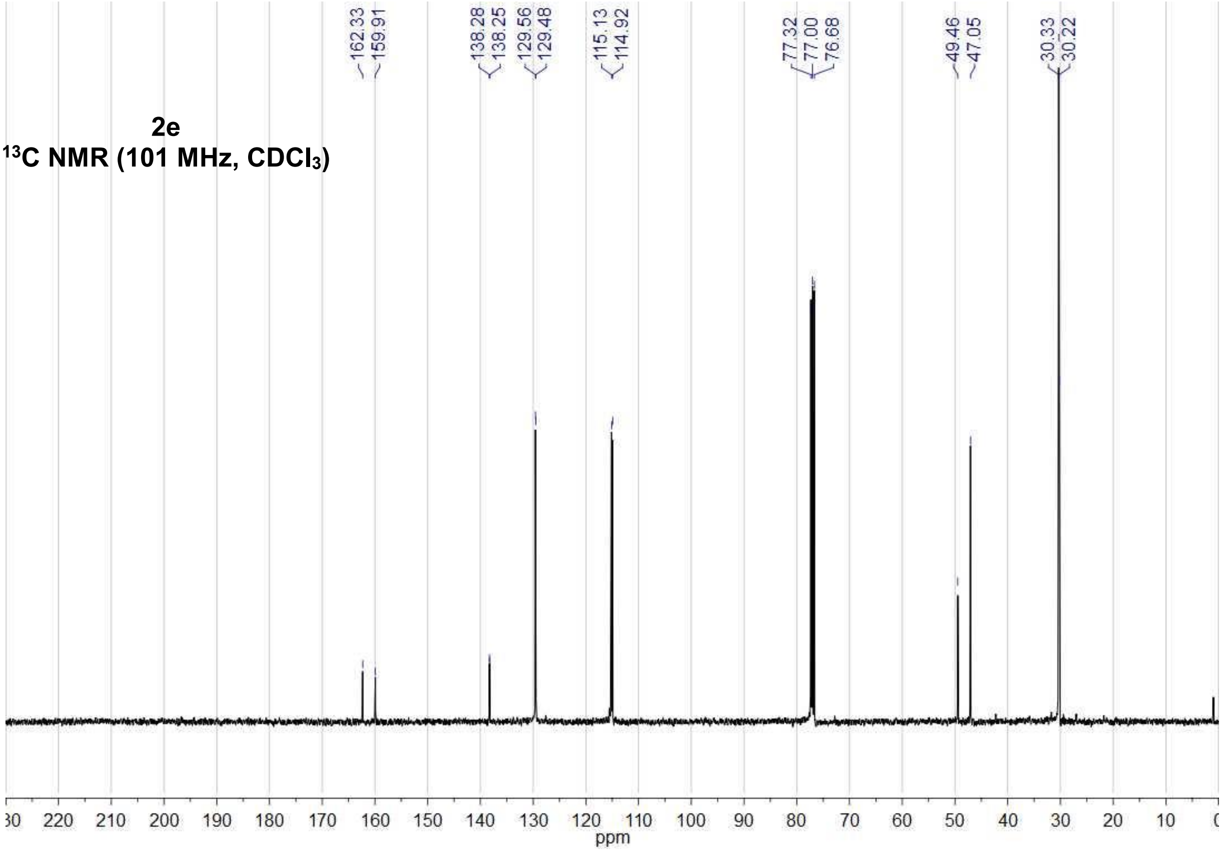
2e
^{13}C NMR (101 MHz, $CDCl_3$)
162.33 159.91 138.28 138.25 129.56 129.48 115.13 114.92 77.32 77.00 76.68 49.46 47.05 30.33 30.22
ppm

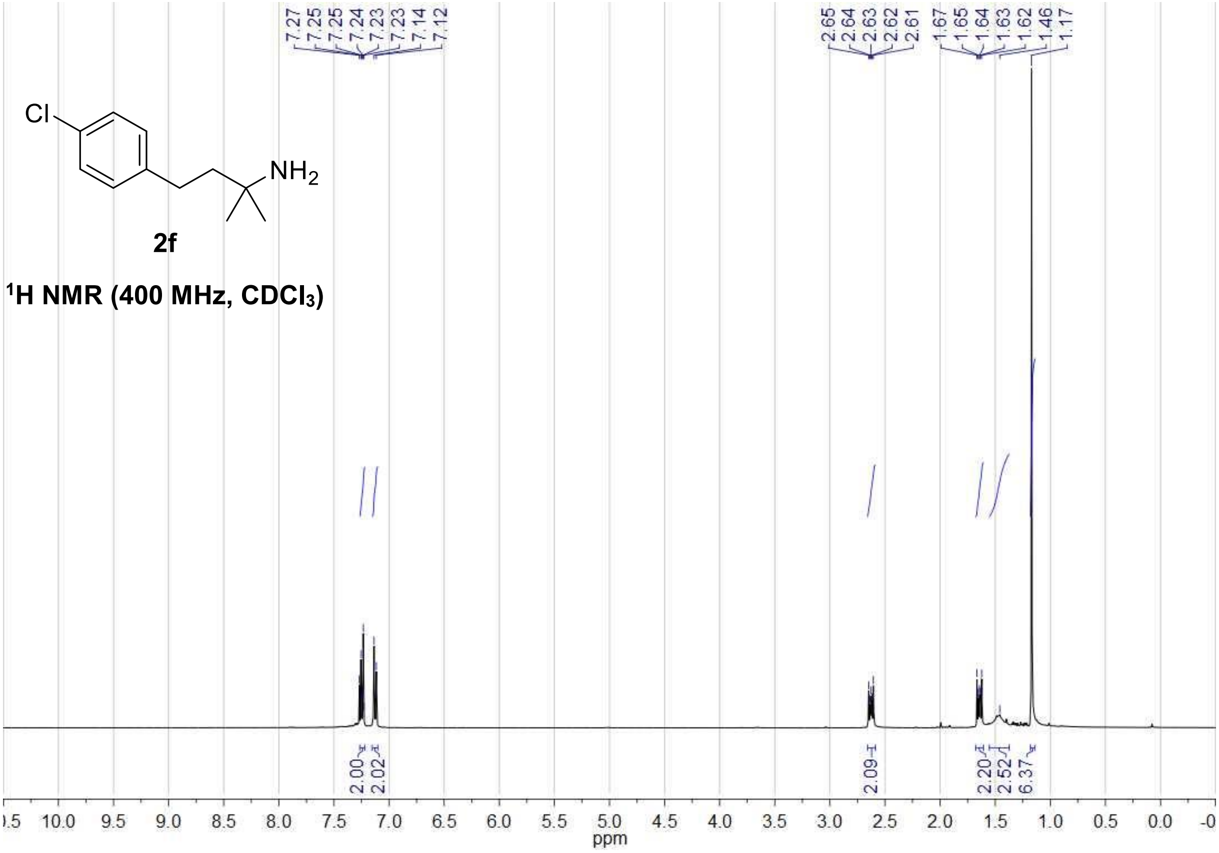
Cl
NH2
2f
1H NMR (400 MHz, CDCl3)
7.27 7.25 7.25 7.24 7.23 7.23 7.14 7.12
2.65 2.64 2.63 2.62 2.61 1.67 1.65 1.64 1.63 1.62 1.46 1.17
2.00 2.02 2.09 2.20 2.52 6.37
10.0 9.5 9.0 8.5 8.0 7.5 7.0 6.5 6.0 5.5 5.0 4.5 4.0 3.5 3.0 2.5 2.0 1.5 1.0 0.5 0.0
ppm

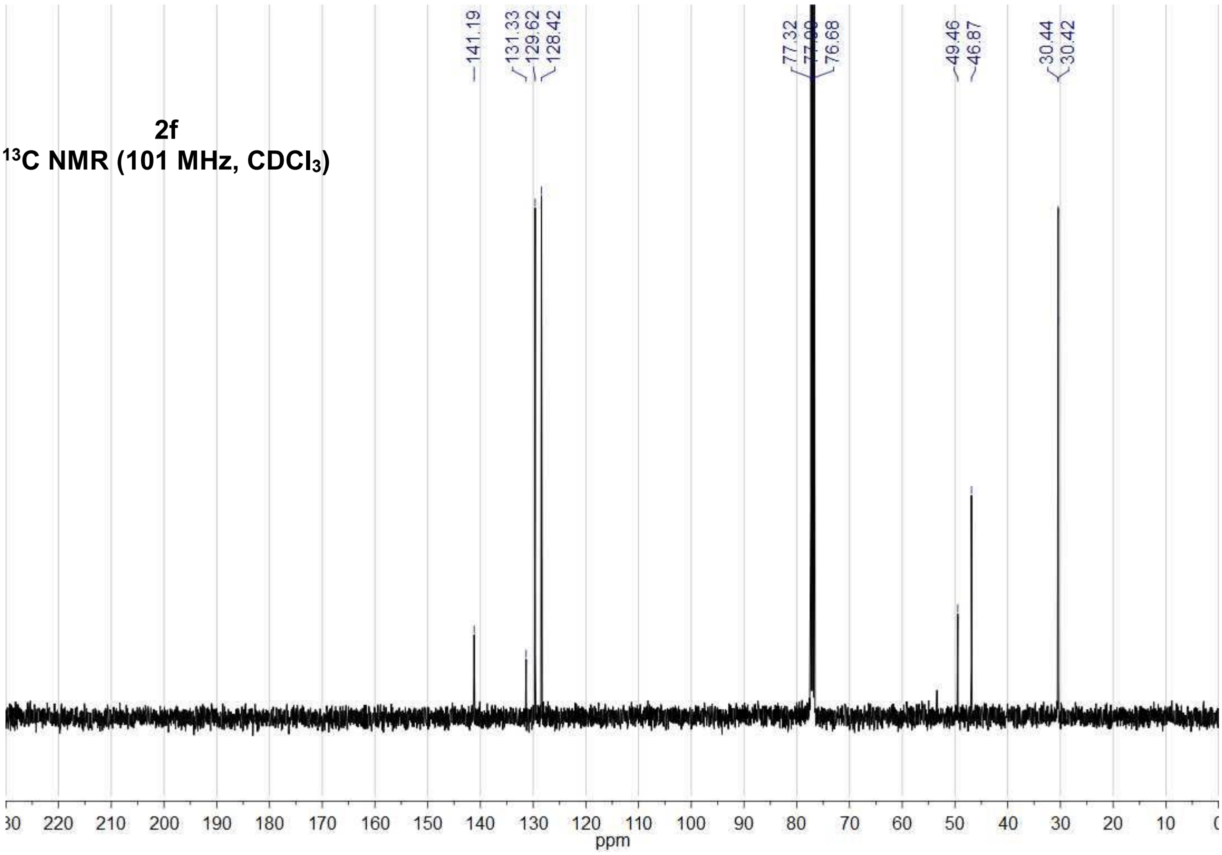
2f
13C NMR (101 MHz, CDCl3)
141.19
131.33 129.62 128.42
77.32 76.68
49.46 46.87
30.44 30.42
220 210 200 190 180 170 160 150 140 130 120 110 100 90 80 70 60 50 40 30 20 10
ppm

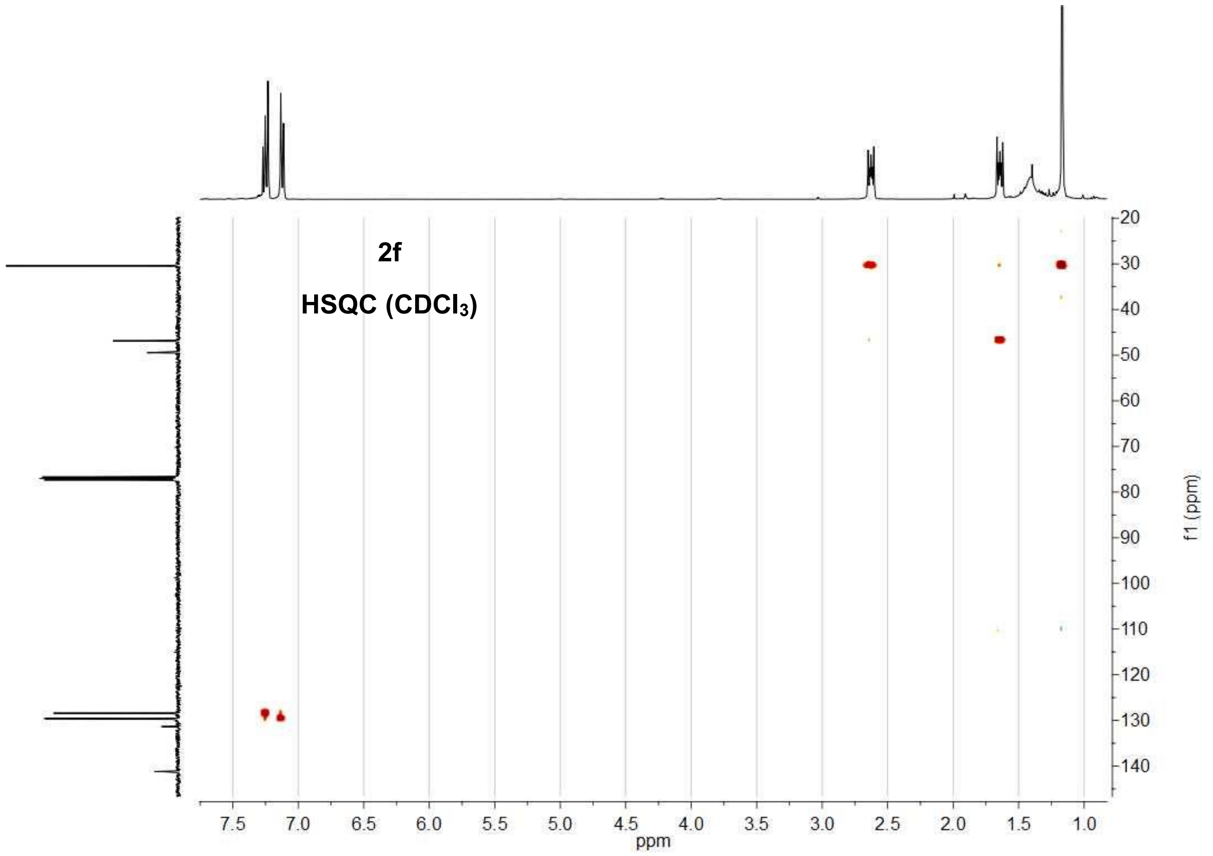
2f
HSQC (CDCl3)
ppm
f1 (ppm)

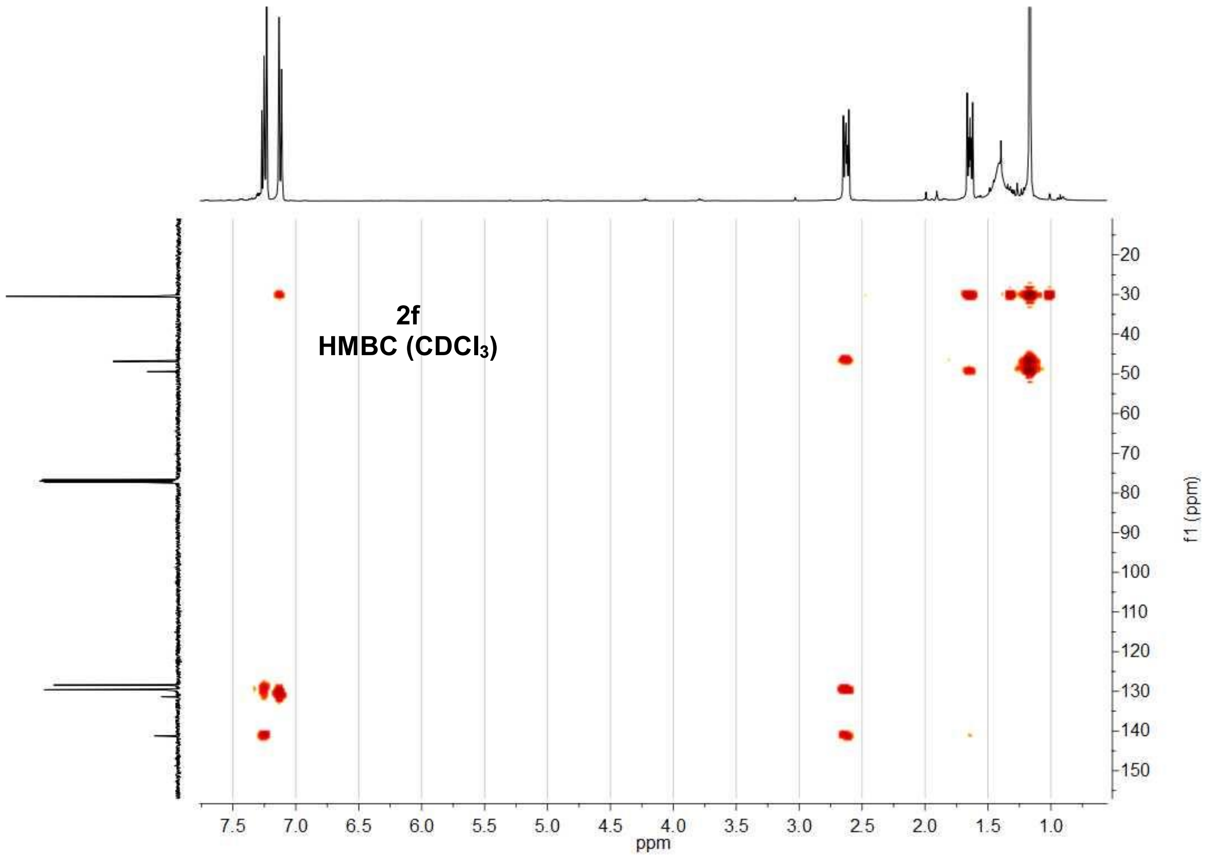
2f
HMBC (CDCl3)
ppm
f1 (ppm)

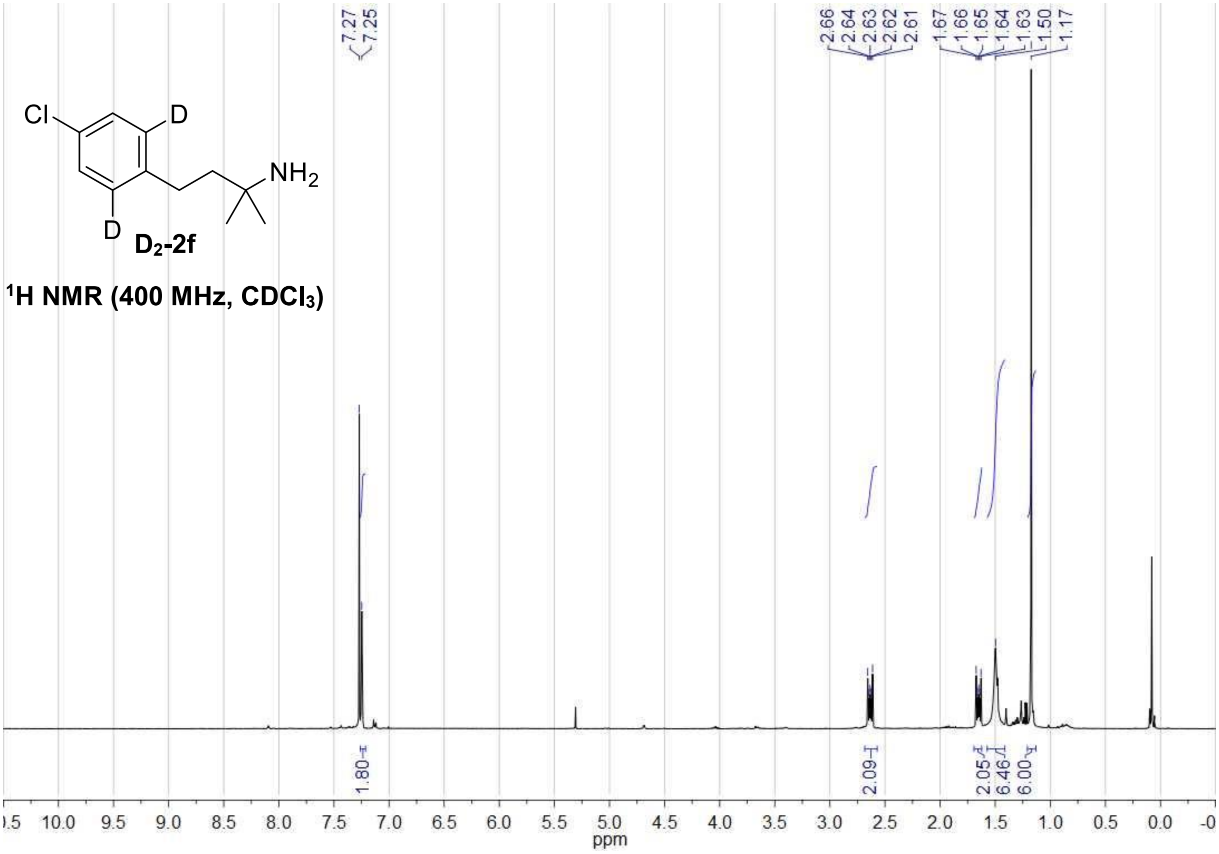
Cl
D
NH2
D
D2-2f
1H NMR (400 MHz, CDCl3)
7.27
7.25
2.66
2.64
2.63
2.62
2.61
1.67
1.66
1.65
1.64
1.63
1.50
1.17
1.80
2.09
2.05
6.46
6.00
ppm

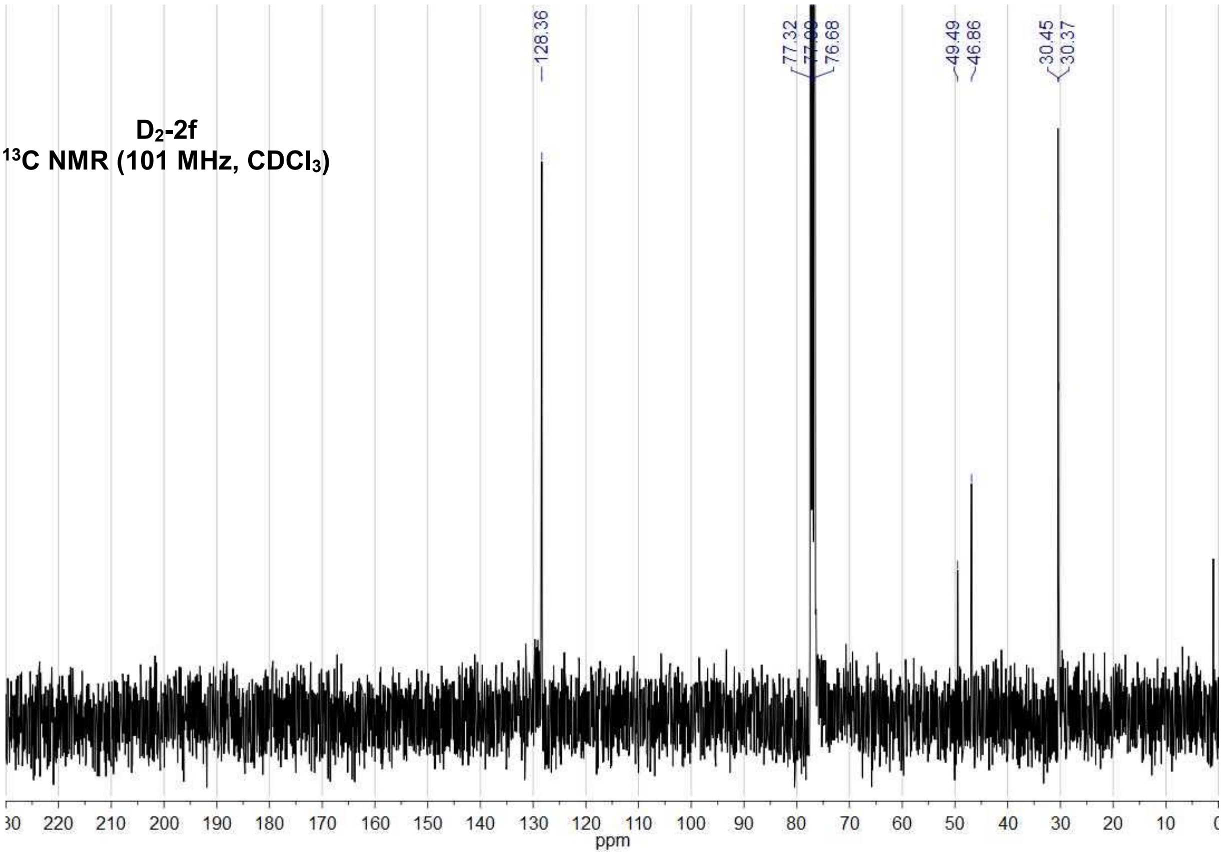
D2-2f
13C NMR (101 MHz, CDCl3)
128.36
77.32
76.68
49.49
46.86
30.45
30.37
ppm

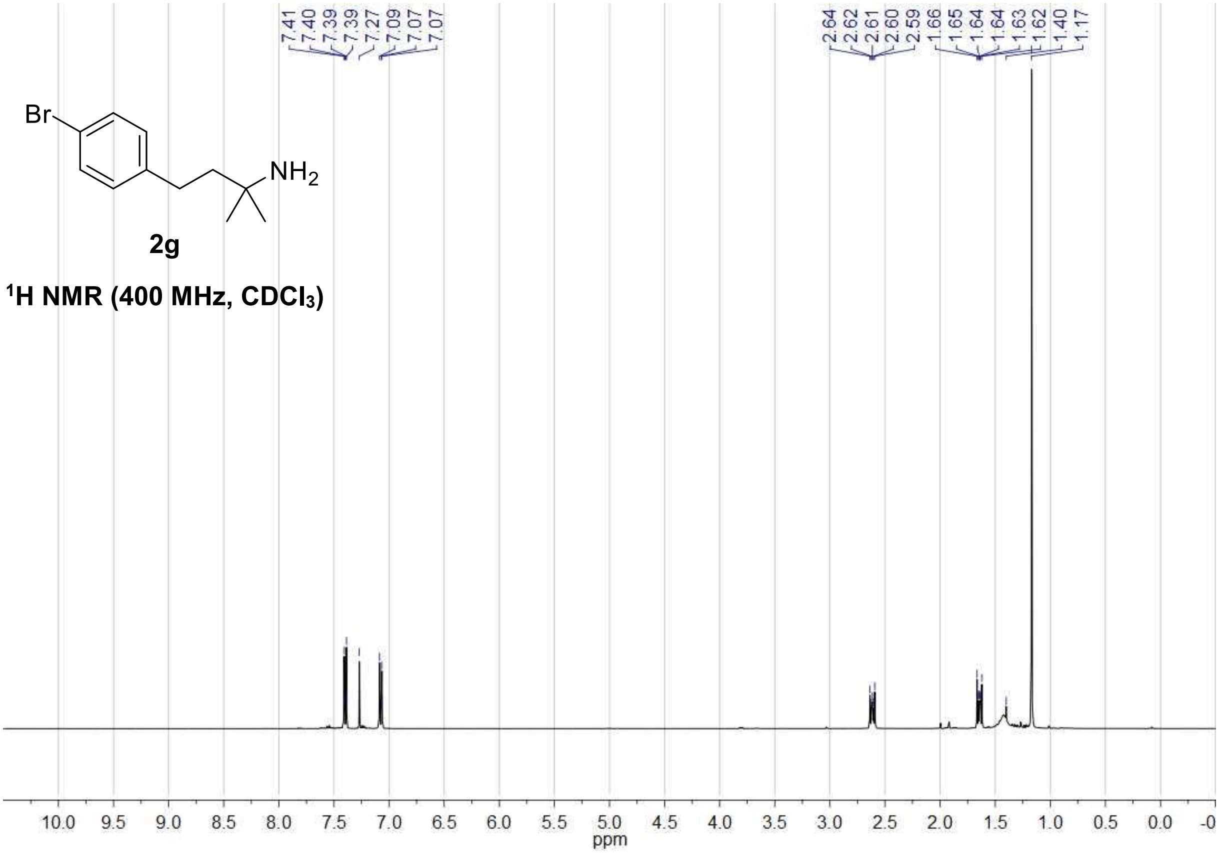

2g

^{1}H NMR (400 MHz, $CDCl_3$)

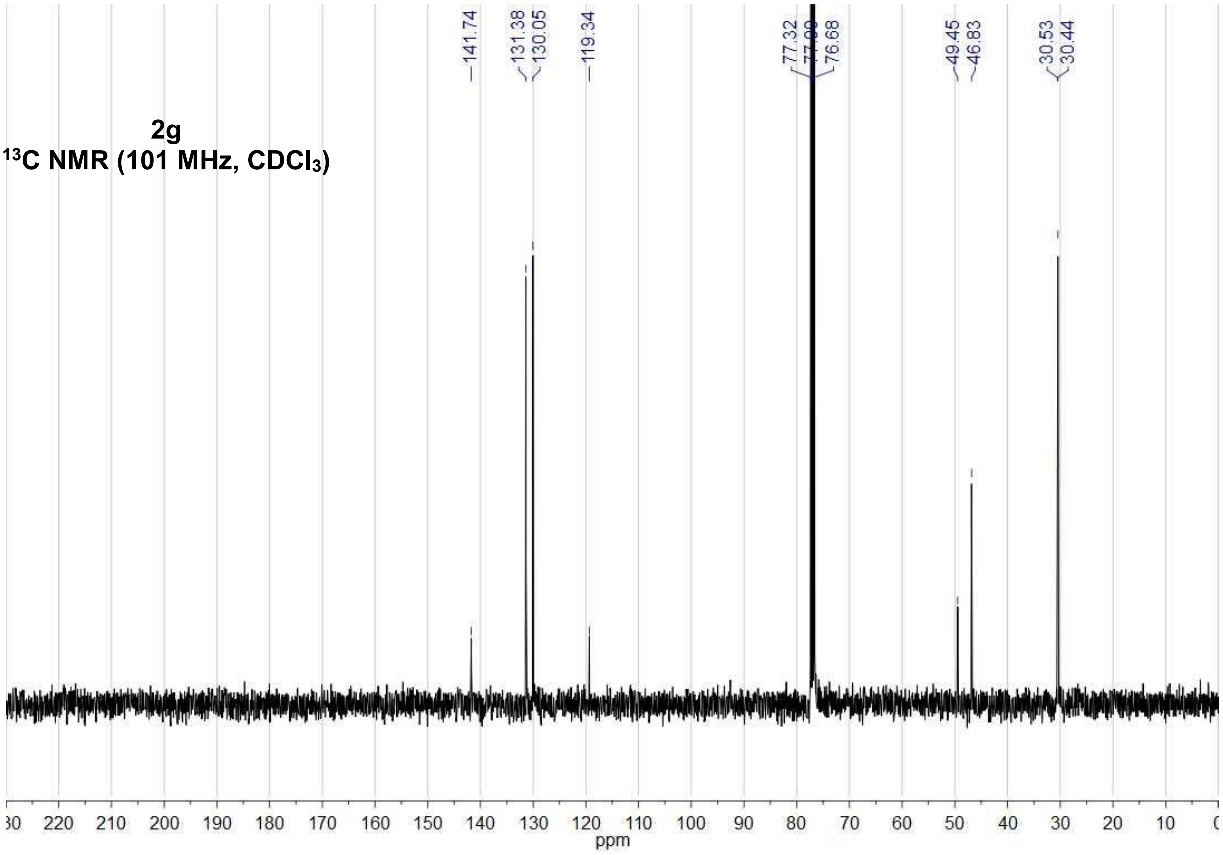

2g

^{13}C NMR (101 MHz, $CDCl_3$)

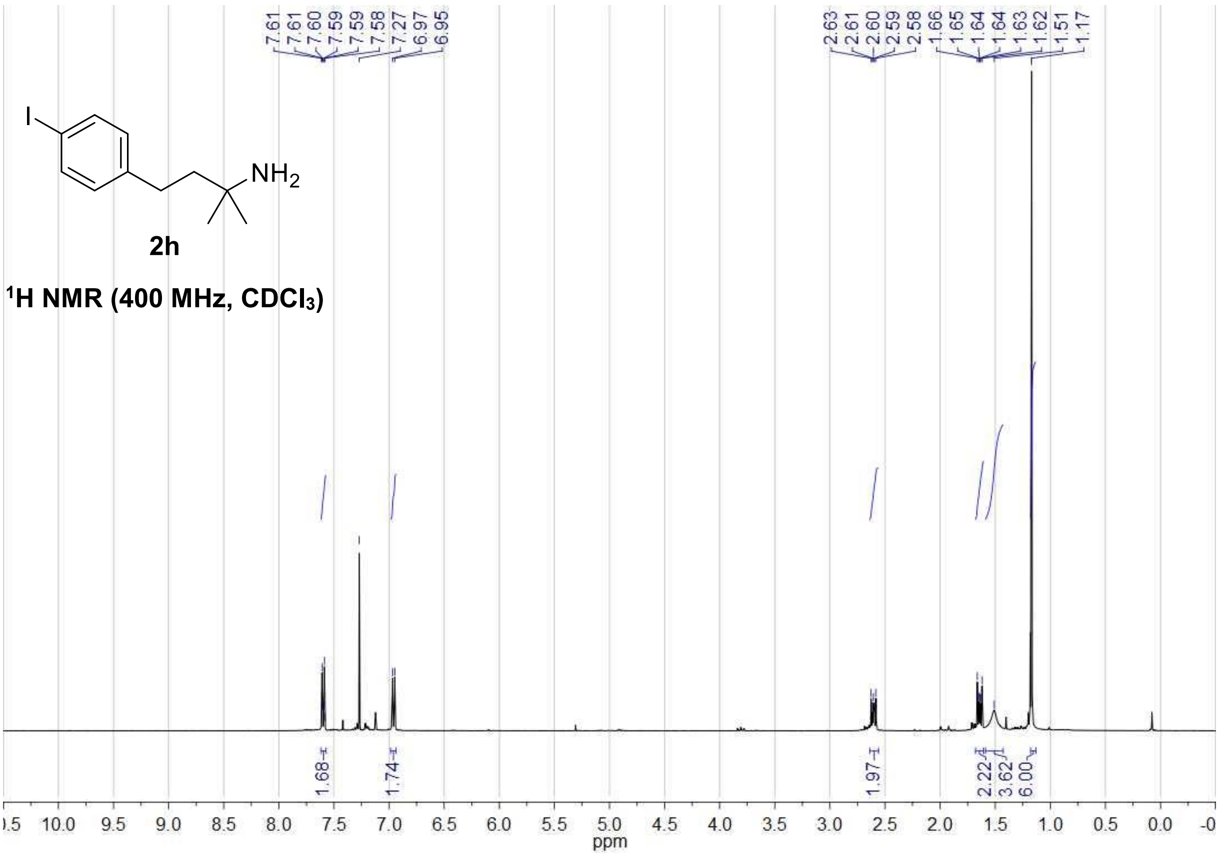
7.61
7.61
7.60
7.59
7.59
7.58
7.27
6.97
6.95
2.63
2.61
2.60
2.59
2.58
1.66
1.65
1.64
1.64
1.63
1.62
1.51
1.17
I
NH2
2h
1H NMR (400 MHz, CDCl3)
1.68
1.74
1.97
2.22
3.62
6.00
10.0 9.5 9.0 8.5 8.0 7.5 7.0 6.5 6.0 5.5 5.0 4.5 4.0 3.5 3.0 2.5 2.0 1.5 1.0 0.5 0.0
ppm

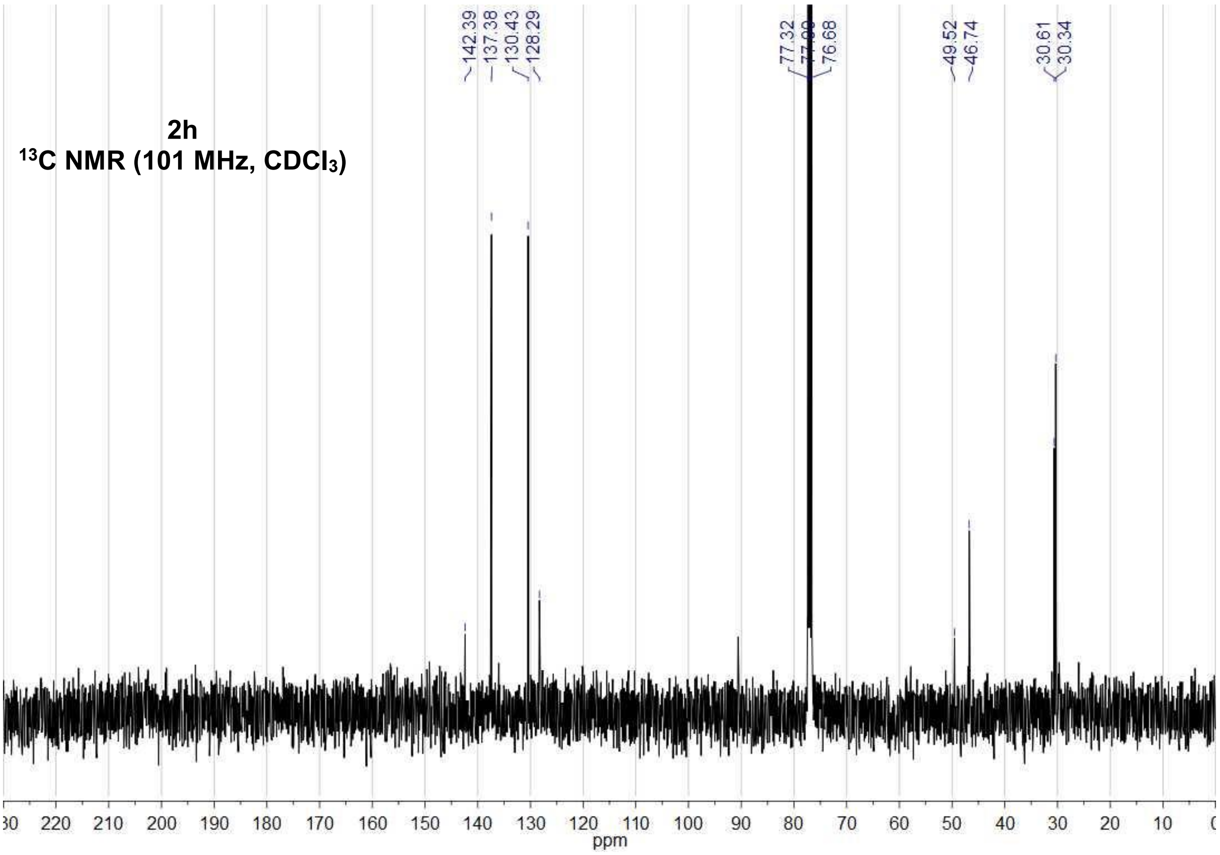
142.39
137.38
130.43
128.29
77.32
76.68
49.52
46.74
30.61
30.34
2h
13C NMR (101 MHz, CDCl3)
220 210 200 190 180 170 160 150 140 130 120 110 100 90 80 70 60 50 40 30 20 10
ppm

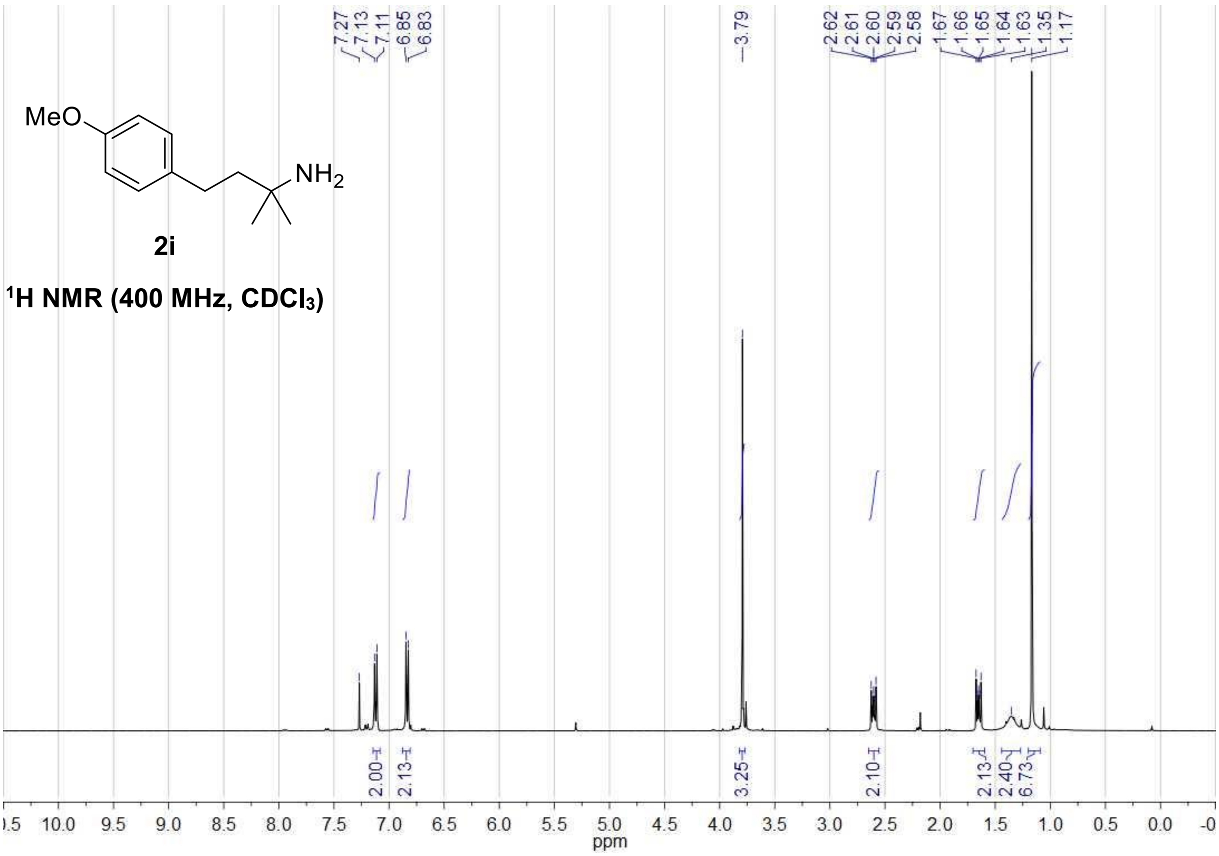
MeO
NH2
2i
1H NMR (400 MHz, CDCl3)
7.27
7.13
7.11
6.85
6.83
3.79
2.62
2.61
2.60
2.59
2.58
1.67
1.66
1.65
1.64
1.63
1.35
1.17
2.00
2.13
3.25
2.10
2.13
2.40
6.73
ppm

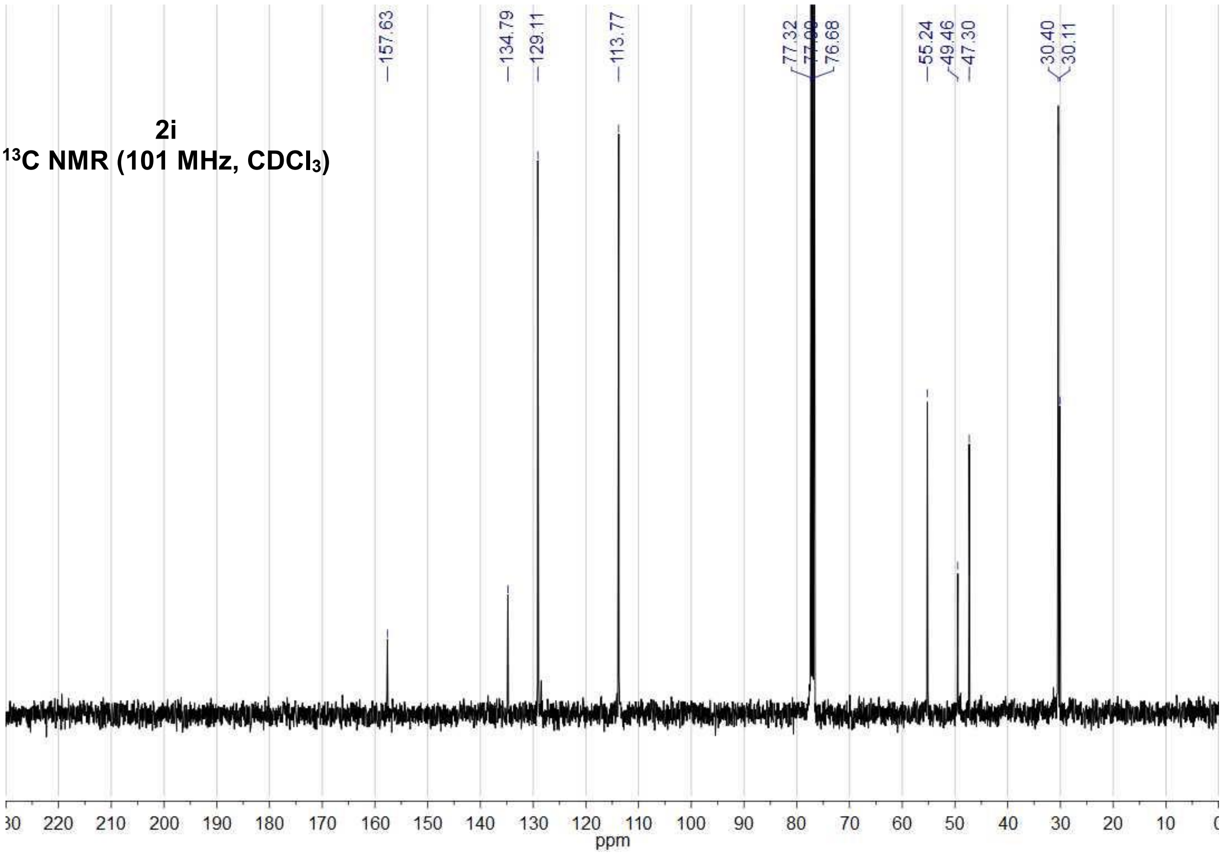
2i
13C NMR (101 MHz, CDCl3)
157.63
134.79
129.11
113.77
77.32
76.68
55.24
49.46
47.30
30.40
30.11
ppm

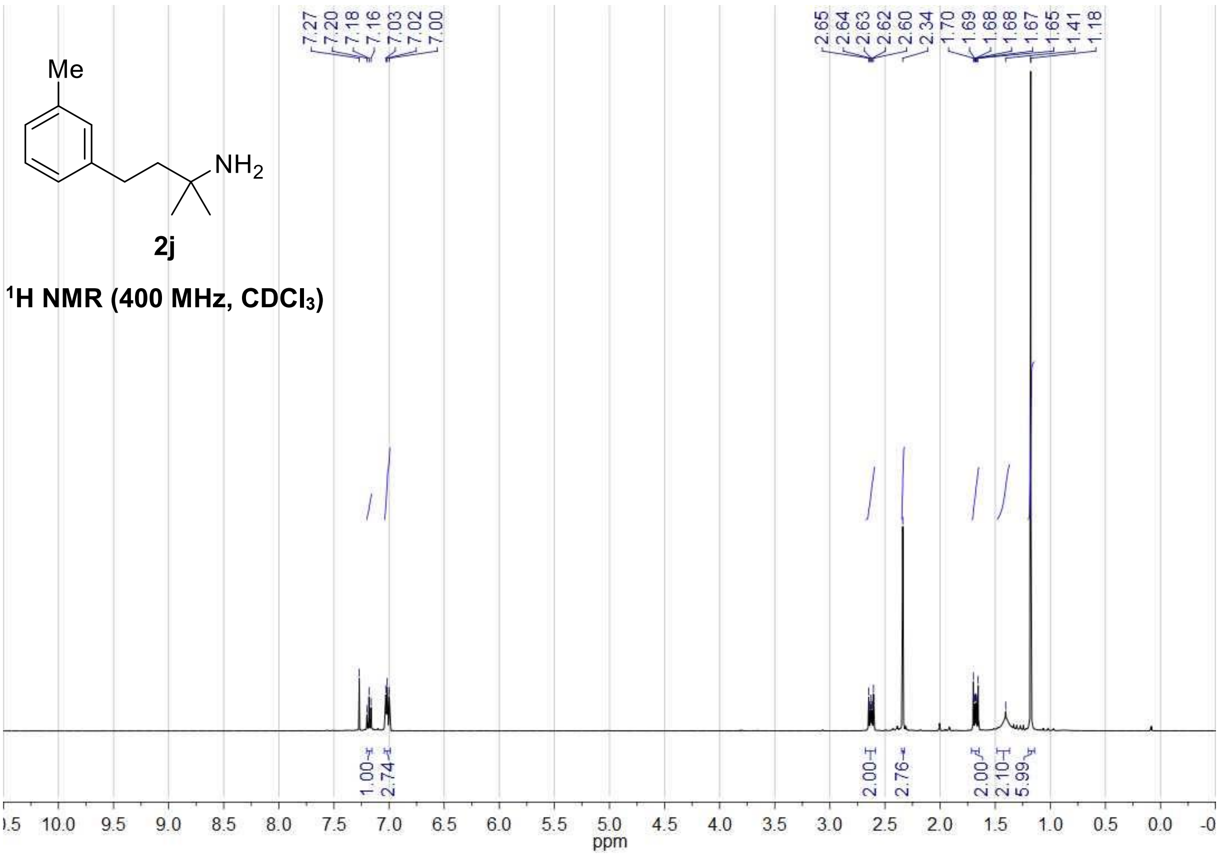

2j

¹H NMR (400 MHz, CDCl$_3$)

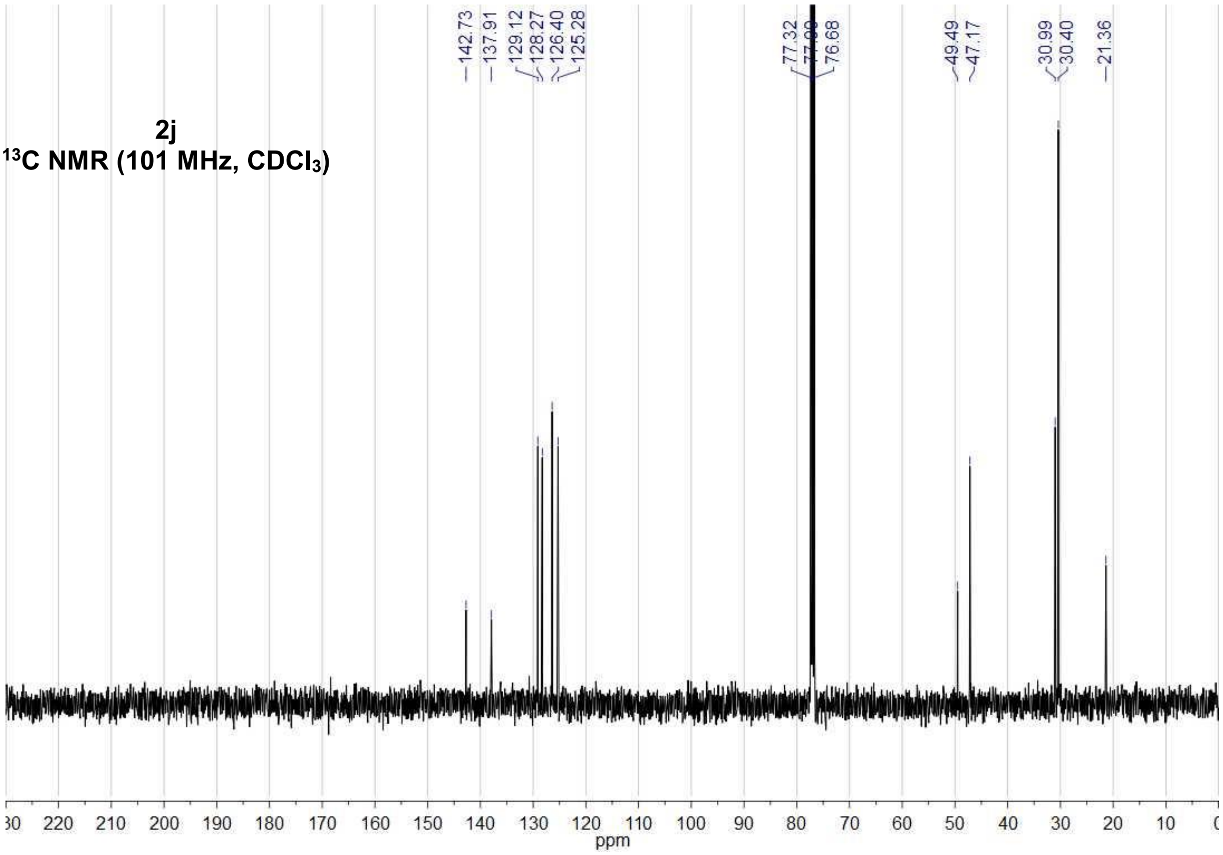

2j

¹³C NMR (101 MHz, CDCl$_3$)

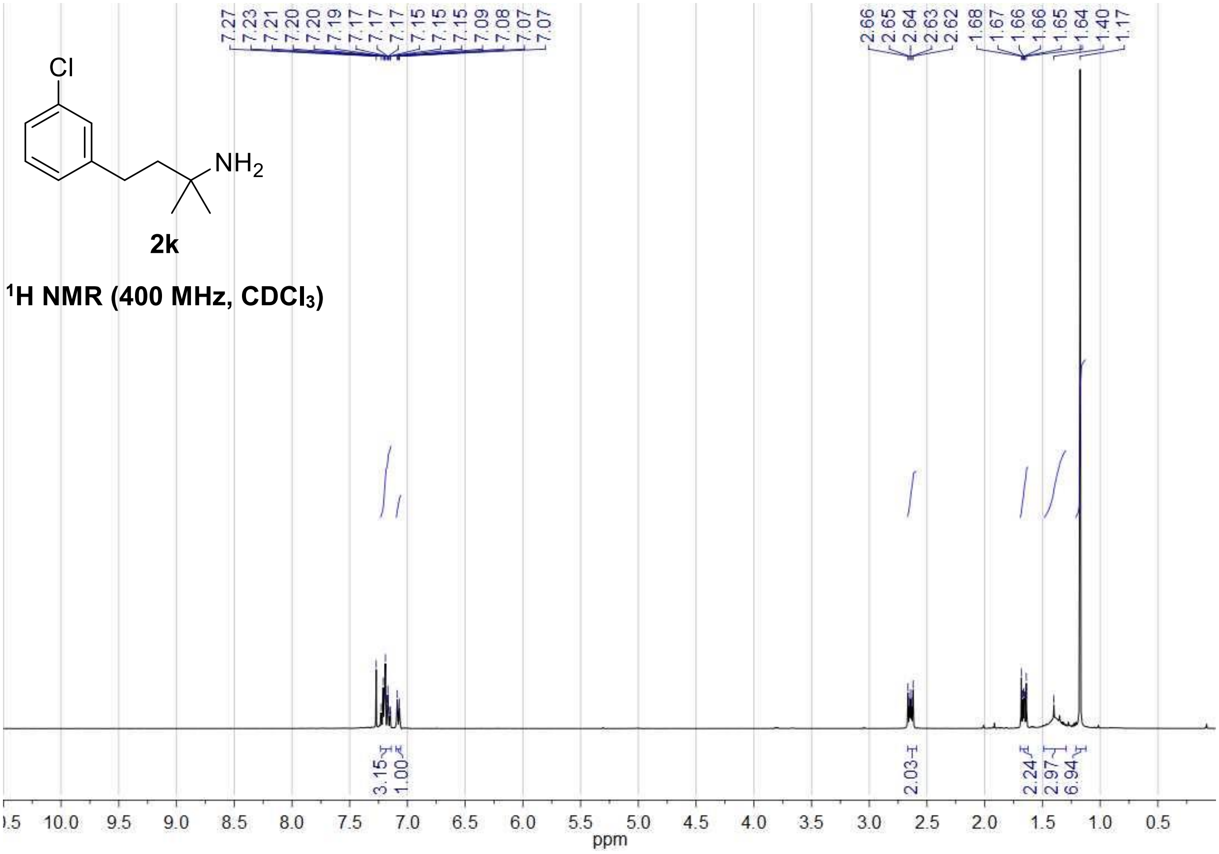

2k

^{1}H NMR (400 MHz, $CDCl_3$)

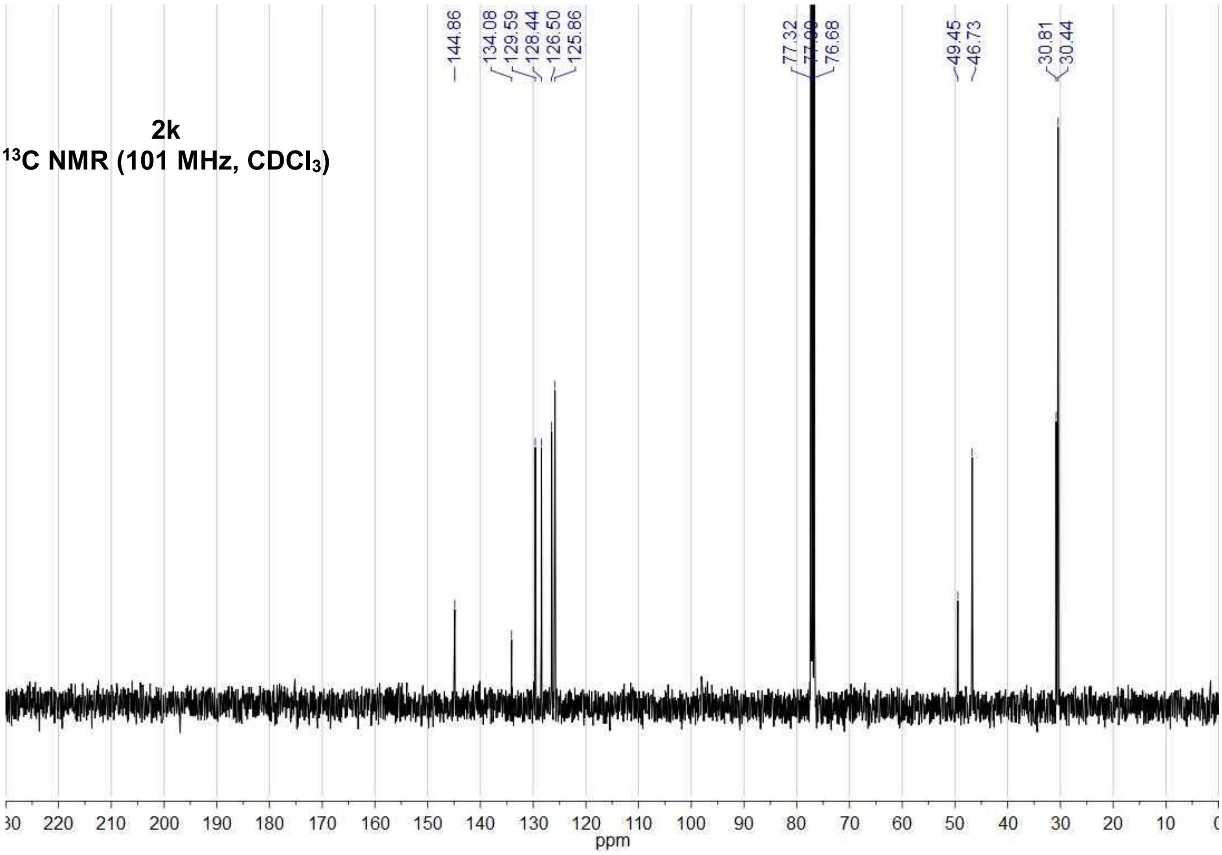

2k

^{13}C NMR (101 MHz, $CDCl_3$)

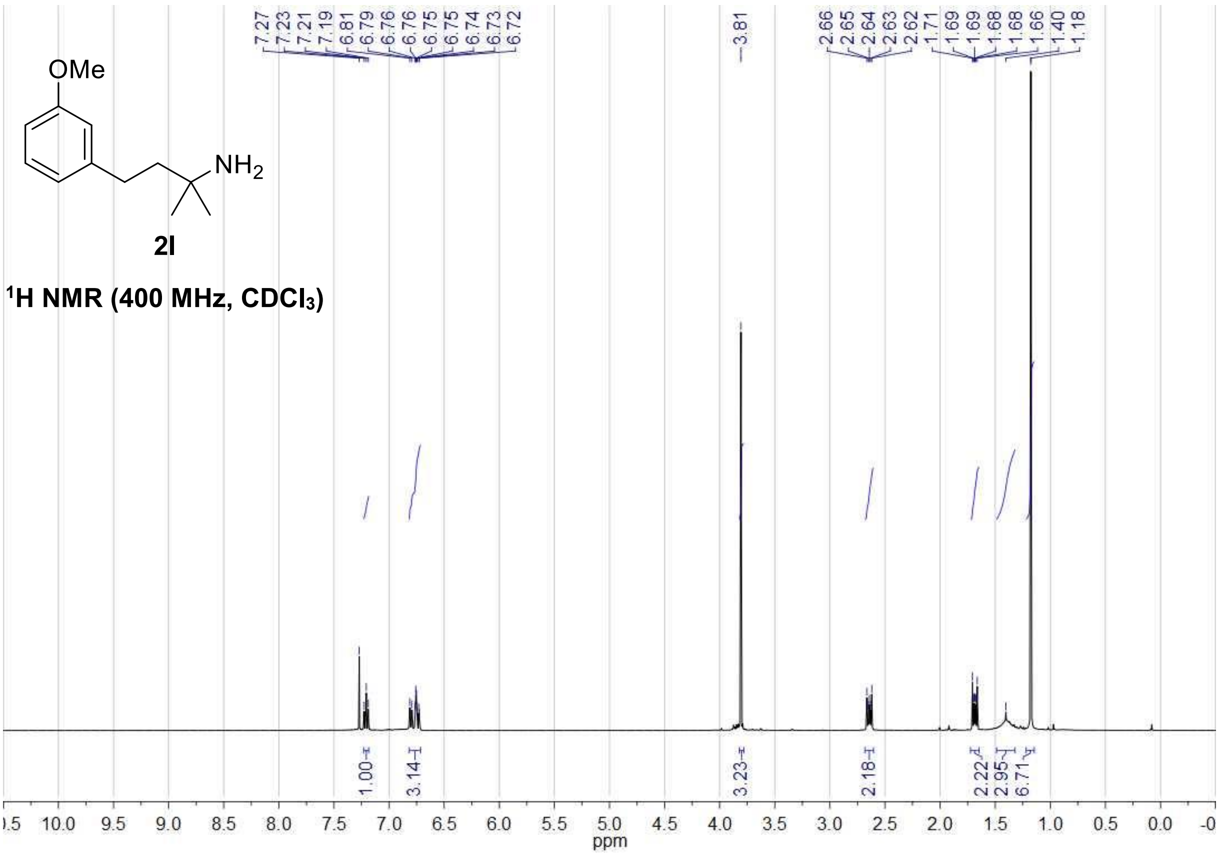

2l

^{1}H NMR (400 MHz, CDCl$_3$)

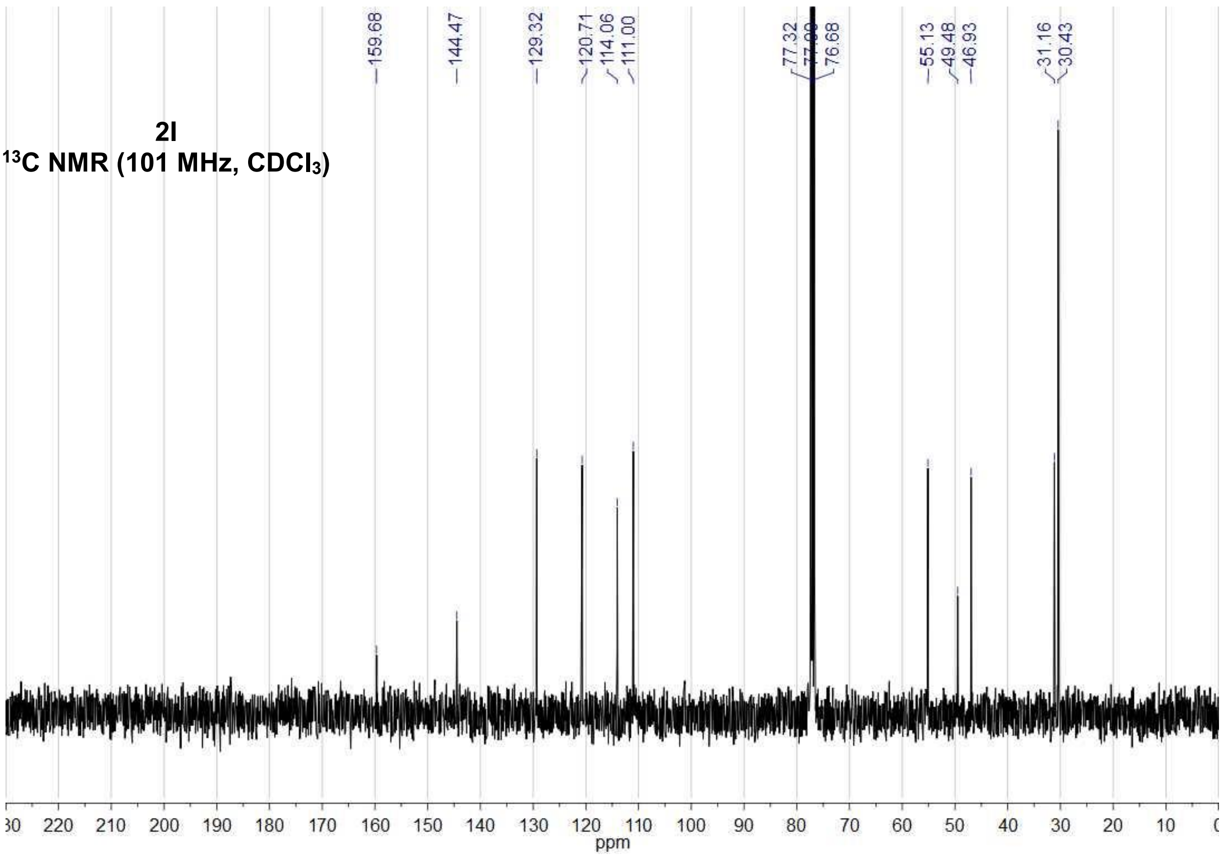

2l

^{13}C NMR (101 MHz, CDCl$_3$)

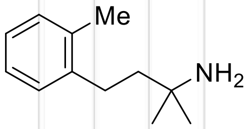

2m

^{1}H NMR (400 MHz, CDCl$_3$)

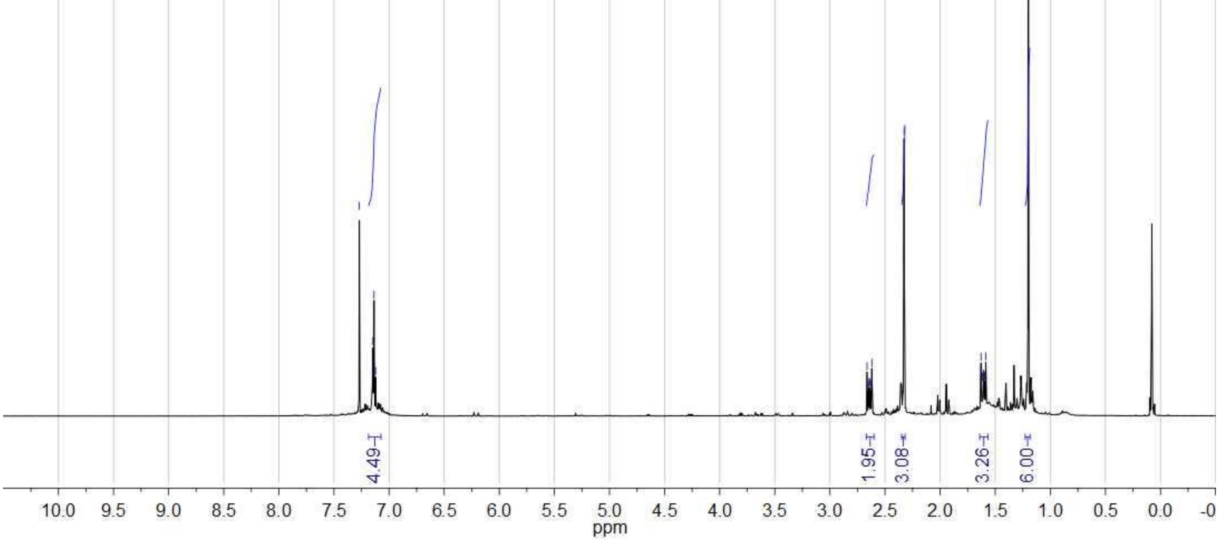

2m

^{13}C NMR (101 MHz, CDCl$_3$)

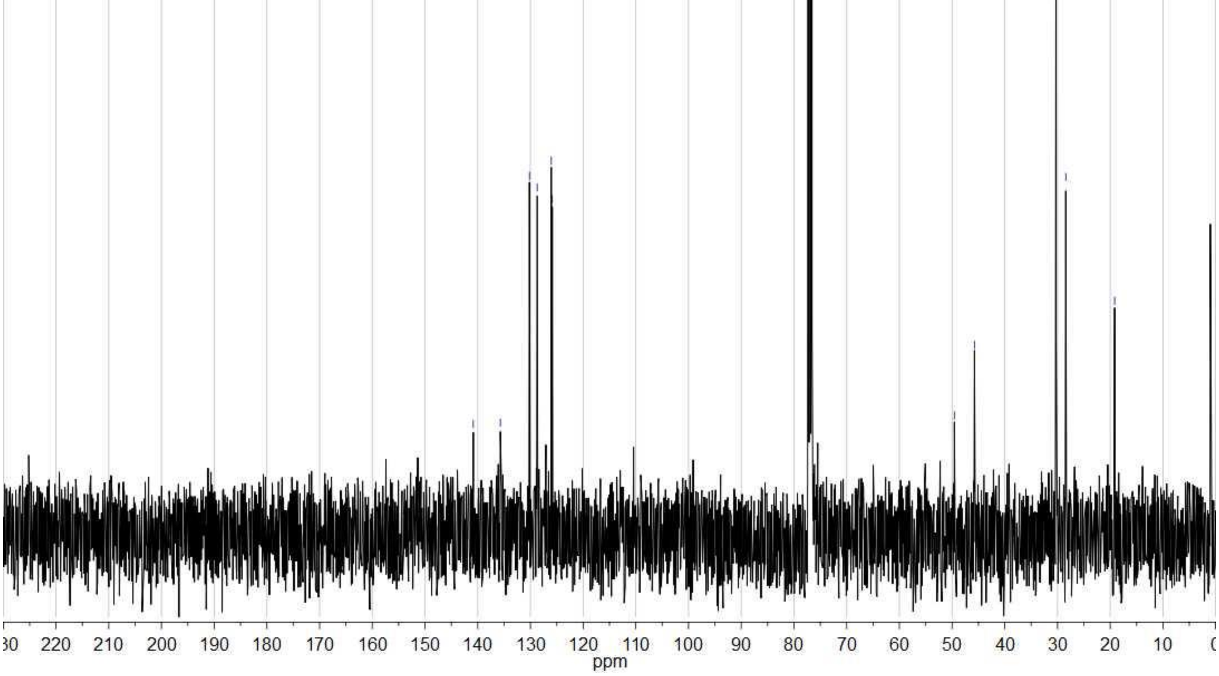

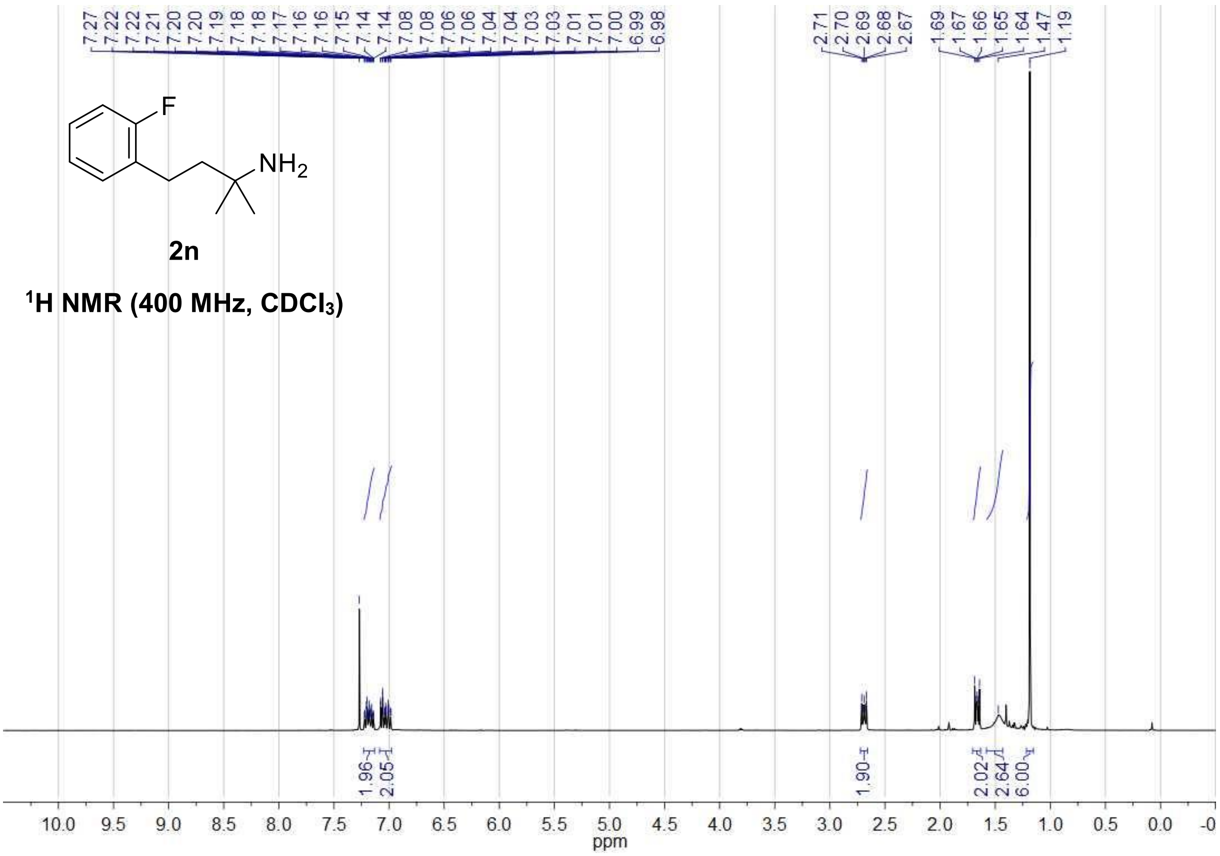
F
NH2
2n
1H NMR (400 MHz, CDCl3)
ppm

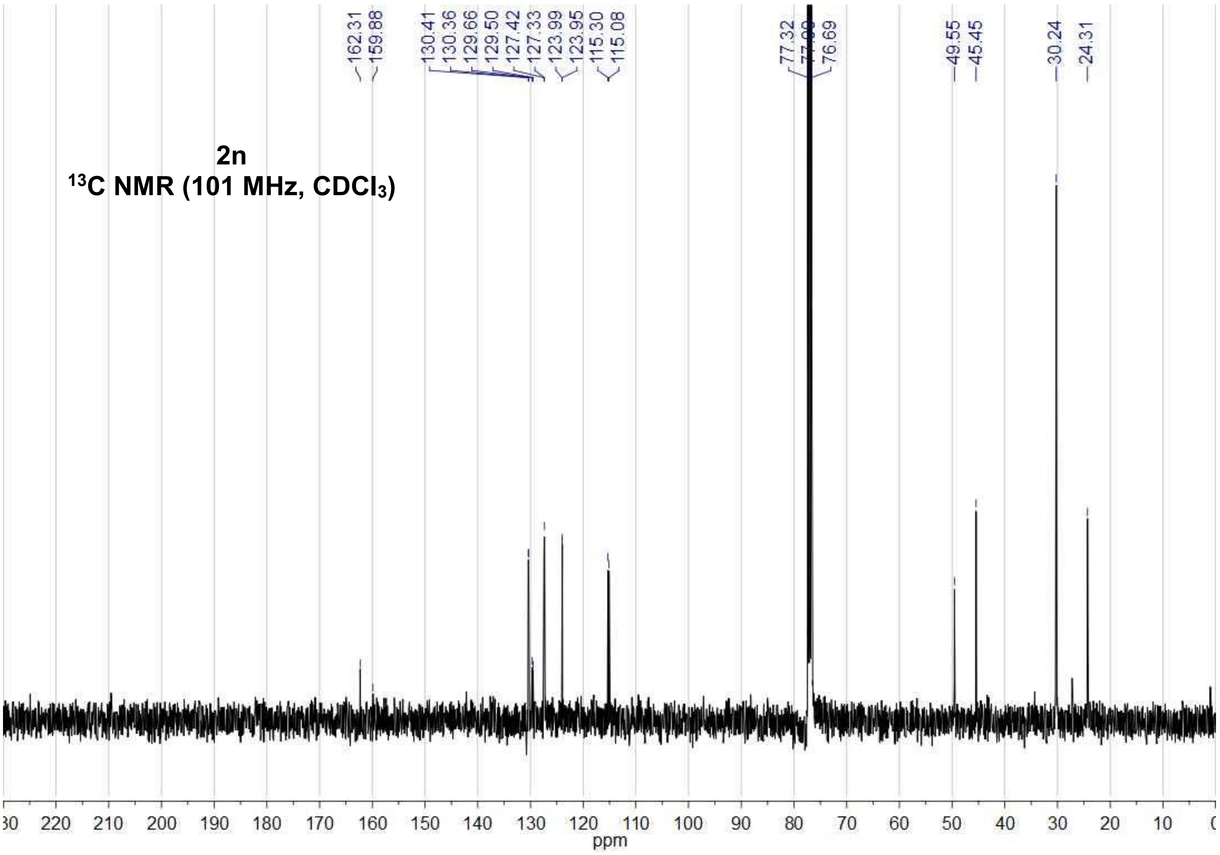
2n
13C NMR (101 MHz, CDCl3)
ppm

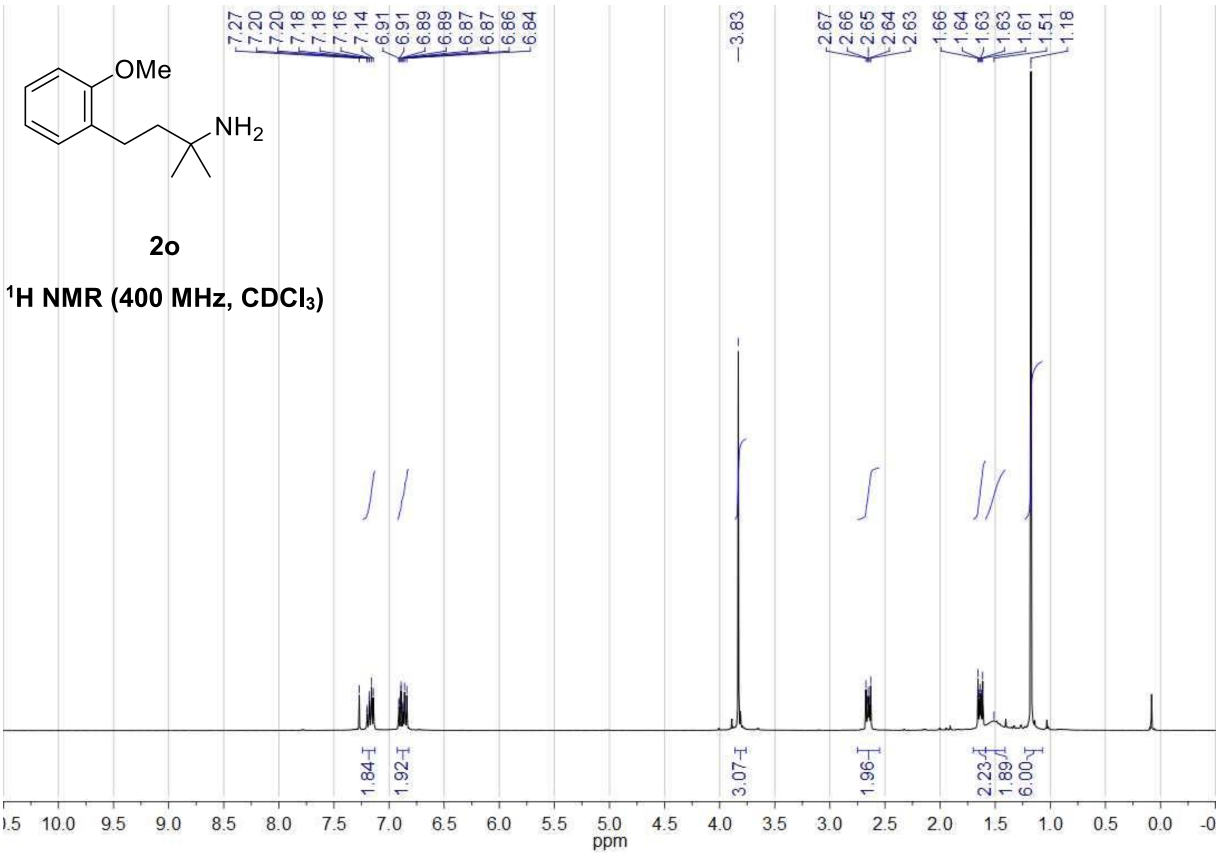
OMe
NH2
2o
1H NMR (400 MHz, CDCl3)
ppm

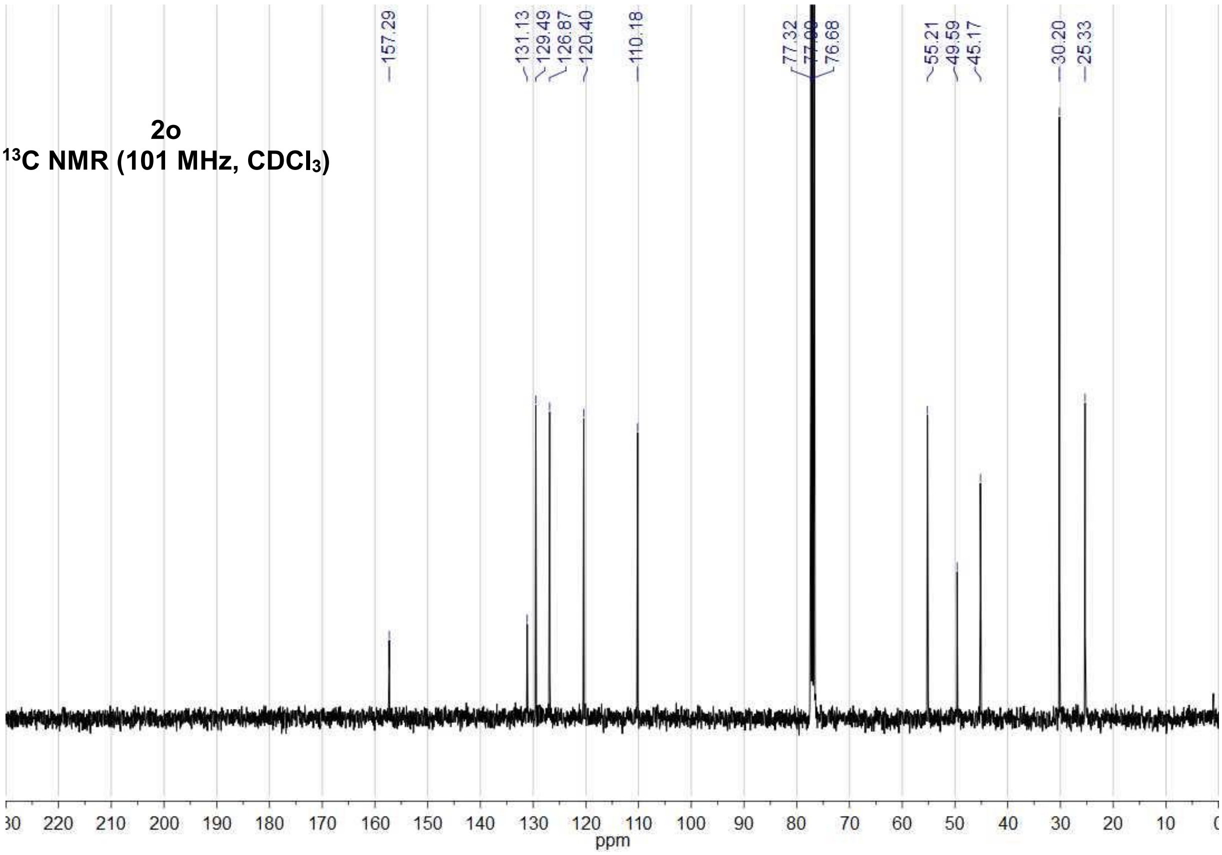
2o
13C NMR (101 MHz, CDCl3)
ppm

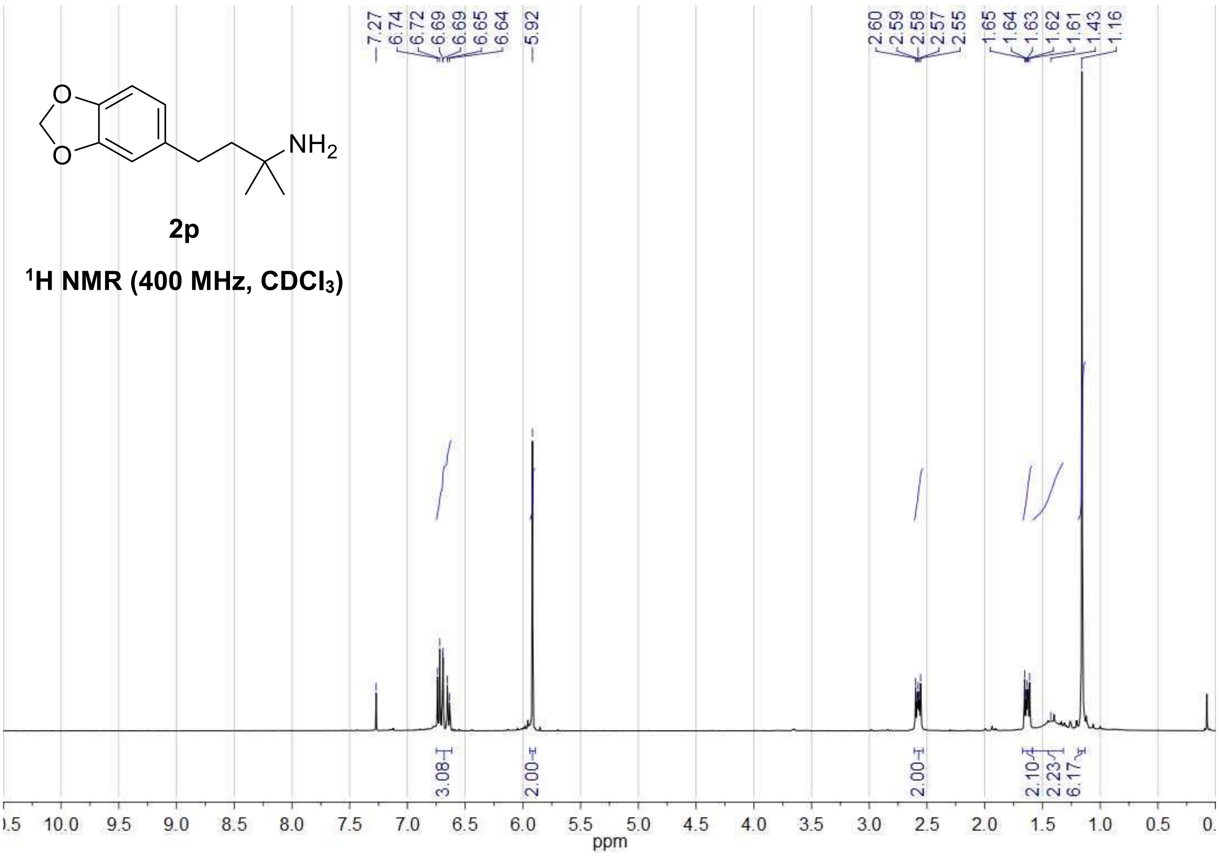
7.27
6.74
6.72
6.69
6.69
6.65
6.64
5.92
2.60
2.59
2.58
2.57
2.55
1.65
1.64
1.63
1.62
1.61
1.43
1.16
NH2
O
O
2p
1H NMR (400 MHz, CDCl3)
3.08
2.00
2.00
2.10
2.23
6.17
ppm

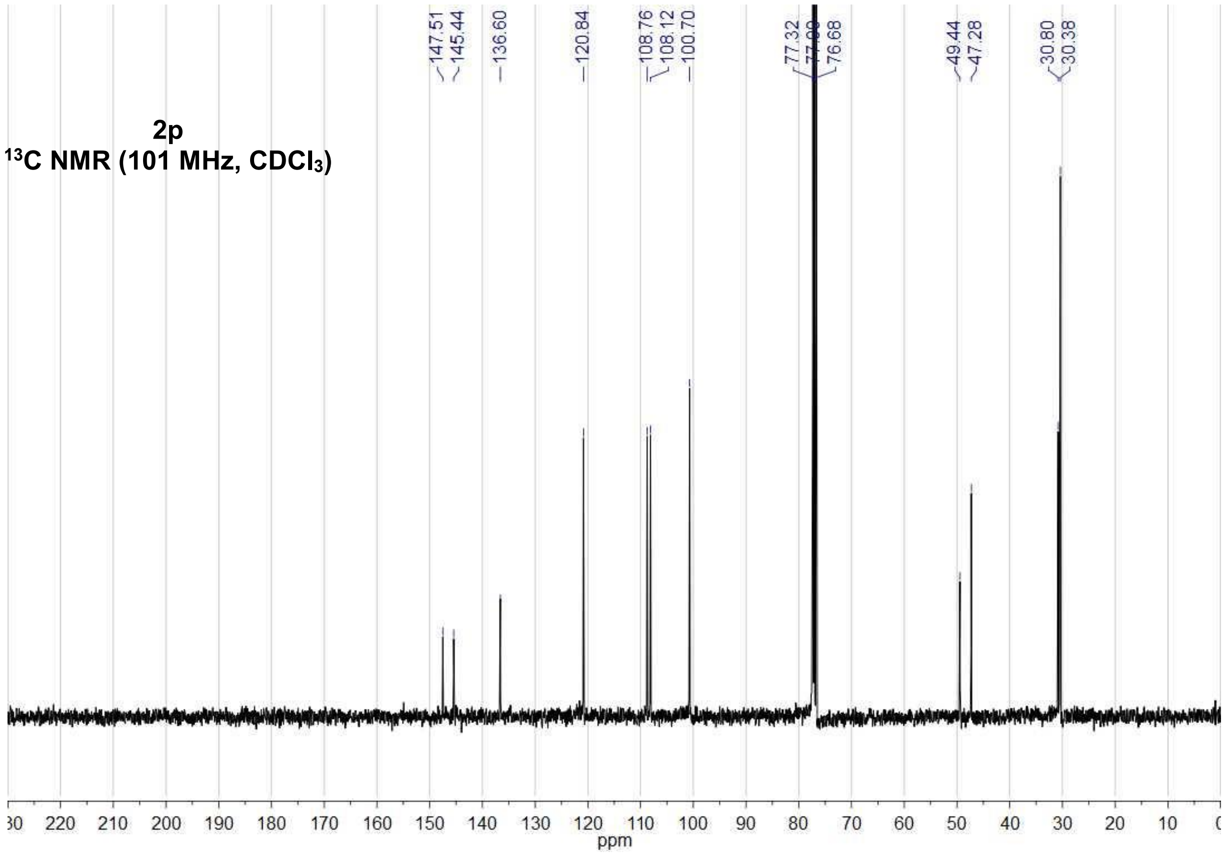
147.51
145.44
136.60
120.84
108.76
108.12
100.70
77.32
76.68
49.44
47.28
30.80
30.38
2p
13C NMR (101 MHz, CDCl3)
ppm

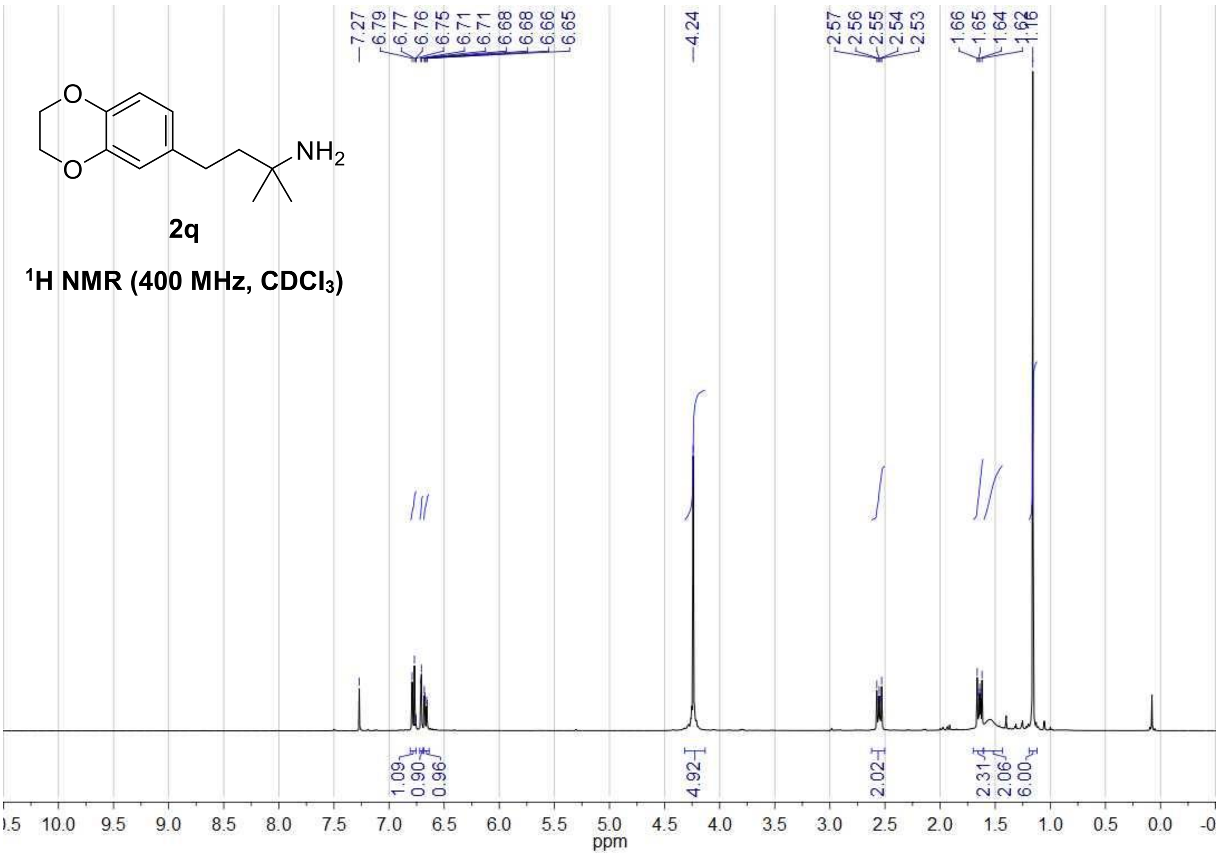
7.27
6.79 6.77 6.76 6.75 6.71 6.71 6.68 6.68 6.66 6.65
4.24
2.57 2.56 2.55 2.54 2.53
1.66 1.65 1.64 1.62 1.16
NH2
2q
1H NMR (400 MHz, CDCl3)
1.09 0.90 0.96
4.92
2.02
2.31 2.06 6.00
ppm

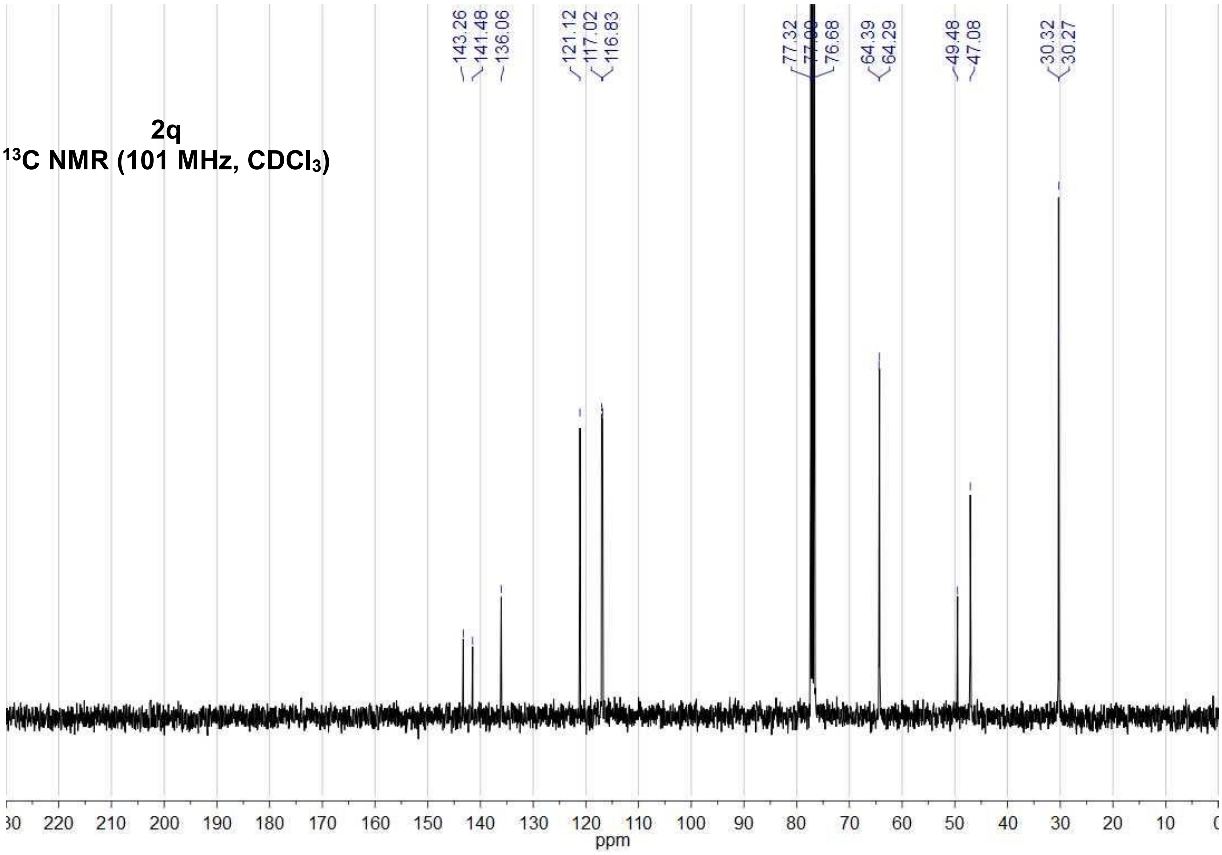
143.26 141.48 136.06
121.12 117.02 116.83
77.32 76.68
64.39 64.29
49.48 47.08
30.32 30.27
2q
13C NMR (101 MHz, CDCl3)
ppm

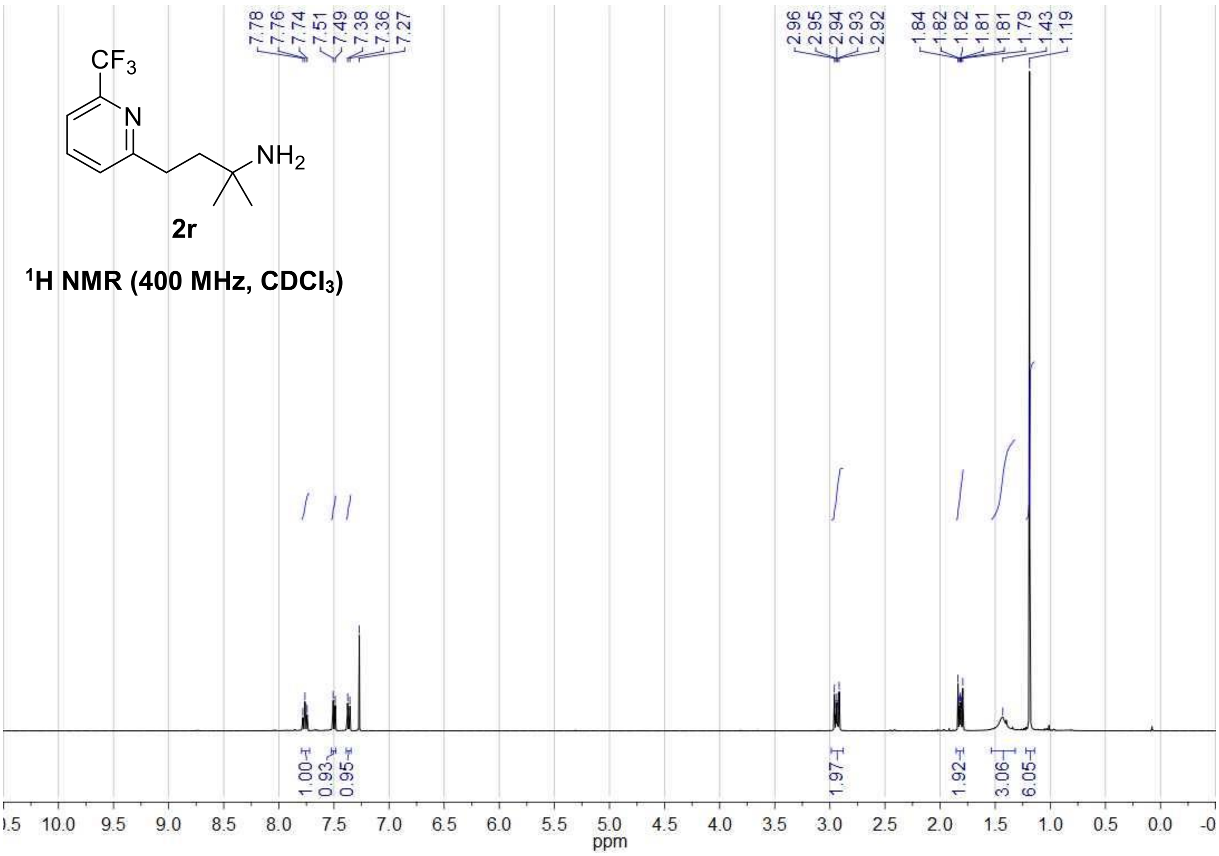
CF3
N
NH2
2r
1H NMR (400 MHz, CDCl3)
7.78 7.76 7.74 7.51 7.49 7.38 7.36 7.27
2.96 2.95 2.94 2.93 2.92
1.84 1.82 1.82 1.81 1.81 1.79 1.43 1.19
1.00 0.93 0.95 1.97 1.92 3.06 6.05
ppm

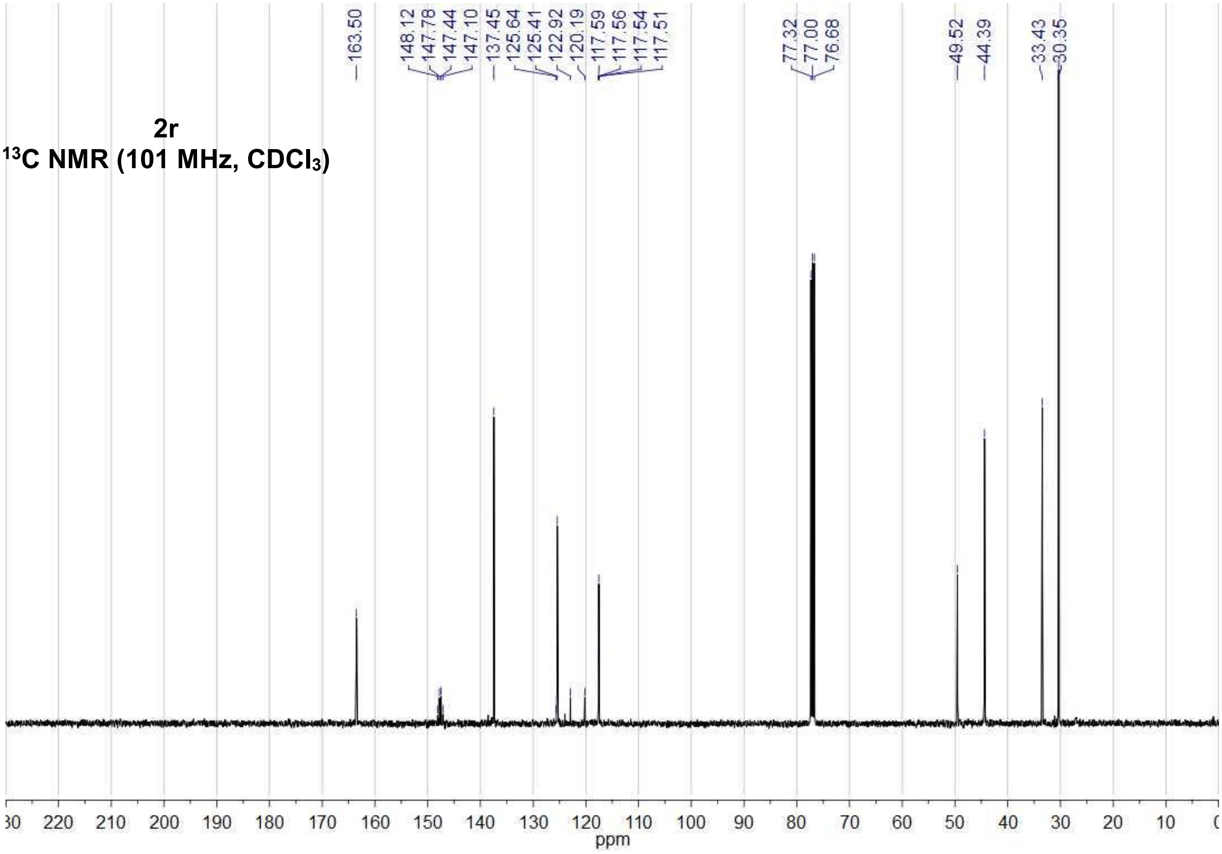
2r
13C NMR (101 MHz, CDCl3)
163.50
148.12 147.78 147.44 147.10 137.45 125.64 125.41 122.92 120.19 117.59 117.56 117.54 117.51
77.32 77.00 76.68
49.52 44.39 33.43 30.35
ppm

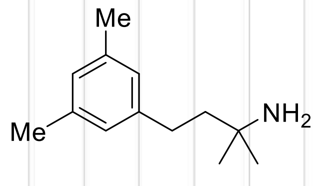

2s

^{1}H NMR (400 MHz, $CDCl_3$)

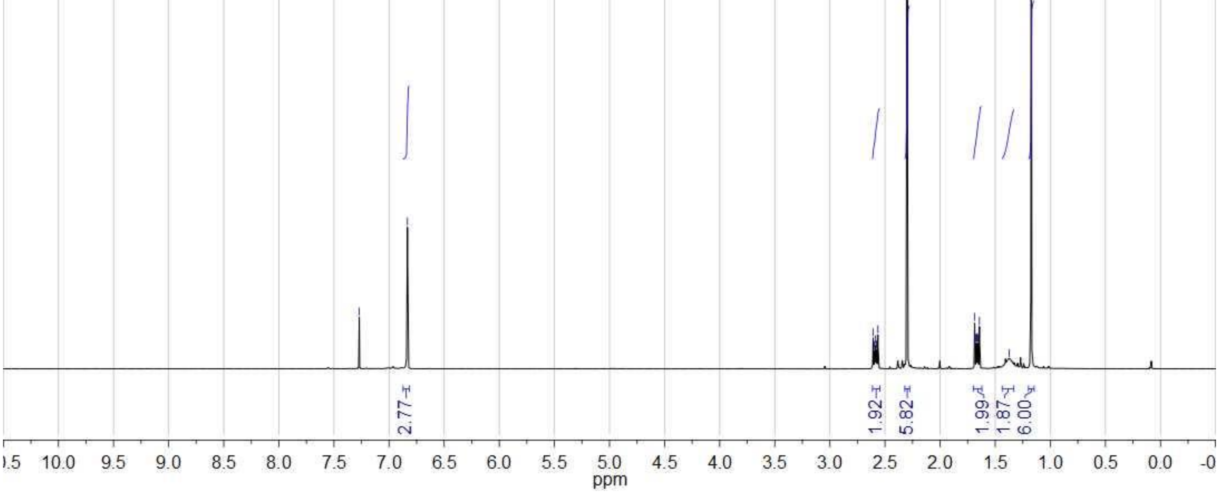

2s

^{13}C NMR (101 MHz, $CDCl_3$)

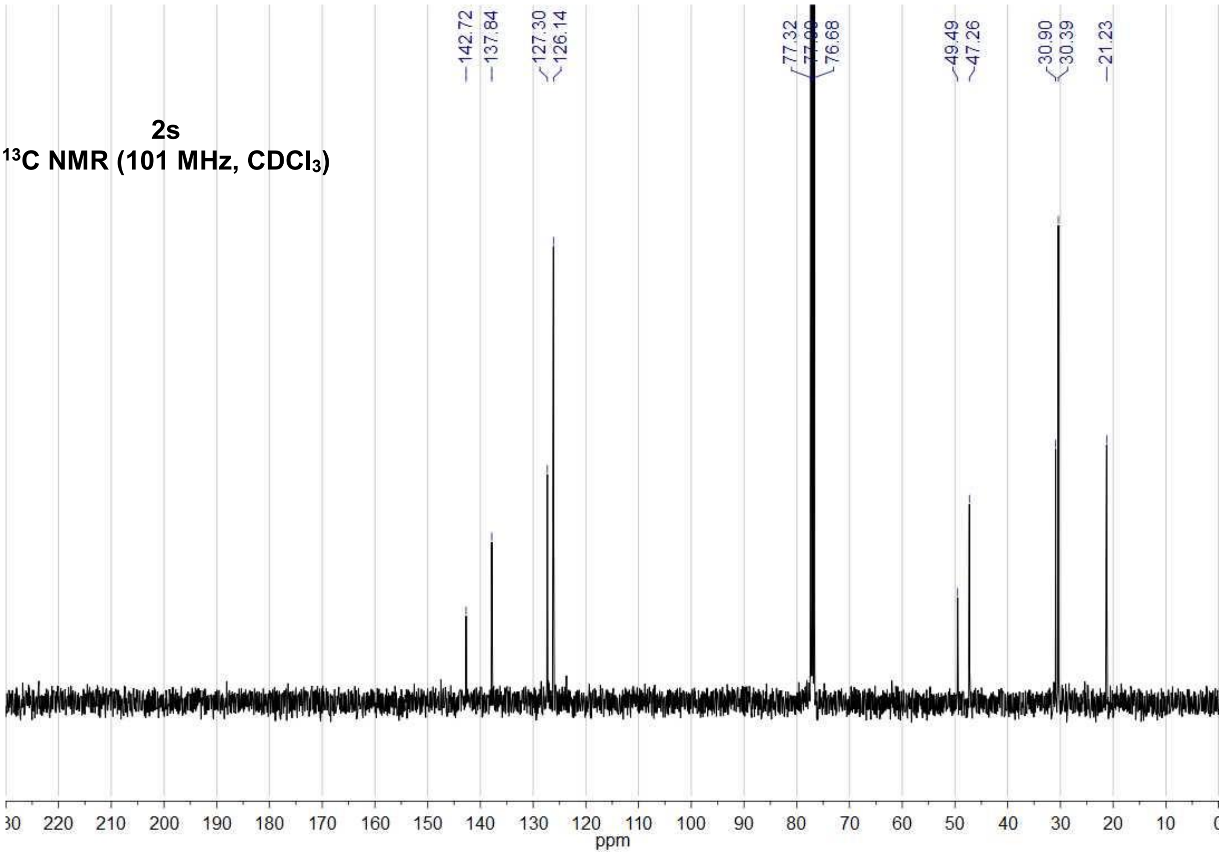

S4

^{1}H NMR (400 MHz, $CDCl_3$)

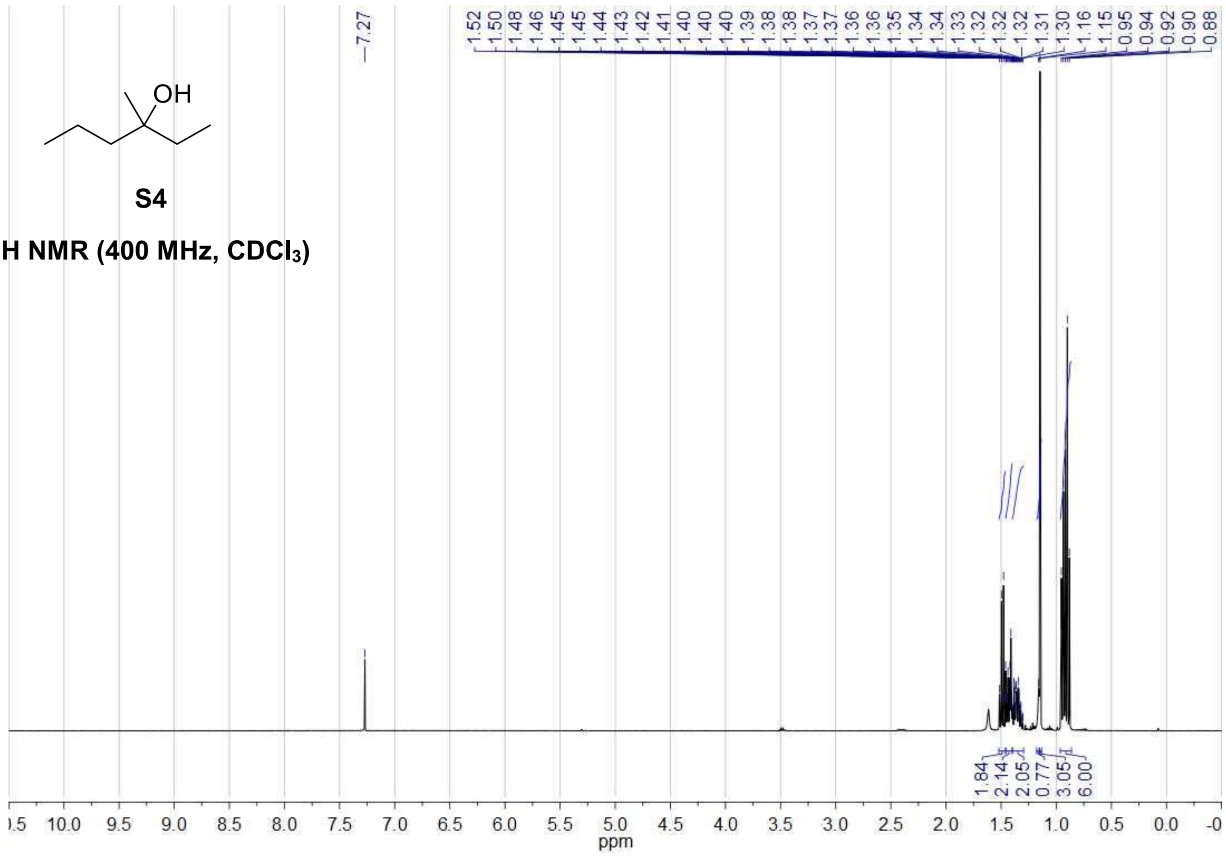

S4

^{13}C NMR (101 MHz, $CDCl_3$)

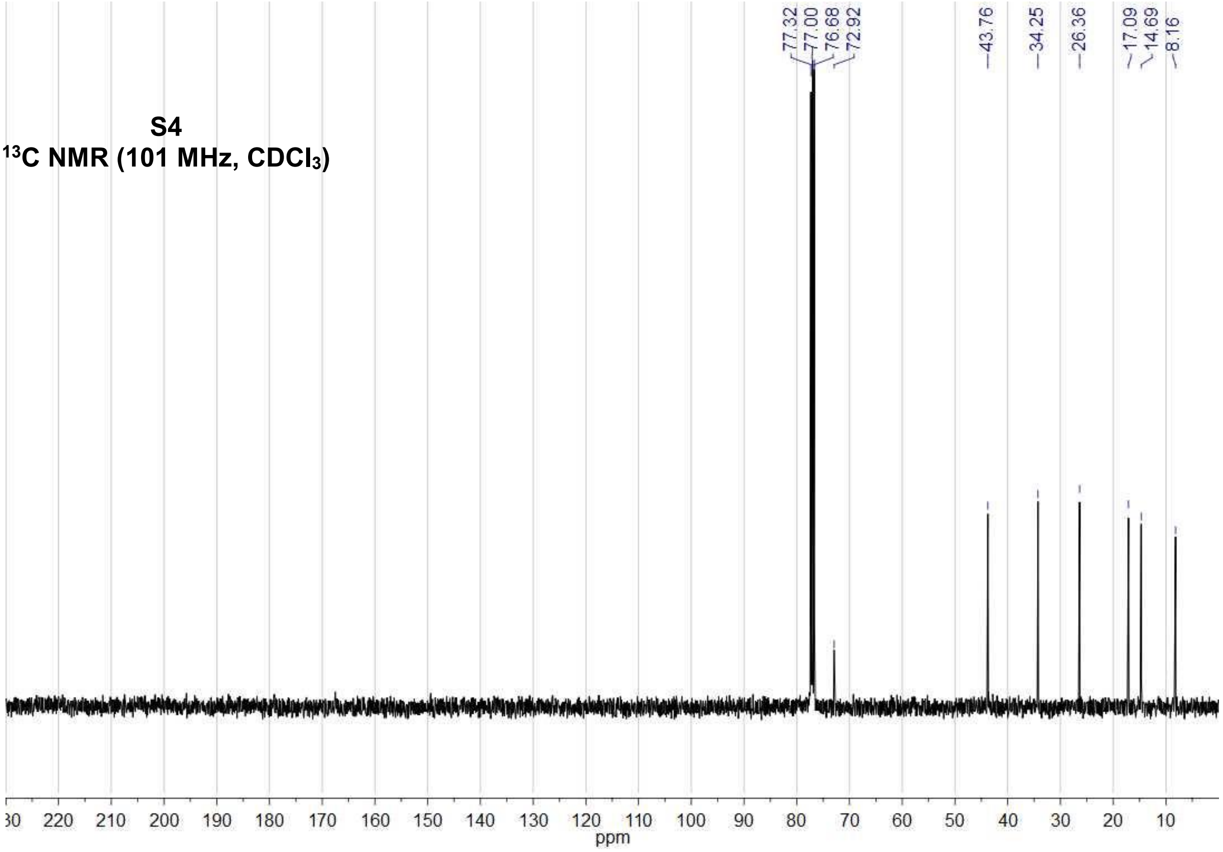

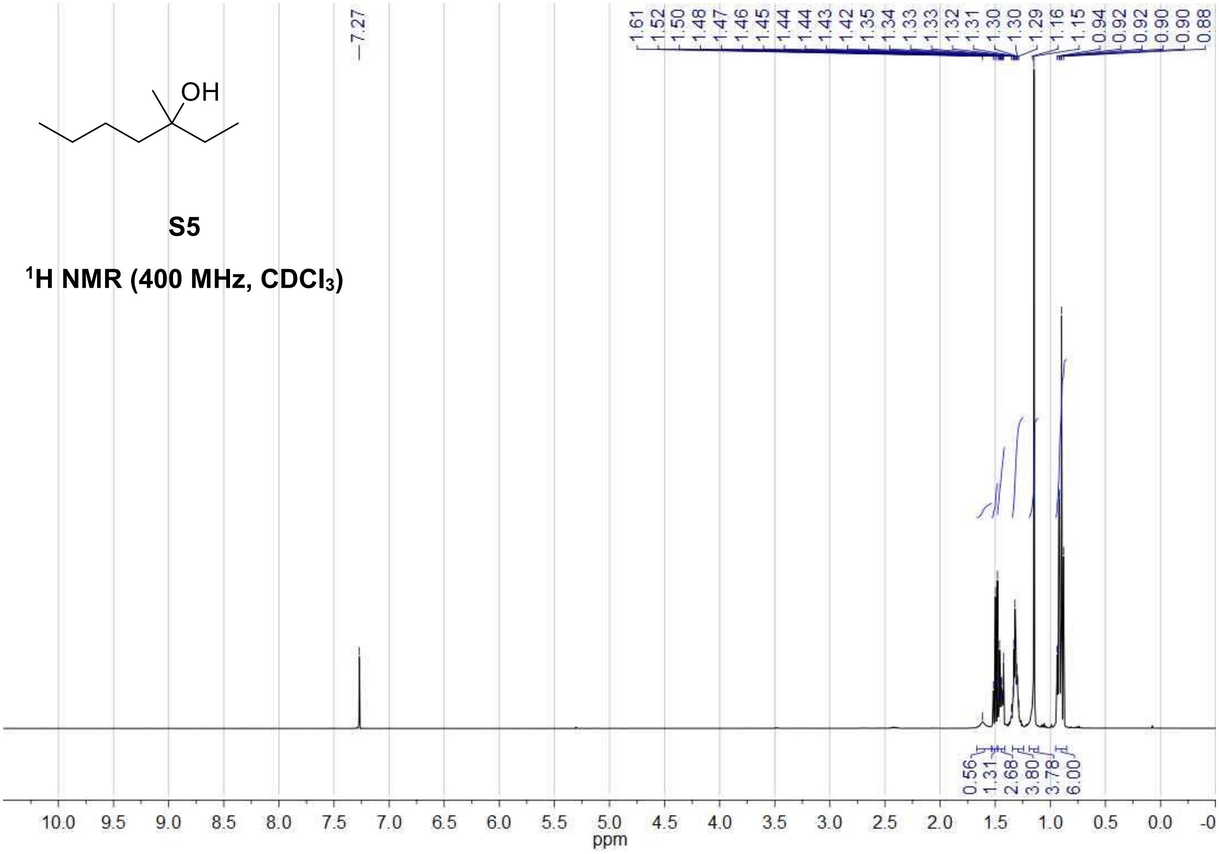

S5

^{1}H NMR (400 MHz, $CDCl_3$)

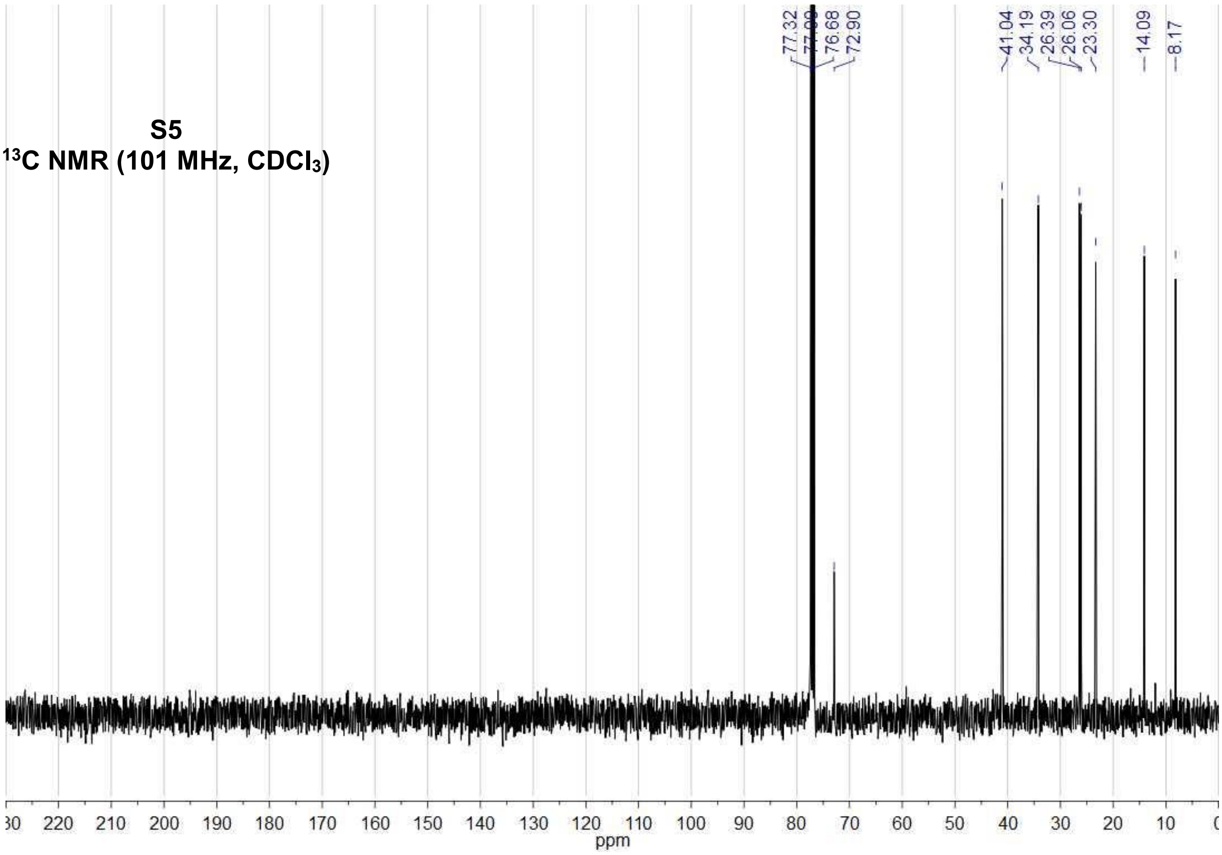

S5

^{13}C NMR (101 MHz, $CDCl_3$)

S6

^{1}H NMR (400 MHz, $CDCl_3$)

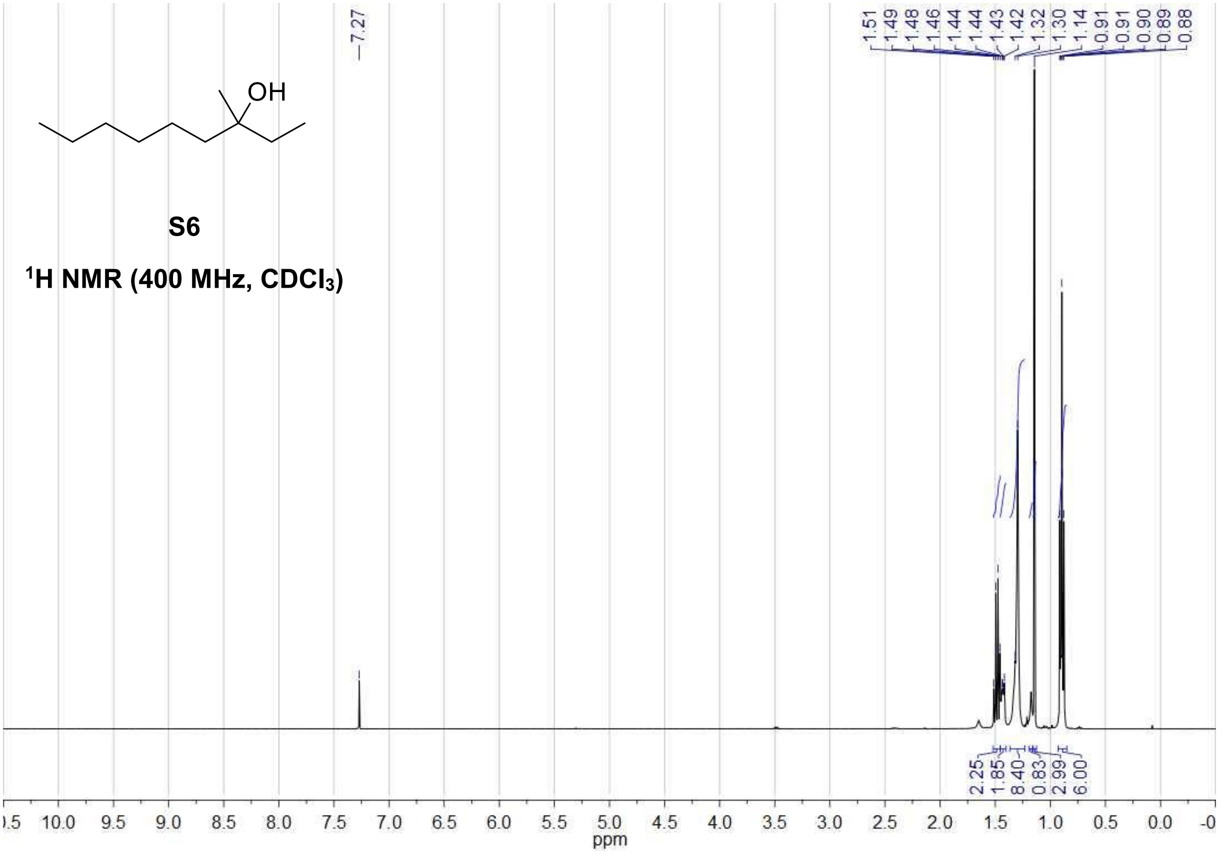

S6

^{13}C NMR (101 MHz, $CDCl_3$)

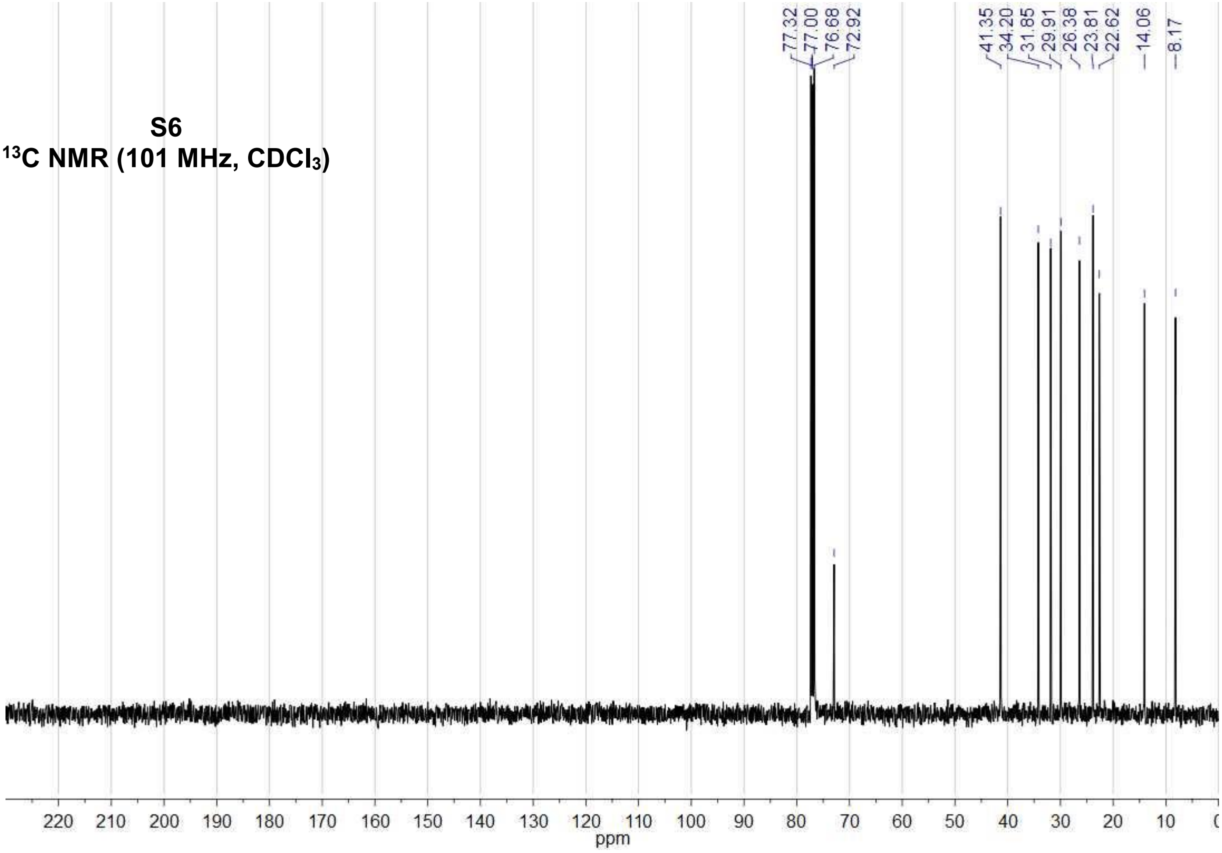

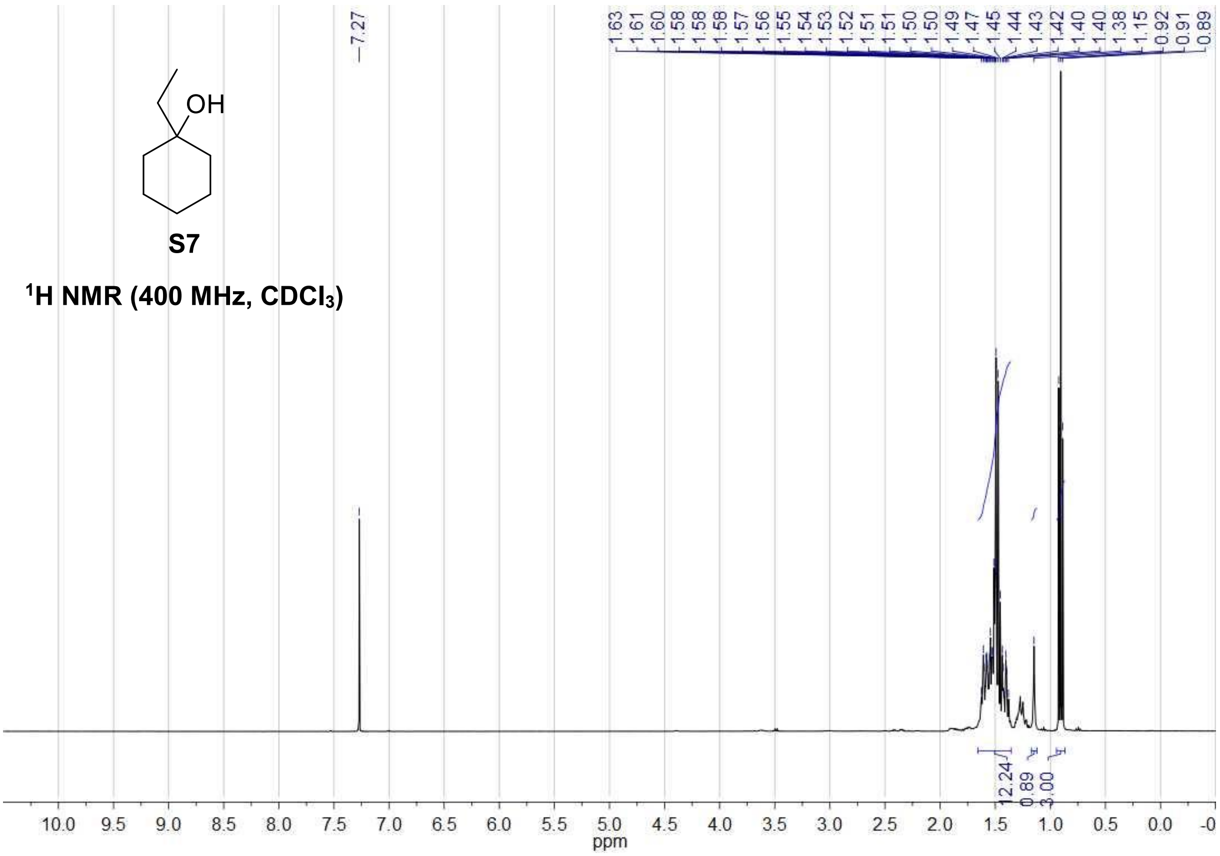
OH
S7
1H NMR (400 MHz, CDCl3)
7.27
1.63 1.61 1.60 1.58 1.58 1.57 1.56 1.55 1.54 1.53 1.52 1.51 1.51 1.50 1.50 1.49 1.47 1.45 1.44 1.43 1.42 1.40 1.40 1.38 1.15 0.92 0.91 0.89
12.24
0.89
3.00
10.0 9.5 9.0 8.5 8.0 7.5 7.0 6.5 6.0 5.5 5.0 4.5 4.0 3.5 3.0 2.5 2.0 1.5 1.0 0.5 0.0
ppm

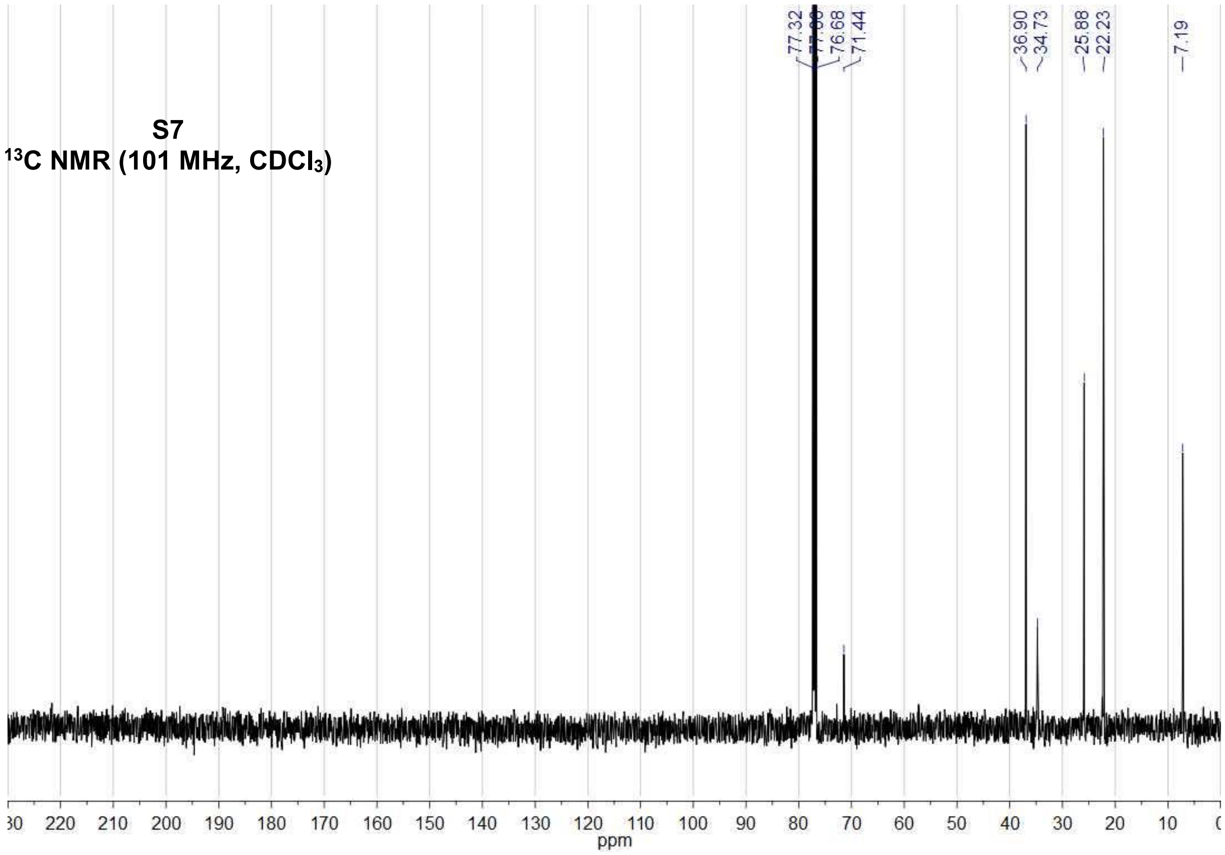
S7
13C NMR (101 MHz, CDCl3)
77.32
76.68
71.44
36.90
34.73
25.88
22.23
7.19
220 210 200 190 180 170 160 150 140 130 120 110 100 90 80 70 60 50 40 30 20 10
ppm

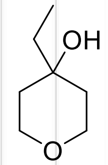

S8

^{1}H NMR (400 MHz, $CDCl_3$)

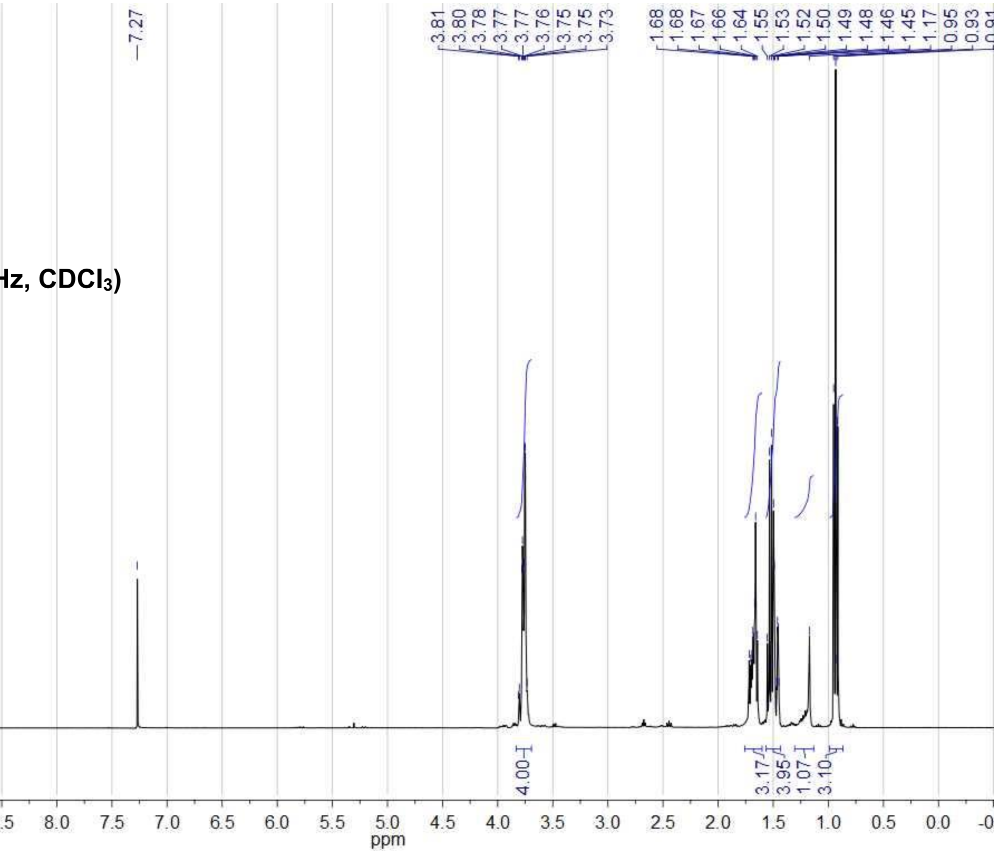

S8

^{13}C NMR (101 MHz, $CDCl_3$)

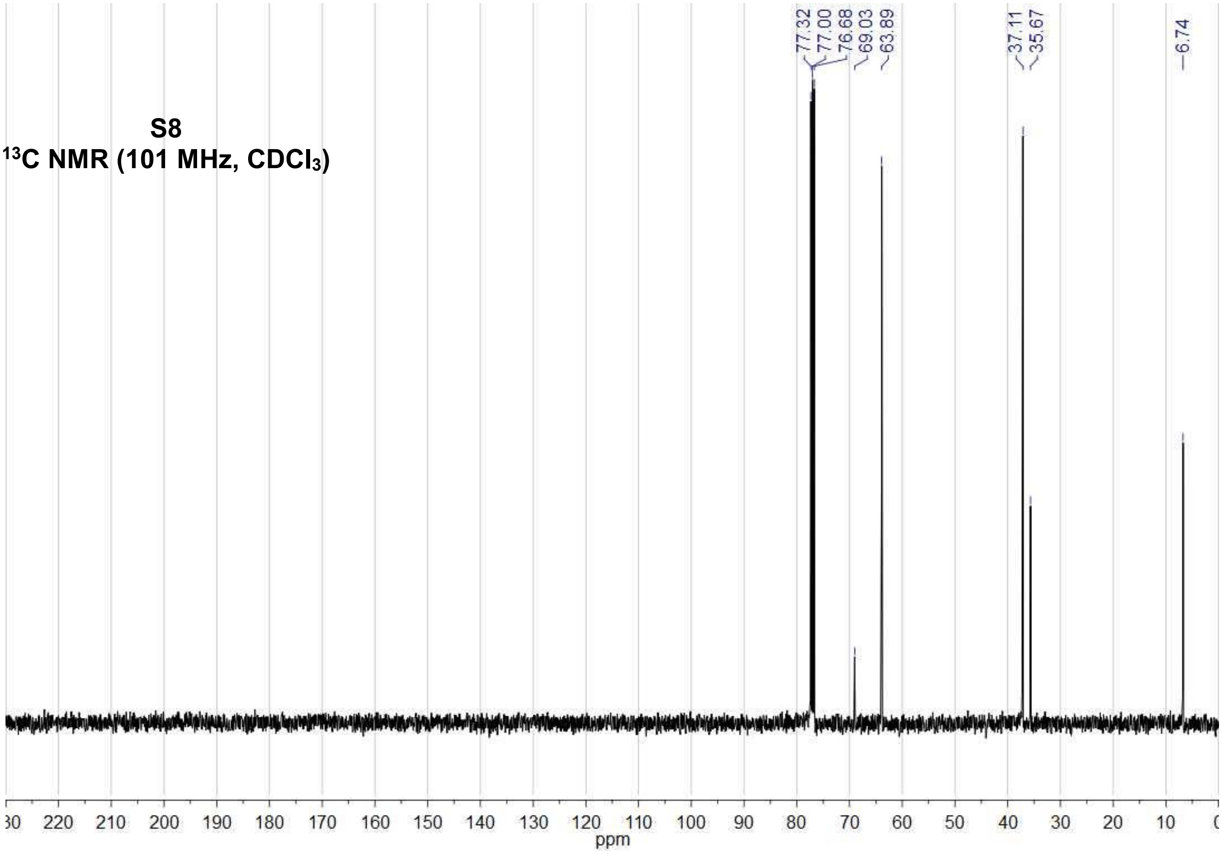

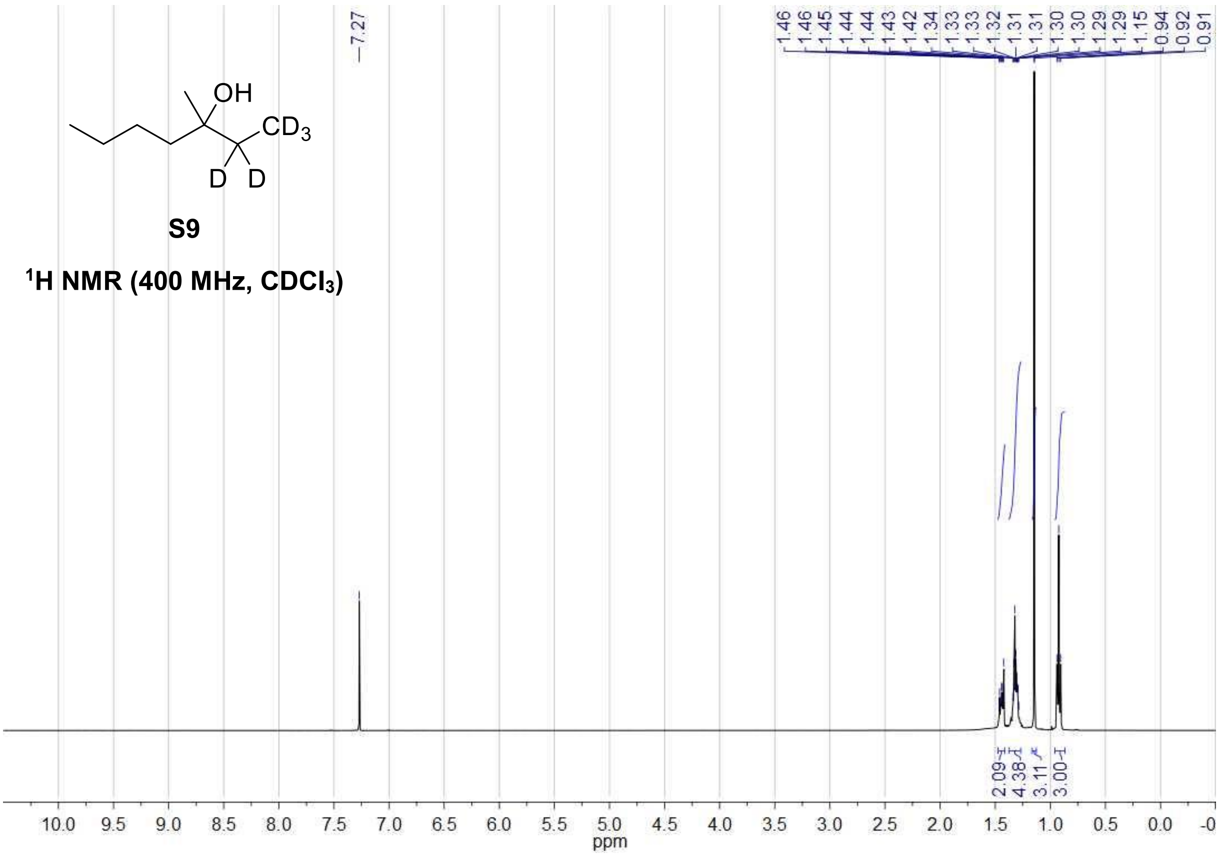
OH
CD3
D D
S9
1H NMR (400 MHz, CDCl3)
7.27
1.46 1.46 1.45 1.44 1.44 1.43 1.42 1.34 1.33 1.33 1.32 1.31 1.31 1.30 1.30 1.29 1.29 1.15 0.94 0.92 0.91
2.09 4.38 3.11 3.00
10.0 9.5 9.0 8.5 8.0 7.5 7.0 6.5 6.0 5.5 5.0 4.5 4.0 3.5 3.0 2.5 2.0 1.5 1.0 0.5 0.0
ppm

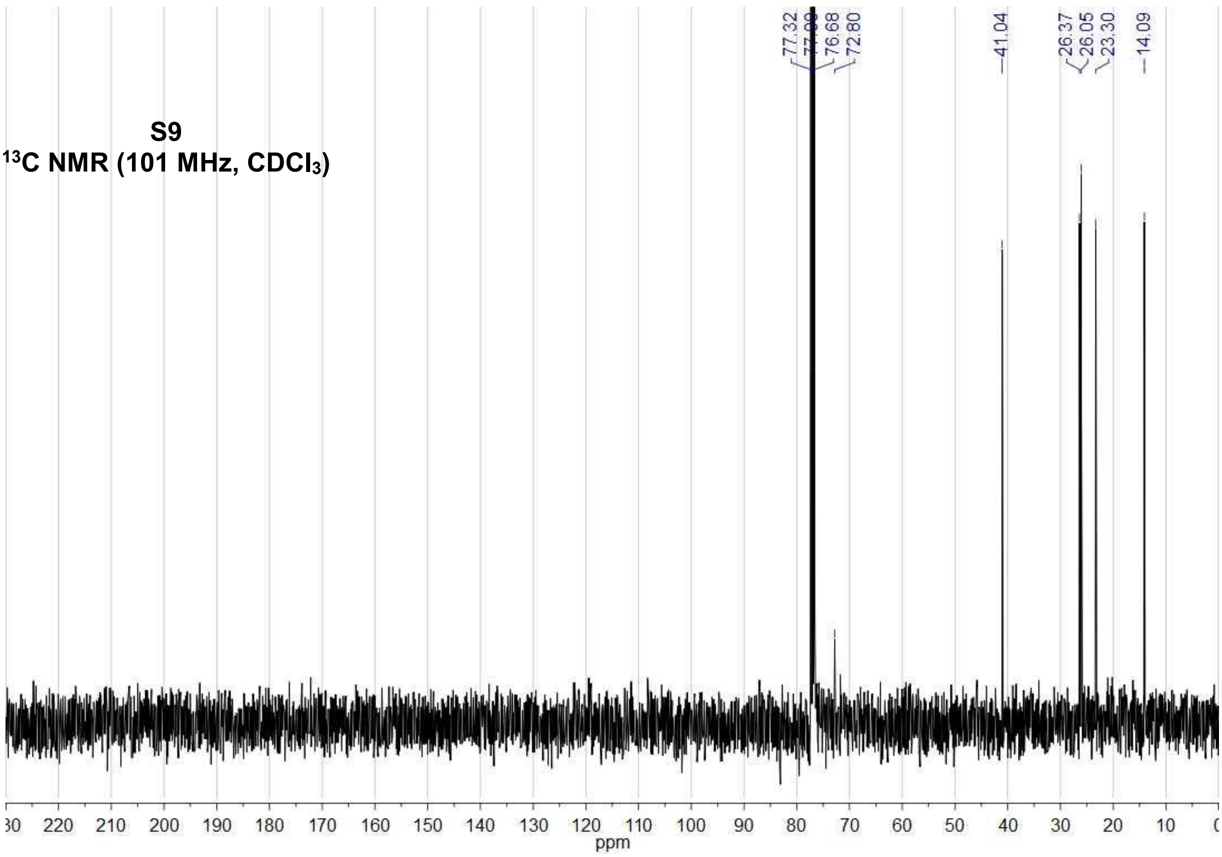
S9
13C NMR (101 MHz, CDCl3)
77.32 76.68 72.80
41.04
26.37 26.05 23.30
14.09
220 210 200 190 180 170 160 150 140 130 120 110 100 90 80 70 60 50 40 30 20 10
ppm

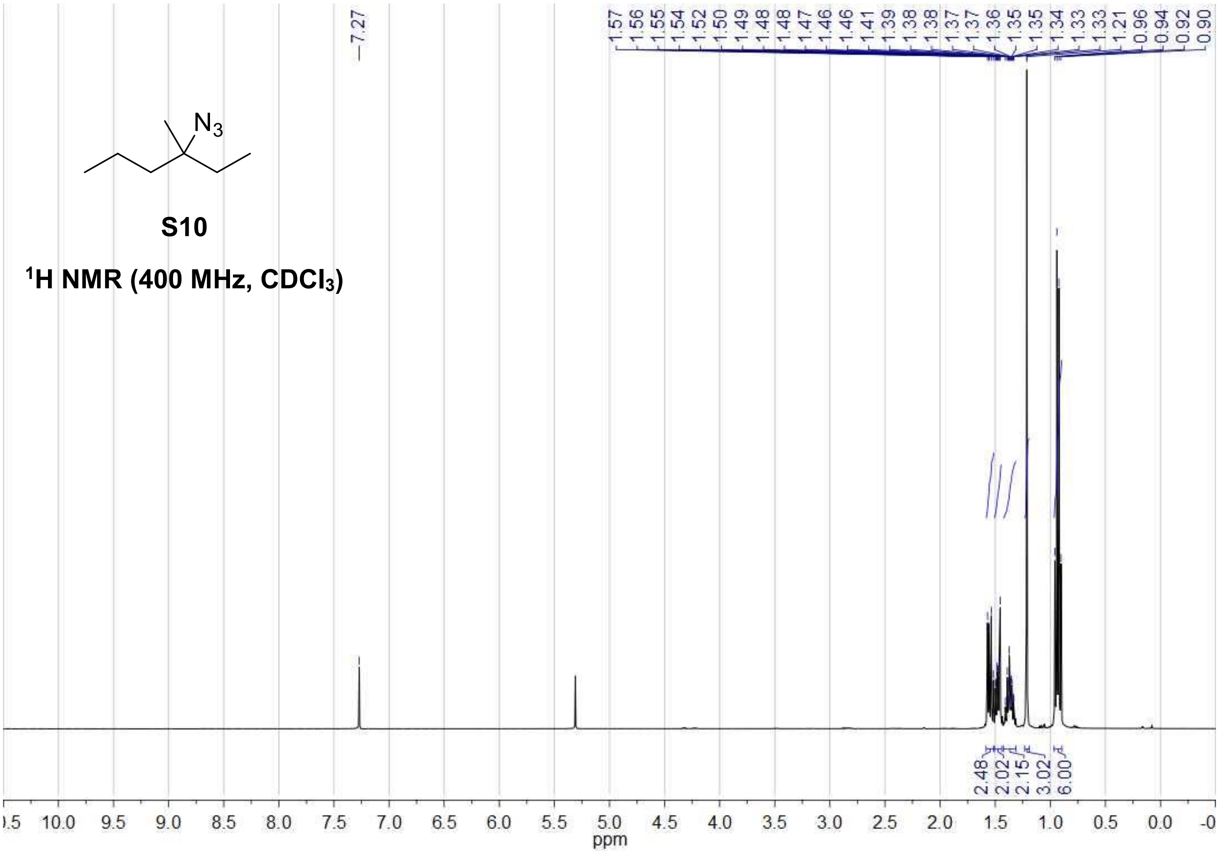

S10

^{1}H NMR (400 MHz, CDCl$_3$)

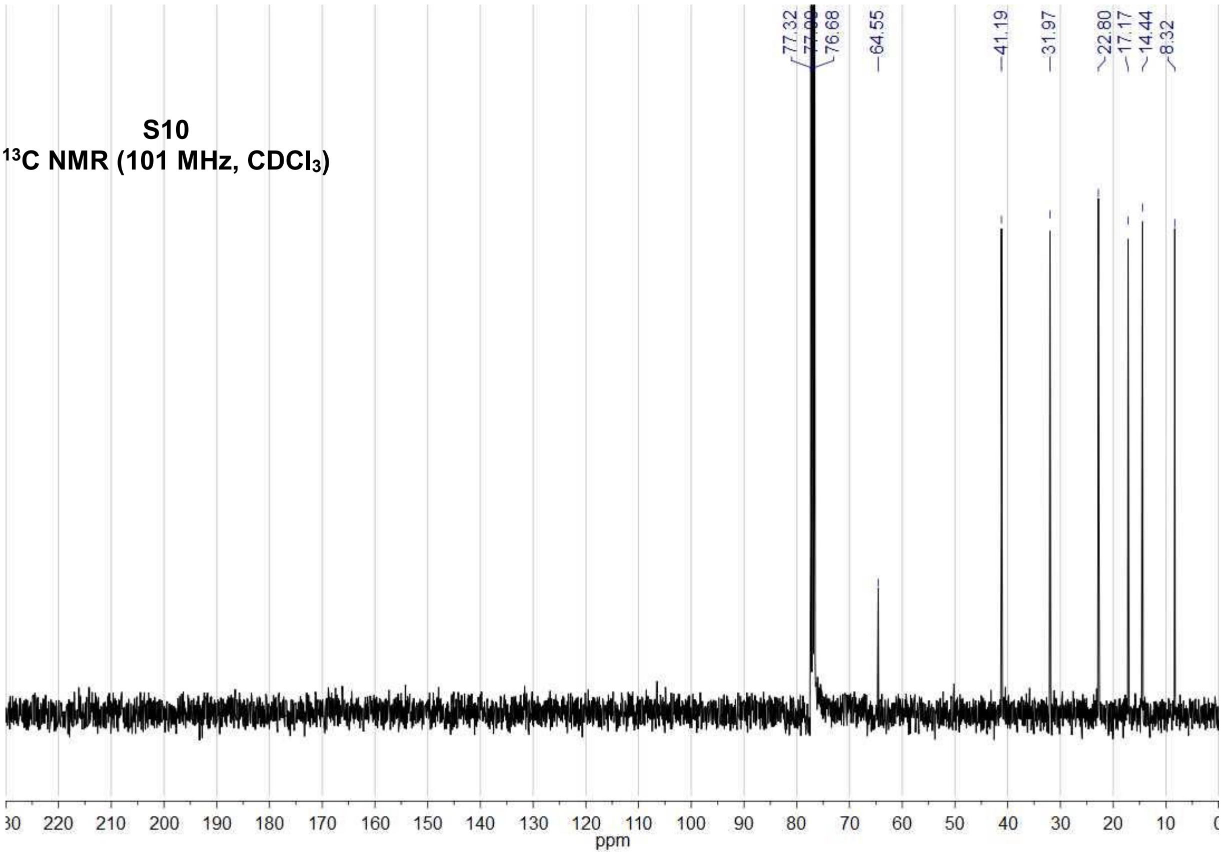

S10

^{13}C NMR (101 MHz, CDCl$_3$)

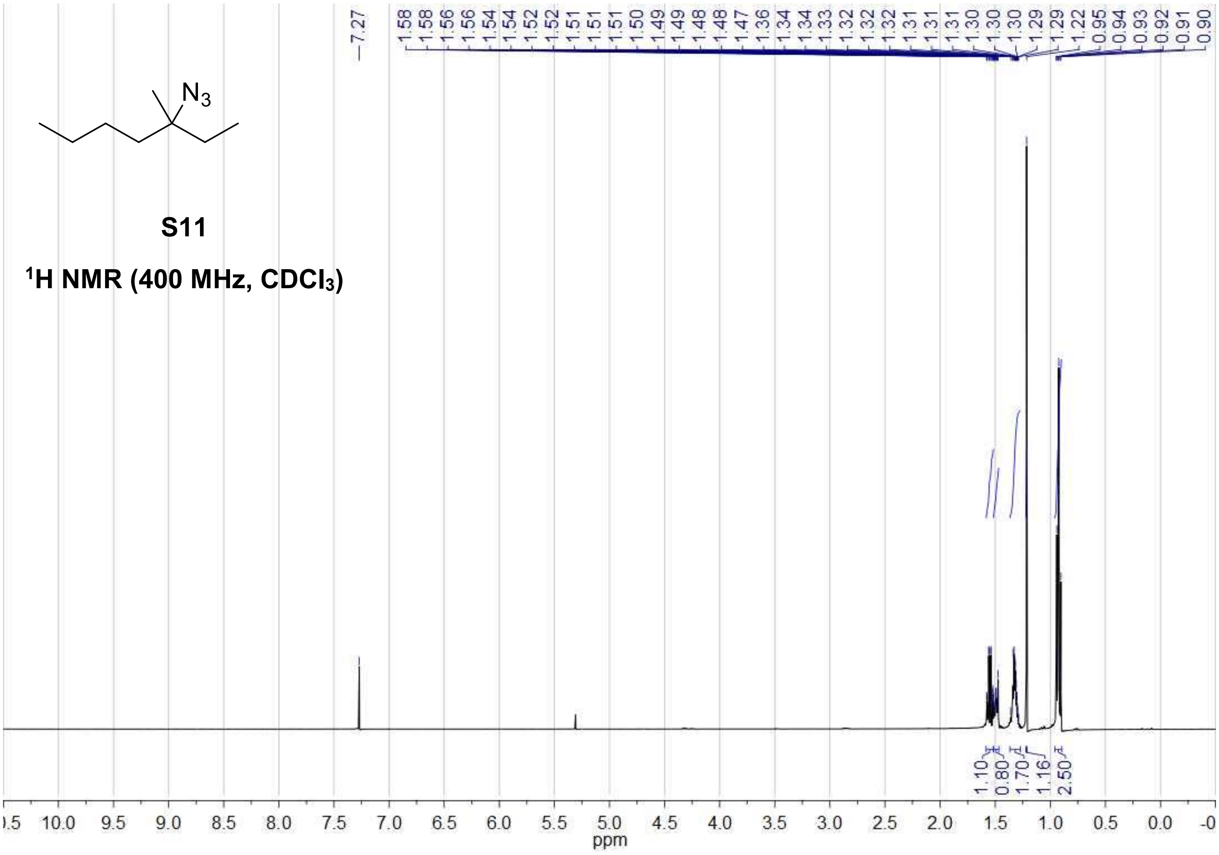

S11

^{1}H NMR (400 MHz, $CDCl_3$)

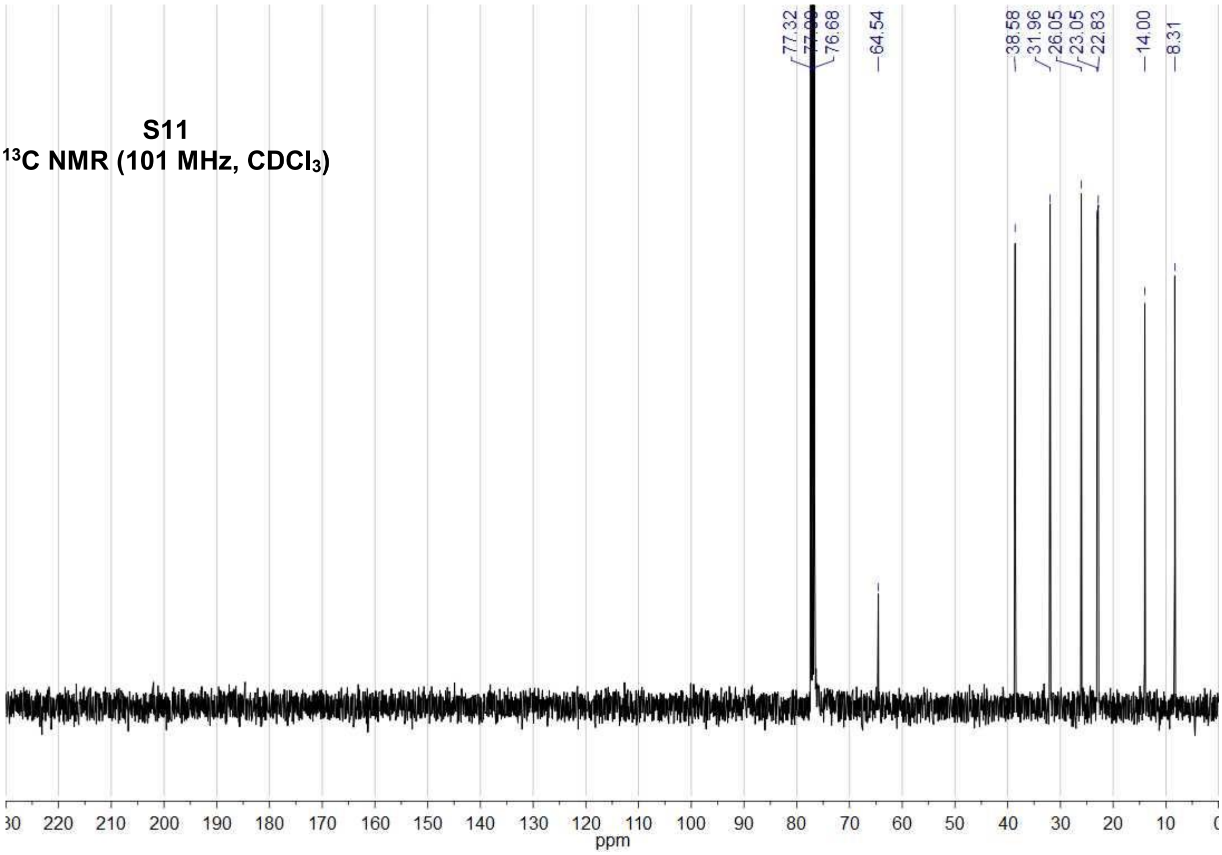

S11

^{13}C NMR (101 MHz, $CDCl_3$)

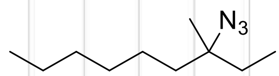

S12

^{1}H NMR (400 MHz, $CDCl_3$)

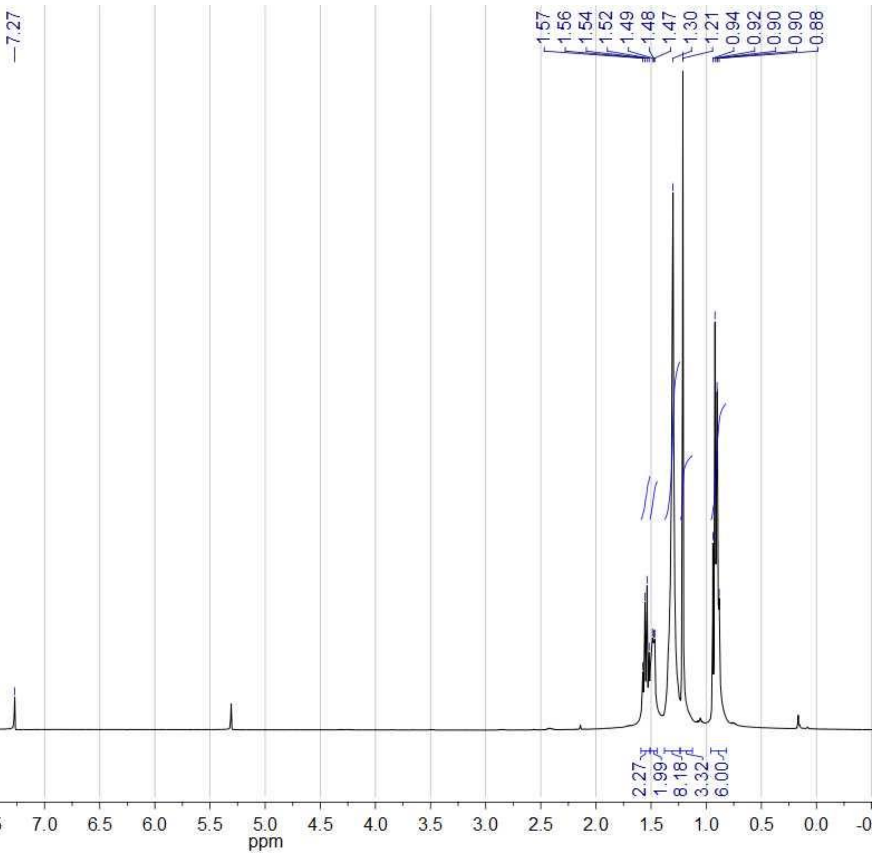

12

^{13}C NMR (101 MHz, $CDCl_3$)

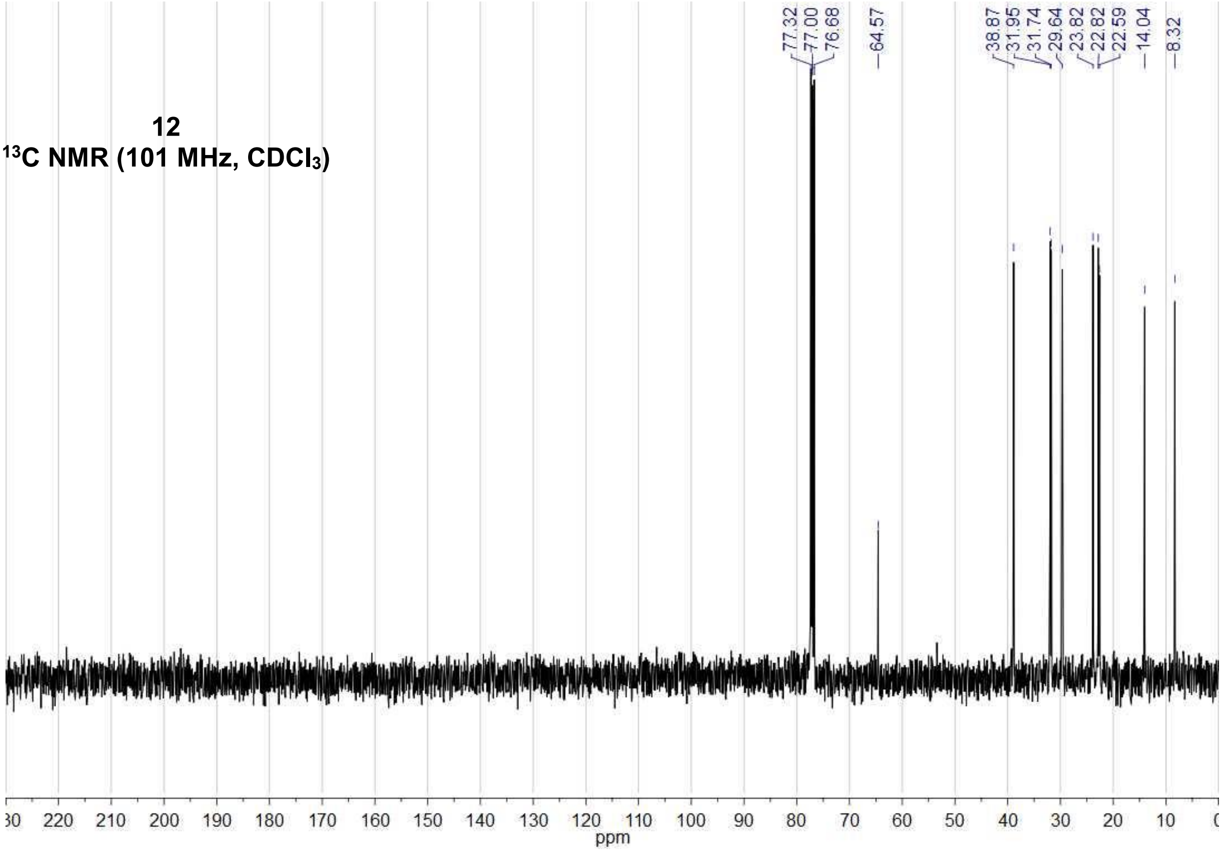

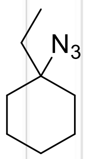

S13

^{1}H NMR (400 MHz, CDCl$_3$)

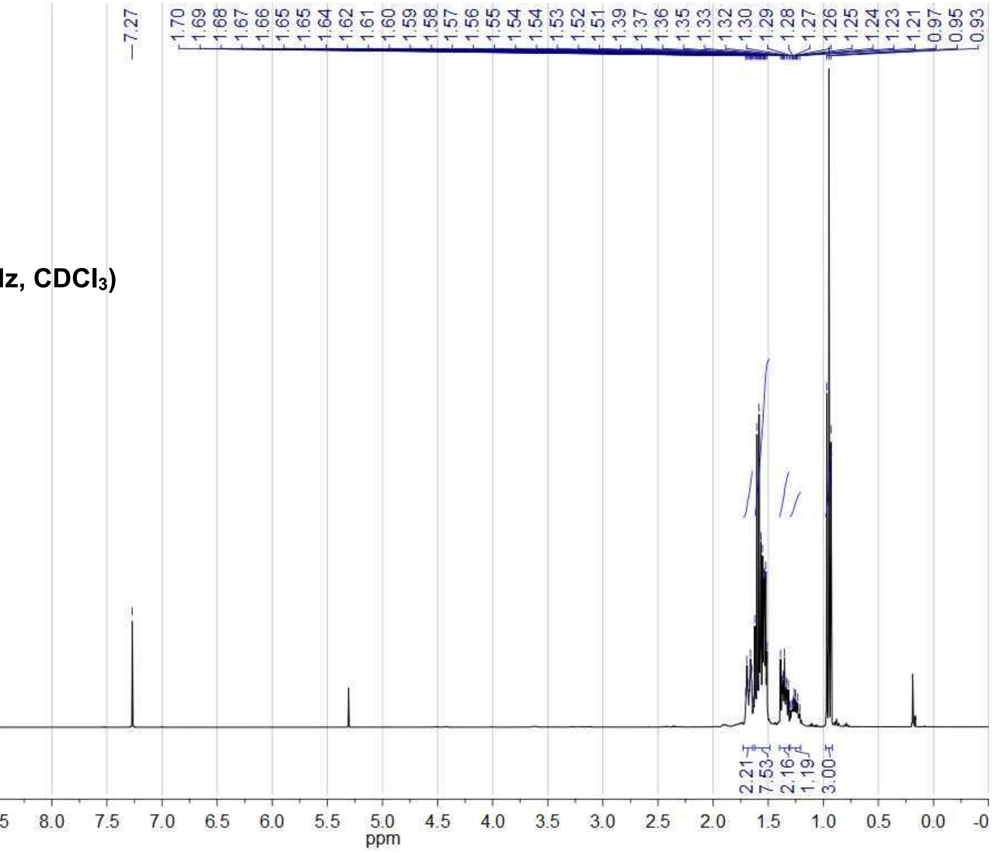

S13

^{13}C NMR (101 MHz, CDCl$_3$)

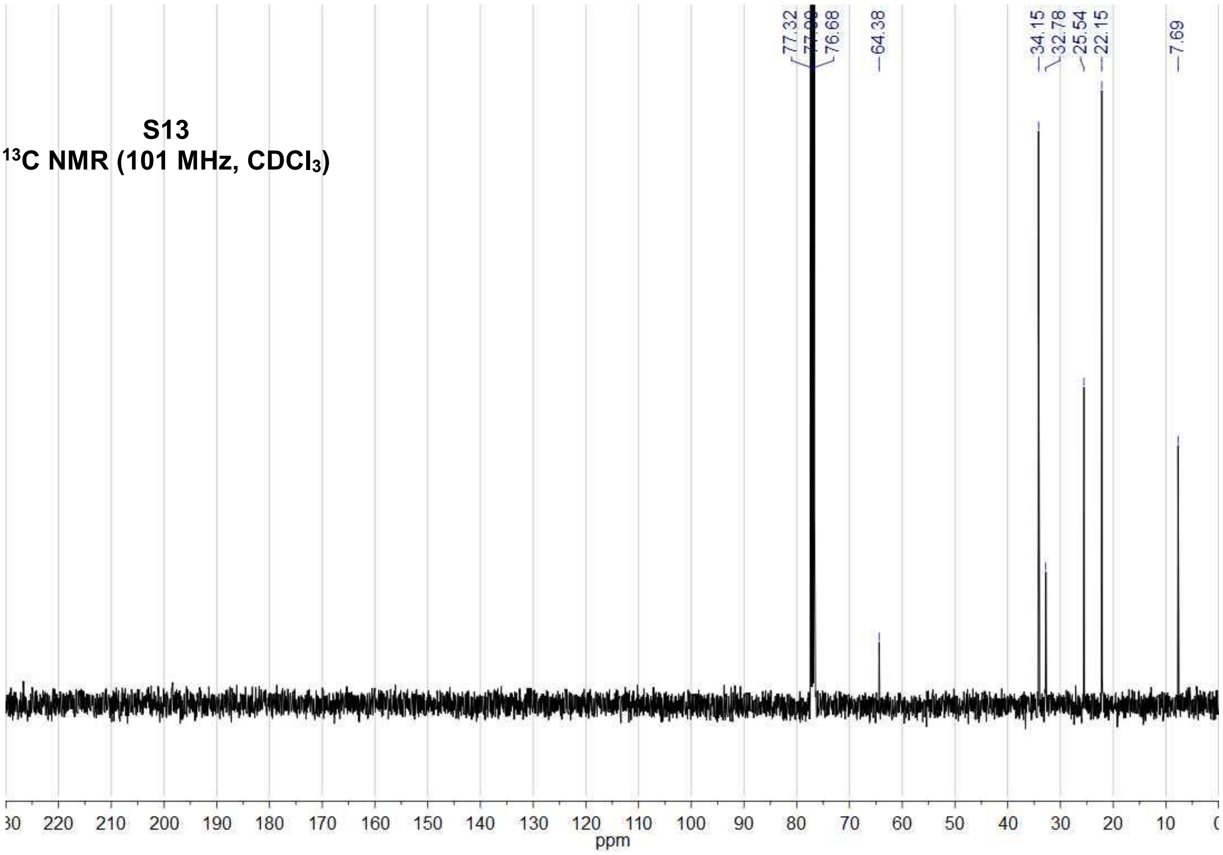

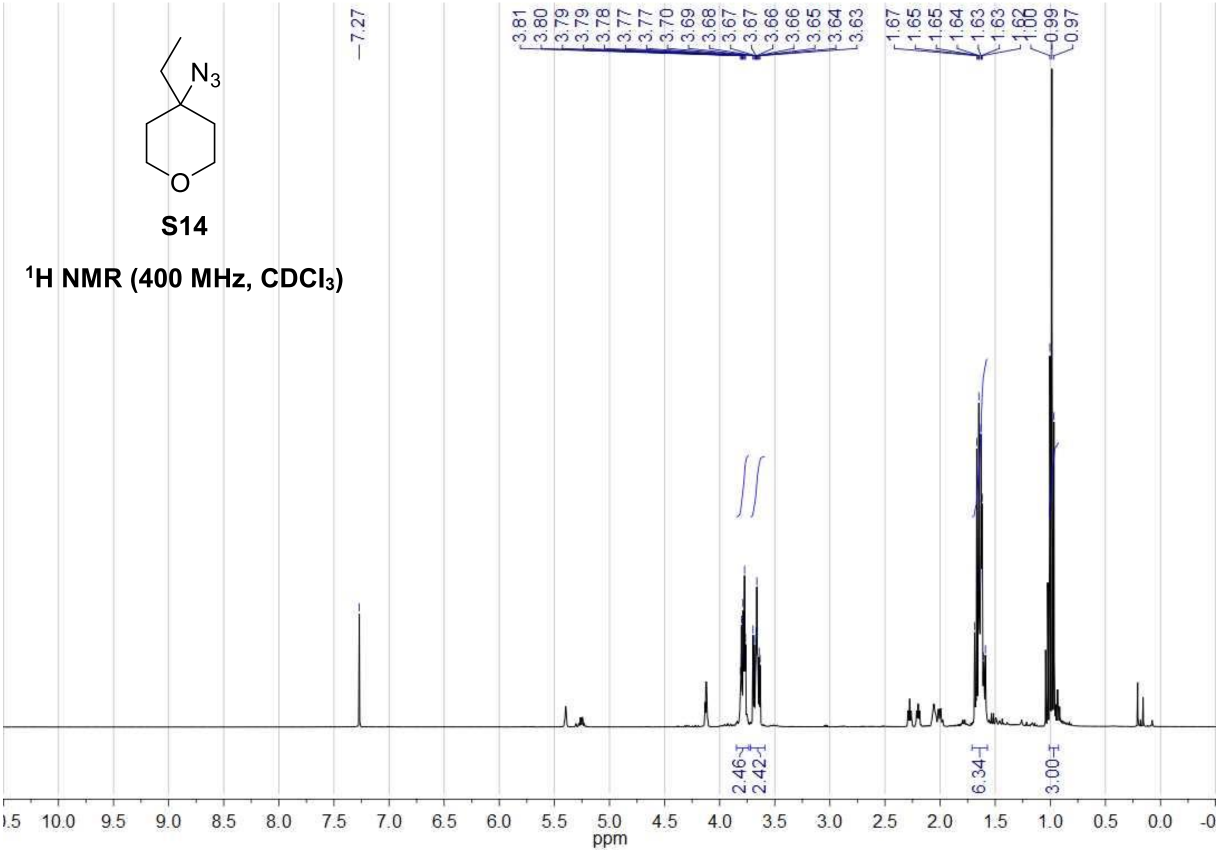

S14

^{1}H NMR (400 MHz, $CDCl_3$)

S14

^{13}C NMR (101 MHz, $CDCl_3$)

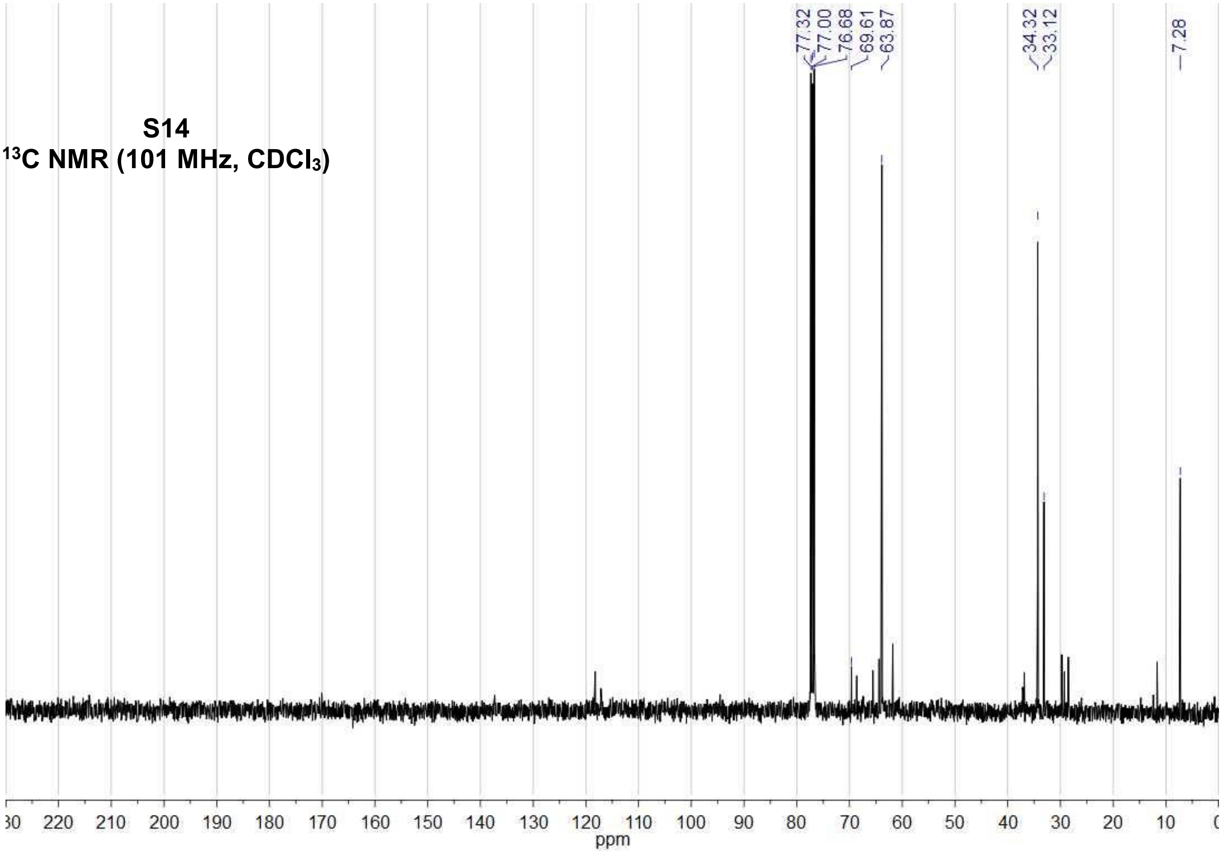

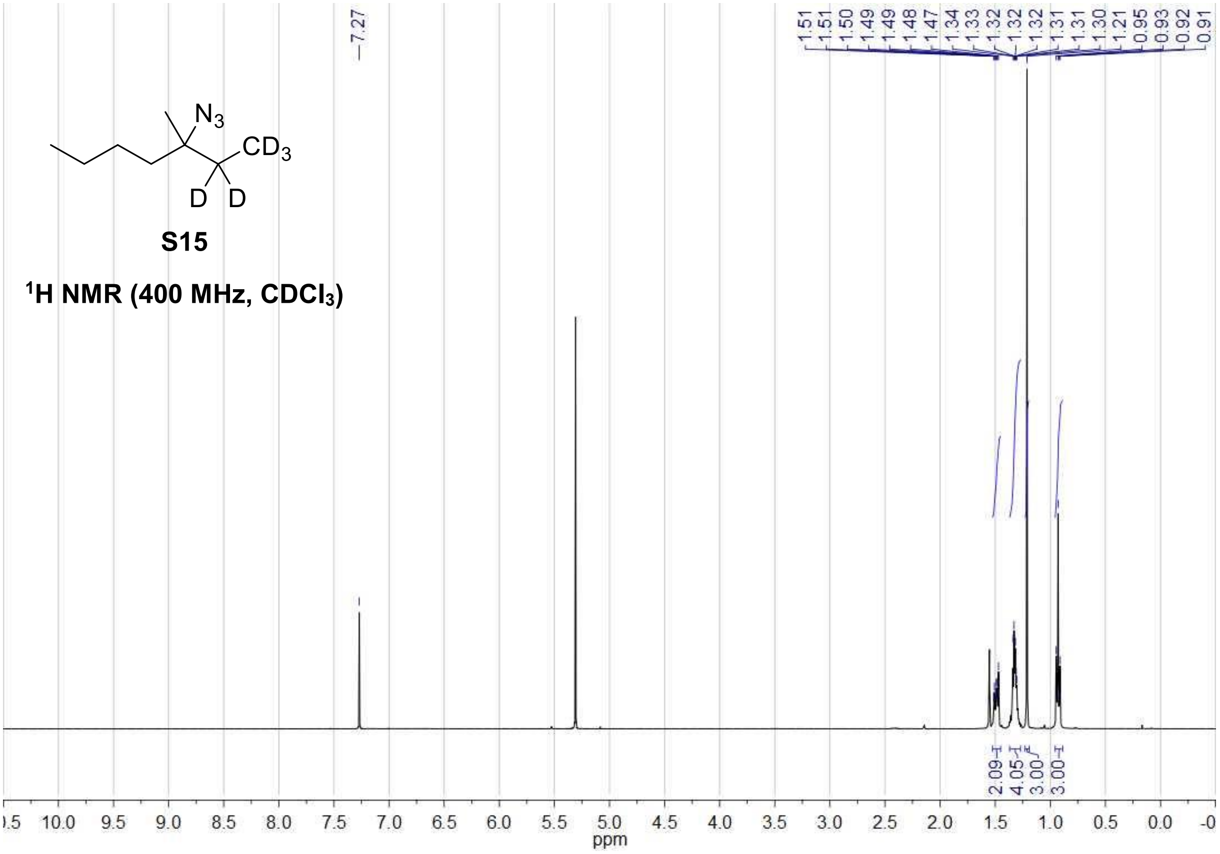
N3
CD3
D D
S15
1H NMR (400 MHz, CDCl3)
7.27
1.51 1.51 1.50 1.49 1.49 1.48 1.47 1.34 1.33 1.32 1.32 1.32 1.32 1.31 1.31 1.30 1.21 0.95 0.93 0.92 0.91
2.09 4.05 3.00 3.00
ppm

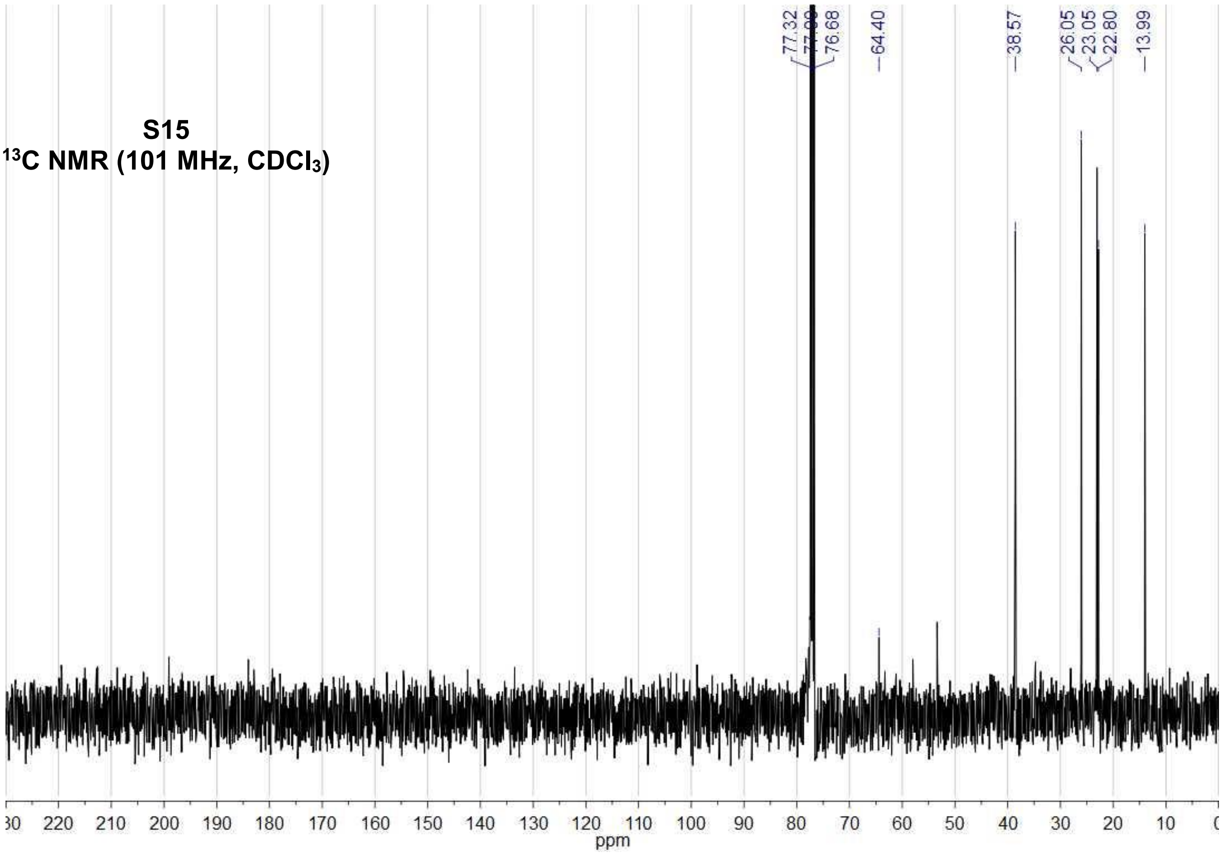
S15
13C NMR (101 MHz, CDCl3)
77.32 76.68
64.40
38.57
26.05 23.05 22.80
13.99
ppm

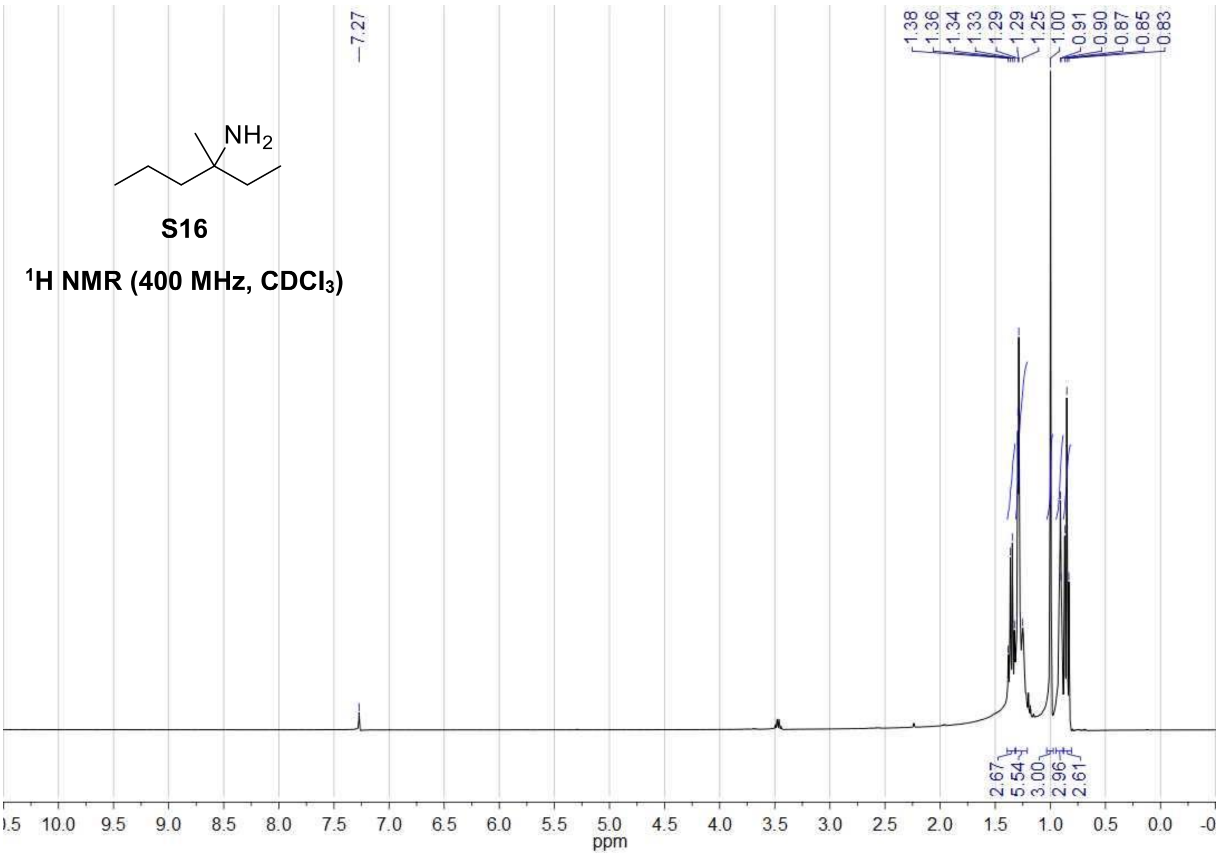
NH2
S16
1H NMR (400 MHz, CDCl3)
7.27
1.38 1.36 1.34 1.33 1.29 1.29 1.25 1.00 0.91 0.90 0.87 0.85 0.83
2.67 5.54 3.00 2.96 2.61
ppm

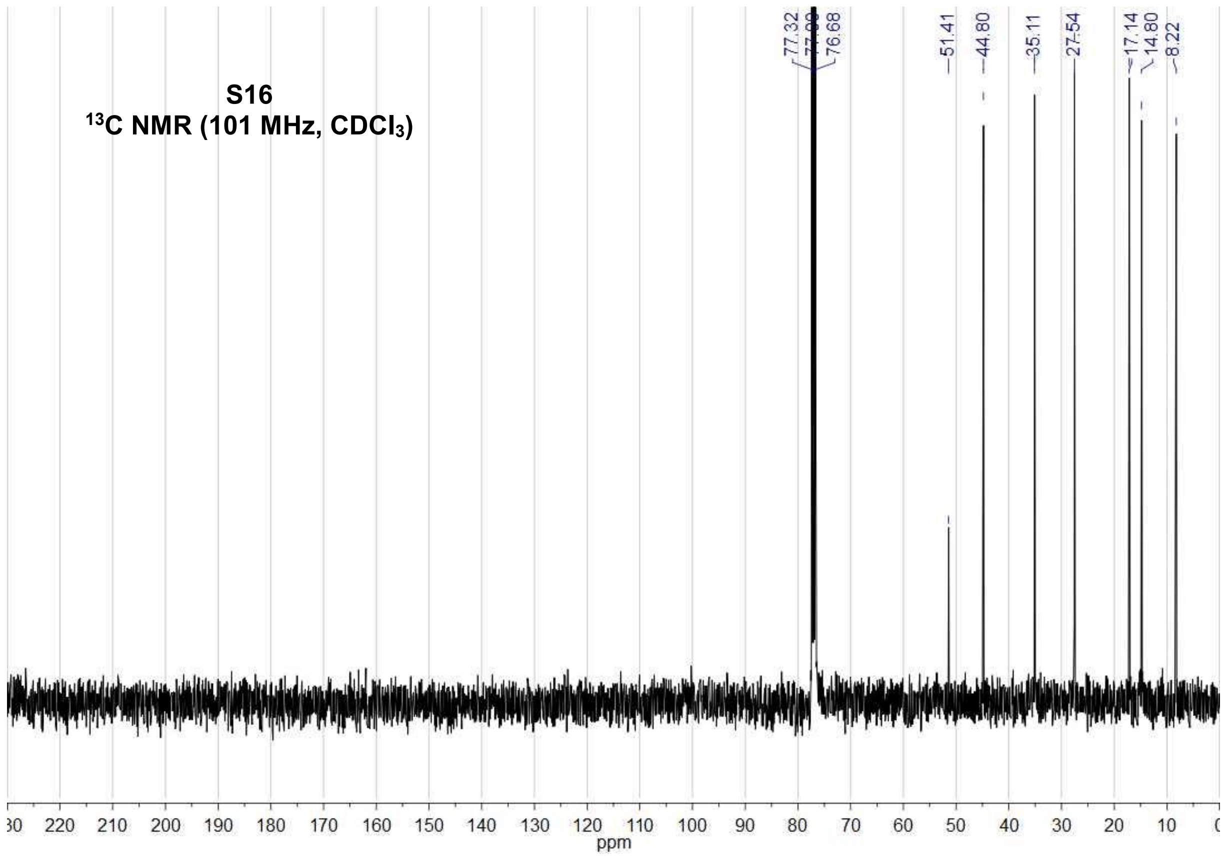
S16
13C NMR (101 MHz, CDCl3)
77.32 77.00 76.68
51.41
44.80
35.11
27.54
17.14
14.80
8.22
ppm

30

^{1}H NMR (400 MHz, $CDCl_3$)

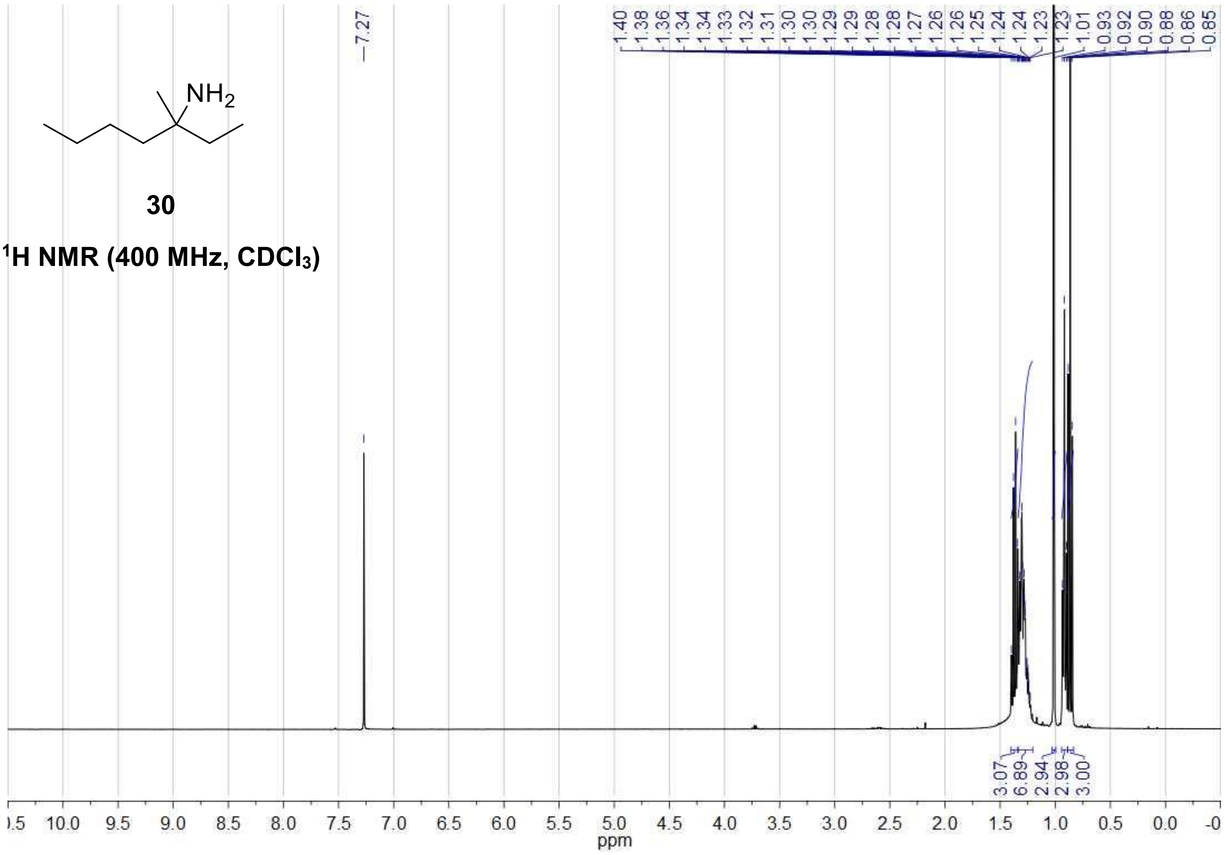

30

^{13}C NMR (101 MHz, $CDCl_3$)

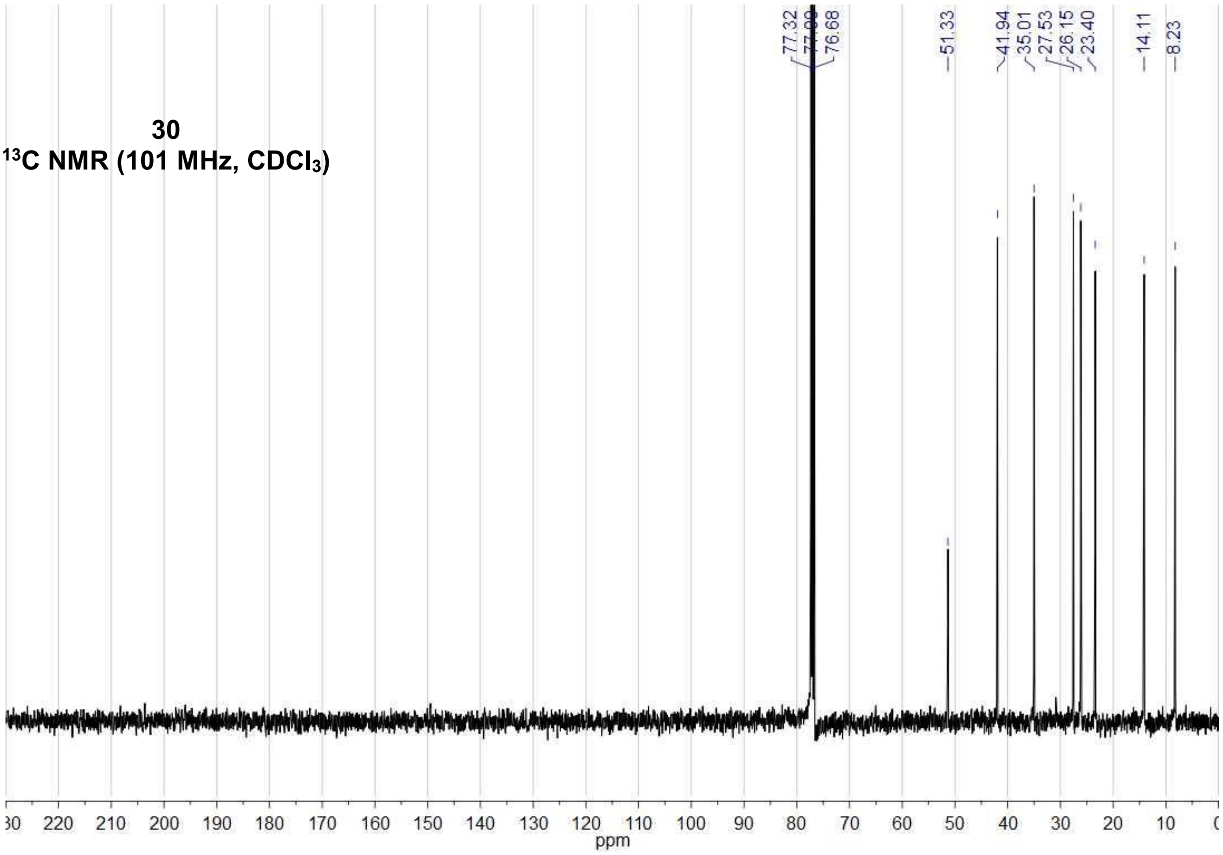

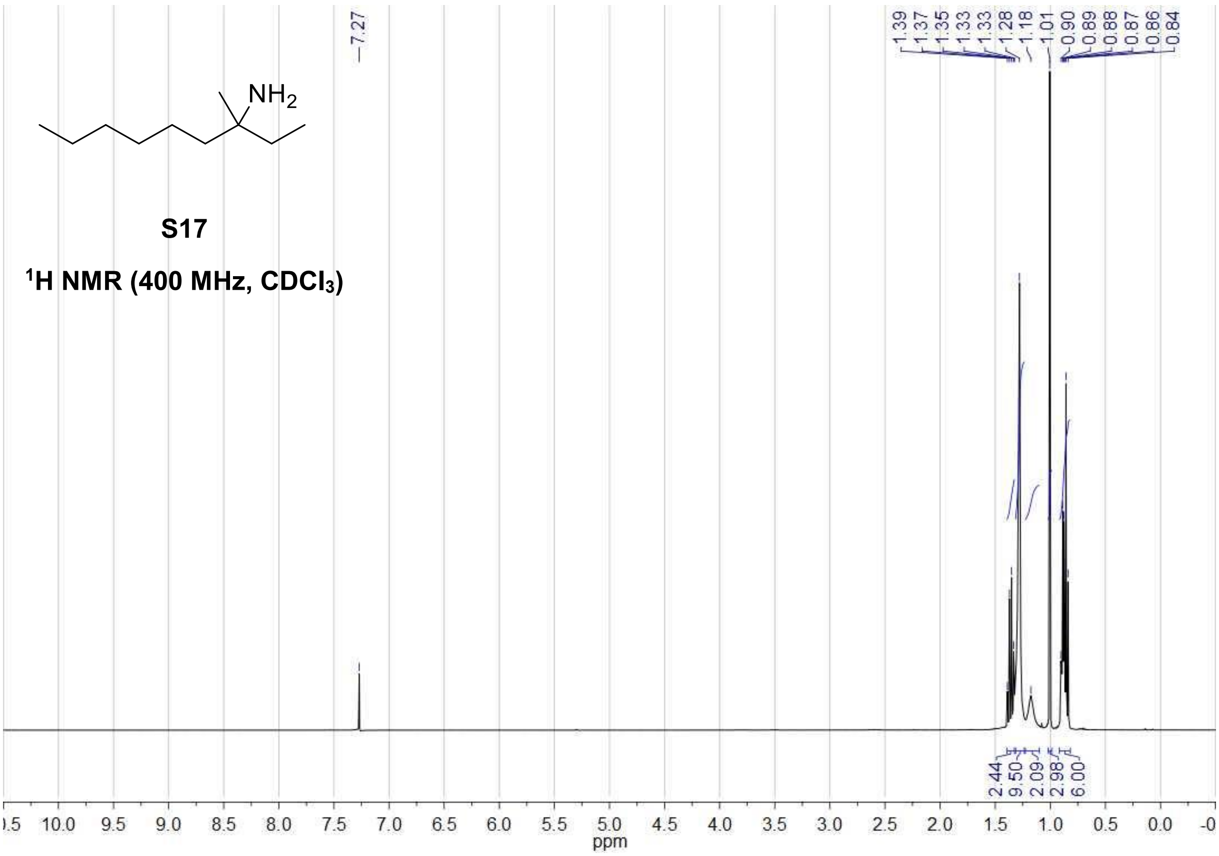
S17
^{1}H NMR (400 MHz, CDCl$_3$)
NH$_2$
7.27
1.39 1.37 1.35 1.33 1.33 1.28 1.18 1.01 0.90 0.89 0.88 0.87 0.86 0.84
2.44 9.50 2.09 2.98 6.00
ppm

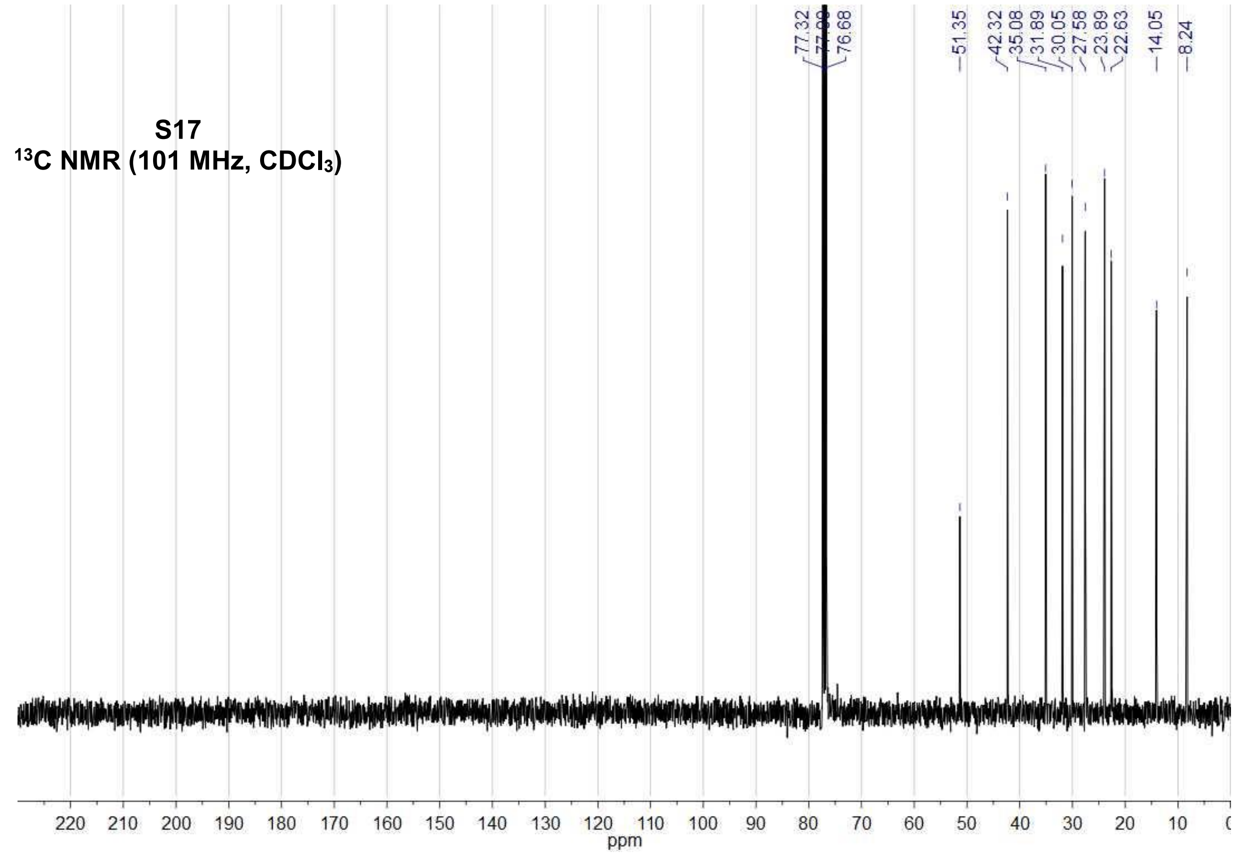
S17
^{13}C NMR (101 MHz, CDCl$_3$)
77.32 76.68
51.35 42.32 35.08 31.89 30.05 27.58 23.89 22.63 14.05 8.24
ppm

S18

^{1}H NMR (400 MHz, CDCl$_3$)

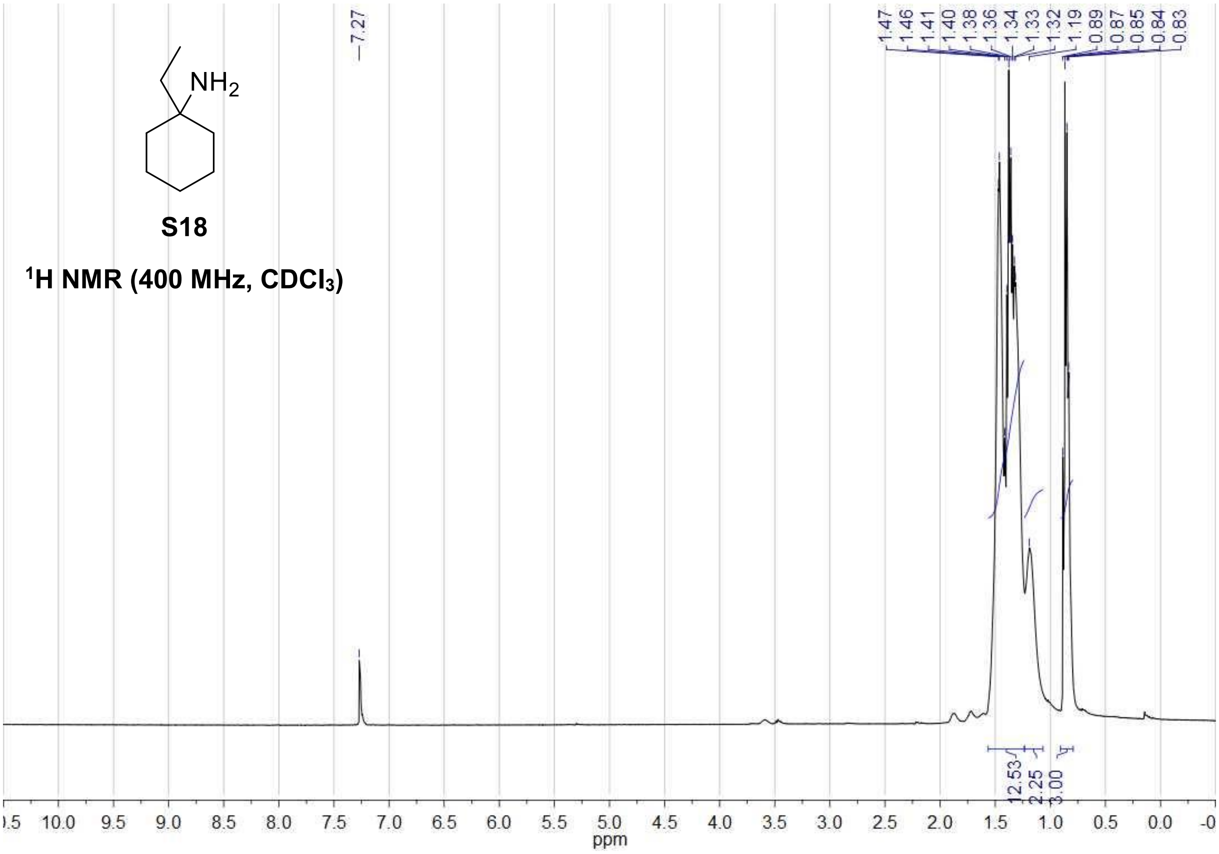

S18

^{13}C NMR (101 MHz, CDCl$_3$)

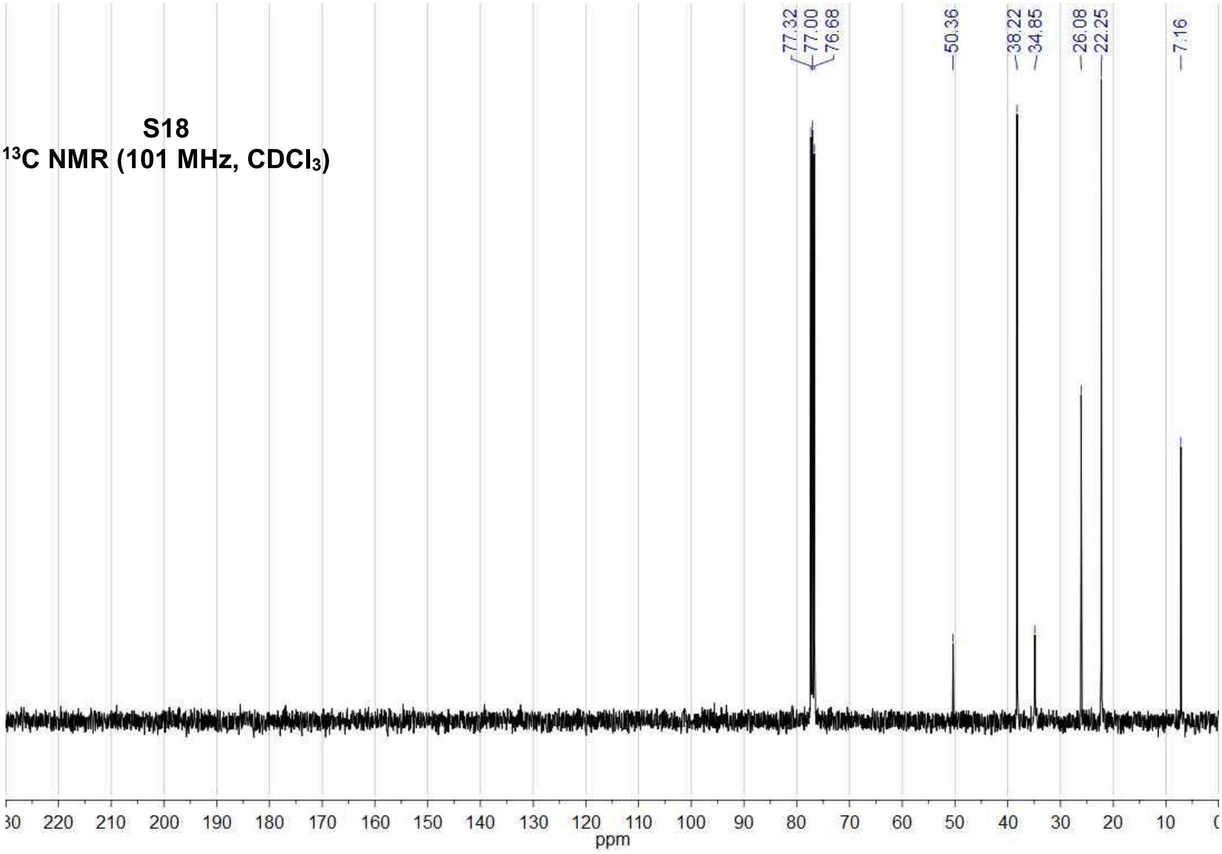

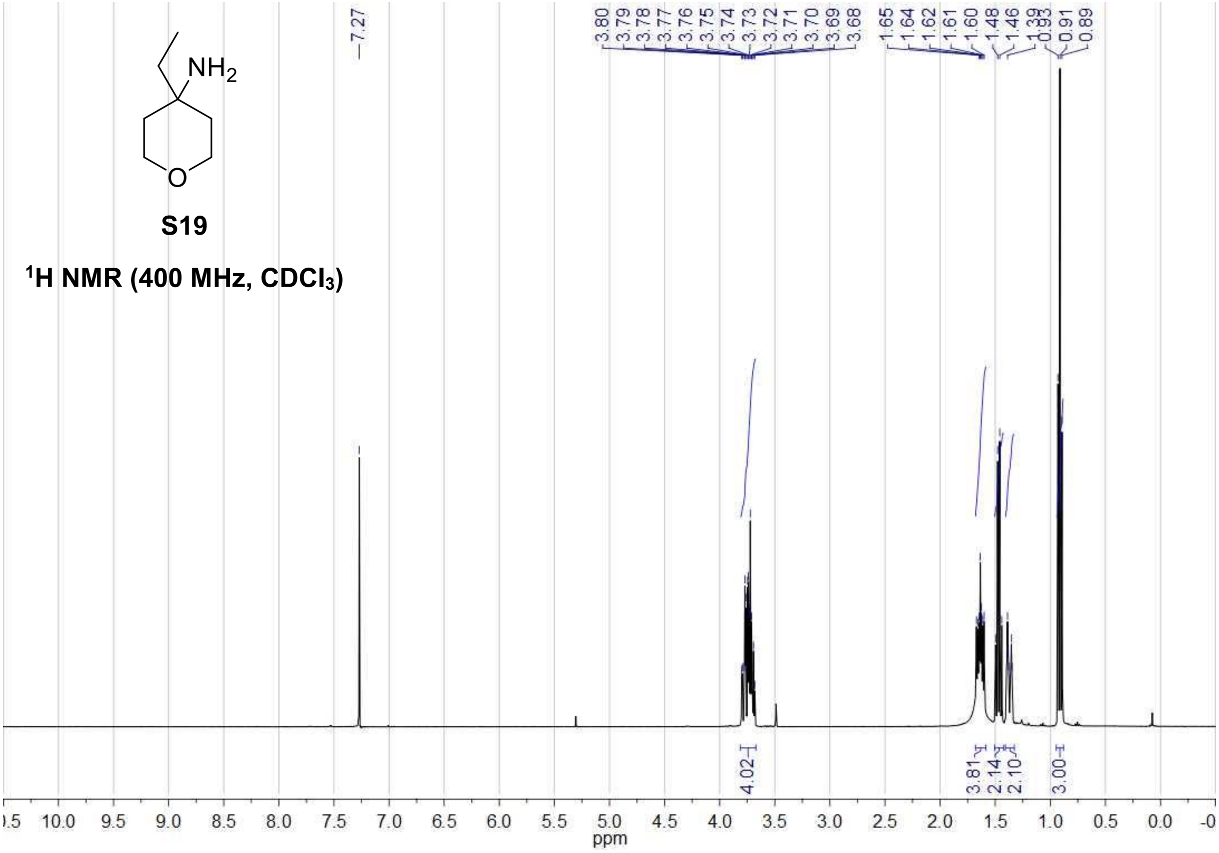

S19

^{1}H NMR (400 MHz, $CDCl_3$)

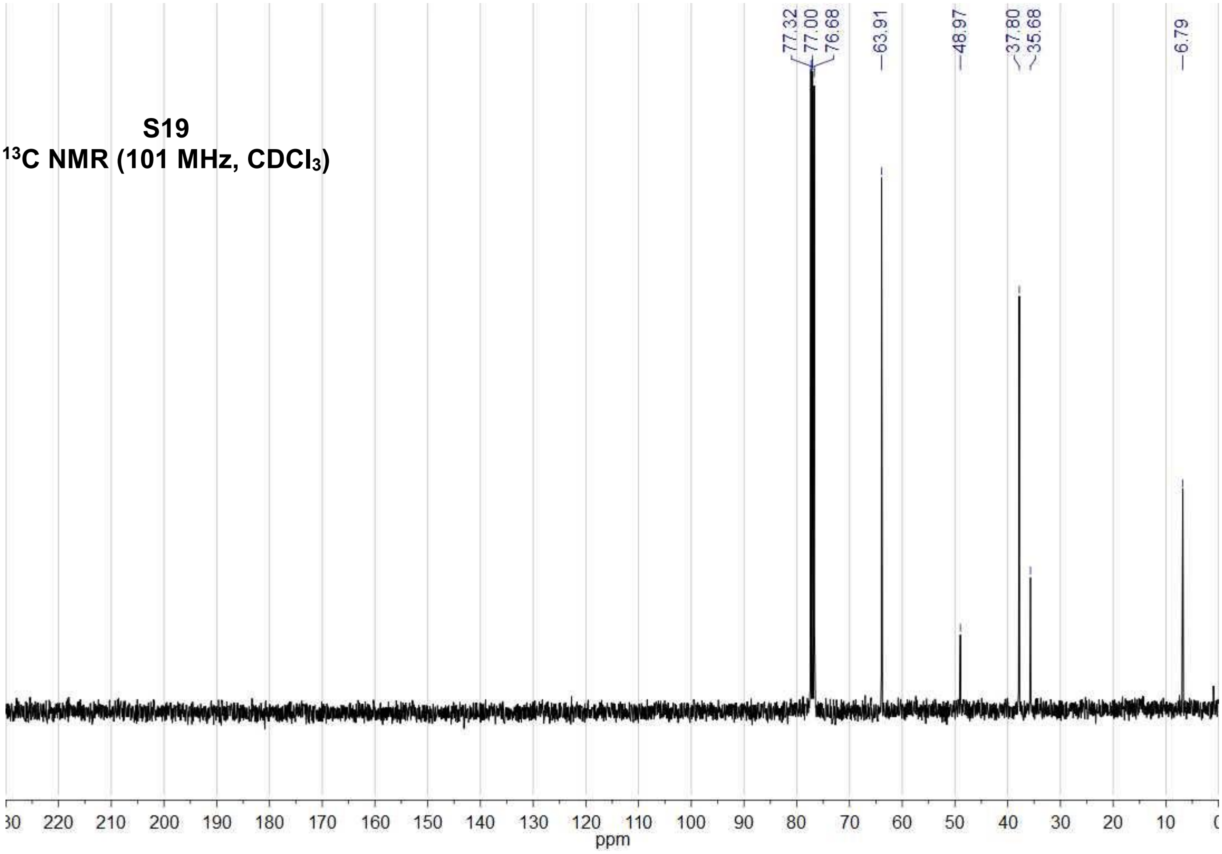

S19

^{13}C NMR (101 MHz, $CDCl_3$)

D5-30

^{1}H NMR (400 MHz, CDCl3)

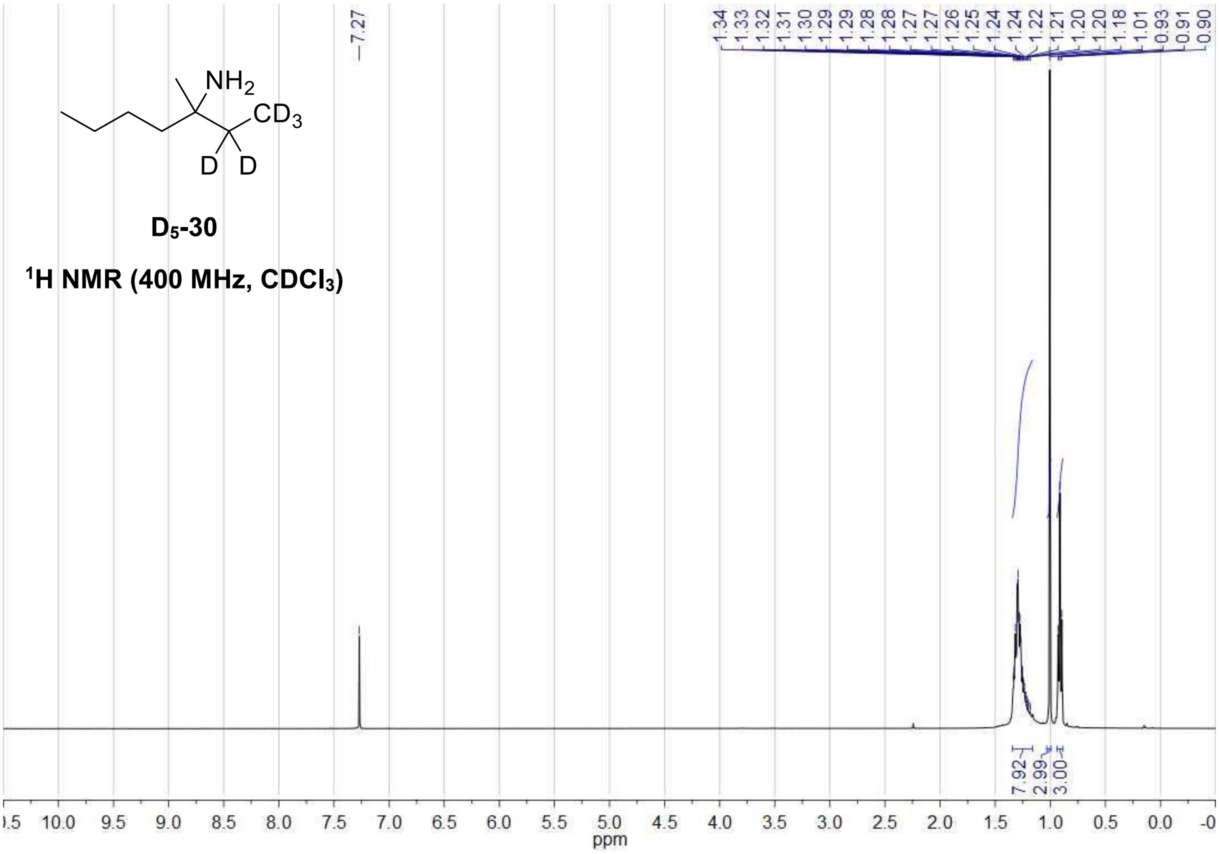

D5-30

^{13}C NMR (101 MHz, CDCl3)

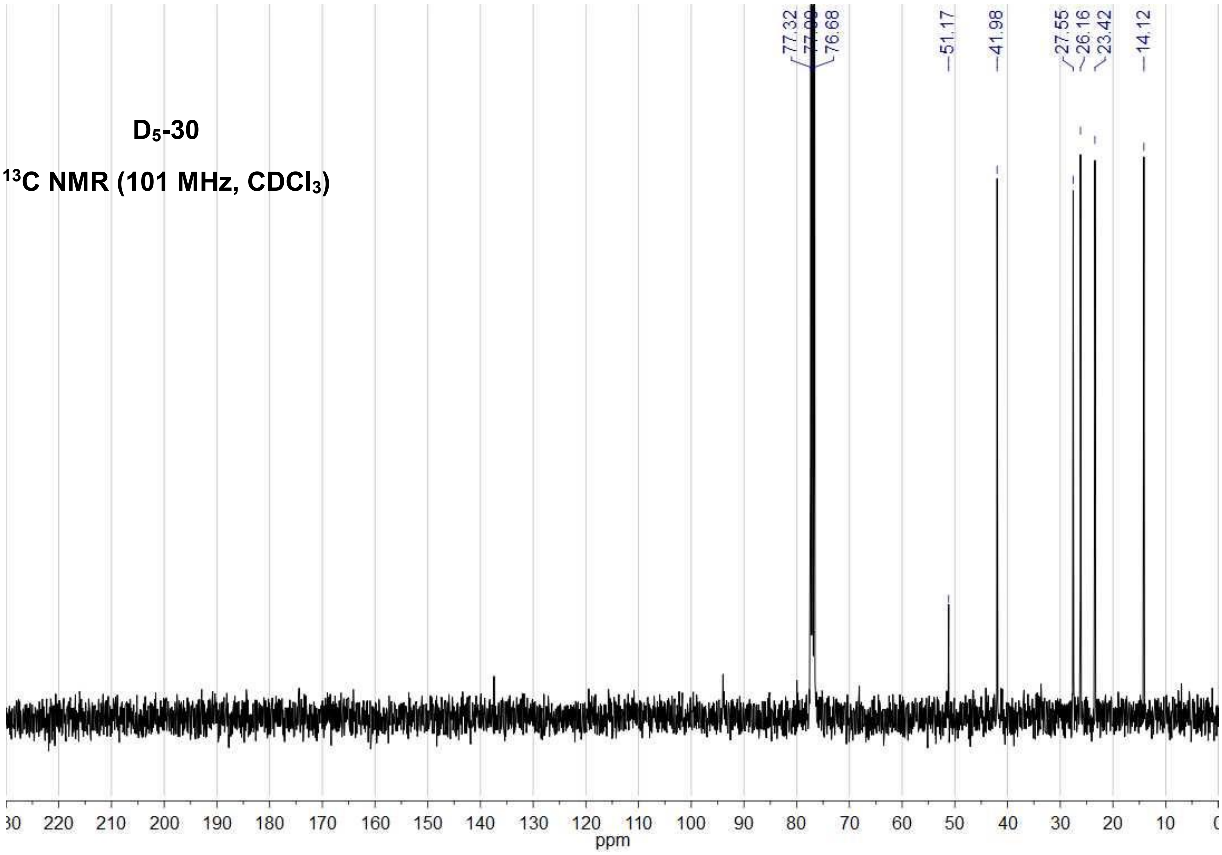

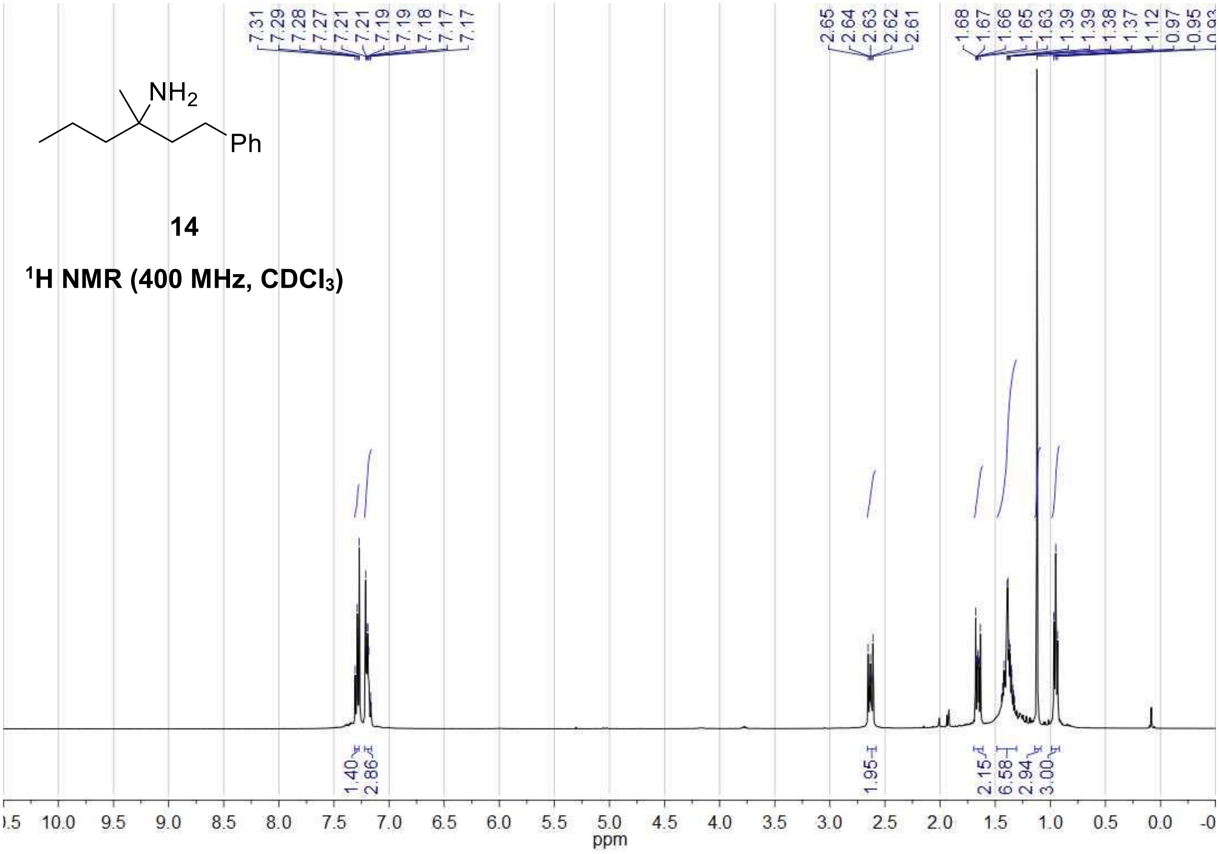

14

^{1}H NMR (400 MHz, $CDCl_3$)

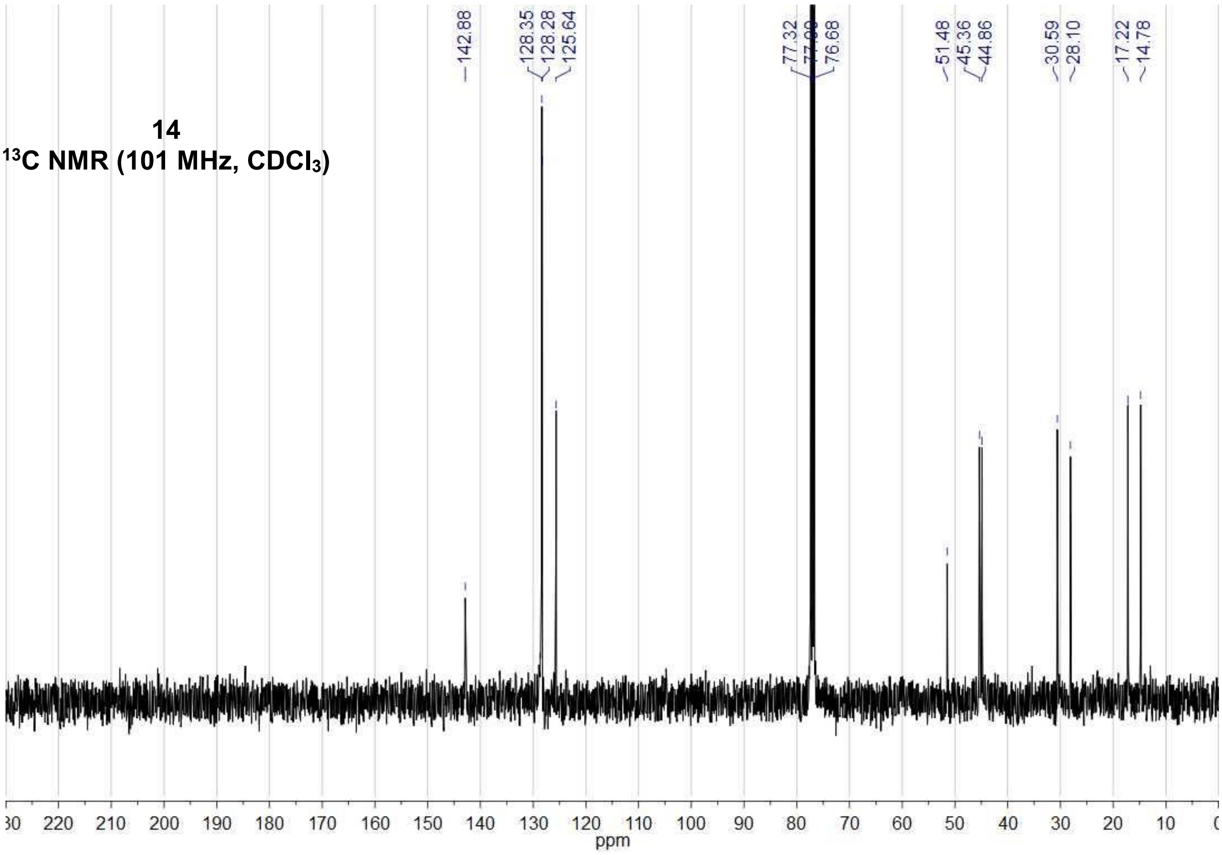

14

^{13}C NMR (101 MHz, $CDCl_3$)

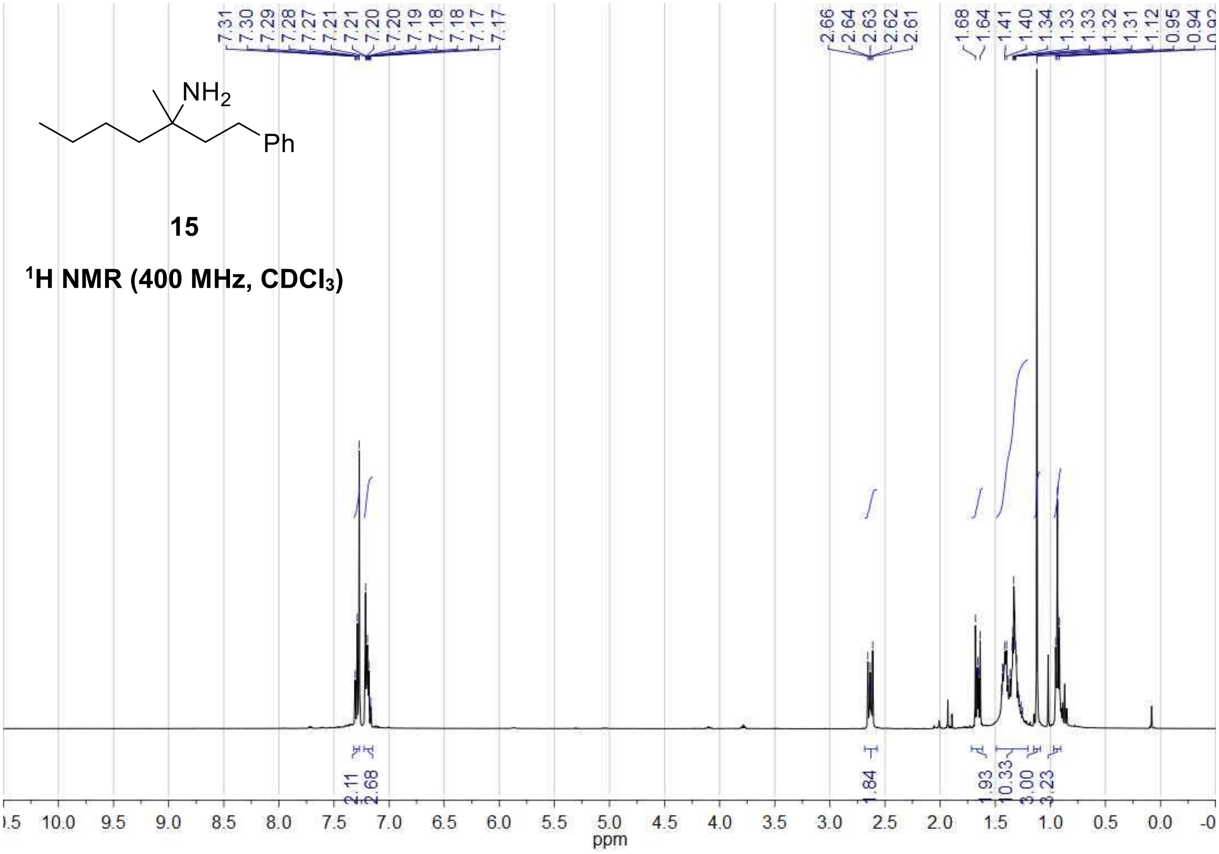

15

^{1}H NMR (400 MHz, $CDCl_3$)

15

^{13}C NMR (101 MHz, $CDCl_3$)

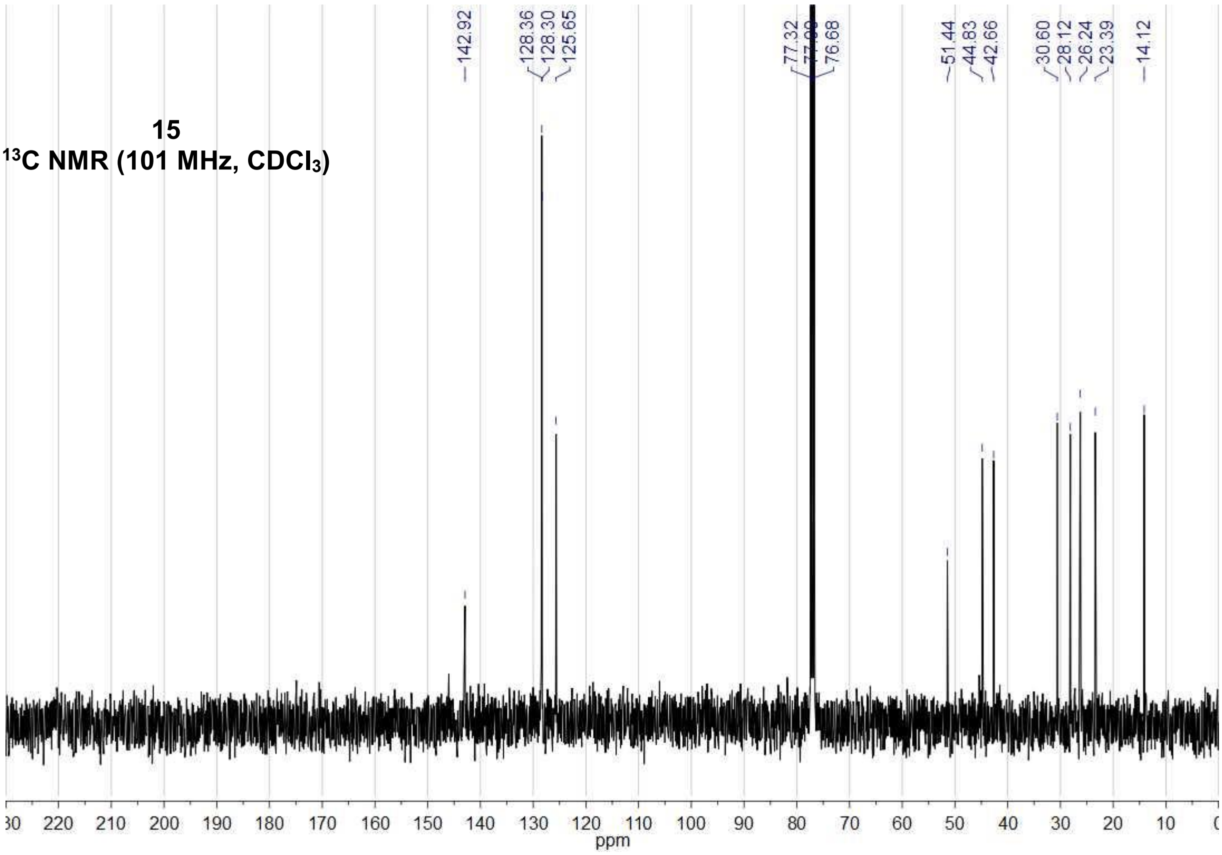

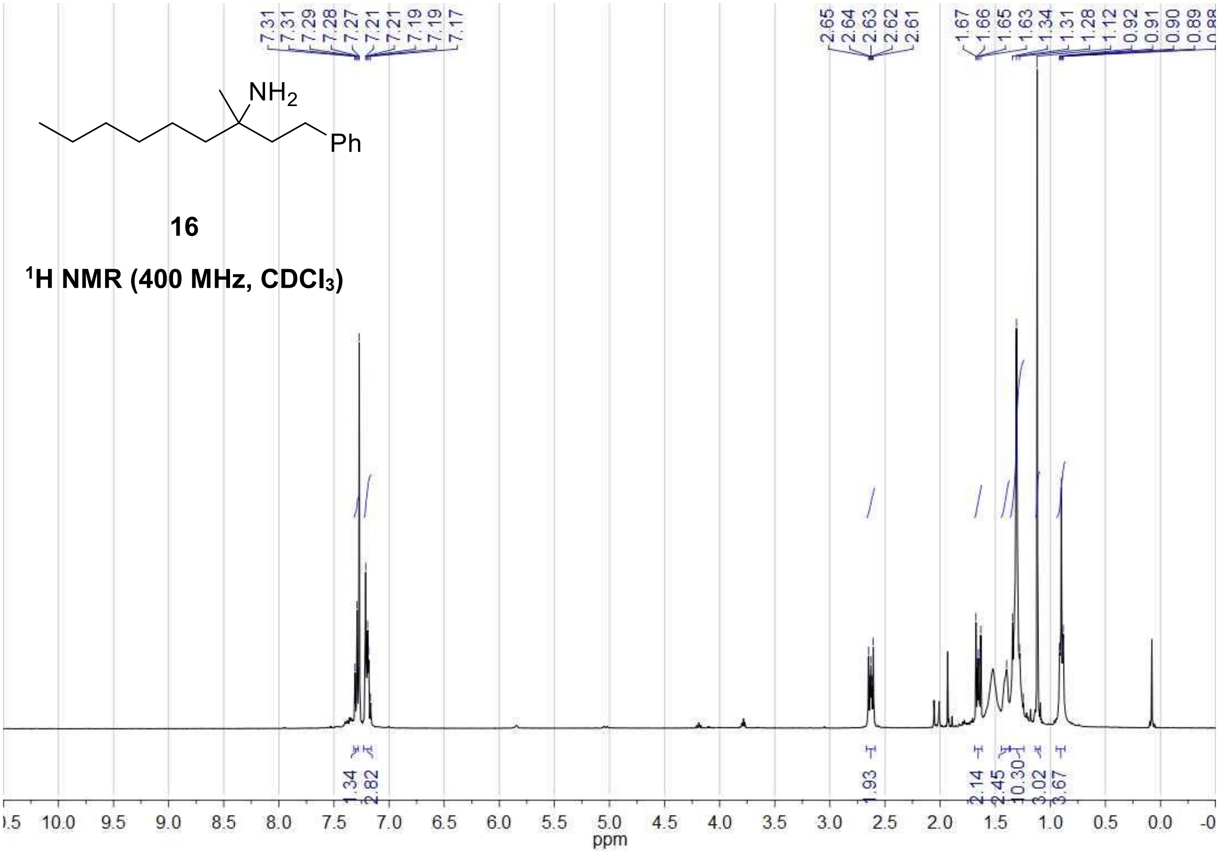

16

^{1}H NMR (400 MHz, $CDCl_3$)

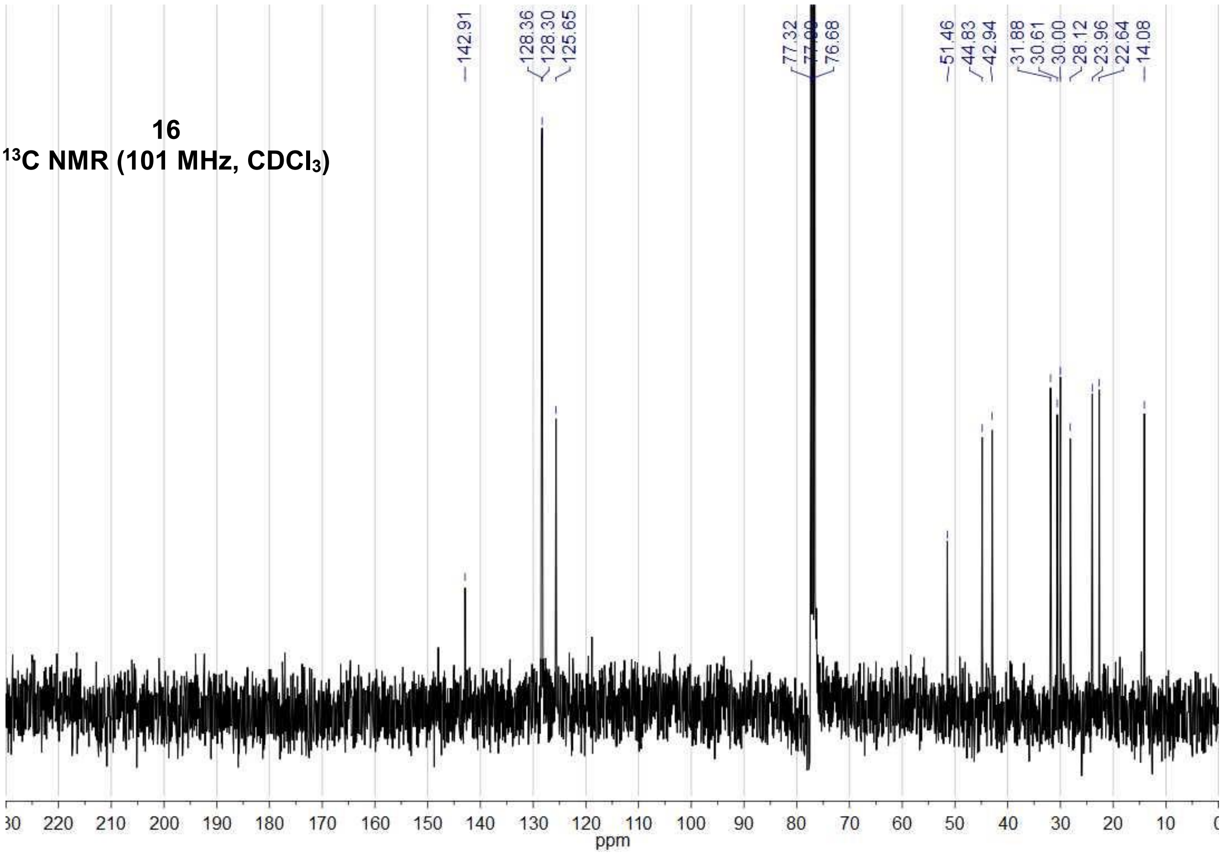

16

^{13}C NMR (101 MHz, $CDCl_3$)

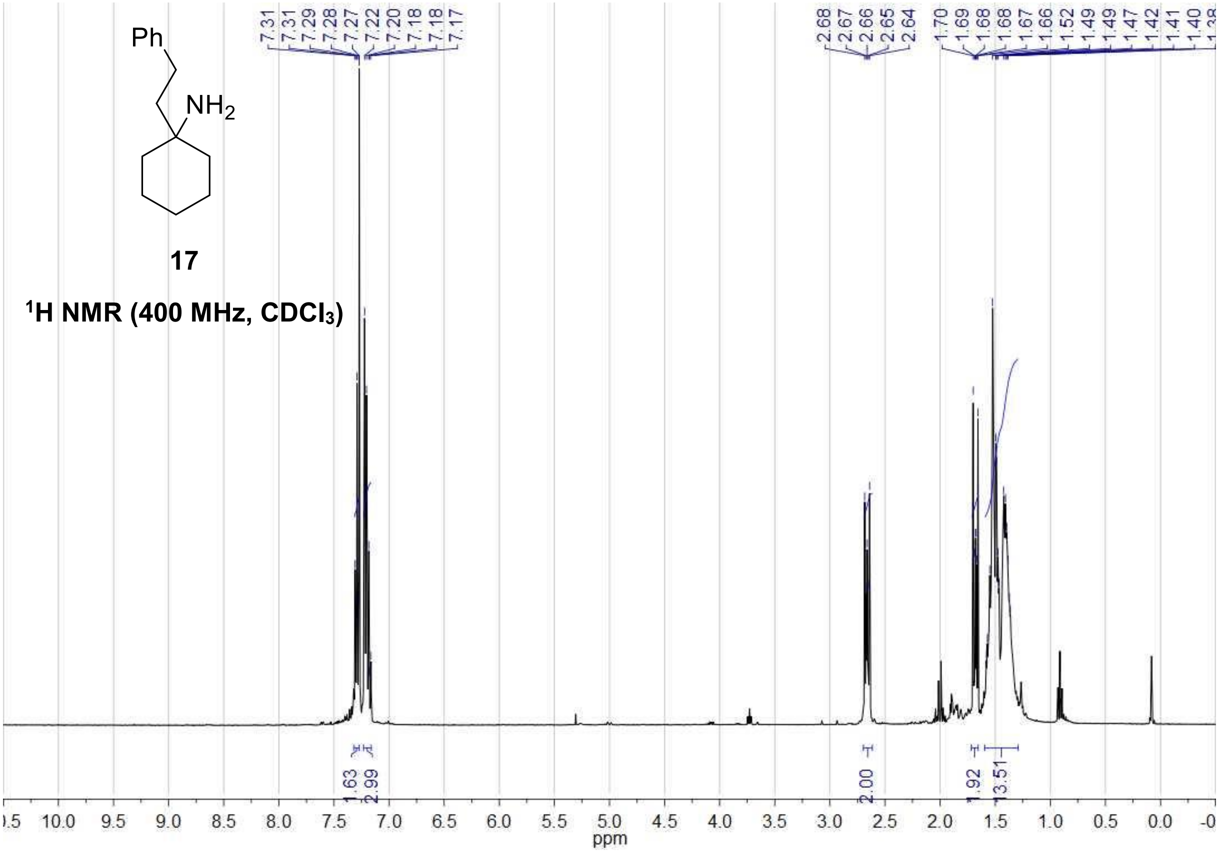
7.31 7.31 7.29 7.28 7.27 7.22 7.20 7.18 7.18 7.17
2.68 2.67 2.66 2.65 2.64
1.70 1.69 1.68 1.68 1.67 1.66 1.52 1.49 1.49 1.47 1.42 1.41 1.40
Ph
NH2
17
1H NMR (400 MHz, CDCl3)
1.63 2.99 2.00 1.92 13.51
ppm

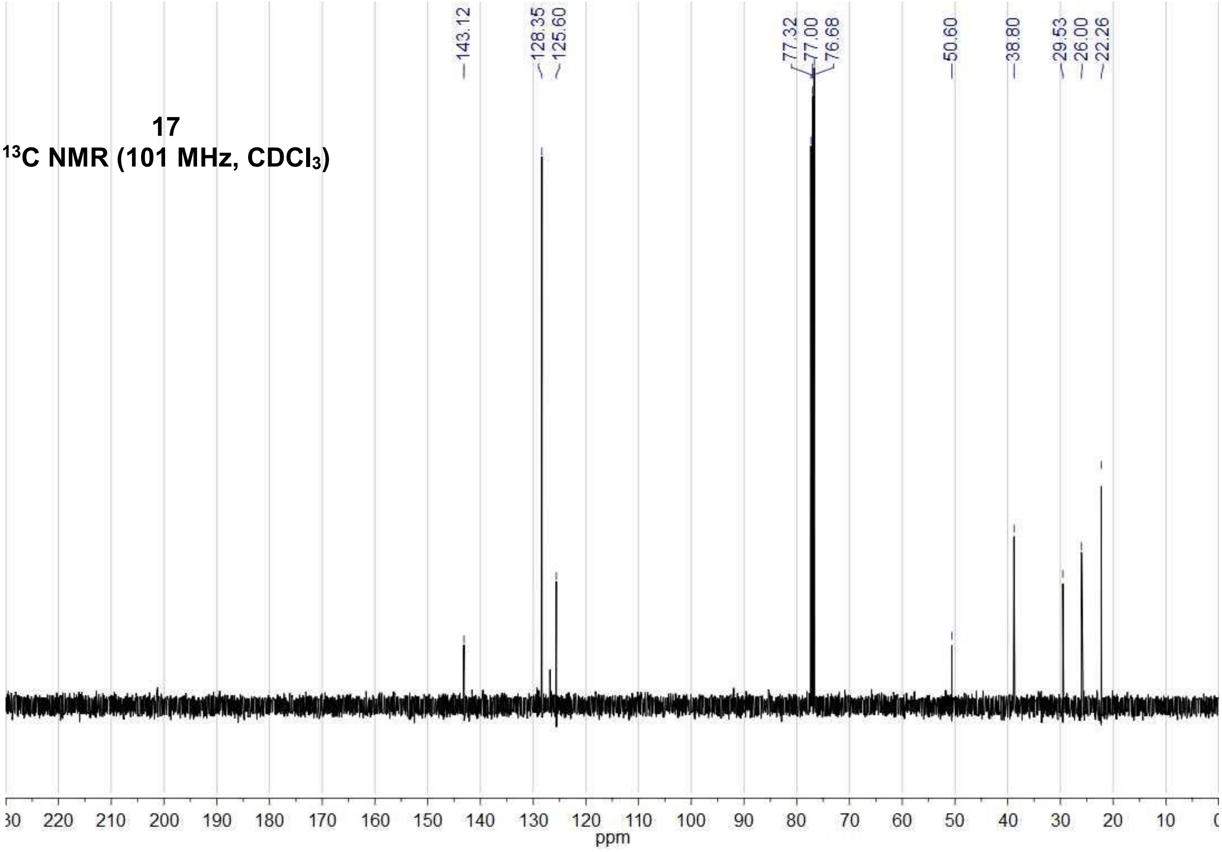
143.12
128.35 125.60
77.32 77.00 76.68
50.60
38.80
29.53 26.00 22.26
17
13C NMR (101 MHz, CDCl3)
ppm

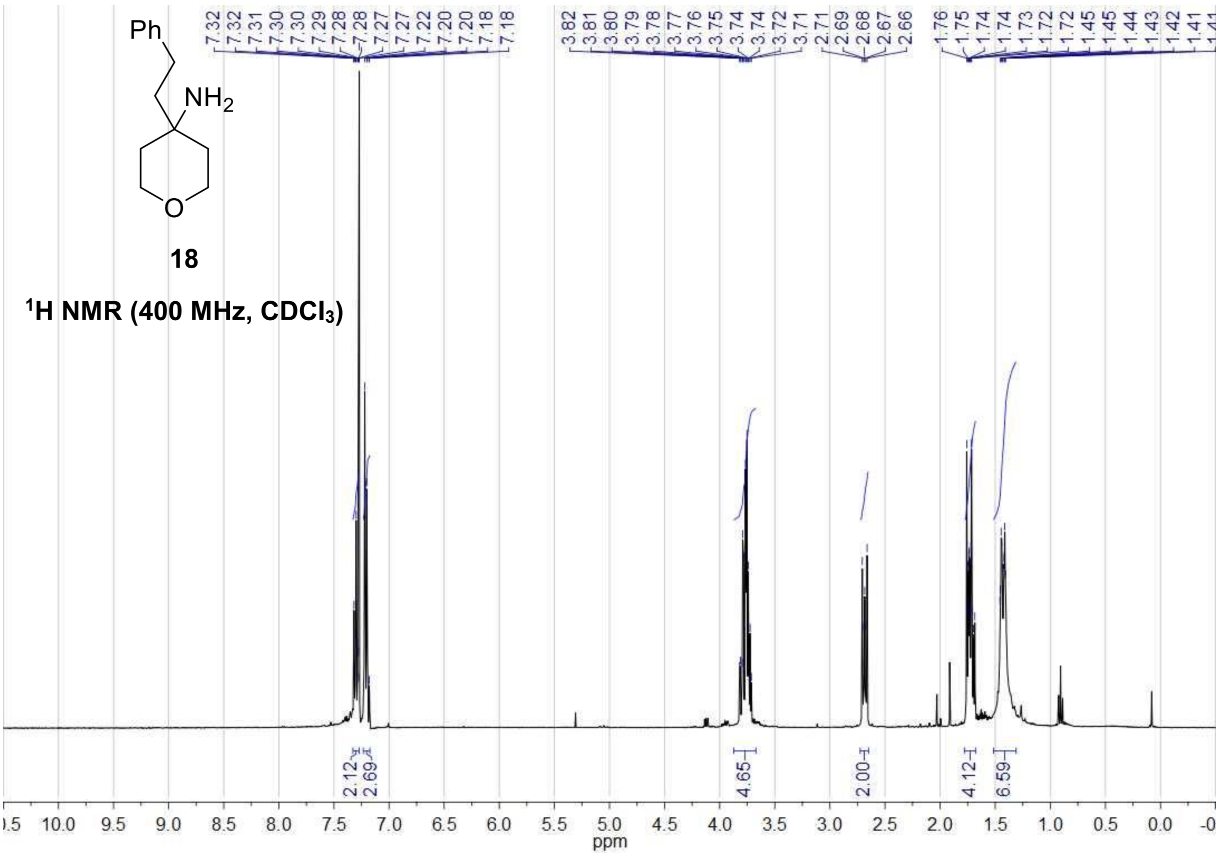
Ph
NH2
O
18
1H NMR (400 MHz, CDCl3)
2.12
2.69
4.65
2.00
4.12
6.59
ppm

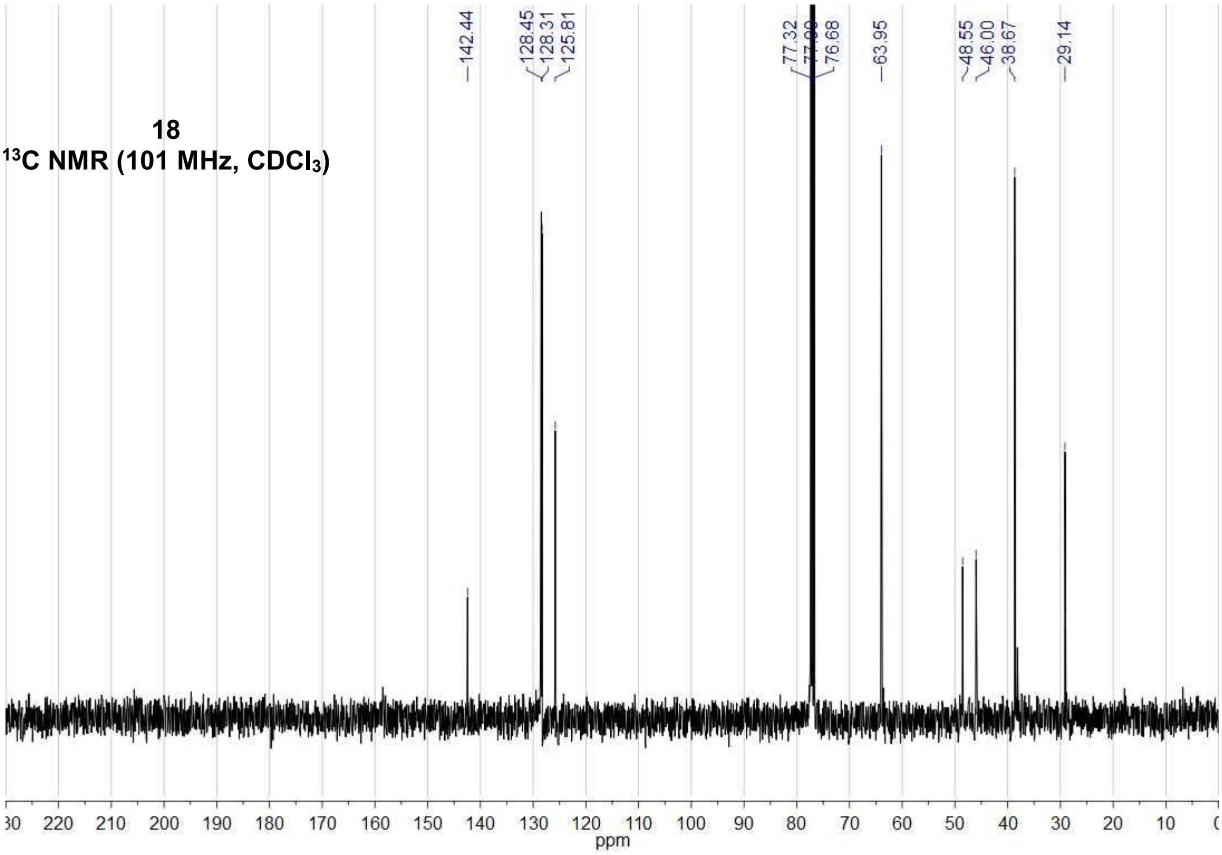
18
13C NMR (101 MHz, CDCl3)
142.44
128.45
128.31
125.81
77.32
76.68
63.95
48.55
46.00
38.67
29.14
ppm

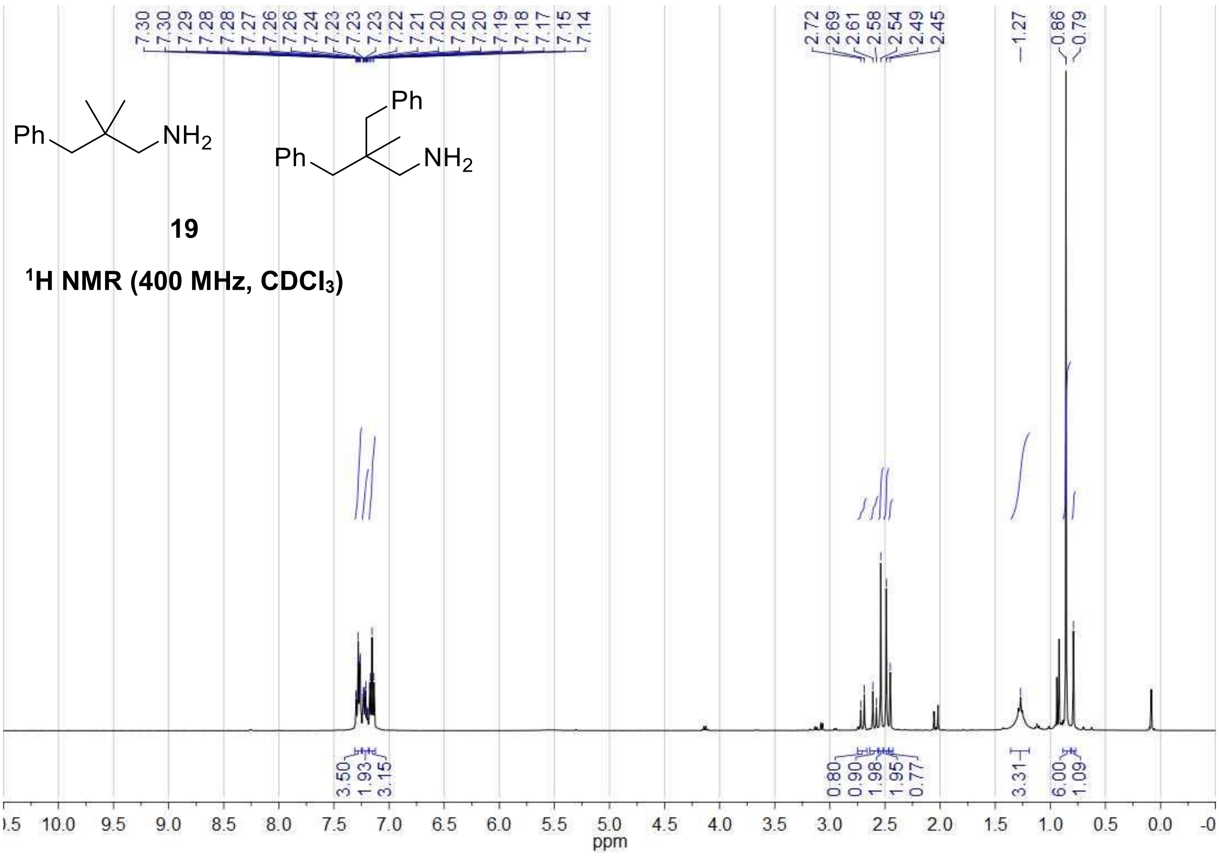

19

^{1}H NMR (400 MHz, CDCl$_3$)

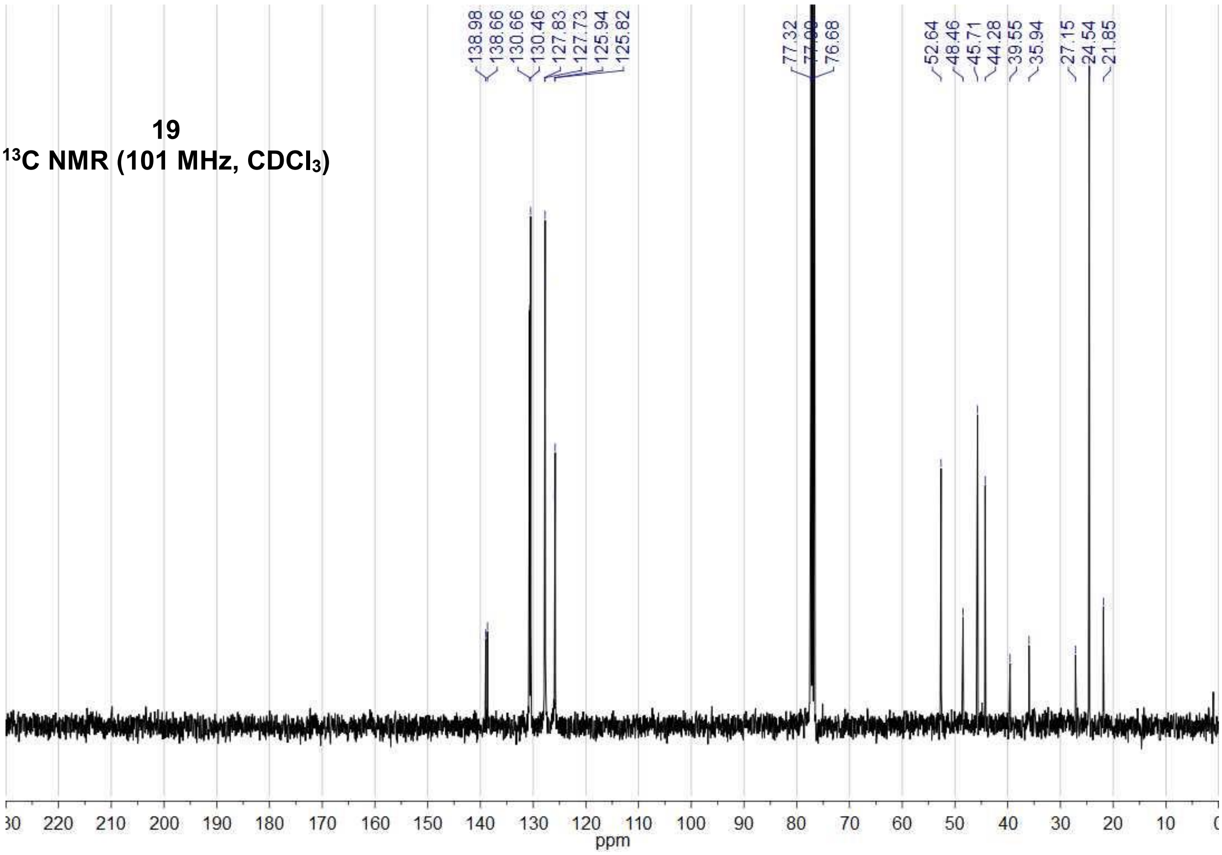

19

^{13}C NMR (101 MHz, CDCl$_3$)

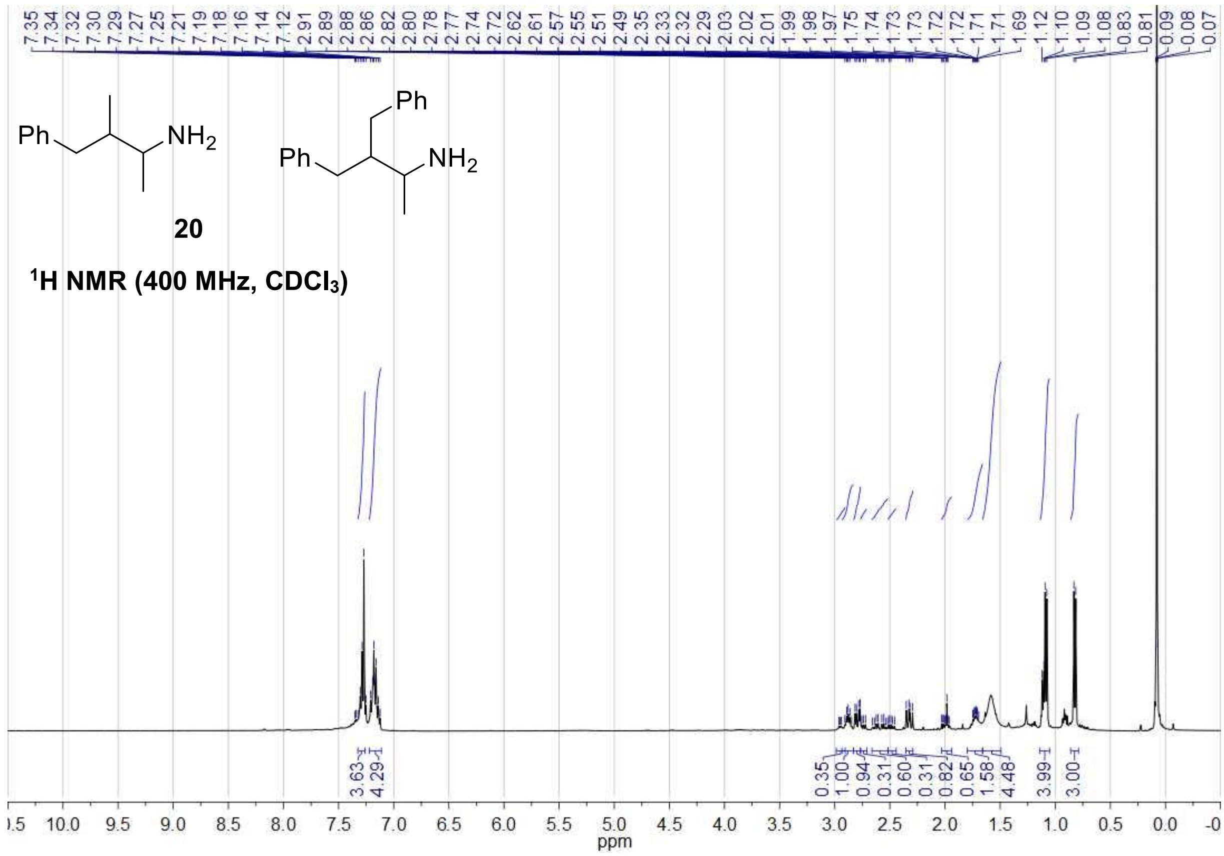
Ph
NH2
Ph
Ph
NH2
20
1H NMR (400 MHz, CDCl3)
ppm

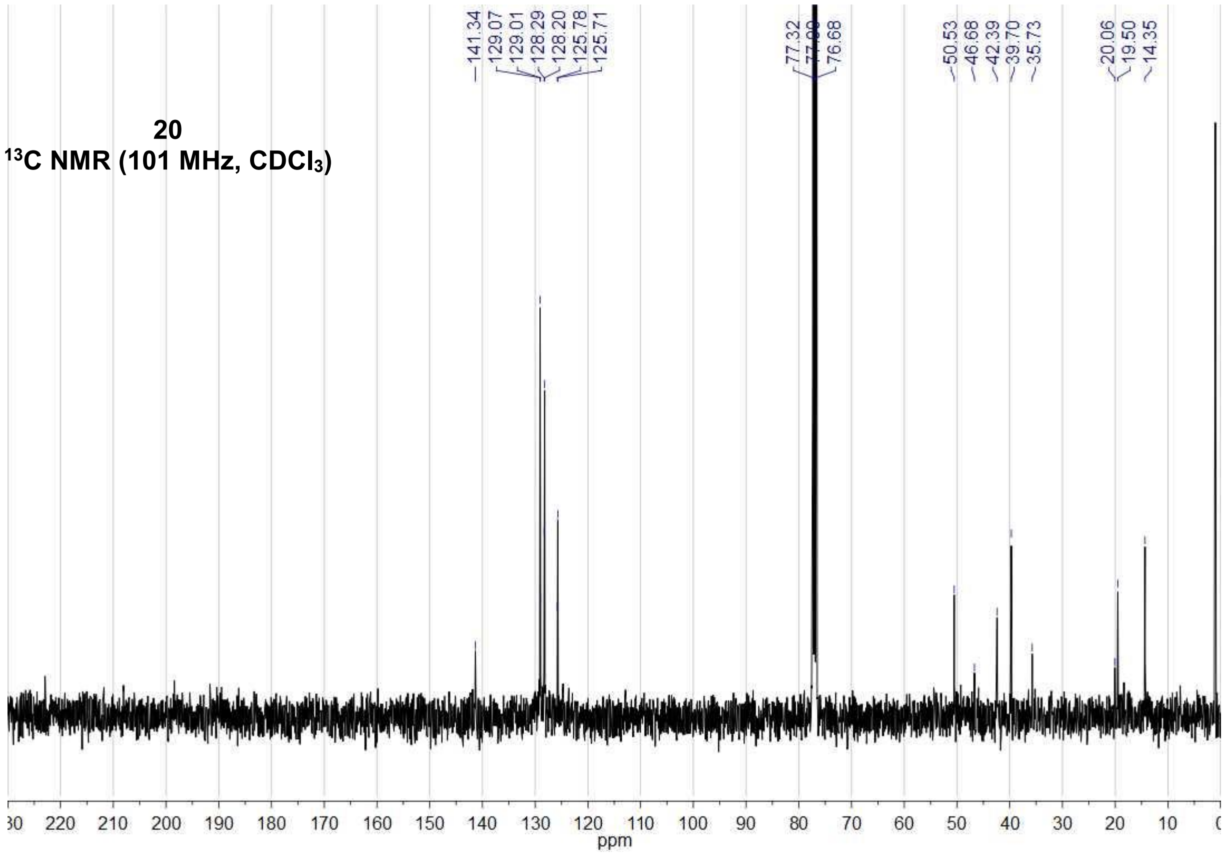
20
13C NMR (101 MHz, CDCl3)
141.34
129.07
129.01
128.29
128.20
125.78
125.71
77.32
76.68
50.53
46.68
42.39
39.70
35.73
20.06
19.50
14.35
ppm

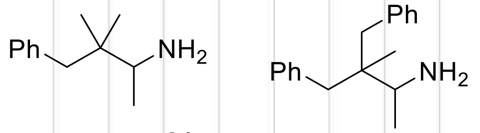

21

^{1}H NMR (400 MHz, $CDCl_3$)

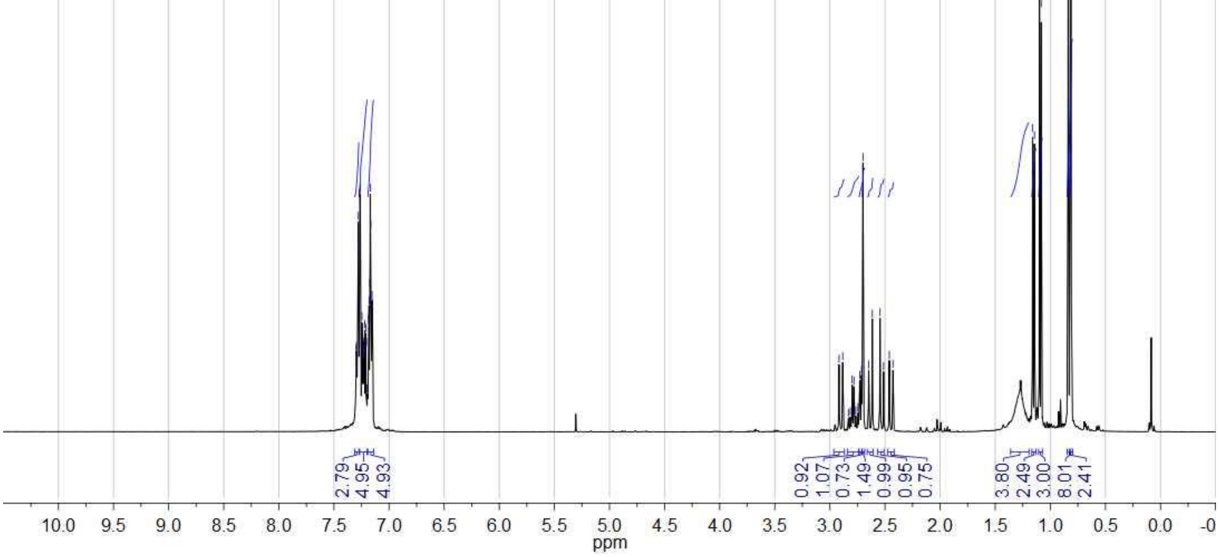

21

^{13}C NMR (101 MHz, $CDCl_3$)

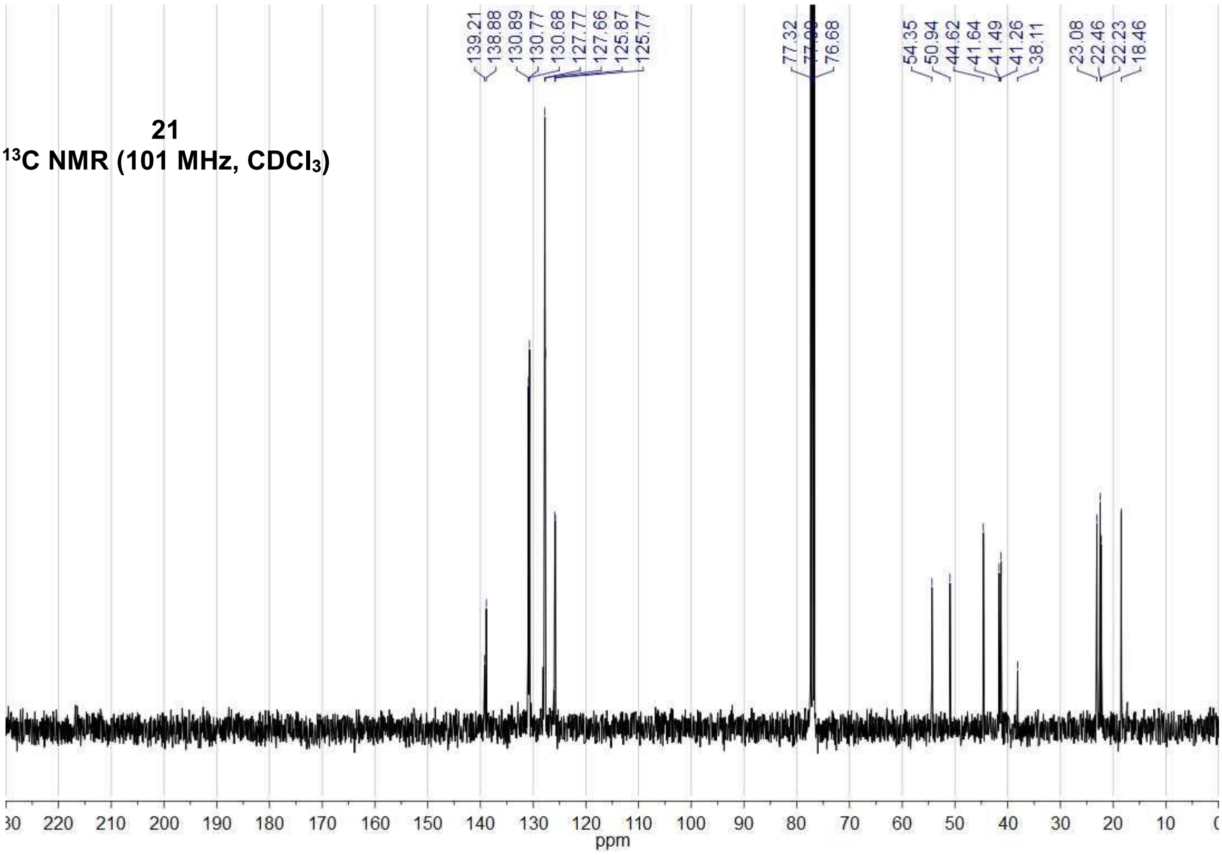

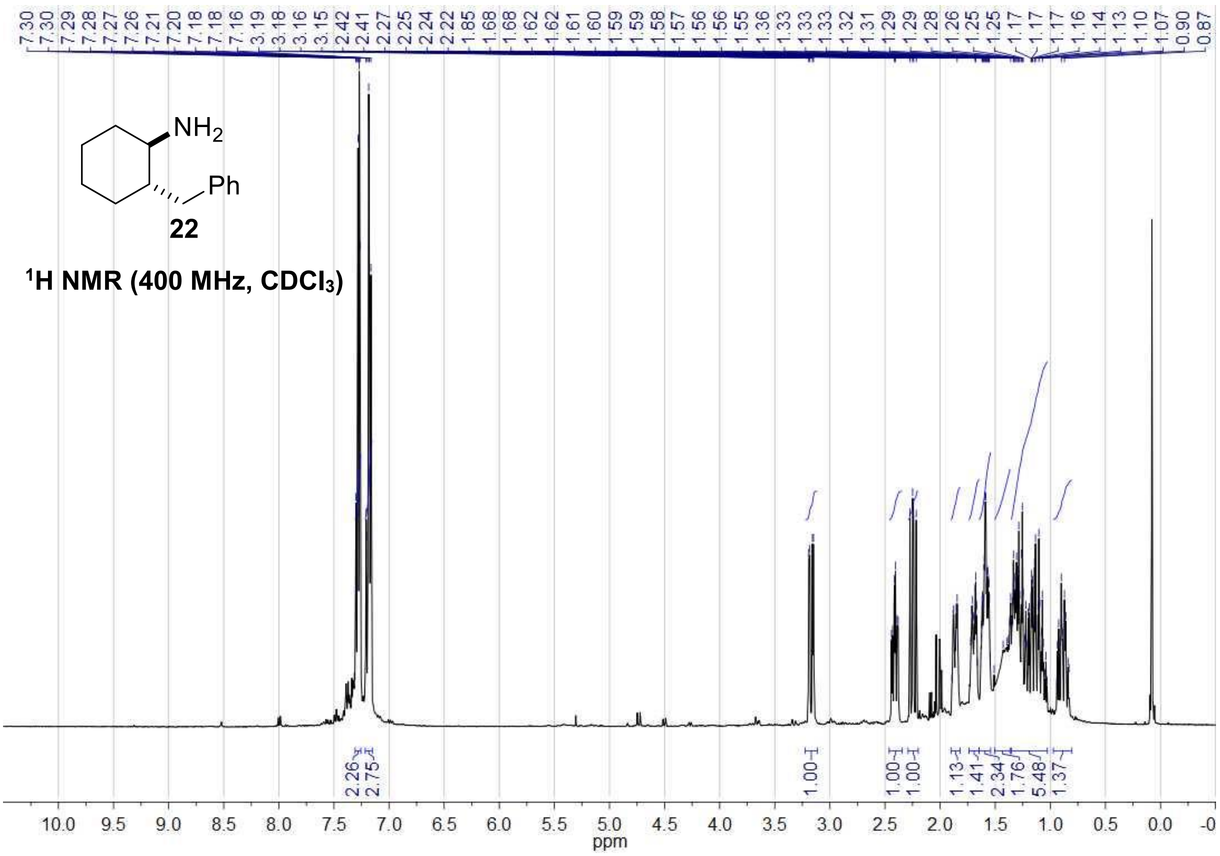
NH2
Ph
22
1H NMR (400 MHz, CDCl3)
7.30 7.30 7.29 7.28 7.27 7.26 7.21 7.20 7.18 7.18 7.16 3.19 3.18 3.16 3.15 2.42 2.41 2.27 2.25 2.24 2.22 1.85 1.68 1.68 1.62 1.62 1.61 1.60 1.59 1.59 1.58 1.57 1.56 1.56 1.55 1.36 1.33 1.33 1.33 1.32 1.31 1.29 1.29 1.28 1.26 1.25 1.25 1.17 1.17 1.17 1.16 1.14 1.13 1.10 1.07 0.90 0.87
2.26 2.75 1.00 1.00 1.00 1.13 1.41 2.34 1.76 5.48 1.37
10.0 9.5 9.0 8.5 8.0 7.5 7.0 6.5 6.0 5.5 5.0 4.5 4.0 3.5 3.0 2.5 2.0 1.5 1.0 0.5 0.0
ppm

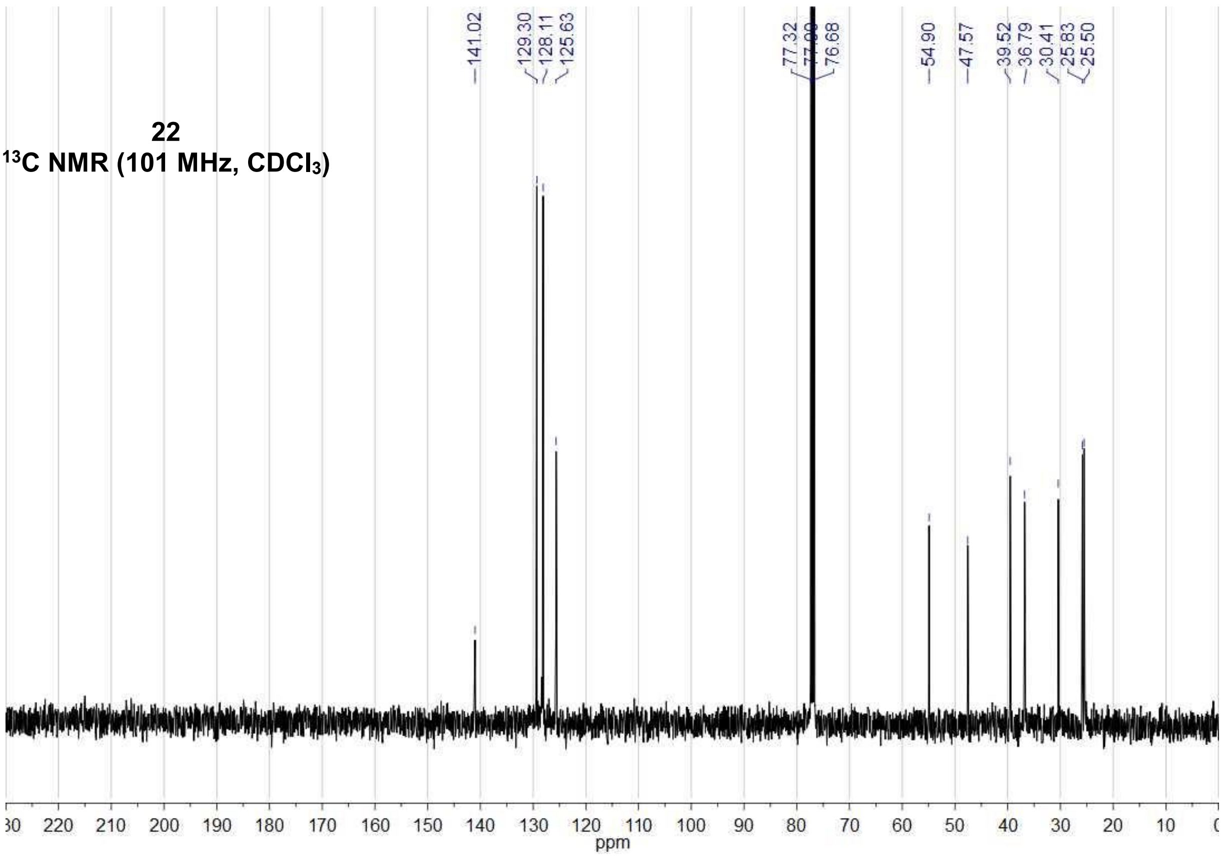
22
13C NMR (101 MHz, CDCl3)
141.02
129.30 128.11 125.63
77.32 76.68
54.90
47.57
39.52 36.79 30.41 25.83 25.50
220 210 200 190 180 170 160 150 140 130 120 110 100 90 80 70 60 50 40 30 20 10
ppm

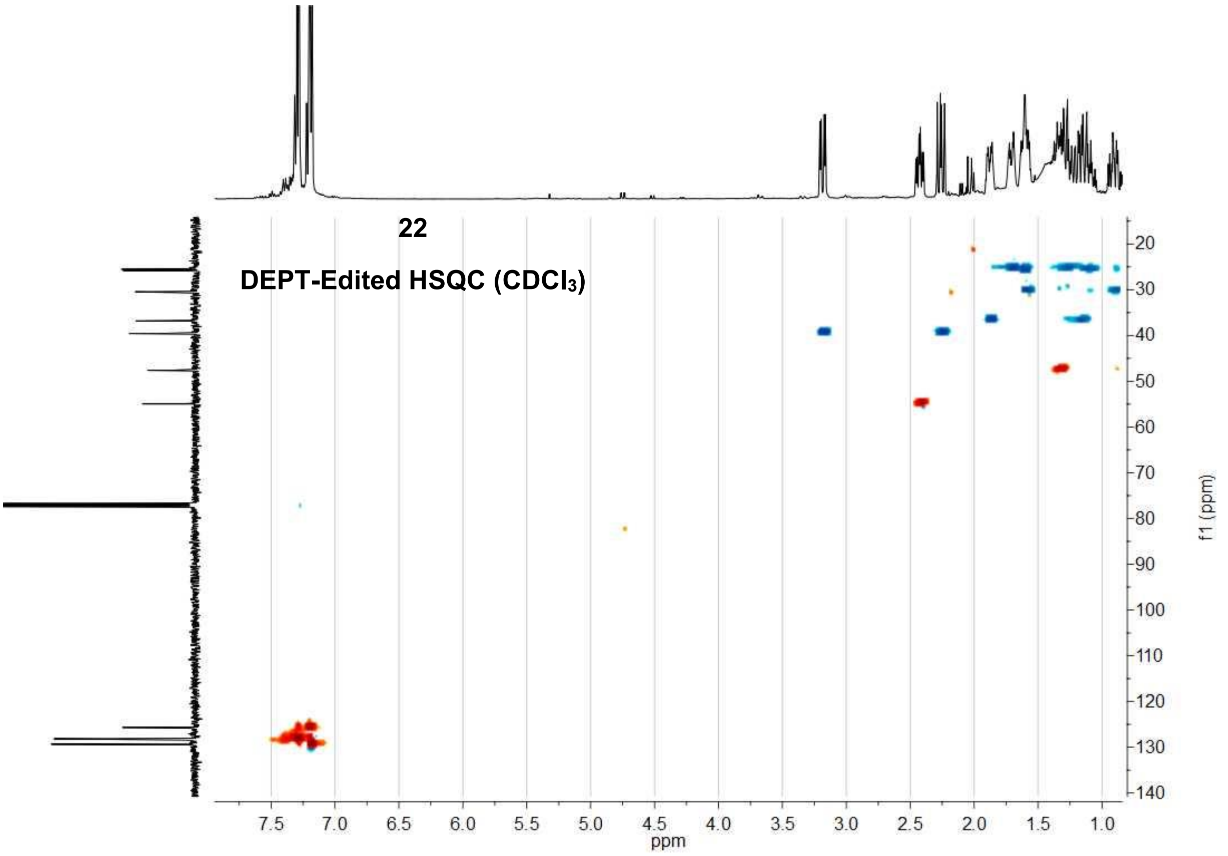
22
DEPT-Edited HSQC (CDCl3)
ppm
f1 (ppm)

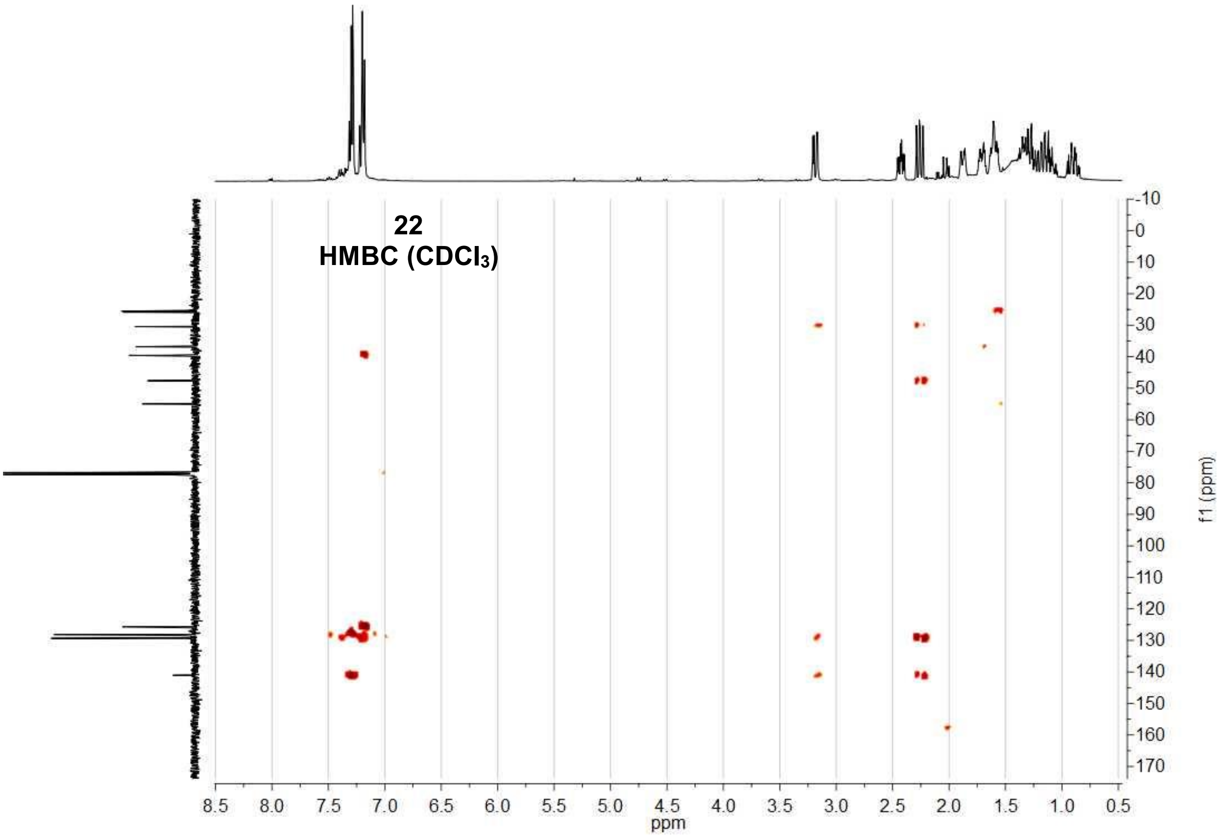
22
HMBC (CDCl3)
ppm
f1 (ppm)

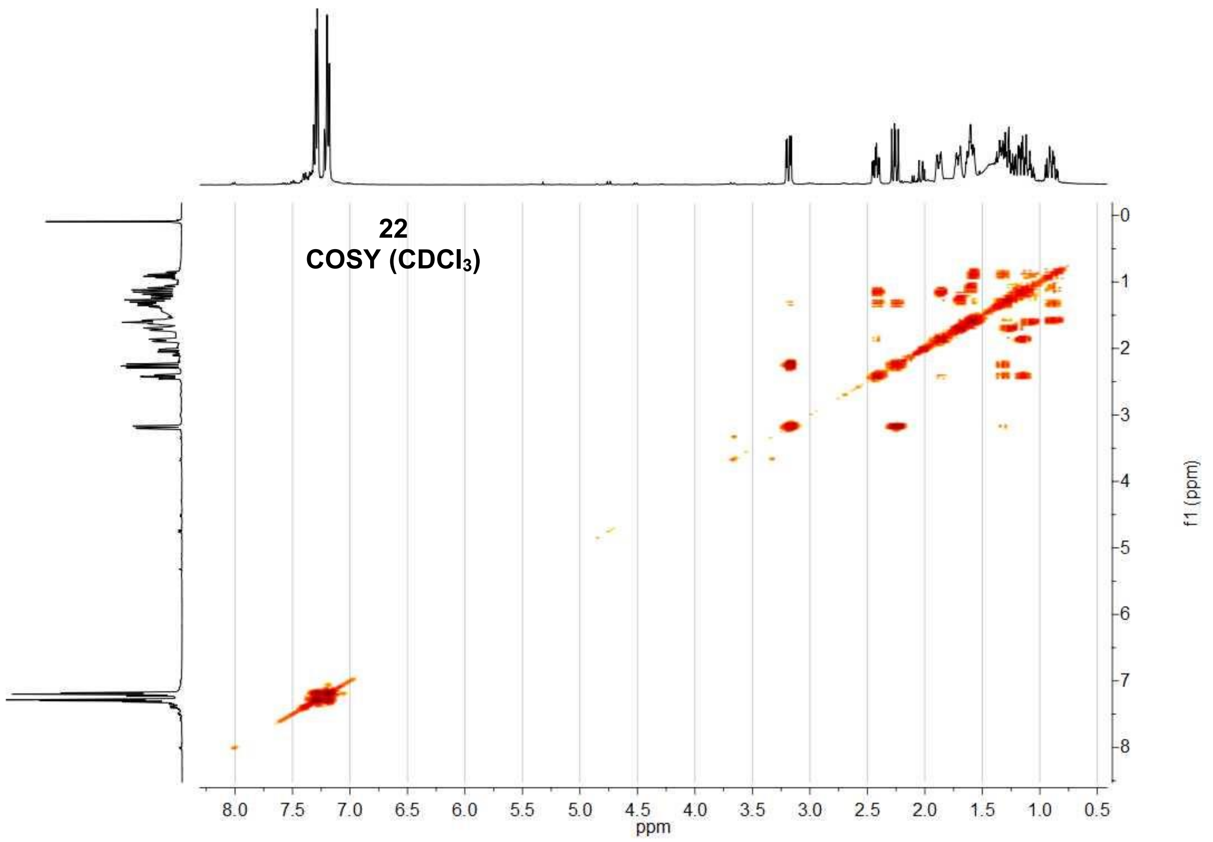
22
COSY ($CDCl_3$)
0
1
2
3
4
5
6
7
8
f1 (ppm)
8.0
7.5
7.0
6.5
6.0
5.5
5.0
4.5
4.0
3.5
3.0
2.5
2.0
1.5
1.0
0.5
ppm

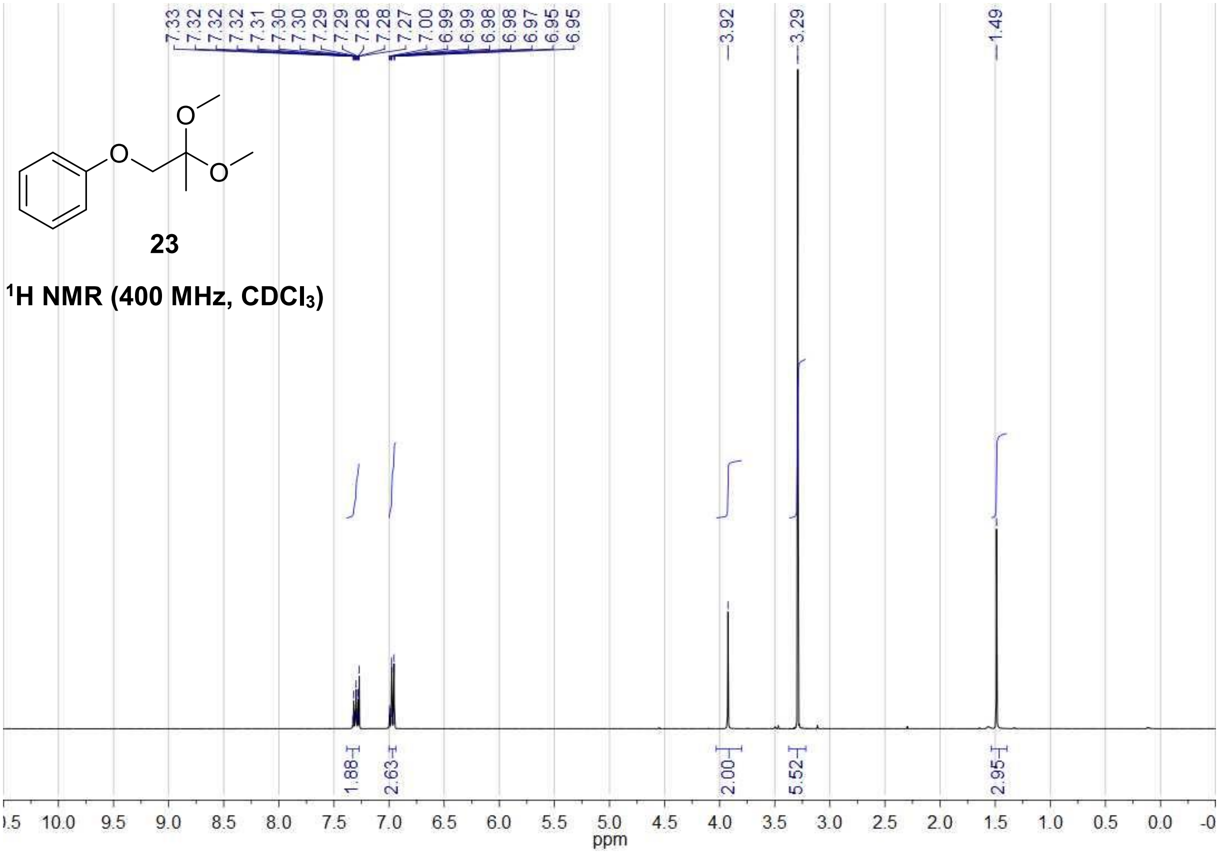

23

^{1}H NMR (400 MHz, $CDCl_3$)

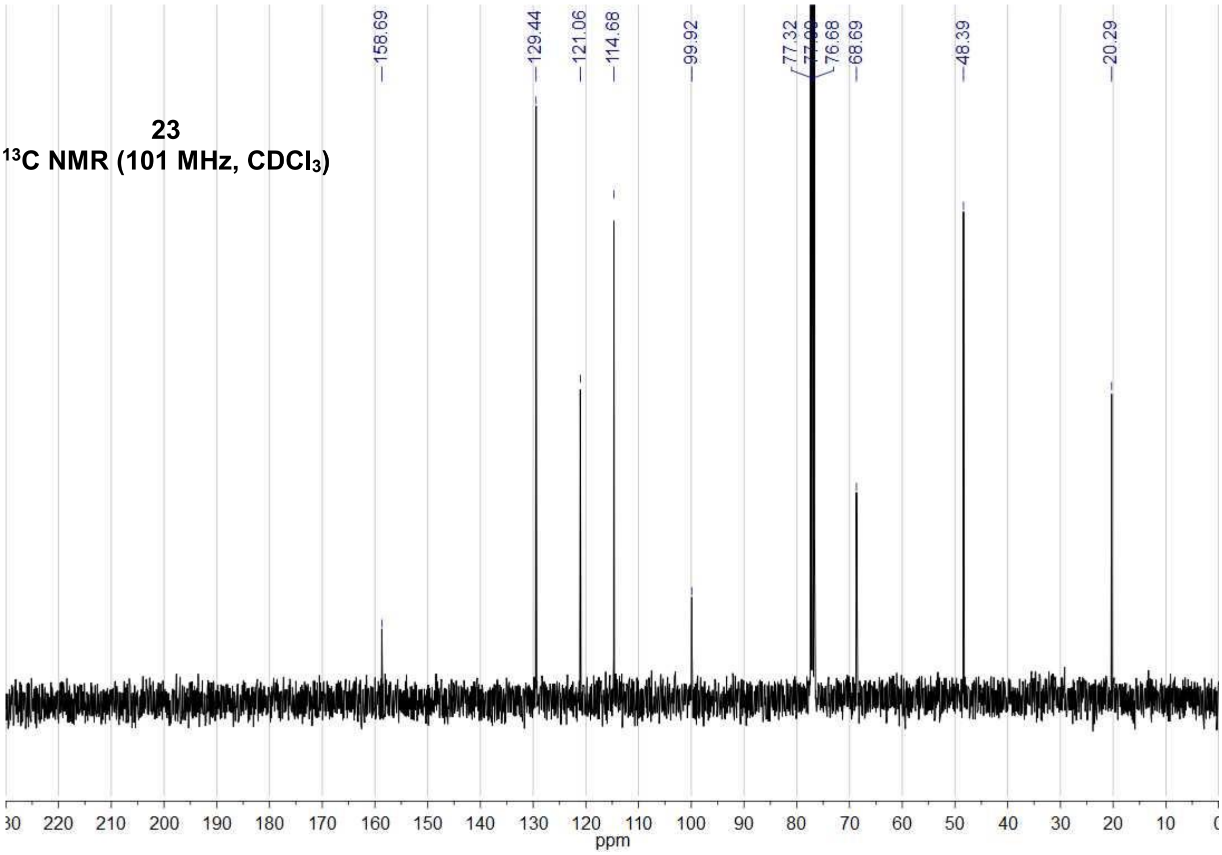

23

^{13}C NMR (101 MHz, $CDCl_3$)

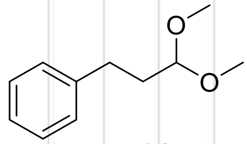

24

^{1}H NMR (400 MHz, $CDCl_3$)

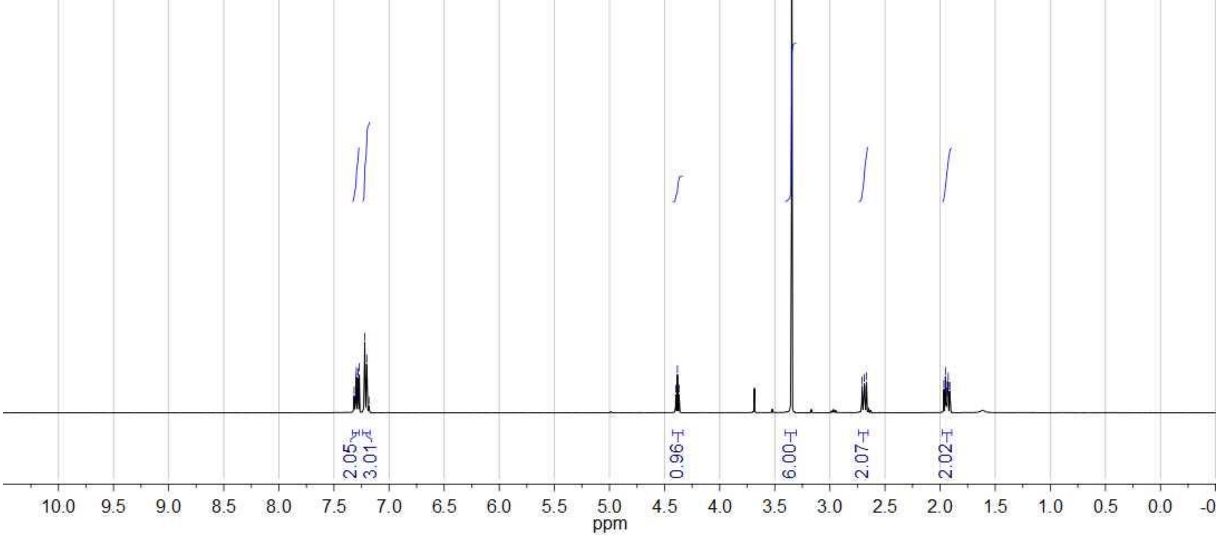

24

^{13}C NMR (101 MHz, $CDCl_3$)

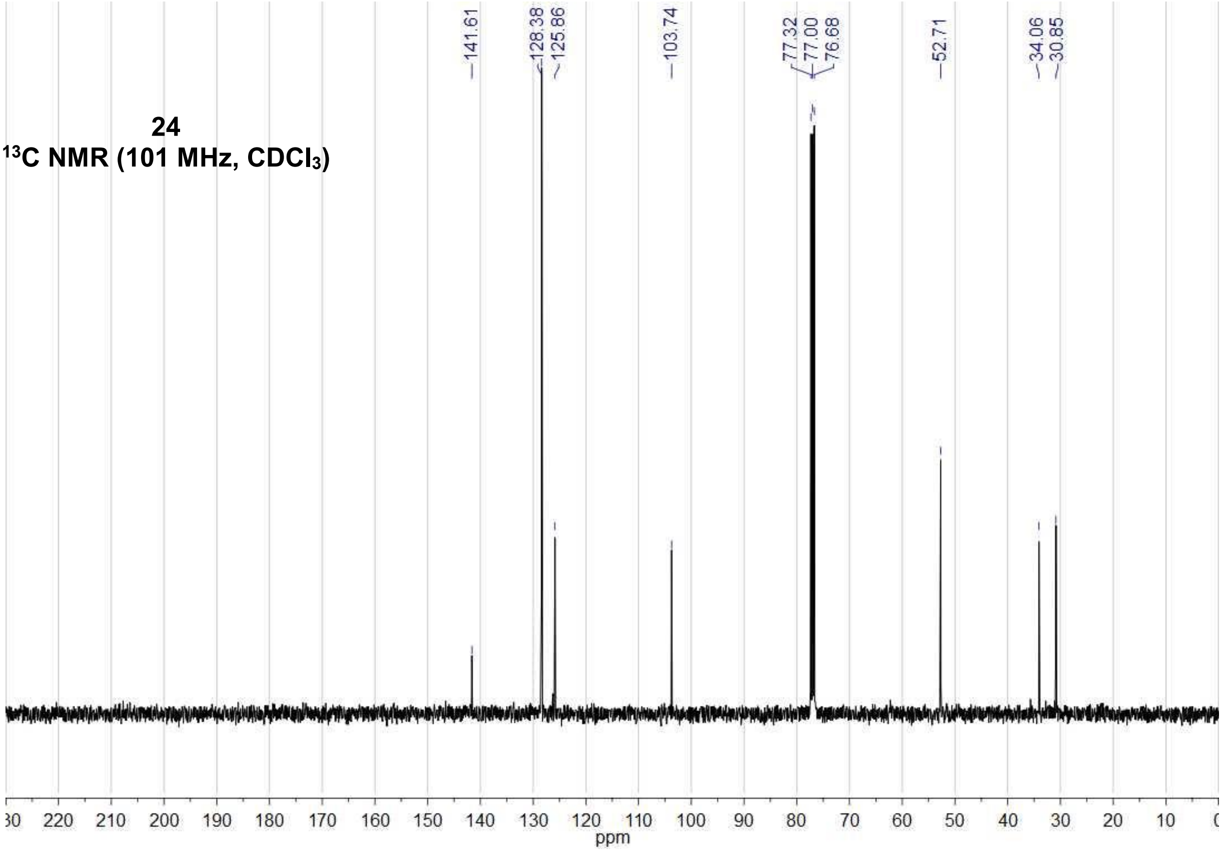

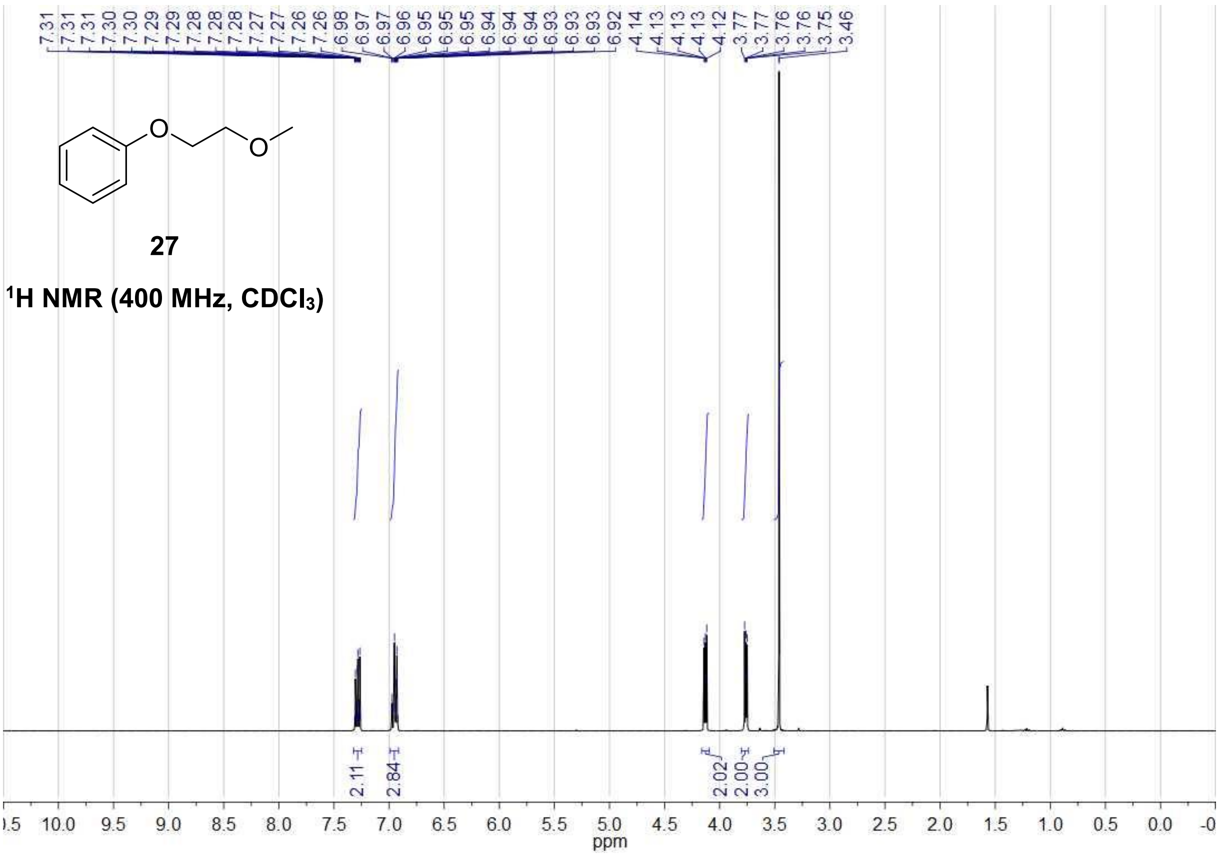
7.31 7.31 7.30 7.30 7.29 7.29 7.28 7.28 7.28 7.27 7.27 7.26 7.26 6.98 6.97 6.97 6.96 6.95 6.95 6.95 6.94 6.94 6.94 6.93 6.93 6.93 6.92 4.14 4.13 4.13 4.13 4.12 3.77 3.77 3.76 3.76 3.75 3.46
27
^{1}H NMR (400 MHz, CDCl$_3$)
2.11 2.84 2.02 2.00 3.00
10.0 9.5 9.0 8.5 8.0 7.5 7.0 6.5 6.0 5.5 5.0 4.5 4.0 3.5 3.0 2.5 2.0 1.5 1.0 0.5 0.0
ppm

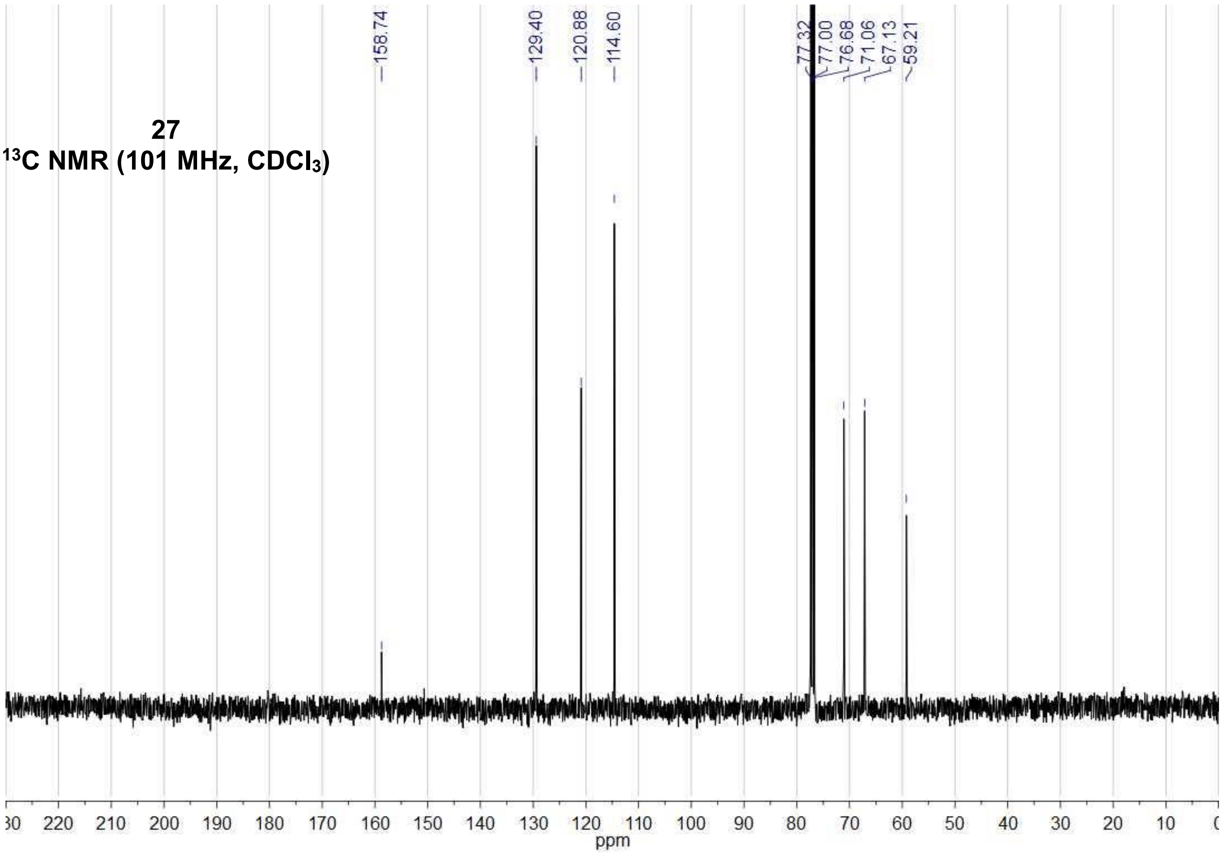
158.74
129.40 120.88 114.60
77.32 77.00 76.68 71.06 67.13 59.21
27
^{13}C NMR (101 MHz, CDCl$_3$)
220 210 200 190 180 170 160 150 140 130 120 110 100 90 80 70 60 50 40 30 20 10
ppm

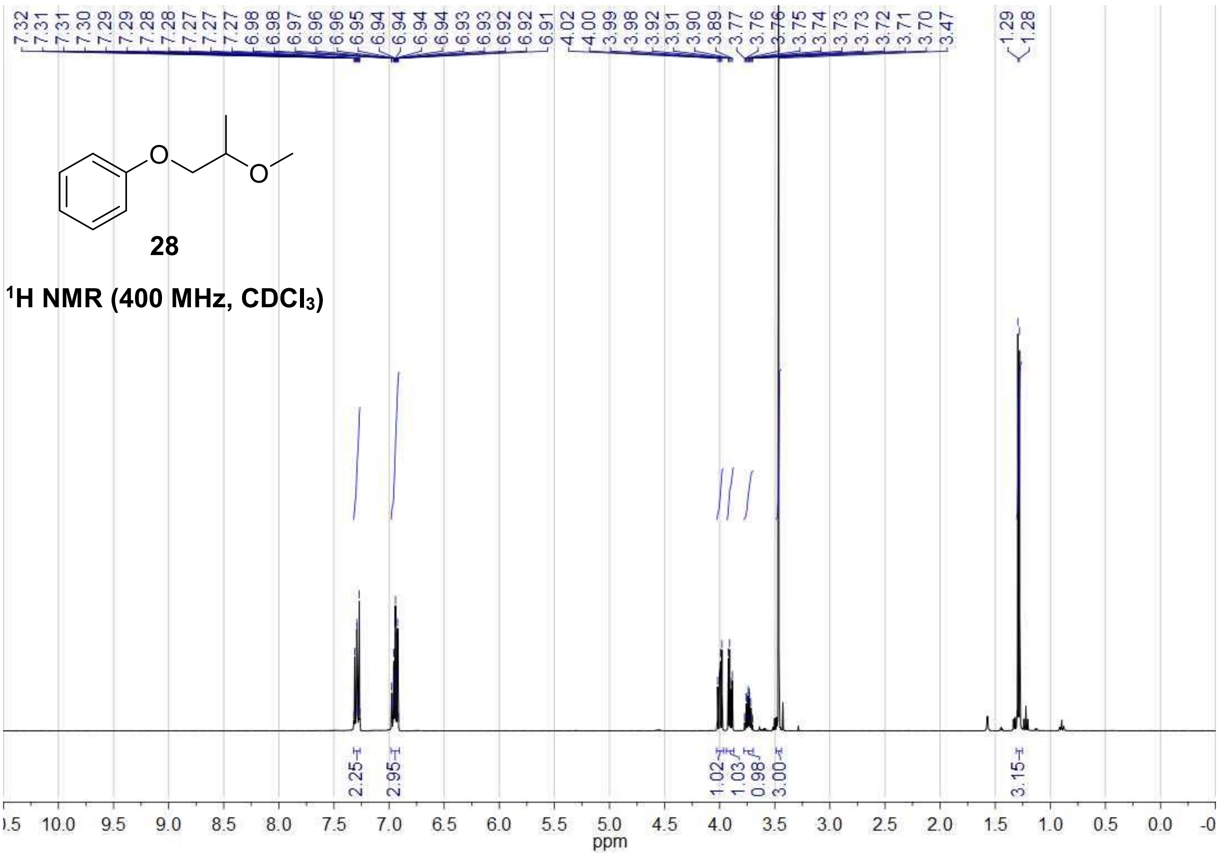
28
1H NMR (400 MHz, CDCl3)
ppm

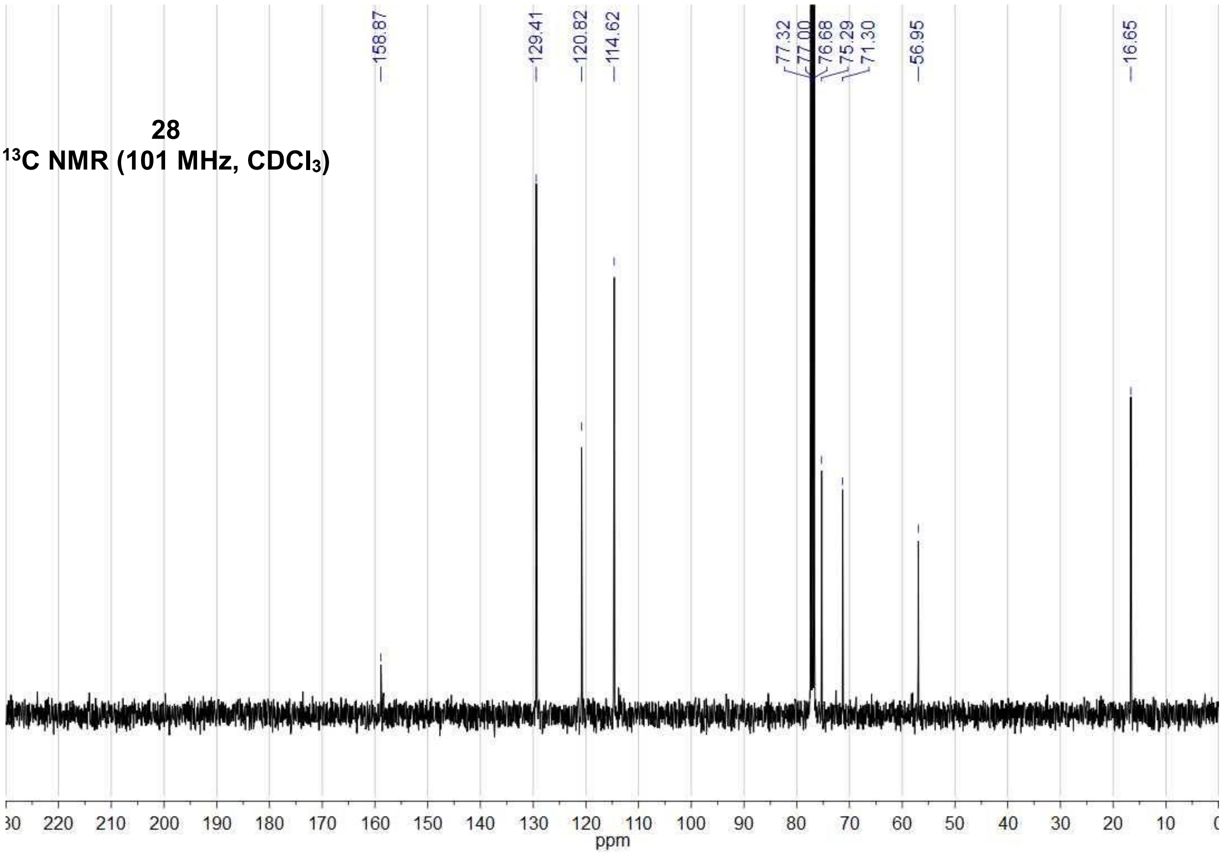
158.87
129.41
120.82
114.62
77.32
77.00
76.68
75.29
71.30
56.95
16.65
28
13C NMR (101 MHz, CDCl3)
ppm

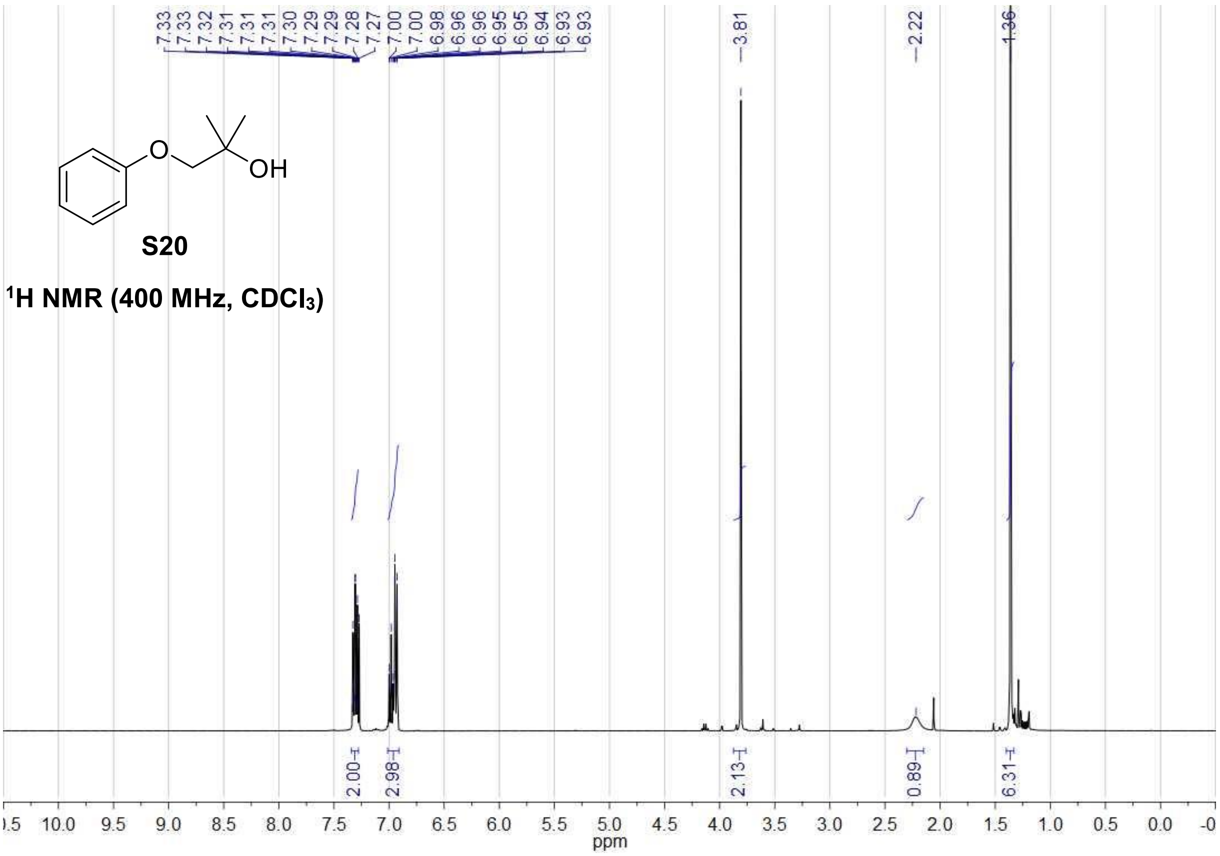
S20
¹H NMR (400 MHz, CDCl₃)
7.33 7.33 7.32 7.31 7.31 7.31 7.30 7.29 7.29 7.28 7.27 7.00 7.00 6.98 6.96 6.96 6.95 6.95 6.94 6.93 6.93
3.81
2.22
1.36
2.00 2.98 2.13 0.89 6.31
ppm

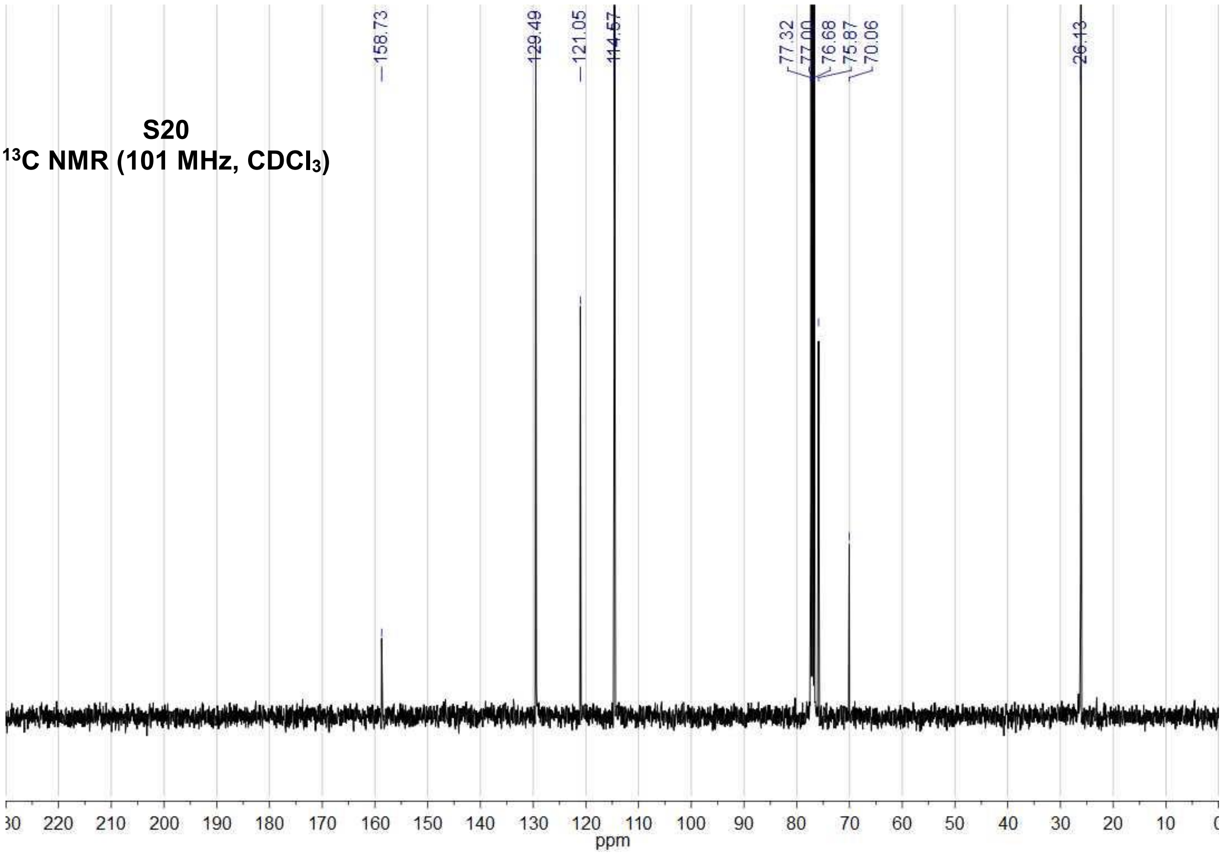
S20
¹³C NMR (101 MHz, CDCl₃)
158.73
129.49
121.05
114.57
77.32 77.00 76.68 75.87 70.06
26.13
ppm

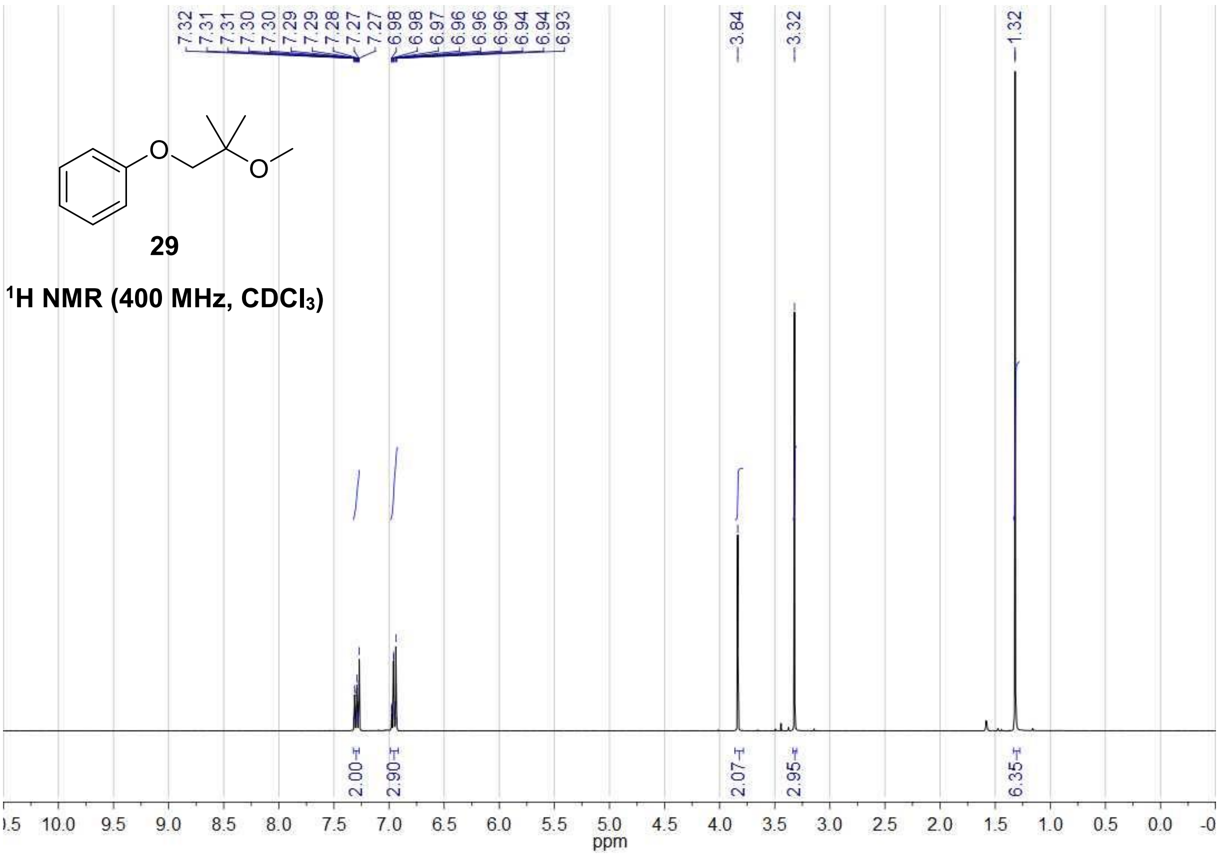
29
1H NMR (400 MHz, CDCl3)
7.32 7.31 7.31 7.30 7.30 7.29 7.29 7.28 7.27 7.27 6.98 6.98 6.97 6.96 6.96 6.96 6.94 6.94 6.93
3.84
3.32
1.32
2.00
2.90
2.07
2.95
6.35
ppm

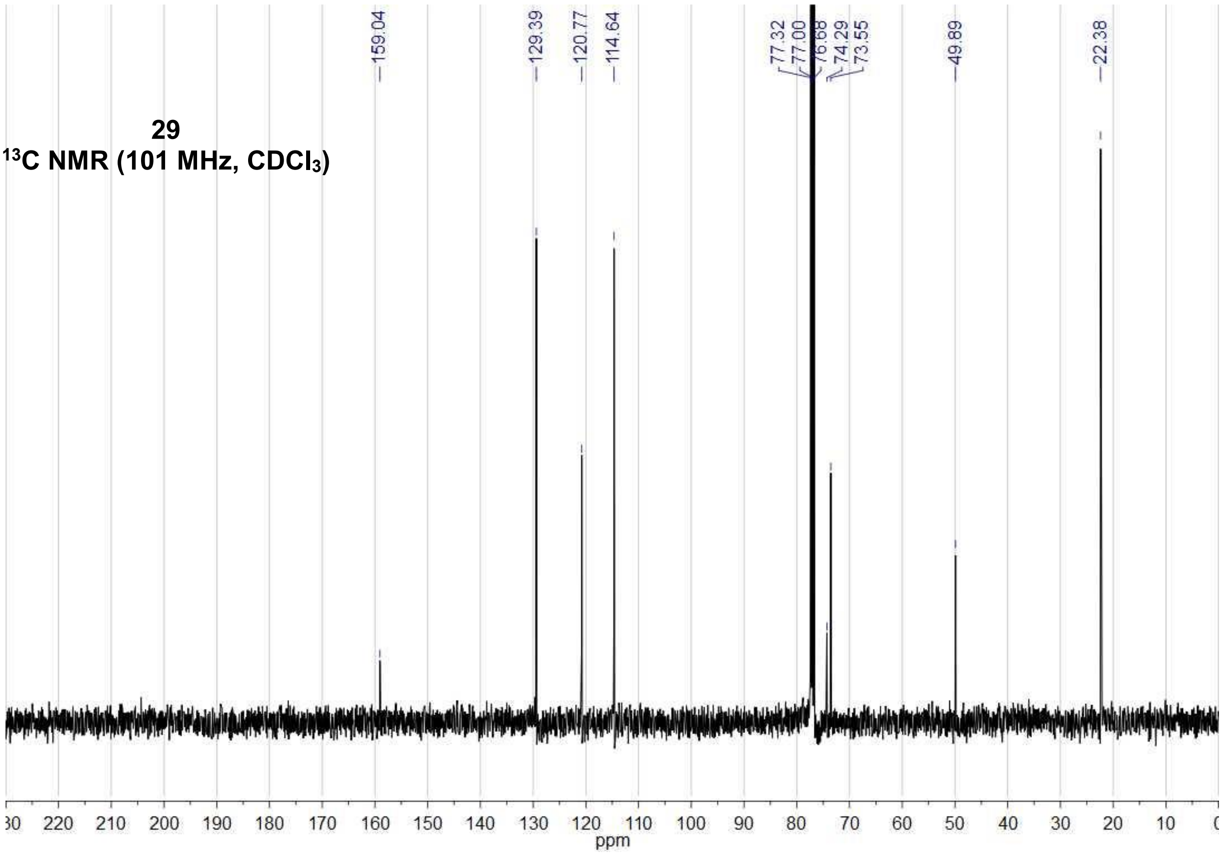
29
13C NMR (101 MHz, CDCl3)
159.04
129.39
120.77
114.64
77.32 77.00 76.68 74.29 73.55
49.89
22.38
ppm
